# Evidence from ileum and liver transcriptomes of resistance to high-salt and water-deprivation conditions in camel

**DOI:** 10.1186/s40851-020-00159-3

**Published:** 2020-06-05

**Authors:** Dong Zhang, Jing Pan, Huanmin Zhou, Yu Cao

**Affiliations:** 1 College of Life Sciences, Inner Mongolia Agricultural University, No. 306 Zhaowuda Road, Hohhot, 010018 P.R. China; 2 Institute of Traditional Chinese Medicine, Tianjin University of Traditional Chinese Medicine, No. 10 Poyanghu Road, Tianjin, 301617 P.R. China

**Keywords:** *Camelus bactrianus*, Ileum, Liver, Salt stress, Water-deprivation stress

## Abstract

Camels have evolved various resistance characteristics adaptive to their desert habitats. In the present study, we used high-throughput sequencing to investigate stress-induced alternative splicing events as well as different genes involved in resistance to water deprivation and salt absorption in the ileum and liver in *Camelus bactrianus*. Through association analyses of mRNA, miRNA and lncRNA, we sought to explicate how camels respond to high salt and water scarcity conditions. There were two modes by which genes driven by alternative splicing were enriched to molecular functions, invoking of which was potentially fixed by organ and stress types. With qRT-PCR detection, the differentially expressed *MUC6*, *AQP5*, *LOC105076960*, *PKP4*, *CDH11*, *TENM1, SDS*, *LOC105061856*, *PLIN2* and *UPP2* were screened as functionally important genes, along with miR-29b, miR-484, miR-362-5p, miR-96, miR-195, miR-128 and miR-148a. These genes contributed to cellular stress resistance, for instance by reducing water loss, inhibiting excessive import of sodium, improving protective barriers and sodium ion homeostasis, and maintaining uridine content. The underlying competing endogenous RNAs referred to LNC001664, let-7e and *LOC105076960* mRNA in ileum, and LNC001438, LNC003417, LNC001770, miR-199c and *TENM1* mRNA in liver. Besides competent interpretation to resistance, there may be inspirations for curing human diseases triggered by high-salt intake.

## Background

Over the course of evolution, desert camels have developed many adaptive characteristics supporting resistance to heat, salt, and dry environments [1, 2]. Prior studies have reported that camels respond to high-salt and water shortage conditions by increased sodium excretion under saline loading [3], production of highly concentrated urine [4], and efficient water reservation by the kidneys [5]. Notably, previous studies on camels have focused on the role of the kidney in resistance to water deprivation, but neglected other organs, such as liver and intestine. Investigations in humans and other model species have shown that the intestinal tract is widely coupled to the regulation of salt and water metabolism [6]. The ileum is the third and final portion of the small intestine, primary function of which is to absorb minerals and nutrients from ingested food [7, 8]. The ileum forms a peritoneal structure with blood vessels, lymphatic vessels, and nerve fibers, suspended within the mesentery. The blood vessels, including the superior mesenteric artery and veins supply the ileum [9]. In rat, sodium is absorbed and transported from mucosa to serosa by the ileum, while water absorption is passive and dependent on osmotic gradients, such as those forced by active salt transport [6, 10, 11]. Additionally, sodium ion absorption is modulated by hormones (e.g. vasopressin and renin) in body fluids and by proteins (e.g. EnAC complex and Na^+^/K^+^ ATP enzymes) in intestinal epithelial cells [12]. In hepatic cells, bile salt export pump and sodium taurocholate cotransporting polypeptide have been identified as salt-related transport proteins [13]. Previous reports have detected higher metal levels in the salt glands of mallard (*Anas platyrhynchos*) and black duck (*Anas rubripes*), as well as in the liver of greater scaup (*Aythya marila*) [14]. However, data on the post-transcriptional regulation of non-coding RNA in the ileum and liver under salt and water-deprivation stresses remain scarce.

Non-coding RNAs (ncRNAs), such as long non-coding RNA (lncRNA) and micro RNA (miRNA), are generally interpreted as RNA molecules that are not translated into proteins [15]. Post-transcriptional regulation mediated by ncRNA is an essential factor on affecting gene expression. Studies have demonstrated that miRNA can inhibit the expression of protein-coding genes by binding to 3′ untranslated regions and protein-coding regions of a targeted mRNA [16, 17]. The competing endogenous RNA (ceRNA) theory posits that lncRNA acts as a molecular sponge for miRNAs and reverses the inhibition protein-coding expression they evoke [18]. In the present study, we exploited these two patterns of miRNA-mRNA and ceRNA to explore regulatory responses to external stresses of salt and water-deprivation in camel.

In addition to illustrating molecular responses in camel organs under stress, we hope to provide novel insights based on scientific evidence to support the development of new treatment strategies for human diseases caused by high-salt diets.

## Materials and methods

### Stress application and sample collection

Nine Alxa bactrian camels were randomly allocated into two experimental groups (salt stress and water-deprivation stress) and one control group (free diet), with three camels per group and a treatment phase of 24 days. The mean ± SD weight of all camels was 350.48 ± 7.68 kg, and the average age was 7.67 ± 0.71 years old. Salt stress (SS) group was treated as follows: salt intake base was 200 g/d, and increased by 100 g every three days [19, 20]. Water-deprivation stress (WS) group was with free feed intake but fasting water. Mercy-killing was performed to nine Alxa bactrian camels by severing the carotid artery after intramuscular injection of 0.5 mg/kg xylazine [21]. Ileum and liver tissues were immediately sampled and stored in 1.5 mL frozen tubes with liquid nitrogen (− 196 °C), respectively.

### RNA isolation and quantification

RNeasy Mini Kit (QIAGEN, Germany) was used to extract the total RNA from ileum and liver tissues of the nine camels. RNA purity, concentration and integrity were detected using the NanoPhotometer spectrophotometer (IMPLEN, CA, USA), Qubit RNA Assay Kit in Qubit 2.0 Fluorometer (Life Technologies, CA, USA) and RNA Nano 6000 Assay Kit with the Agilent Bioanalyzer 2100 system (Agilent Technologies, CA, USA) consecutively.

### Library preparation, clustering and sequencing for lncRNA and small RNA

The amount of 3 μg total RNA per sample was inputted separately to construct libraries of lncRNA and small RNA. For lncRNA library construction, ribosomal RNA was first removed by Epicentre Ribo-zero rRNA Removal Kit (Epicentre, USA), after which libraries were prepared by using NEBNext Ultra Directional RNA Library Prep Kit for Illumina (NEB, USA). NEBNext Multiplex Small RNA Library Prep Set for Illumina (NEB, USA) was used to prepare the small RNA libraries. Clustering of the index-coded samples was executed on a cBot Cluster Generation System using TruSeq PE Cluster Kit v3-cBot-HS (Illumina) for lncRNA, and TruSeq SR Cluster Kit v3-cBot-HS (Illumina) for small RNA. The library constructions of lncRNA and small RNA were performed respectively on an Illumina HiSeq 4000 platform and Illumina HiSeq 2500 platform via pooled RNA-seq [22].

### Analysis of miRNA and lncRNA data

Regarding data processing, quality control, reads mapping to *Camelus bactrianus* genome via TopHat v2.0.9 (parameters: --library-type fr-firststrand) [23], transcript assembly via Cufflinks (parameters: min-frags-per-transfrag = 0) [24], lncRNA and mRNA sorting via CNCI v2 were performed at default parameters [25], CPC 0.9-r2 (parameters: -evalue 1e-10) [26], Pfam Scan v1.3 (parameters: -E 0.001 --domE 0.001 -pfamB) [27, 28], PhyloCSF v20121028 (parameters: --orf = ATGStop; −frames = 3; −removeRefGaps) [29], mRNA and lncRNA annotation via *Camelus bactrianus* and *Bos Taurus* databases, differential expression analysis via Cuffdiff (http://cole-trapnell-lab.github.io/cufflinks/cuffdiff/index.html), target gene prediction via lncRNA gene upstream/downstream 100 kb and Pearson Correlation Coefficient with |Pearson correlation| > 0.95 [30], Gene Ontology (GO) and Kyoto Encyclopedia of Genes and Genomes (KEGG) pathway enrichment analyses of protein-coding genes of differential expression via Enrichr [31, 32], DAVID 6.8 [33, 34] and KOBAS 2.0 (parameters: blastx 1e-10 and padjust: BH) [35] were implemented for lncRNA data analysis. Quality control, reads mapping to *Camelus bactrianus* genome via Bowtie (parameters: -v 0 -k 1) [36], novel miRNA prediction via miREvo (parameters: -i -r -M -m -k -p 10 -g 50,000) [37] and mirdeep2 (parameters: quantifier.pl -p -m -r -y -g 0 -T 10) [38], differential expression analysis via TPM [39] and DEGseq with qvalue < 0.01 and |log2(foldchange)| > 1 [40], small RNA annotation by *Bos taurus* database, target gene prediction via miRanda (parameters: -sc 140 -en − 10 -scale 4 -strict -out) were ran for data analysis of small RNA.

### Quantitative detection

RNA was isolated from ileum and liver tissues of camel via mirVana miRNA Isolation Kit (Ambion, USA). The primers were designed by Primer Express 3.0.1 (Additional file 1: Table S1) and qRT-PCR was performed using 7900 HT Sequence Detection System (ABI, USA), following the manufacturer’s protocol, with ReverTra Ace qPCR RT Kit (TOYOBO, Japan) and Power SYBR Green PCR Master Mix (ABI, USA). The student’s t test was performed to compare the mean values between groups and a *P*-value of less than 0.05 was considered as statistically significant.

## Results

### Differential alternative splicing events under salt and water-deprivation stresses

**Fig. 1:**
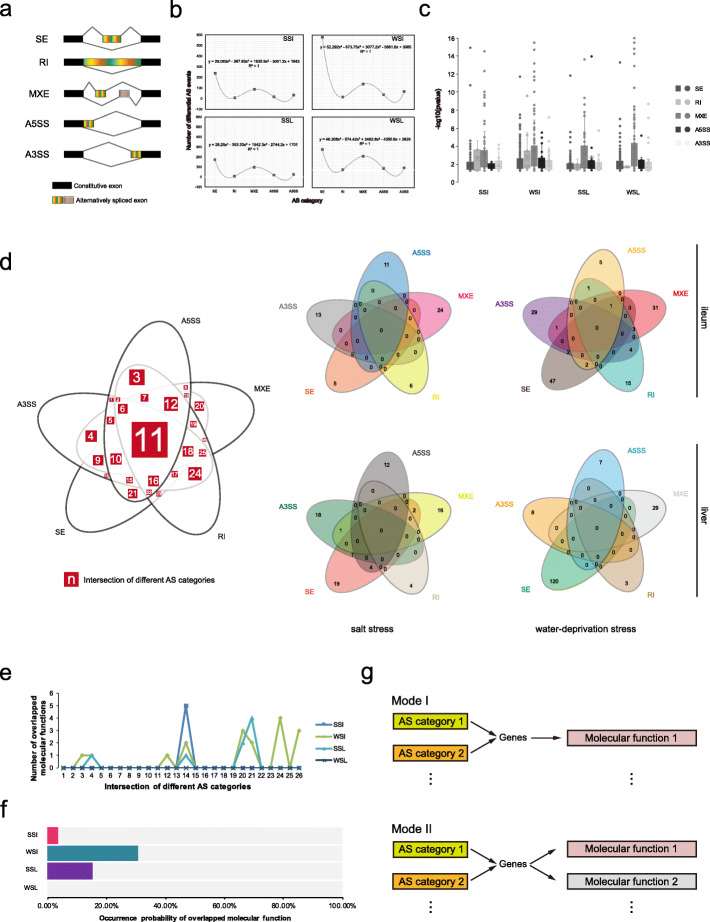
Alternative splicing events in the ileum and liver of camel under salt stress and water-deprivation stress. **a** Five categories of alternative splicing. **b** Number of differential AS events in the five AS categories of SSI, WSI, SSL and WSL. **c** Distribution of -log10 (*p* value) values of differential AS events in each AS category. **d** Number of molecular functions enriched by protein-coding genes involving differential AS events. **e** Number of overlapped molecular functions on intersections of different AS categories. **f** Incidence of enriched similar molecular function from genes regulated by different AS categories. **g** Two tendentious modes. AS means alternative splicing. A3SS, A5SS, MXE, RI and SE refer respectively to alternative 3′ splice site, alternative 5′ splice site, mutually exclusive exons, retained intron and skipped exon. SSI, WSI, SSL, and WSL separately indicate ileum under salt stress, ileum under water-deprivation stress, liver under salt stress and liver under water-deprivation stress

In the ileum and liver of salt stress (SS) and water-deprivation stress (WS) groups, five alternatively splicing (AS) categories were statistically analysed, including skipped exon (SE), retained intron (RI), mutually exclusive exons (MXE), alternative 5′ splice site (A5SS) and alternative 3′ splice site (A3SS) (Fig. 1a). Focusing on event number of differential alternative splicing (*P* < 0.05), there were 239 (ileum under salt stress, SSI), 579 (ileum under water-deprivation stress, WSI), 174 (liver under salt stress, SSL) and 233 (liver under water-deprivation stress, WSL) in skipped exon, 7 (SSI), 17 (WSI), 7 (SSL) and 3 (WSL) in retained intron, 87 (SSI), 139 (WSI), 97 (SSL) and 156 (WSL) in mutually exclusive exons, 17 (SSI), 40 (WSI), 19 (SSL) and 18 (WSL) in alternative 5′ splice site, 33 (SSI), 70 (WSI), 26 (SSL) and 24 (WSL) in alternative 3′ splice site (Fig. 1b; Additional file 1: Tables S2 and S3). Collecting number of differential AS events, the distribution tendency was consistent with forth-order function (R^2^ = 1) and skipped exon mostly occurred in four conditions: SSI, WSI, SSL, and WSL (Fig. 1b, gray lines), with more significant difference of AS events manipulated by skipped exon and mutually exclusive exons (Fig. 1c).

Using Gene Ontology analysis, the molecular functions of genes involving differential AS were enriched, and their numbers were exhibited on Venn diagrams (Fig. 1d). We recorded the number of overlapped molecular functions in 26 laminated regions (Fig. 1d). It was observed that 1 and 8 intersections were severally presented in SSI and WSI, while 4 and 0 intersections were shown respectively in SSL and WSL (Fig. 1d and e). Hence the probabilities of overlapped molecular functions were 3.85% (SSI), 30.77% (WSI), 15.38% (SSL) and 0.00% (WSL) between five AS categories (Fig. 1f). These findings suggest that genes affected by different AS categories in ileum exhibit preferential enrichment compared to similar molecular functions as mode I under water-deprivation stress, and various molecular functions as mode II under salt stress; this pattern is opposite to that in liver of stress groups (Fig. 1g).

### Changed expression of protein-coding genes in ileum and liver

**Fig. 2:**
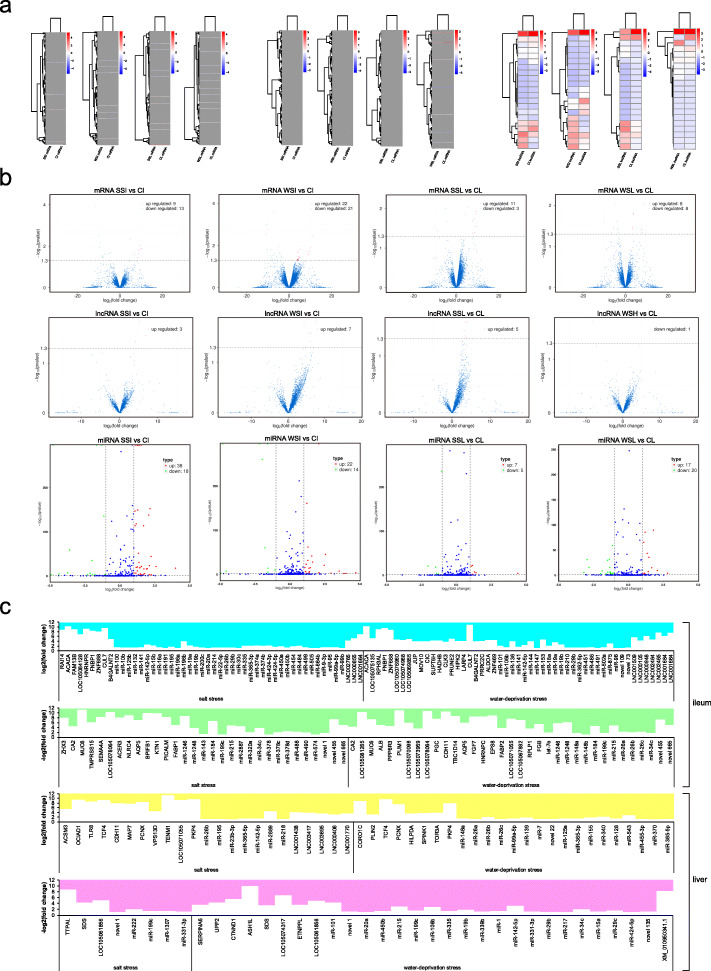
Differential mRNAs, miRNAs and lncRNAs in ileum and liver under salt stress and water-deprivation stress. **a** Expressed level of mRNA and lncRNA with value of log10(FPKM+ 1), and miRNA with value of log10(TPM + 1). **b** Number of differential mRNAs, miRNAs and lncRNAs. **c** Log2(fold change) values of up-regulated mRNAs, miRNAs and lncRNAs and -log2(fold change) values of down-regulated mRNAs, miRNAs and lncRNAs. SSI, WSI, CI, SSL, WSL and CL separately indicate ileum under salt stress, ileum under water-deprivation stress, control ileum, liver under salt stress, liver under water-deprivation stress and control liver

**Fig. 3:**
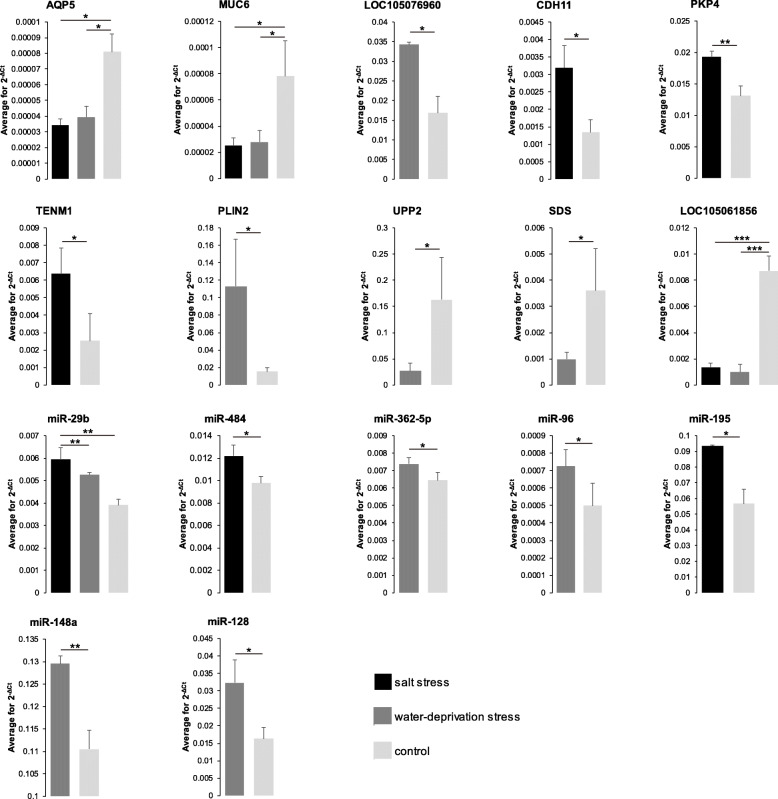
qRT-PCR detection of candidate water-deprivation resistant genes. Data are presented as mean ± SD. Symbols: *, 0.01 < *P* < 0.05; **, 0.001 < *P* < 0.01; ***, *P* < 0.001

**Fig. 4:**
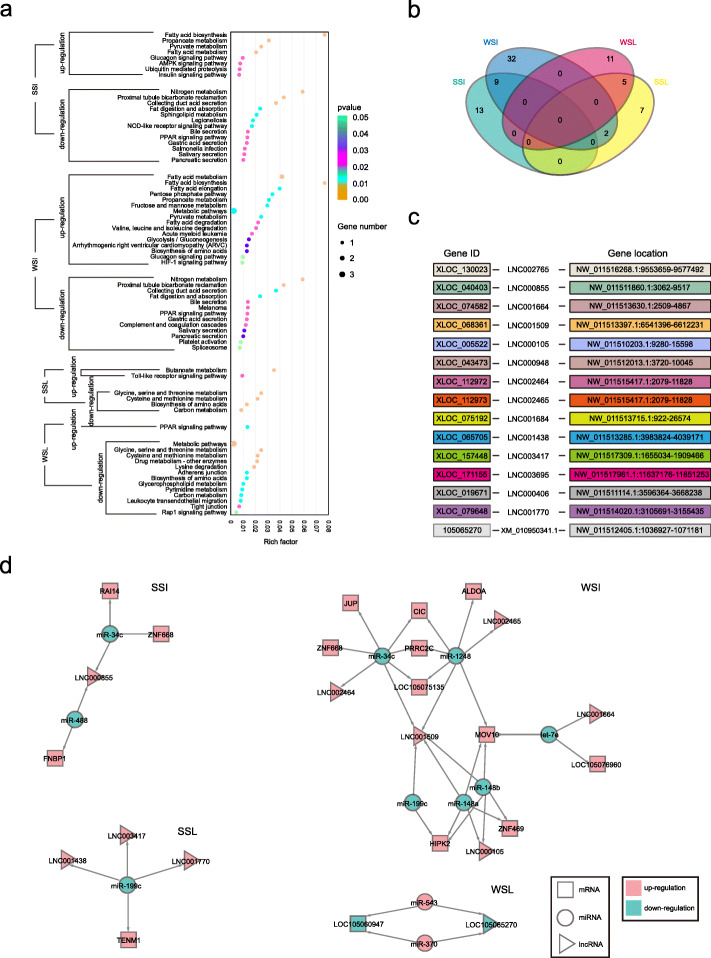
Bioinformatics analysis for significantly differential (*P* < 0.05) mRNAs, miRNAs and lncRNAs under stress. **a** KEGG pathway enrichment based on differential protein-coding genes. **b** Number of common protein-coding genes between stress groups. **c** Detailed genes information of differential lncRNAs. **d** Potential mRNAs, miRNAs and lncRNAs serving ceRNA theory. SSI, WSI, SSL and WSL separately indicate ileum under salt stress, ileum under water-deprivation stress, liver under salt stress and liver under water-deprivation stress

Within salt stress, the protein-coding genes with differential expression (*P* < 0.05) consisted of 22 genes (e.g. up-regulated *RAI14* and down-regulated *MUC6*) in ileum and 14 genes (e.g. up-regulated *CDH11* and down-regulated *LOC105061856*) in liver (Fig. 2a-c; Additional file 1: Tables S4 and S5). There were differentially expressed (*P* < 0.05) 43 genes (e.g. up-regulated *LOC105076960* and down-regulated *AQP5*) in ileum and 16 genes (e.g. up-regulated *PLIN2* and down-regulated *SDS*) in liver under water-deprivation stress (Fig. 2a-c; Additional file 1: Tables S4 and S5). Due to the correlation with sodium ion transport [41], fluid secretion [42] and energy metabolism [43], *MUC6*, *AQP5* and *LOC105076960* were screened as resistance genes in ileum. The *CDH11*, *PKP4*, *TENM1*, *SDS*, *LOC105061856*, *PLIN2* and *UPP2* that severally involved in cell–cell adhesion (GO:0098609), cadherin binding (GO:0045296), cell adhesion molecule binding (GO:0050839), gluconeogenesis (GO:0006094), oxygen transport (GO:0015671), long-chain fatty acid transport (GO:0015909) and uridine phosphorylase activity (GO:0004850) were selected as resistance-related genes in liver by GO enrichment analysis. Based on the results of qRT-PCR experiment, the expression level of *MUC6*, *AQP5*, *LOC105076960*, *PKP4*, *CDH11*, *TENM1*, *SDS*, *LOC105061856*, *PLIN2* and *UPP2* was consistent with RNA-seq data (Fig. 3). Except for salivary secretion with *AQP5*, no other pathways associated with salt and water metabolism were detected by KEGG pathway enrichment analysis (Fig. 4a). Notably, expression of *MUC6* and *AQP5* in ileum and *LOC105061856* in liver were down-regulated under both salt stress and water-deprivation stress conditions (Fig. 2 c and Fig. 4 b).

### Identification of miRNAs responding to stress

The miRNA data were obtained in salt and water-deprivation conditions by RNA-seq (Fig. 2a and b; Additional file 1: Tables S4 and S5). The differentially expressed (*P* < 0.05) 56 miRNAs (e.g. up-regulated miR-196b and down-regulated miR-574) under salt stress and 36 miRNAs (e.g. up-regulated miR-362-5p and down-regulated let-7e) under water-deprivation stress were detected in ileum (Fig. 2c). Besides, following the expressive difference (*P* < 0.05), 12 miRNAs (e.g. up-regulated miR-195 and down-regulated miR-199c) under salt stress and 37 miRNAs (e.g. up-regulated miR-543 and down-regulated miR-20a) under water-deprivation stress were discovered in liver (Fig. 2c). With miRNA target prediction, we were aware that *MUC6* mRNA was directed by miR-29b (SSI and WSI) and *AQP5* mRNA was aimed by miR-484 (SSI), miR-362-5p (WSI) and miR-96 (WSI). *LOC105061856* mRNA was uncovered as objective of miR-195 (SSL), miR-128 (WSL) and miR-148a (WSL), while *UPP2* mRNA was an alternative target of miR-128 (WSL). qRT-PCR results showed that the expression trend of miR-29b, miR-484, miR-362-5p, miR-96, miR-195, miR-128 and miR-148a was matched to RNA-seq data (Fig. 3).

### LncRNAs and evaluation of RNA-RNA interaction

For ileum and liver of camel under stresses, the differential lncRNAs (*P* < 0.05) were emerged by RNA-seq (Fig. 2a and b; Additional file 1: Tables S4-S6). The data of RNA-seq indicated that three up-regulated novel lncRNAs (LNC002765, LNC000855 and LNC001664) were detected under salt stress along with seven up-regulated novel lncRNAs (LNC001509, LNC000105, LNC000948, LNC002464, LNC002465, LNC001684 and LNC001664) under water-deprivation stress in ileum (Fig. 2 c and Fig. 4 c). There were five up-regulated new lncRNAs (LNC001438, LNC003417, LNC003695, LNC000406 and LNC001770) under salt stress and one down-regulated new lncRNA (XM_010950341.1) under water-deprivation stress in liver (Fig. 2 c and Fig. 4 c). Interestingly, we determined that the newfound lncRNA XM_010950341.1 was annotated as one of *LOC105065270* transcripts and related to response to water-deprivation stress in camel liver. Apart from coding protein CutA homolog, a type of RNA with unknown function is also transcribed from *LOC105065270* in the light of the annotated *Camelus bactrianus* genome. The transcription of *LOC105065270* into RNA sequences may be divided into two directions, comprising messenger RNA of coding protein and long non-coding RNA of performing post-transcriptional regulation. Using ceRNAs (mRNA-miRNA-lncRNA) prediction (Fig. 4d), we found that only LNC001664, let-7e and *LOC105076960* mRNA accorded with ceRNA principle in ileum, which *LOC105076960* participates in energy buffer and thus may assist in preventing the disorder of ionic gradients by regulating sodium potassium pump [43, 44]. But *MUC6* and *AQP5* mRNAs that are closely related to salt and water metabolism were not predicted as ceRNA. In liver, LNC001438, LNC003417, LNC001770, miR-199c and *TENM1* mRNA were detected to be the important factors for serving ceRNA theory (Fig. 4d).

## Discussion

In order to investigate how camels respond to the salt and water-deprivation stresses, we focused on differential alternative splicing and gene expression in ileum and liver. Based on statistical analysis of differential AS events, we discovered that the skipped exon as a class of AS category is mainly invoked in ileum and liver of camel under salt and water-deprivation stresses. This functional response is supported by a variety of stress-related studies on biological resistances, such as those to salt, temperature, and air exposure stresses [45]. Intriguingly, two modes were revealed in this test: genes influenced by five AS categories were preferentially enriched to similar molecular functions (mode I) under water-deprivation stress and diverse molecular functions (mode II) under salt stress in ileum. By contrast, in liver, the parallel molecular functions enriched by the AS genes as a tendency (mode I) occurred in salt stress group, with varied molecular functions (mode II) in water-deprivation stress group. The results implied that the molecular function enrichment pattern of genes driven by AS was influenced by organ type in response to stress.

**Fig. 5:**
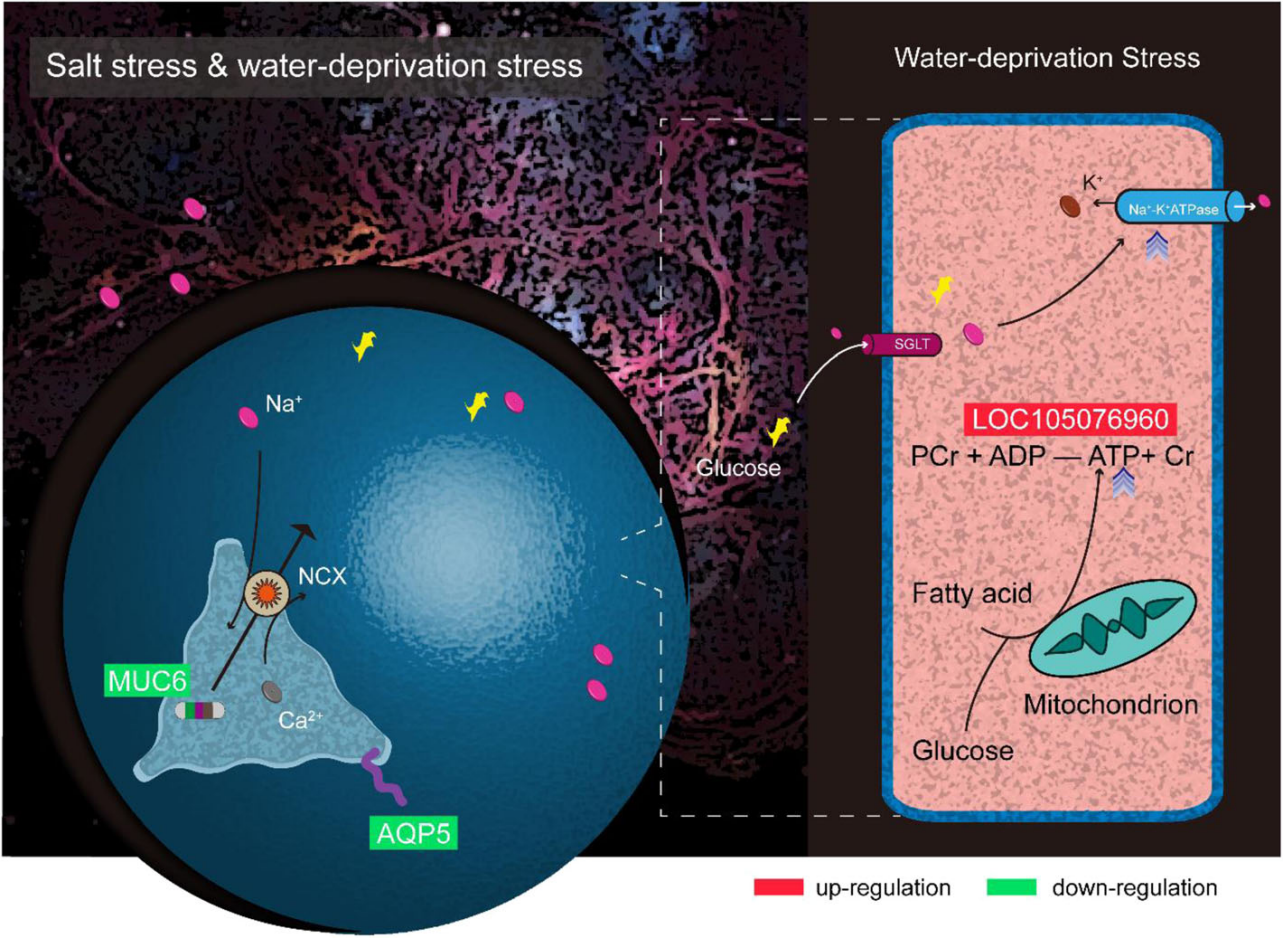
Weakened sodium ion import and water loss with active energy buffer responding to ambient challenge in camel ileum

Among the protein-coding genes with differential expression, *AQP5* encodes aquaporin-5 as a water channel protein located on biological cell membranes that promotes passage of water through the lipid bilayer at high flux [46, 47]. Scientists confirm that water channel protein enhances the permeability of membranes to water, and enables organism to adapt to salt and drought stresses [48]. AQP5 is supported to participate in water movement in acinar cells [49, 50]. These biological functions are also involved in sweating and salivary secretion, where expression and distribution of AQPs are modified under various xerostomic conditions. Furthermore, AQP5 translocation may improve the water permeability of the apical membranes of sweat glands [51, 52]. In mice, AQP5 deletion in submucosal glands reduced fluid secretion by more than 50 % [42], that is, AQP5 plays an important role in fluid secretion [53]. However, the effects of AQP5 have not been probed thoroughly in camel. There is evidence that AQP2 and AQP3 show stronger expression than in other species, but AQP4 deficiency in camel kidney [5, 54]. *MUC6* encoding mucin-6 is a member of the mucin family [55]. Reports reveal that mucin secretion is Na^+^/Ca^2+^ exchanger-dependent such as MUC5AC in goblet cells of the respiratory [56, 57], and substantially increases level of intracellular Na^+^ (up to 600-fold) with Ca^2+^ export [41]. In test outcomes, the gene expression of *AQP5* and *MUC6* was down-regulated in ileum under salt and water-deprivation stresses. Down-regulated *AQP5* appears to maintain osmotic homeostasis and prevent dehydration of cells, and the down-regulated *MUC6* probably encourages decreasing mucin secretion and avoiding excessive Na^+^ import by Na^+^/Ca^2+^ exchanger (Fig. 5). In term of camels, weakened import of Na^+^ into cells is a positive response in high-salt and waterless conditions [58]. In miRNA-mediated interaction in which miRNA suppresses the expression of protein-coding gene, the *AQP5* mRNA is predicted as potential target of up-regulated miR-484, miR-362-5p and miR-96, while *MUC6* mRNA is targeted by up-regulated miR-29b. Another interesting finding was that, under water-deprivation stress, the *LOC105076960* mRNA may act as the member of potential ceRNAs containing up-regulated LNC001664, down-regulated let-7e and up-regulated *LOC105076960* mRNA in the post-transcriptional regulation level. *LOC105076960* encodes mitochondrial creatine kinase (U-type) catalyzing the reversible reaction of “phospho-creatine + ADP ⇌ ATP + creatine” (also called as CK/PCr system) [43, 59]. Creatine phosphate (phospho-creatine) serves as a high energy reserve and rapid energy buffer, and works on quick regeneration of ATP in situ, fluctuation of spatial and temporal ATP levels, and energy transfer inside cells [60]. In water-deprivation condition (Fig. 5), the energy reserve may contribute to reducing the amount of sodium ion being pumped outside cells, thus preventing excess glucose into the ileum cells due to sodium ion gradients mediated by sodium-glucose cotransporter (SGLT) [61]. Osmosis unbalance and cell damage induced by massively accumulated glucose are further hindered [62]. Therefore in the present study, miRNA-mediated silencing of *AQP5* and *MUC6* was identified as core positively responding to salt and water-deprivation stresses with ceRNA-related activating of *LOC105076960*.

**Fig. 6:**
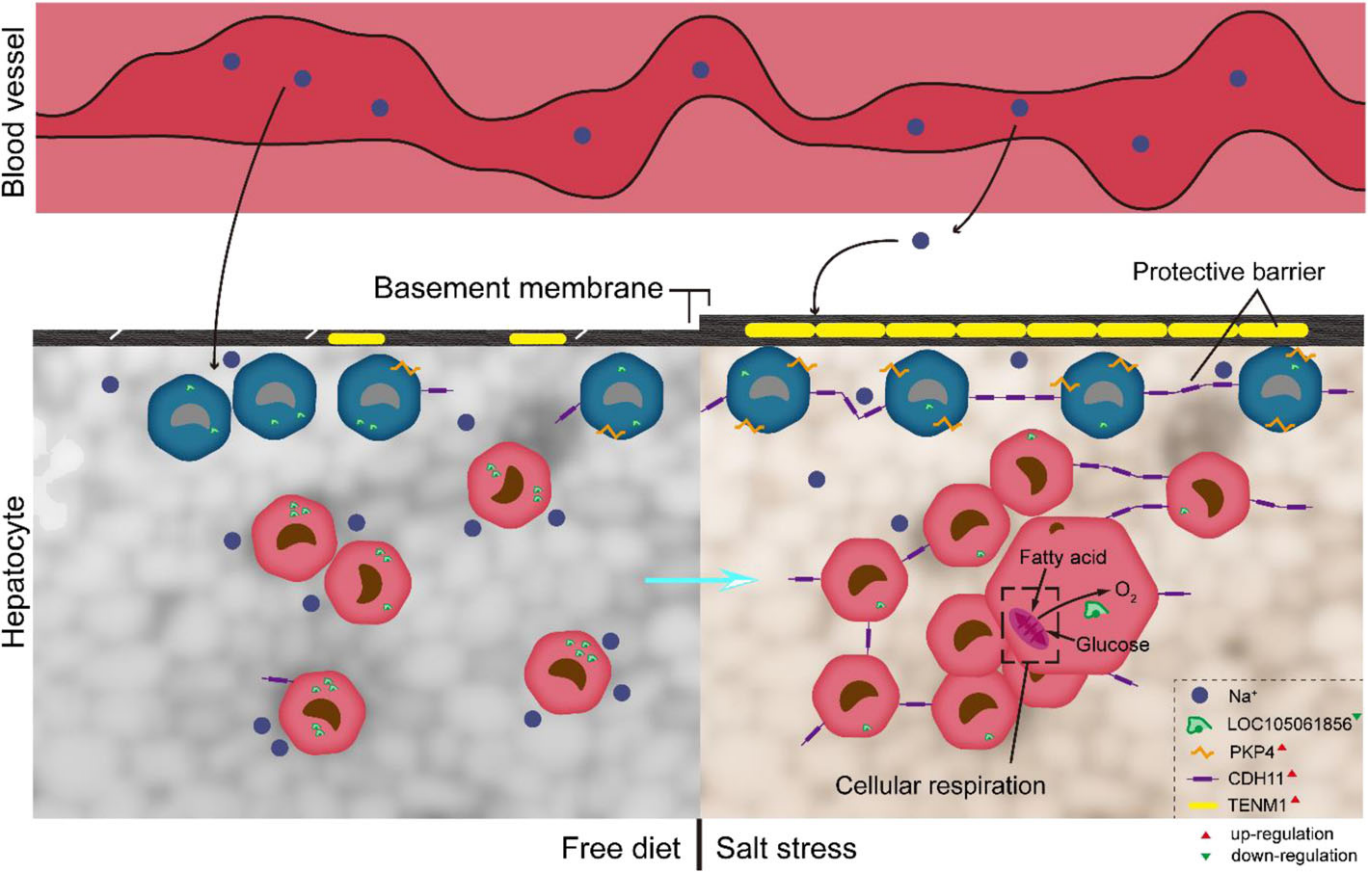
Advanced protective barrier and sodium ion homeostasis, and reduced aerobic respiration contributing on salt resistance in camel liver

In differentially expressed genes of liver under salt stress, *TENM1* is reported to play an indispensable role in maintaining integrity of basement membrane, in which the dearth of TENM1 causes local basement membrane deficiency of *Caenorhabditis elegans* teneurin ortholog [63]. Basement membranes are multifunctional such as constructing filtration barrier, mediating organogenesis and activating tissue repair [64, 65], and follow with interest high sodium loading. For instance, sodium hyper-reabsorption pushes glomerular filtration to higher rates [66]; moreover, glomerular hyperfiltration leads to an increase in glomerular basement membrane length [67]. Relying on the basement membrane of its gills, tilapia sustains sodium homeostasis and adapts to seawater by active Na^+^/K^+^-ATPase [68]. In rat colon, sodium invasion switches on remodeling of the basement membrane [69]. The cell-adhesion molecule cadherin-11 is encoded by *CDH11* [70–72]. Cadherins compose a family of cell surface proteins that, using the adherens junction as a basis, participate in cell–cell adhesion, an essential process in tissue morphogenesis and integrity [73]. A previous study reported that adherens junctions form defensive barriers and maintain ion homeostasis, and that their functional deficiency triggers ion imbalance and tissue inflammation [74]. In intestinal epithelium, the selective barrier formed by adherens junction supervises absorption of fluid and solutes [75]. The past studies have proved that the overexpressed plakophilin-4 encoded by *PKP4* is positioned along cell borders and strengthens cell-cell adhesion via recruiting cadherins to the membrane [76]. *LOC105061856* encodes hemoglobin subunit beta which is a component of hemoglobin that partakes in oxygen transport and mitochondrial oxidative phosphorylation [77–79]. There is a reasonable interpretation about how camels succeed in salt resistance by liver (Fig. 6). Specifically, with up-regulated LNC001438, LNC003417 and LNC001770 and down-regulated miR-199c, the up-regulated *TENM1* involving in ceRNA is conducive to liver survival by protective barrier role of basement membrane, while the up-regulated *CDH11* and *PKP4* would promote cell-cell adhesion and further attain sodium ion homeostasis by barrier. The down-regulated *LOC105061856*, mRNA of which was targeted by up-regulated miR-195, is able to slow down aerobic respiration and metabolism through reducing oxygen transported to mitochondria. Several lines of evidence from animal species have demonstrated that responses to high temperatures and arid conditions depend on dormancy, characteristics of which are inactivity and a lowered metabolic rate [80, 81].

**Fig. 7:**
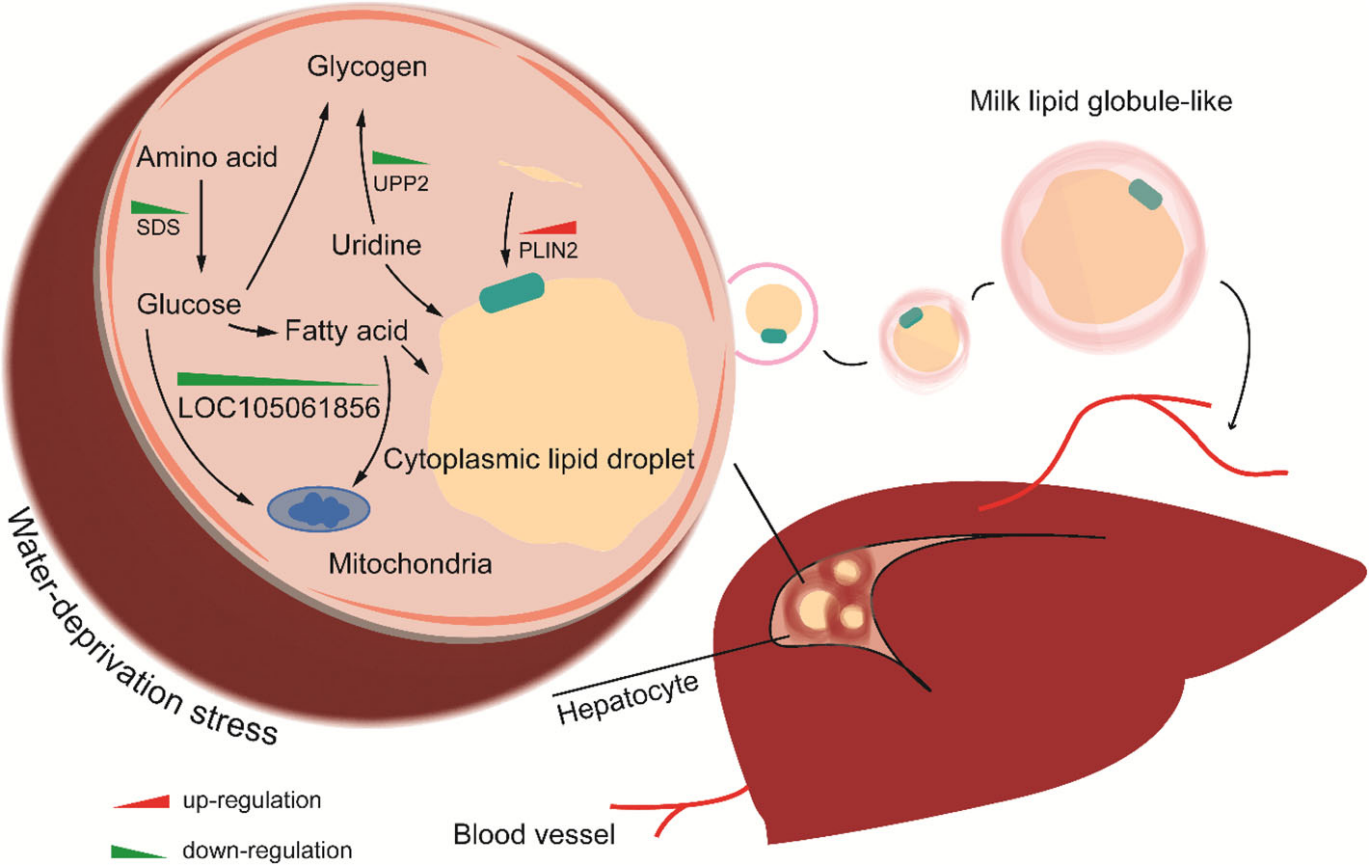
Improved uridine, glucose and lipid metabolism under water-deprivation stress in camel liver

Concerning the hepatic genes resisting water-deprivation stress, the role of *SDS* is presented as stimulatory gluconeogenesis in hepatocytes [82]. *SDS* can accelerate the reaction of serine to pyruvate, further increase generation of oxaloacetic acid and glucose in the process of gluconeogenesis [83]. Uridine, a pyrimidine nucleoside, shows restorative effects in positive response to stress in tissues, while mice experiment points out that long-period feeding of uridine arouses glucose intolerance and lipid accumulation in liver [84]. The uridine phosphorylase 2 encoded by *UPP2* promotes reaction from uridine to uridine diphosphate glucose and uridine dipho-sphate N-acetylglucosamine which are taken as substrates for glycogen synthesis and the protein of O-linked glycosylation. Gene *PLIN2* encodes perilipin-2, also known as adipose differentiation-related protein or adipophilin [85]. In nonadipose tissue, typically in liver, PLIN2 is the most common protein relevant to lipid droplet and its expression usually evokes intracellular lipid accumulation [86]. In addition, PLIN2 is also one of the most abundant proteins in the membrane of milk lipid globule which secretes massive lipid into milk by a unique process of membrane envelopment of cytoplasmic lipid droplets [87, 88]. The overexpression of PLIN2 increases lipid accumulation and triglyceride concentration in goat mammary epithelial cell [89]. In liver, as for camel responding positively to water-deprivation stress (Fig. 7), the down-regulated *UPP2* targeted by up-regulated miR-128 was implied to ensure uridine performing restorative function. In regard to glucose intolerance and lipid deposition induced by uridine accumulation, the down-regulated *SDS* would help to reduce gluconeogenesis and the up-regulated *PLIN2* could boost lipid accumulation and lipid droplet formation, then transfer lipid droplet to blood and deliver it to hump to store fat by membrane envelopment of milk lipid globule-like secretion as the responsible regulation pathway. Similarly, the down-regulated expression of *LOC105061856* targeted by up-regulated miR-128 and miR-148a is also considered as a facilitator to decrease the rate of cell aerobic metabolism.

In addition, we note that the present study identified down-regulation of LOC105061856 mRNA in camel liver under salt and water-deprivation stresses, consistent with the absence of its expression in camel renal medulla with water-deprivation condition [90]. This may signify that different organs utilize an overlapped mediation on gene expression to respond to ambient challenges in camels.

## Conclusions

Association analysis was applied on the mRNA, miRNA, and lncRNA data derived by RNA-seq. We unveiled two modes in the enrichment process from AS genes to their molecular functions, responding to stress in ileum and liver. Combined with the results of qRT-PCR, we screened out resistant coding/non-coding genes. In the post-transcriptional regulation level, the targeting relations were identified between resistant mRNAs, miRNAs and lncRNAs trying to explain how camels cope with salt and water-deprivation stresses in ileum and liver. We are hoping to make up for the deficiency in resistance-related research of camel and provide new theoretical inspirations for human diseases induced by high-salt diet. Nevertheless, there still remains a challenging task in amplifying full length of lncRNA genes coupled to water-deprivation condition, so we will seek appropriate approaches to solve it in the future.

## Supplementary information

**Additional file 1 Table S1.** Primer sequences of resistance-related candidate genes. **Table S2.** Differential alternative splicing events in ileum under salt stress and water-deprivation stress. **Table S3.** Differential alternative splicing events in liver under salt stress and water-deprivation stress. **Table S4.** Differential mRNAs, miRNAs and lncRNAs in ileum under salt stress and water-deprivation stress. **Table S5.** Differential mRNAs, miRNAs and lncRNAs in liver under salt stress and water-deprivation stress. **Table S6.** Sequence of differential novel lncRNAs in ileum and liver of camel under salt stress and water-deprivation stress.

## Data Availability

The datasets presented during the current study are available from the corresponding author on reasonable request.

## Abbreviations

A3SS: Alternative 3′ splice site
A5SS: Alternative 5′ splice site
AS: Alternative splicing
ceRNAs: Competing endogenous RNAs
GO: Gene Ontology
KEGG: Kyoto Encyclopedia of Genes and Genomes
lncRNA: Long non-coding RNA
miRNA: Micro RNA
MXE: Mutually exclusive exons
ncRNAs: Non-coding RNAs
RI: Retained intron
SE: Skipped exon
SGLT: Sodium-glucose cotransporter
SS: Salt stress
SSI: Ileum under salt stress
SSL: Liver under salt stress
WS: Water-deprivation stress
WSI: Ileum under water-deprivation stress
WSL: Liver under water-deprivation stress
