# Supplementary material for: Evidence from ileum and liver transcriptomes of resistance to high-salt and water-deprivation conditions in camel
Source: Zoological Lett. 2020 Jun 5;6:8. doi: 10.1186/s40851-020-00159-3 (PMC7275387; doi:10.1186/s40851-020-00159-3)
Supplement: Supplementary file 1 — Additional file 1 Table S1. Primer sequences of resistance-related candidate genes. Table S2. Differential alternative splicing events in ileum under salt stress and water-deprivation stress. Table S3. Differential alternative splicing events in liver under salt stress and water-deprivation stress. Table S4. Differential mRNAs, miRNAs and lncRNAs in ileum under salt stress and water-deprivation stress. Table S5. Differential mRNAs, miRNAs and lncRNAs in liver under salt stress and water-deprivation stress. Table S6. Sequence of differential novel lncRNAs in ileum and liver of camel under salt stress and water-deprivation stress. [file 40851_2020_159_MOESM1_ESM.docx]

Table S1. The primer sequence of resistance-related candidate genes

| **Gene** | **Primer name** | **Primer sequence (5' to 3')** |
| --- | --- | --- |
| *AQP5* | 170303-AQP5-F | GACCCTCCCCAGAGCTTTTTA |
|  | 170303-AQP5-R | CCCTACCCCATGCATCCTT |
| *MUC6* | 170303-MUC6-F | GCAACTACAACGGGAACATGAA |
|  | 170303-MUC6-R | GCTGGATGCCACATACTTGCT |
| *LOC105076960* | 180023-LOC105076960-F | GACCGACTGGGCAAGTCAGA |
|  | 180023-LOC105076960-R | GCCGTTCACAGTCAATCAAATAGT |
| *CDH11* | 180023-CDH11-F | CCTCGCCTGCATCGTCAT |
|  | 180023-CDH11-R | TTGCCTTCGTAGGGTCACAAA |
| *PKP4* | 180023-PKP4-F | CCTCACCAGCAAGAGAACAAAA |
|  | 180023-PKP4-R | GTTCTTTCTGTTGGAGTCATCTTGAC |
| *TENM1* | 170303-TENM1-F | CCTGAGCCATGTTGCTAGGAA |
|  | 170303-TENM1-R | GCACAGGAGTGAGAGCAGGAA |
| *PLIN2* | 170303-PLIN2-F | CACAGAACATCCAAGACCAGGC |
|  | 170303-PLIN2-R | GCGAGGAGGCTTTCAGACACT |
| *UPP2* | 180023-UPP2-F | GCGTCAGGAATATCGAAATGG |
|  | 180023-UPP2-R | CACAGCAGCTTTTAGACCACAAA |
| *SDS* | 180023-SDS-F | CAGCCCATTCTCTCTGAAGTTGT |
|  | 180023-SDS-R | TCTCATCATCTGCAAACTTCTCAAT |
| *LOC105061856* | 170303-LOC105061856-F | GGGACCTGTCCACTGCTGAT |
|  | 170303-LOC105061856-R | CTTACTGCCATGGGCCTTCA |
| miR-29b | 170303-bta-miR-29b | TAGGACCATTTGAAATCAGTGTT |
| miR-484 | 170303-bta-miR-484 | TCTCAGTCCCCTCCCGAT |
| miR-362-5p | 170303-bta-miR-362-5p | ATCCTTGGAACCTATGTGTGAG |
| miR-96 | 170303-bta-miR-96 | TTTGGCACTAGCACATTTTTG |
| miR-195 | 170303-bta-miR-195 | TAGCAGCACAGAACTATTGGC |
| miR-148a | 170303-bta-miR-148a | TCAGTGCCCTACAGAACTTTGT |
| miR-128 | 170303-bta-miR-128 | TCACAGTGAACCCGTCTCTTT |
| β-actin | 170303-actin-F | CCACCCTTCTCTTGACAAAACC |
|  | 170303-actin-R | CCAAATAAAGCCATGCCAATC |
| U6 | U6 | TTCGTGAAGCGTTCCATATTTT |

Table S2. The differential alternative splicing events of ileum under salt stress and water-deprivation stress

| **AS category** | **Stress** | **GeneID** | **strand** | **exonStart_0base** | **exonEnd** | **upstreamES** | **upstreamEE** | **downstreamES** | **downstreamEE** | **IC_SAMPLE_1** | **SC_SAMPLE_1** | **IC_SAMPLE_2** | **SC_SAMPLE_2** | **IncFormLen** | **SkipFormLen** | **PValue** | **IncLevel1** | **IncLevel2** | **IncLevelDifference** |  |  |
| --- | --- | --- | --- | --- | --- | --- | --- | --- | --- | --- | --- | --- | --- | --- | --- | --- | --- | --- | --- | --- | --- |
| SE | SS | 105068831 | + | 4955125 | 4955203 | 4952297 | 4952465 | 4955332 | 4955460 | 410 | 1 | 138 | 46 | 128 | 121 | 1.22E-15 | 0.997 | 0.739 | 0.258 |  |  |
|  |  | 105064294 | + | 490327 | 490429 | 489055 | 489163 | 491360 | 491462 | 333 | 1 | 210 | 42 | 116 | 61 | 1.68E-11 | 0.994 | 0.724 | 0.27 |  |  |
|  |  | 105063946 | + | 4786796 | 4786915 | 4778035 | 4778099 | 4790623 | 4793490 | 26 | 20 | 39 | 1 | 146 | 57 | 1.74E-05 | 0.337 | 0.938 | -0.601 |  |  |
|  |  | 105079809 | - | 1191273 | 1191585 | 1190859 | 1190993 | 1201024 | 1201219 | 460 | 26 | 233 | 49 | 425 | 127 | 2.28E-05 | 0.841 | 0.587 | 0.254 |  |  |
|  |  | 105063054 | + | 5028632 | 5028845 | 5003543 | 5004041 | 5029620 | 5030999 | 40 | 3 | 13 | 16 | 334 | 135 | 3.46E-05 | 0.843 | 0.247 | 0.596 |  |  |
|  |  | 105074805 | + | 6169920 | 6170001 | 6169664 | 6169805 | 6170227 | 6170372 | 3 | 13 | 19 | 2 | 147 | 134 | 3.78E-05 | 0.174 | 0.896 | -0.722 |  |  |
|  |  | 105062951 | - | 6087240 | 6087477 | 6073546 | 6073814 | 6091621 | 6091909 | 299 | 20 | 391 | 89 | 358 | 135 | 4.97E-05 | 0.849 | 0.624 | 0.225 |  |  |
|  |  | 105073519 | + | 2045071 | 2045156 | 2039570 | 2039765 | 2068206 | 2068335 | 17 | 39 | 53 | 22 | 143 | 122 | 5.78E-05 | 0.271 | 0.673 | -0.402 |  |  |
|  |  | 105078648 | - | 2053152 | 2053409 | 2050795 | 2050922 | 2054739 | 2054921 | 58 | 15 | 126 | 2 | 363 | 120 | 6.48E-05 | 0.561 | 0.954 | -0.393 |  |  |
|  |  | 105067430 | + | 20889130 | 20889286 | 20881995 | 20882175 | 20891837 | 20892435 | 65 | 5 | 53 | 31 | 277 | 135 | 7.26E-05 | 0.864 | 0.455 | 0.409 |  |  |
|  |  | 105074848 | - | 6897698 | 6897850 | 6897395 | 6897572 | 6898500 | 6898559 | 1702 | 149 | 1022 | 179 | 190 | 52 | 9.58E-05 | 0.758 | 0.61 | 0.148 |  |  |
|  |  | 105068634 | - | 828942 | 829218 | 825629 | 827166 | 831519 | 831706 | 49 | 25 | 33 | 75 | 397 | 135 | 0.000147705 | 0.4 | 0.13 | 0.27 |  |  |
|  |  | 105070045 | + | 2268763 | 2269027 | 2268180 | 2268309 | 2269383 | 2269524 | 142 | 31 | 102 | 69 | 371 | 121 | 0.000173547 | 0.599 | 0.325 | 0.274 |  |  |
|  |  | 105063387 | - | 1206296 | 1206436 | 1202494 | 1202667 | 1207374 | 1207574 | 10 | 0 | 2 | 10 | 266 | 135 | 0.000188096 | 1 | 0.092 | 0.908 |  |  |
|  |  | 105070835 | - | 1137581 | 1137659 | 1137140 | 1137322 | 1140434 | 1140572 | 19 | 0 | 18 | 16 | 138 | 131 | 0.000325958 | 1 | 0.516 | 0.484 |  |  |
|  |  | 105062496 | + | 1153376 | 1153646 | 1143974 | 1144038 | 1155875 | 1156039 | 131 | 0 | 110 | 12 | 313 | 57 | 0.000412402 | 1 | 0.625 | 0.375 |  |  |
|  |  | 105063408 | - | 347781 | 347844 | 343264 | 343441 | 352375 | 352617 | 79 | 92 | 40 | 130 | 112 | 135 | 0.000495642 | 0.509 | 0.271 | 0.238 |  |  |
|  |  | 105076526 | - | 11909431 | 11909604 | 11906009 | 11906906 | 11911319 | 11911501 | 40 | 0 | 34 | 12 | 294 | 135 | 0.000624307 | 1 | 0.565 | 0.435 |  |  |
|  |  | 105063149 | - | 18564155 | 18564296 | 18560873 | 18561104 | 18564493 | 18564647 | 110 | 13 | 157 | 0 | 268 | 135 | 0.000880612 | 0.81 | 1 | -0.19 |  |  |
|  |  | 105065666 | - | 5439510 | 5439775 | 5436960 | 5437104 | 5442520 | 5442595 | 83 | 8 | 176 | 0 | 319 | 68 | 0.000898954 | 0.689 | 1 | -0.311 |  |  |
|  |  | 105064040 | + | 7336510 | 7336644 | 7335281 | 7335471 | 7336815 | 7336936 | 46 | 0 | 59 | 15 | 233 | 114 | 0.001095704 | 1 | 0.658 | 0.342 |  |  |
|  |  | 105069345 | - | 1649586 | 1649718 | 1647328 | 1647445 | 1650121 | 1650263 | 55 | 17 | 82 | 3 | 225 | 110 | 0.001250765 | 0.613 | 0.93 | -0.317 |  |  |
|  |  | 105069009 | - | 24420 | 24609 | 23236 | 23386 | 25476 | 25675 | 10 | 0 | 0 | 4 | 310 | 135 | 0.001281294 | 1 | 0 | 1 |  |  |
|  |  | 105083626 | - | 1587679 | 1587829 | 1586293 | 1586443 | 1608884 | 1609024 | 12 | 1 | 2 | 8 | 269 | 133 | 0.001300253 | 0.856 | 0.11 | 0.746 |  |  |
|  |  | 105063387 | - | 1206296 | 1206436 | 1202207 | 1202395 | 1207374 | 1207574 | 11 | 0 | 2 | 6 | 266 | 135 | 0.001466742 | 1 | 0.145 | 0.855 |  |  |
|  |  | 105071932 | - | 12355973 | 12356218 | 12354868 | 12354928 | 12360032 | 12360163 | 40 | 6 | 41 | 35 | 273 | 42 | 0.001473572 | 0.506 | 0.153 | 0.353 |  |  |
|  |  | 105083159 | - | 4527043 | 4527108 | 4526474 | 4526698 | 4529914 | 4530057 | 16 | 2 | 9 | 16 | 116 | 135 | 0.001505169 | 0.903 | 0.396 | 0.507 |  |  |
|  |  | 105066739 | + | 25247 | 25529 | 21563 | 21914 | 25643 | 25844 | 102 | 15 | 148 | 2 | 403 | 135 | 0.001510341 | 0.695 | 0.961 | -0.266 |  |  |
|  |  | 105064040 | + | 7336040 | 7336260 | 7335281 | 7335471 | 7336815 | 7336936 | 133 | 0 | 160 | 15 | 320 | 114 | 0.001597581 | 1 | 0.792 | 0.208 |  |  |
|  |  | 105082155 | - | 26623860 | 26623926 | 26622239 | 26622450 | 26626617 | 26626765 | 28 | 52 | 17 | 125 | 118 | 135 | 0.001624445 | 0.381 | 0.135 | 0.246 |  |  |
|  |  | 105064019 | - | 6862017 | 6862201 | 6860138 | 6860452 | 6862435 | 6862457 | 28 | 17 | 20 | 0 | 185 | 15 | 0.001976568 | 0.118 | 1 | -0.882 |  |  |
|  |  | 105068205 | - | 2489888 | 2490007 | 2489129 | 2489744 | 2490236 | 2490283 | 146 | 7 | 118 | 26 | 129 | 40 | 0.001999149 | 0.866 | 0.585 | 0.281 |  |  |
|  |  | 105070450 | - | 1569362 | 1569562 | 1567936 | 1568289 | 1570499 | 1570642 | 88 | 0 | 39 | 7 | 321 | 135 | 0.002198898 | 1 | 0.701 | 0.299 |  |  |
|  |  | 105082741 | + | 1470957 | 1471020 | 1469108 | 1469165 | 1478358 | 1478469 | 45 | 1 | 19 | 9 | 25 | 19 | 0.002208122 | 0.972 | 0.616 | 0.356 |  |  |
|  |  | 105074560 | + | 226280 | 226679 | 224735 | 225239 | 227251 | 227803 | 32 | 0 | 9 | 5 | 520 | 135 | 0.002379337 | 1 | 0.318 | 0.682 |  |  |
|  |  | 105069222 | + | 9642985 | 9643042 | 9641635 | 9641735 | 9648667 | 9648765 | 10 | 7 | 0 | 11 | 14 | 49 | 0.002461782 | 0.833 | 0 | 0.833 |  |  |
|  |  | 105072948 | - | 14397522 | 14397553 | 14392487 | 14392567 | 14398235 | 14398391 | 25 | 3 | 2 | 7 | 24 | 73 | 0.002482492 | 0.962 | 0.465 | 0.497 |  |  |
|  |  | 105069792 | - | 393604 | 393727 | 393093 | 393281 | 395579 | 395678 | 896 | 28 | 405 | 45 | 189 | 92 | 0.002529642 | 0.94 | 0.814 | 0.126 |  |  |
|  |  | 105070810 | + | 1119179 | 1119383 | 1113961 | 1114114 | 1120628 | 1120693 | 85 | 23 | 170 | 12 | 248 | 58 | 0.002534895 | 0.464 | 0.768 | -0.304 |  |  |
|  |  | 105078982 | - | 4900717 | 4900896 | 4899118 | 4899240 | 4902502 | 4903341 | 66 | 3 | 39 | 14 | 280 | 115 | 0.002655464 | 0.9 | 0.534 | 0.366 |  |  |
|  |  | 105066284 | - | 19001335 | 19001621 | 18999910 | 18999972 | 19005302 | 19005396 | 500 | 0 | 598 | 11 | 279 | 7 | 0.002675499 | 1 | 0.577 | 0.423 |  |  |
|  |  | 105066231 | - | 13385759 | 13385830 | 13382294 | 13382376 | 13392629 | 13392729 | 0 | 3 | 28 | 1 | 26 | 33 | 0.002726566 | 0 | 0.973 | -0.973 |  |  |
|  |  | 105074331 | - | 841987 | 842109 | 839598 | 839746 | 842246 | 842479 | 209 | 22 | 117 | 0 | 230 | 135 | 0.002736087 | 0.848 | 1 | -0.152 |  |  |
|  |  | 105062889 | + | 1695077 | 1695178 | 1694274 | 1694404 | 1695855 | 1695887 | 62 | 45 | 93 | 21 | 82 | 13 | 0.00288656 | 0.179 | 0.412 | -0.233 |  |  |
|  |  | 105074339 | - | 967883 | 968025 | 967542 | 967731 | 968260 | 968513 | 229 | 21 | 205 | 1 | 270 | 135 | 0.002892444 | 0.845 | 0.99 | -0.145 |  |  |
|  |  | 105084142 | - | 1779098 | 1779446 | 1777290 | 1777572 | 1783779 | 1783913 | 325 | 0 | 253 | 13 | 461 | 127 | 0.002897595 | 1 | 0.843 | 0.157 |  |  |
|  |  | 105075368 | + | 9328687 | 9328828 | 9328249 | 9328368 | 9332017 | 9332204 | 38 | 3 | 15 | 11 | 245 | 112 | 0.002943131 | 0.853 | 0.384 | 0.469 |  |  |
|  |  | 105071932 | - | 12355973 | 12356263 | 12354868 | 12354928 | 12360032 | 12360163 | 55 | 6 | 68 | 35 | 318 | 42 | 0.003035488 | 0.548 | 0.204 | 0.344 |  |  |
|  |  | 105073643 | - | 5776982 | 5777113 | 5775083 | 5776007 | 5781842 | 5782009 | 7 | 8 | 32 | 3 | 248 | 135 | 0.003248748 | 0.323 | 0.853 | -0.53 |  |  |
|  |  | 105068283 | + | 1826834 | 1826917 | 1825405 | 1825644 | 1828935 | 1829103 | 3 | 19 | 14 | 9 | 152 | 135 | 0.003682748 | 0.123 | 0.58 | -0.457 |  |  |
|  |  | 105073202 | - | 6094216 | 6094331 | 6088769 | 6088926 | 6094554 | 6094825 | 181 | 19 | 198 | 1 | 216 | 135 | 0.003727436 | 0.856 | 0.992 | -0.136 |  |  |
|  |  | 105084142 | - | 1779682 | 1779767 | 1777290 | 1777572 | 1783779 | 1783913 | 55 | 0 | 61 | 13 | 148 | 127 | 0.003751158 | 1 | 0.801 | 0.199 |  |  |
|  |  | 105079442 | + | 11039511 | 11039571 | 11037737 | 11038127 | 11042751 | 11043153 | 22 | 10 | 32 | 62 | 106 | 135 | 0.003791531 | 0.737 | 0.397 | 0.34 |  |  |
|  |  | 105078641 | - | 780400 | 780533 | 778767 | 778888 | 784684 | 784893 | 28 | 9 | 33 | 0 | 231 | 114 | 0.003800699 | 0.606 | 1 | -0.394 |  |  |
|  |  | 105082307 | - | 3170877 | 3171066 | 3169721 | 3170173 | 3171276 | 3171444 | 84 | 13 | 64 | 34 | 310 | 135 | 0.003931509 | 0.738 | 0.45 | 0.288 |  |  |
|  |  | 105074584 | - | 592652 | 592673 | 583388 | 583517 | 593308 | 593482 | 5 | 11 | 0 | 31 | 15 | 122 | 0.004238356 | 0.787 | 0 | 0.787 |  |  |
|  |  | 105083395 | - | 11388324 | 11388579 | 11385340 | 11385492 | 11390642 | 11390869 | 26 | 5 | 23 | 25 | 376 | 135 | 0.004861797 | 0.651 | 0.248 | 0.403 |  |  |
|  |  | 105063335 | + | 9701127 | 9701196 | 9693353 | 9693439 | 9703903 | 9703967 | 9 | 0 | 6 | 10 | 6 | 1 | 0.005034649 | 1 | 0.091 | 0.909 |  |  |
|  |  | 105078648 | - | 2054739 | 2054921 | 2050795 | 2050922 | 2060811 | 2060951 | 44 | 0 | 54 | 11 | 286 | 118 | 0.00508876 | 1 | 0.669 | 0.331 |  |  |
|  |  | 105083781 | - | 2715472 | 2715775 | 2711719 | 2712104 | 2716826 | 2716977 | 12 | 24 | 23 | 8 | 424 | 135 | 0.005145022 | 0.137 | 0.478 | -0.341 |  |  |
|  |  | 105062654 | - | 4726661 | 4726728 | 4725685 | 4725770 | 4733353 | 4733548 | 6 | 19 | 16 | 7 | 63 | 78 | 0.005345845 | 0.281 | 0.739 | -0.458 |  |  |
|  |  | 105070223 | - | 3035946 | 3036113 | 3030469 | 3030591 | 3039853 | 3039936 | 39 | 4 | 32 | 19 | 209 | 56 | 0.005409321 | 0.723 | 0.311 | 0.412 |  |  |
|  |  | 105075658 | - | 459499 | 459605 | 452265 | 453997 | 461305 | 461426 | 33 | 28 | 14 | 45 | 177 | 114 | 0.005778966 | 0.432 | 0.167 | 0.265 |  |  |
|  |  | 105083961 | + | 9112395 | 9113249 | 9110012 | 9110207 | 9141638 | 9142010 | 582 | 14 | 602 | 43 | 975 | 135 | 0.005895951 | 0.852 | 0.66 | 0.192 |  |  |
|  |  | 105080639 | + | 6520402 | 6520567 | 6520102 | 6520244 | 6520657 | 6520846 | 9 | 0 | 1 | 4 | 286 | 135 | 0.006064617 | 1 | 0.106 | 0.894 |  |  |
|  |  | 105062496 | + | 1150334 | 1150658 | 1143974 | 1144038 | 1155875 | 1156039 | 177 | 0 | 248 | 12 | 367 | 57 | 0.006305717 | 1 | 0.762 | 0.238 |  |  |
|  |  | 105072350 | + | 2062656 | 2062810 | 2062284 | 2062364 | 2064192 | 2064846 | 47 | 7 | 18 | 14 | 213 | 73 | 0.00664747 | 0.697 | 0.306 | 0.391 |  |  |
|  |  | 105074044 | + | 244482 | 244584 | 244210 | 244325 | 244804 | 244953 | 72 | 12 | 59 | 0 | 163 | 108 | 0.00675466 | 0.799 | 1 | -0.201 |  |  |
|  |  | 105076183 | - | 3345591 | 3345706 | 3341780 | 3341944 | 3346205 | 3346384 | 78 | 24 | 82 | 66 | 216 | 135 | 0.006978784 | 0.67 | 0.437 | 0.233 |  |  |
|  |  | 105078444 | - | 2056306 | 2056405 | 2053529 | 2054251 | 2059520 | 2059761 | 15 | 0 | 14 | 9 | 184 | 135 | 0.007384384 | 1 | 0.533 | 0.467 |  |  |
|  |  | 105064839 | - | 2201189 | 2201244 | 2200799 | 2201012 | 2205972 | 2206112 | 36 | 10 | 63 | 0 | 94 | 133 | 0.007405102 | 0.836 | 1 | -0.164 |  |  |
|  |  | 105077797 | - | 4930273 | 4930641 | 4928792 | 4928916 | 4932176 | 4932319 | 11 | 18 | 13 | 2 | 471 | 117 | 0.007483037 | 0.132 | 0.618 | -0.486 |  |  |
|  |  | 105072948 | - | 14397533 | 14397553 | 14392487 | 14392567 | 14398235 | 14398391 | 25 | 3 | 2 | 7 | 13 | 73 | 0.007859091 | 0.979 | 0.616 | 0.363 |  |  |
|  |  | 105075416 | - | 169323 | 169512 | 169161 | 169249 | 169833 | 170241 | 15 | 12 | 33 | 4 | 256 | 81 | 0.007978714 | 0.283 | 0.723 | -0.44 |  |  |
|  |  | 105064316 | - | 234084 | 234181 | 233233 | 233486 | 235283 | 235391 | 5 | 0 | 0 | 4 | 146 | 101 | 0.008201877 | 1 | 0 | 1 |  |  |
|  |  | 105066411 | - | 31863 | 32213 | 26062 | 26457 | 36223 | 36485 | 32 | 17 | 47 | 5 | 471 | 135 | 0.008364943 | 0.35 | 0.729 | -0.379 |  |  |
|  |  | 105073108 | + | 6139028 | 6139146 | 6135351 | 6135438 | 6139224 | 6139914 | 4 | 0 | 0 | 5 | 167 | 80 | 0.008649474 | 1 | 0 | 1 |  |  |
|  |  | 105075058 | - | 2063410 | 2063616 | 2061449 | 2061543 | 2068147 | 2068283 | 0 | 3 | 7 | 0 | 273 | 81 | 0.008698918 | 0 | 1 | -1 |  |  |
|  |  | 105083155 | + | 4401115 | 4401232 | 4399874 | 4399929 | 4411237 | 4411364 | 7 | 1 | 1 | 7 | 118 | 33 | 0.00876132 | 0.662 | 0.038 | 0.624 |  |  |
|  |  | 105073718 | - | 5934126 | 5934212 | 5931420 | 5931808 | 5934707 | 5934767 | 12 | 8 | 1 | 11 | 79 | 53 | 0.00922092 | 0.502 | 0.057 | 0.445 |  |  |
|  |  | 105082067 | - | 15708902 | 15709051 | 15692923 | 15693121 | 15722756 | 15722898 | 347 | 31 | 415 | 7 | 270 | 135 | 0.009674282 | 0.848 | 0.967 | -0.119 |  |  |
|  |  | 105079841 | + | 952 | 1056 | 3 | 90 | 2968 | 3072 | 13 | 28 | 32 | 17 | 101 | 42 | 0.009674812 | 0.162 | 0.439 | -0.277 |  |  |
|  |  | 105079851 | - | 1445286 | 1445349 | 1436042 | 1436148 | 1530127 | 1530192 | 6 | 8 | 0 | 15 | 20 | 22 | 0.009758309 | 0.452 | 0 | 0.452 |  |  |
|  |  | 105077947 | - | 9492950 | 9493086 | 9490007 | 9490210 | 9505204 | 9505286 | 49 | 6 | 96 | 0 | 198 | 75 | 0.009852196 | 0.756 | 1 | -0.244 |  |  |
|  |  | 105070935 | + | 6054228 | 6054315 | 6052195 | 6052345 | 6075347 | 6075554 | 31 | 0 | 26 | 8 | 160 | 135 | 0.009983183 | 1 | 0.733 | 0.267 |  |  |
|  |  | 105083330 | - | 3296193 | 3296390 | 3277563 | 3278729 | 3298430 | 3298644 | 9 | 5 | 20 | 0 | 318 | 135 | 0.01007397 | 0.433 | 1 | -0.567 |  |  |
|  |  | 105076297 | - | 7029463 | 7029535 | 7027769 | 7028803 | 7029778 | 7029885 | 25 | 10 | 9 | 18 | 95 | 100 | 0.010549054 | 0.725 | 0.345 | 0.38 |  |  |
|  |  | 105065606 | - | 3418310 | 3418427 | 3416244 | 3416439 | 3421525 | 3421669 | 15 | 0 | 4 | 4 | 220 | 135 | 0.010856875 | 1 | 0.38 | 0.62 |  |  |
|  |  | 105078561 | - | 1568688 | 1569229 | 1563670 | 1563891 | 1572929 | 1573010 | 183 | 7 | 245 | 0 | 601 | 74 | 0.011140593 | 0.763 | 1 | -0.237 |  |  |
|  |  | 105071508 | + | 1734973 | 1735063 | 1731390 | 1731535 | 1745718 | 1745901 | 24 | 7 | 34 | 0 | 166 | 135 | 0.011287305 | 0.736 | 1 | -0.264 |  |  |
|  |  | 105083028 | + | 8723443 | 8724210 | 8723230 | 8723345 | 8725555 | 8725675 | 554 | 18 | 667 | 4 | 839 | 86 | 0.011291152 | 0.759 | 0.945 | -0.186 |  |  |
|  |  | 105080967 | - | 1568242 | 1568281 | 1562141 | 1563134 | 1573600 | 1573779 | 5 | 11 | 0 | 23 | 64 | 135 | 0.011420115 | 0.489 | 0 | 0.489 |  |  |
|  |  | 105083627 | - | 1831608 | 1831731 | 1816687 | 1816778 | 1900569 | 1900717 | 11 | 6 | 0 | 6 | 181 | 84 | 0.011637366 | 0.46 | 0 | 0.46 |  |  |
|  |  | 105079610 | + | 1272177 | 1272330 | 1264177 | 1264310 | 1272778 | 1272939 | 56 | 0 | 79 | 11 | 265 | 126 | 0.011731899 | 1 | 0.773 | 0.227 |  |  |
|  |  | 105077288 | + | 31139296 | 31139455 | 31134546 | 31134627 | 31148920 | 31149455 | 93 | 14 | 123 | 3 | 219 | 74 | 0.012179673 | 0.692 | 0.933 | -0.241 |  |  |
|  |  | 105073919 | - | 3956504 | 3956564 | 3951482 | 3951614 | 3958198 | 3958273 | 30 | 12 | 26 | 37 | 43 | 58 | 0.012271607 | 0.771 | 0.487 | 0.284 |  |  |
|  |  | 105068997 | + | 10386433 | 10386517 | 10384929 | 10385260 | 10387754 | 10387939 | 7 | 0 | 3 | 6 | 154 | 135 | 0.012285365 | 1 | 0.305 | 0.695 |  |  |
|  |  | 105066637 | + | 9913325 | 9913454 | 9904540 | 9904688 | 9915389 | 9915549 | 14 | 6 | 38 | 1 | 244 | 135 | 0.012527906 | 0.564 | 0.955 | -0.391 |  |  |
|  |  | 105074924 | + | 3744161 | 3744323 | 3741189 | 3741246 | 3747971 | 3748073 | 81 | 0 | 98 | 8 | 158 | 10 | 0.013147924 | 1 | 0.437 | 0.563 |  |  |
|  |  | 105062096 | + | 179681 | 179797 | 178282 | 178351 | 183310 | 183826 | 30 | 13 | 95 | 10 | 145 | 62 | 0.013503661 | 0.497 | 0.802 | -0.305 |  |  |
|  |  | 105066054 | - | 2950149 | 2950302 | 2948288 | 2948478 | 2950468 | 2950701 | 23 | 16 | 26 | 3 | 274 | 135 | 0.013686299 | 0.415 | 0.81 | -0.395 |  |  |
|  |  | 105073937 | - | 6902135 | 6902372 | 6897106 | 6897237 | 6905337 | 6905490 | 28 | 6 | 39 | 0 | 347 | 124 | 0.013693422 | 0.625 | 1 | -0.375 |  |  |
|  |  | 105077678 | + | 2450067 | 2450277 | 2445551 | 2445737 | 2451121 | 2451324 | 109 | 0 | 149 | 12 | 331 | 135 | 0.013716074 | 1 | 0.835 | 0.165 |  |  |
|  |  | 105065468 | - | 365147 | 365186 | 363643 | 364665 | 365397 | 365535 | 1 | 19 | 8 | 11 | 60 | 131 | 0.014042255 | 0.103 | 0.614 | -0.511 |  |  |
|  |  | 105072384 | + | 9314478 | 9314600 | 9314251 | 9314398 | 9317183 | 9318051 | 4 | 0 | 0 | 4 | 230 | 135 | 0.014118013 | 1 | 0 | 1 |  |  |
|  |  | 105070582 | - | 635066 | 635097 | 634408 | 634540 | 637979 | 638126 | 17 | 0 | 2 | 4 | 38 | 125 | 0.014321158 | 1 | 0.622 | 0.378 |  |  |
|  |  | 105080247 | + | 2511338 | 2511436 | 2511067 | 2511203 | 2511529 | 2511676 | 28 | 1 | 29 | 12 | 176 | 129 | 0.014618005 | 0.954 | 0.639 | 0.315 |  |  |
|  |  | 105069345 | - | 1649586 | 1649715 | 1647328 | 1647445 | 1650121 | 1650263 | 61 | 17 | 60 | 3 | 219 | 110 | 0.014995507 | 0.643 | 0.909 | -0.266 |  |  |
|  |  | 105065177 | + | 163100 | 163208 | 155349 | 155669 | 167914 | 168024 | 26 | 6 | 40 | 0 | 170 | 103 | 0.015251564 | 0.724 | 1 | -0.276 |  |  |
|  |  | 105074461 | + | 5472985 | 5473082 | 5472792 | 5472907 | 5473588 | 5473749 | 19 | 5 | 42 | 0 | 153 | 108 | 0.015927501 | 0.728 | 1 | -0.272 |  |  |
|  |  | 105064321 | - | 453869 | 453959 | 452306 | 452405 | 456904 | 456987 | 36 | 0 | 19 | 5 | 64 | 33 | 0.015980191 | 1 | 0.662 | 0.338 |  |  |
|  |  | 105076452 | + | 1360464 | 1360597 | 1359919 | 1360009 | 1361391 | 1361474 | 361 | 35 | 352 | 12 | 141 | 24 | 0.016170967 | 0.637 | 0.833 | -0.196 |  |  |
|  |  | 105066010 | + | 1233201 | 1233314 | 1231715 | 1231926 | 1234060 | 1234178 | 80 | 29 | 76 | 8 | 188 | 111 | 0.016641365 | 0.62 | 0.849 | -0.229 |  |  |
|  |  | 105078142 | - | 859925 | 860124 | 859782 | 859854 | 860265 | 860429 | 79 | 3 | 20 | 6 | 250 | 65 | 0.016778171 | 0.873 | 0.464 | 0.409 |  |  |
|  |  | 105081464 | - | 4373007 | 4373052 | 4364419 | 4365908 | 4374637 | 4374723 | 18 | 46 | 9 | 84 | 38 | 79 | 0.016934355 | 0.449 | 0.182 | 0.267 |  |  |
|  |  | 105078648 | - | 2053152 | 2053409 | 2050795 | 2050922 | 2060811 | 2060951 | 62 | 0 | 104 | 11 | 361 | 118 | 0.016978321 | 1 | 0.756 | 0.244 |  |  |
|  |  | 105064425 | - | 11828904 | 11828922 | 11826882 | 11827016 | 11835422 | 11835467 | 5 | 5 | 0 | 11 | 3 | 30 | 0.0181326 | 0.909 | 0 | 0.909 |  |  |
|  |  | 105076617 | + | 26100296 | 26100454 | 26075577 | 26075662 | 26122287 | 26122484 | 41 | 2 | 36 | 12 | 222 | 78 | 0.018326842 | 0.878 | 0.513 | 0.365 |  |  |
|  |  | 105065892 | - | 4684983 | 4685051 | 4682527 | 4683927 | 4685921 | 4686093 | 26 | 6 | 41 | 37 | 122 | 135 | 0.018501602 | 0.827 | 0.551 | 0.276 |  |  |
|  |  | 105066662 | + | 12143124 | 12143282 | 12138402 | 12138617 | 12144196 | 12144331 | 10 | 1 | 4 | 7 | 272 | 128 | 0.018591845 | 0.825 | 0.212 | 0.613 |  |  |
|  |  | 105076794 | - | 22746610 | 22746779 | 22746193 | 22746339 | 22747559 | 22749358 | 2 | 6 | 5 | 0 | 290 | 135 | 0.019160425 | 0.134 | 1 | -0.866 |  |  |
|  |  | 105081811 | - | 8269625 | 8269788 | 8269328 | 8269478 | 8271641 | 8271861 | 156 | 12 | 49 | 14 | 284 | 135 | 0.019250633 | 0.861 | 0.625 | 0.236 |  |  |
|  |  | 105074095 | - | 894258 | 894399 | 893058 | 893460 | 894787 | 894842 | 18 | 27 | 15 | 4 | 181 | 48 | 0.019405655 | 0.15 | 0.499 | -0.349 |  |  |
|  |  | 105073983 | - | 14938274 | 14938401 | 14937319 | 14937696 | 14952412 | 14952842 | 52 | 0 | 35 | 6 | 240 | 135 | 0.019862982 | 1 | 0.766 | 0.234 |  |  |
|  |  | 105064881 | - | 3083024 | 3083160 | 3082597 | 3082933 | 3083717 | 3083912 | 13 | 7 | 5 | 17 | 258 | 135 | 0.020171748 | 0.493 | 0.133 | 0.36 |  |  |
|  |  | 105079157 | + | 8006912 | 8006949 | 8005941 | 8006010 | 8008092 | 8010657 | 10 | 3 | 1 | 6 | 30 | 62 | 0.020685653 | 0.873 | 0.256 | 0.617 |  |  |
|  |  | 105066714 | + | 8621558 | 8621652 | 8596994 | 8597198 | 8634736 | 8634901 | 8 | 8 | 8 | 0 | 174 | 135 | 0.020716582 | 0.437 | 1 | -0.563 |  |  |
|  |  | 105064537 | - | 2673915 | 2674042 | 2673449 | 2673507 | 2674920 | 2675342 | 95 | 7 | 126 | 0 | 156 | 51 | 0.021101237 | 0.816 | 1 | -0.184 |  |  |
|  |  | 105070726 | + | 2172566 | 2172839 | 2171681 | 2171954 | 2175612 | 2175885 | 46 | 4 | 28 | 12 | 394 | 135 | 0.022346237 | 0.798 | 0.444 | 0.354 |  |  |
|  |  | 105083079 | - | 930175 | 930202 | 929686 | 929829 | 930297 | 930422 | 9 | 5 | 1 | 9 | 23 | 118 | 0.022513513 | 0.902 | 0.363 | 0.539 |  |  |
|  |  | 105070731 | + | 2721319 | 2721436 | 2719652 | 2719748 | 2721698 | 2721891 | 125 | 13 | 122 | 1 | 174 | 89 | 0.02314031 | 0.831 | 0.984 | -0.153 |  |  |
|  |  | 105082307 | - | 3171316 | 3171444 | 3170877 | 3171066 | 3171613 | 3171995 | 27 | 0 | 28 | 7 | 242 | 135 | 0.023462435 | 1 | 0.691 | 0.309 |  |  |
|  |  | 105080557 | + | 3187064 | 3187208 | 3185875 | 3186094 | 3191701 | 3191832 | 3 | 11 | 7 | 2 | 259 | 124 | 0.023487372 | 0.115 | 0.626 | -0.511 |  |  |
|  |  | 105083279 | - | 578359 | 578401 | 576799 | 576868 | 578858 | 582177 | 47 | 4 | 24 | 13 | 35 | 62 | 0.023608103 | 0.954 | 0.766 | 0.188 |  |  |
|  |  | 105075089 | - | 1271790 | 1271858 | 1271487 | 1271553 | 1272097 | 1272196 | 14 | 6 | 18 | 0 | 18 | 16 | 0.02367481 | 0.675 | 1 | -0.325 |  |  |
|  |  | 105080182 | + | 731030 | 731178 | 730604 | 730728 | 731665 | 731850 | 57 | 6 | 101 | 0 | 252 | 117 | 0.023995656 | 0.815 | 1 | -0.185 |  |  |
|  |  | 105070031 | + | 1319007 | 1319046 | 1317351 | 1317462 | 1320832 | 1320969 | 8 | 2 | 1 | 6 | 28 | 99 | 0.024253817 | 0.934 | 0.371 | 0.563 |  |  |
|  |  | 105072129 | - | 779835 | 779946 | 771541 | 772630 | 780719 | 780983 | 9 | 2 | 1 | 5 | 208 | 135 | 0.024368176 | 0.745 | 0.115 | 0.63 |  |  |
|  |  | 105075030 | - | 5137487 | 5137681 | 5134633 | 5136135 | 5141337 | 5141479 | 111 | 8 | 213 | 0 | 315 | 135 | 0.024920269 | 0.856 | 1 | -0.144 |  |  |
|  |  | 105073346 | - | 2934555 | 2934787 | 2933509 | 2933685 | 2944990 | 2945140 | 24 | 0 | 22 | 6 | 353 | 135 | 0.025424904 | 1 | 0.584 | 0.416 |  |  |
|  |  | 105072132 | - | 2820529 | 2820595 | 2815116 | 2816189 | 2822328 | 2822575 | 2 | 18 | 9 | 10 | 118 | 135 | 0.026139958 | 0.113 | 0.507 | -0.394 |  |  |
|  |  | 105072206 | - | 7226017 | 7226176 | 7222274 | 7222437 | 7227333 | 7227455 | 4 | 4 | 10 | 0 | 260 | 115 | 0.026224818 | 0.307 | 1 | -0.693 |  |  |
|  |  | 105067849 | + | 4181152 | 4181185 | 4170535 | 4170631 | 4200736 | 4200868 | 8 | 2 | 0 | 4 | 16 | 79 | 0.026330375 | 0.952 | 0 | 0.952 |  |  |
|  |  | 105071974 | + | 8620595 | 8620720 | 8618643 | 8618932 | 8625800 | 8625939 | 4 | 0 | 0 | 3 | 233 | 132 | 0.026450478 | 1 | 0 | 1 |  |  |
|  |  | 105063300 | - | 1538064 | 1538107 | 1534533 | 1534638 | 1547100 | 1547310 | 11 | 9 | 3 | 16 | 36 | 98 | 0.027903596 | 0.769 | 0.338 | 0.431 |  |  |
|  |  | 105064860 | + | 2685773 | 2685887 | 2685496 | 2685678 | 2686182 | 2686300 | 60 | 13 | 78 | 3 | 190 | 111 | 0.028150066 | 0.729 | 0.938 | -0.209 |  |  |
|  |  | 105080079 | - | 54257 | 54368 | 53843 | 54008 | 55060 | 55136 | 263 | 56 | 271 | 107 | 142 | 69 | 0.028477931 | 0.695 | 0.552 | 0.143 |  |  |
|  |  | 105072812 | - | 6042456 | 6042524 | 5997140 | 5997305 | 6043069 | 6043157 | 6 | 0 | 4 | 6 | 68 | 81 | 0.029245866 | 1 | 0.443 | 0.557 |  |  |
|  |  | 105064508 | - | 283012 | 283167 | 272490 | 272605 | 291685 | 291748 | 81 | 7 | 174 | 2 | 170 | 29 | 0.029460077 | 0.664 | 0.937 | -0.273 |  |  |
|  |  | 105074089 | - | 954328 | 954391 | 951575 | 951866 | 955084 | 955788 | 5 | 18 | 14 | 10 | 112 | 135 | 0.029507687 | 0.251 | 0.628 | -0.377 |  |  |
|  |  | 105076451 | - | 1349020 | 1349287 | 1348553 | 1348762 | 1349703 | 1349892 | 490 | 28 | 370 | 49 | 388 | 135 | 0.02962359 | 0.859 | 0.724 | 0.135 |  |  |
|  |  | 105082307 | - | 3171276 | 3171444 | 3170877 | 3171066 | 3171613 | 3171995 | 60 | 0 | 59 | 7 | 289 | 135 | 0.0297678 | 1 | 0.797 | 0.203 |  |  |
|  |  | 105065309 | - | 2179748 | 2179833 | 2176791 | 2176901 | 2182070 | 2182144 | 3 | 3 | 12 | 0 | 56 | 35 | 0.030079449 | 0.385 | 1 | -0.615 |  |  |
|  |  | 105063342 | + | 10129108 | 10129244 | 10127857 | 10128031 | 10131820 | 10131990 | 21 | 0 | 6 | 3 | 258 | 135 | 0.030221012 | 1 | 0.511 | 0.489 |  |  |
|  |  | 105072424 | + | 5425139 | 5425667 | 5420490 | 5420608 | 5431458 | 5431574 | 314 | 32 | 238 | 50 | 599 | 85 | 0.030230115 | 0.582 | 0.403 | 0.179 |  |  |
|  |  | 105064881 | - | 3083024 | 3083160 | 3082597 | 3082933 | 3083617 | 3083645 | 9 | 1 | 2 | 5 | 144 | 21 | 0.030335831 | 0.568 | 0.055 | 0.513 |  |  |
|  |  | 105070146 | + | 2712318 | 2712506 | 2711312 | 2711474 | 2712589 | 2712724 | 151 | 10 | 142 | 0 | 302 | 128 | 0.030511084 | 0.865 | 1 | -0.135 |  |  |
|  |  | 105070229 | - | 3214364 | 3215009 | 3211986 | 3212025 | 3215503 | 3215620 | 240 | 1 | 148 | 6 | 638 | 7 | 0.030581896 | 0.725 | 0.213 | 0.512 |  |  |
|  |  | 105081604 | - | 11162397 | 11162527 | 11158597 | 11158712 | 11173134 | 11173226 | 43 | 7 | 67 | 1 | 169 | 58 | 0.030707066 | 0.678 | 0.958 | -0.28 |  |  |
|  |  | 105082893 | + | 3733491 | 3733592 | 3668247 | 3668386 | 3787888 | 3788027 | 12 | 0 | 3 | 3 | 182 | 129 | 0.03078721 | 1 | 0.415 | 0.585 |  |  |
|  |  | 105065793 | - | 860272 | 860355 | 859439 | 859563 | 861700 | 862165 | 0 | 31 | 8 | 32 | 134 | 117 | 0.030849831 | 0 | 0.179 | -0.179 |  |  |
|  |  | 105063192 | + | 554594 | 554748 | 545591 | 545750 | 560072 | 560211 | 83 | 0 | 128 | 11 | 272 | 132 | 0.031348134 | 1 | 0.85 | 0.15 |  |  |
|  |  | 105065298 | - | 1286501 | 1286621 | 1259701 | 1262736 | 1296229 | 1296280 | 37 | 7 | 101 | 3 | 135 | 44 | 0.031702386 | 0.633 | 0.916 | -0.283 |  |  |
|  |  | 105082880 | + | 7045489 | 7045516 | 7044331 | 7044543 | 7046258 | 7046447 | 44 | 3 | 27 | 16 | 40 | 135 | 0.032116145 | 0.98 | 0.851 | 0.129 |  |  |
|  |  | 105078648 | - | 2054739 | 2054921 | 2053152 | 2053409 | 2060811 | 2060951 | 41 | 16 | 84 | 10 | 301 | 133 | 0.032349546 | 0.531 | 0.788 | -0.257 |  |  |
|  |  | 105081758 | - | 4982881 | 4982929 | 4971883 | 4972047 | 4985073 | 4985243 | 6 | 1 | 1 | 5 | 82 | 135 | 0.03251738 | 0.908 | 0.248 | 0.66 |  |  |
|  |  | 105075581 | + | 15980894 | 15981746 | 15979404 | 15979545 | 15982173 | 15982346 | 222 | 0 | 223 | 7 | 972 | 134 | 0.03294563 | 1 | 0.815 | 0.185 |  |  |
|  |  | 105073791 | + | 310314 | 310417 | 309303 | 309796 | 310772 | 311032 | 81 | 81 | 45 | 94 | 192 | 135 | 0.033012879 | 0.413 | 0.252 | 0.161 |  |  |
|  |  | 105079021 | + | 10701248 | 10701437 | 10696379 | 10696518 | 10705540 | 10705687 | 93 | 0 | 80 | 7 | 307 | 132 | 0.033083184 | 1 | 0.831 | 0.169 |  |  |
|  |  | 105069149 | - | 5609208 | 5609381 | 5605092 | 5605241 | 5609748 | 5609854 | 130 | 9 | 225 | 1 | 258 | 99 | 0.03386486 | 0.847 | 0.989 | -0.142 |  |  |
|  |  | 105079556 | - | 4218106 | 4218253 | 4217310 | 4217495 | 4219275 | 4219364 | 6 | 0 | 0 | 2 | 217 | 82 | 0.034460097 | 1 | 0 | 1 |  |  |
|  |  | 105071835 | + | 5162393 | 5162564 | 5147249 | 5147426 | 5170316 | 5170382 | 21 | 4 | 36 | 0 | 216 | 59 | 0.034611872 | 0.589 | 1 | -0.411 |  |  |
|  |  | 105072193 | + | 6021081 | 6021114 | 6017302 | 6017458 | 6025233 | 6025406 | 0 | 2 | 9 | 0 | 52 | 135 | 0.034654256 | 0 | 1 | -1 |  |  |
|  |  | 105067488 | + | 672105 | 672244 | 670801 | 670886 | 672474 | 672658 | 15 | 15 | 6 | 0 | 207 | 78 | 0.0347029 | 0.274 | 1 | -0.726 |  |  |
|  |  | 105061734 | + | 1306363 | 1306516 | 1286753 | 1286868 | 1321070 | 1321159 | 32 | 8 | 52 | 2 | 194 | 55 | 0.035002701 | 0.531 | 0.881 | -0.35 |  |  |
|  |  | 105062440 | + | 8731698 | 8731769 | 8729541 | 8729637 | 8732245 | 8732340 | 7 | 4 | 16 | 0 | 35 | 42 | 0.035033879 | 0.677 | 1 | -0.323 |  |  |
|  |  | 105067096 | - | 1302422 | 1302556 | 1301480 | 1301646 | 1320033 | 1320421 | 0 | 2 | 6 | 0 | 254 | 135 | 0.035129628 | 0 | 1 | -1 |  |  |
|  |  | 105079018 | - | 7340396 | 7340474 | 7339389 | 7339942 | 7350850 | 7350973 | 11 | 14 | 11 | 2 | 123 | 116 | 0.035472668 | 0.426 | 0.838 | -0.412 |  |  |
|  |  | 105065225 | + | 392091 | 392121 | 390510 | 391113 | 399062 | 402411 | 1 | 7 | 7 | 3 | 46 | 135 | 0.035805612 | 0.295 | 0.873 | -0.578 |  |  |
|  |  | 105064301 | + | 838753 | 838789 | 838307 | 838418 | 839800 | 842105 | 20 | 11 | 16 | 32 | 29 | 104 | 0.036062624 | 0.867 | 0.642 | 0.225 |  |  |
|  |  | 105069332 | + | 562593 | 562793 | 559771 | 560115 | 564777 | 564950 | 30 | 7 | 23 | 0 | 321 | 135 | 0.036539871 | 0.643 | 1 | -0.357 |  |  |
|  |  | 105071056 | - | 16303266 | 16303400 | 16303023 | 16303172 | 16303477 | 16303640 | 48 | 0 | 32 | 5 | 254 | 135 | 0.037234245 | 1 | 0.773 | 0.227 |  |  |
|  |  | 105078430 | + | 1229314 | 1231411 | 1227898 | 1228404 | 1232720 | 1232846 | 453 | 5 | 318 | 13 | 2202 | 119 | 0.03732472 | 0.83 | 0.569 | 0.261 |  |  |
|  |  | 105063954 | - | 5207738 | 5207868 | 5206786 | 5206956 | 5208702 | 5208792 | 108 | 16 | 120 | 4 | 194 | 83 | 0.037446979 | 0.743 | 0.928 | -0.185 |  |  |
|  |  | 105082219 | - | 6252056 | 6252180 | 6250852 | 6251102 | 6271703 | 6271870 | 1 | 2 | 25 | 1 | 234 | 135 | 0.037599133 | 0.224 | 0.935 | -0.711 |  |  |
|  |  | 105081417 | - | 1375073 | 1375235 | 1374349 | 1374481 | 1378374 | 1378554 | 3 | 8 | 6 | 1 | 273 | 125 | 0.037977066 | 0.147 | 0.733 | -0.586 |  |  |
|  |  | 105079408 | - | 10638685 | 10638839 | 10638267 | 10638359 | 10639557 | 10639739 | 56 | 7 | 45 | 0 | 225 | 85 | 0.03799901 | 0.751 | 1 | -0.249 |  |  |
|  |  | 105064019 | - | 6856233 | 6856398 | 6854568 | 6855393 | 6857453 | 6858053 | 32 | 11 | 14 | 0 | 286 | 135 | 0.038062928 | 0.579 | 1 | -0.421 |  |  |
|  |  | 105074697 | + | 3999283 | 3999388 | 3997598 | 3997790 | 4001547 | 4001763 | 5 | 3 | 16 | 0 | 196 | 135 | 0.038672407 | 0.534 | 1 | -0.466 |  |  |
|  |  | 105078375 | - | 2337040 | 2337124 | 2336410 | 2336752 | 2340951 | 2341075 | 12 | 31 | 3 | 0 | 136 | 117 | 0.038726751 | 0.25 | 1 | -0.75 |  |  |
|  |  | 105069072 | + | 1132211 | 1132392 | 1131727 | 1131913 | 1132673 | 1132853 | 36 | 2 | 18 | 7 | 302 | 135 | 0.038853856 | 0.889 | 0.535 | 0.354 |  |  |
|  |  | 105081649 | + | 1730158 | 1730289 | 1722523 | 1722808 | 1736095 | 1736207 | 27 | 7 | 72 | 3 | 218 | 105 | 0.039282457 | 0.65 | 0.92 | -0.27 |  |  |
|  |  | 105063924 | + | 2430528 | 2430714 | 2430125 | 2430267 | 2432160 | 2432329 | 8 | 4 | 4 | 15 | 307 | 135 | 0.039426633 | 0.468 | 0.105 | 0.363 |  |  |
|  |  | 105077494 | - | 332620 | 332802 | 332117 | 332359 | 333010 | 333069 | 72 | 0 | 41 | 4 | 220 | 52 | 0.03955805 | 1 | 0.708 | 0.292 |  |  |
|  |  | 105082194 | + | 28273605 | 28273757 | 28268633 | 28268653 | 28303695 | 28303911 | 13 | 0 | 13 | 6 | 151 | 13 | 0.039816774 | 1 | 0.157 | 0.843 |  |  |
|  |  | 105083441 | - | 1443121 | 1443217 | 1437565 | 1437672 | 1445581 | 1445701 | 52 | 11 | 29 | 19 | 121 | 78 | 0.04000093 | 0.753 | 0.496 | 0.257 |  |  |
|  |  | 105070333 | + | 13396773 | 13396873 | 13396546 | 13396614 | 13397143 | 13397291 | 5 | 11 | 0 | 24 | 112 | 61 | 0.040016657 | 0.198 | 0 | 0.198 |  |  |
|  |  | 105073953 | + | 9714822 | 9714897 | 9714516 | 9714645 | 9715027 | 9715144 | 60 | 52 | 28 | 55 | 98 | 97 | 0.04004395 | 0.533 | 0.335 | 0.198 |  |  |
|  |  | 105075855 | - | 152696 | 152829 | 150031 | 150152 | 155161 | 155305 | 0 | 4 | 4 | 1 | 231 | 114 | 0.040108455 | 0 | 0.664 | -0.664 |  |  |
|  |  | 105074331 | - | 838509 | 838673 | 838120 | 838247 | 838901 | 839069 | 195 | 4 | 155 | 16 | 270 | 120 | 0.040216581 | 0.956 | 0.812 | 0.144 |  |  |
|  |  | 105063829 | + | 3578316 | 3578432 | 3571851 | 3571926 | 3578634 | 3578727 | 12 | 4 | 19 | 0 | 102 | 19 | 0.040475372 | 0.358 | 1 | -0.642 |  |  |
|  |  | 105072388 | + | 9472801 | 9473138 | 9472229 | 9472477 | 9482375 | 9482452 | 186 | 6 | 337 | 0 | 393 | 70 | 0.041197491 | 0.847 | 1 | -0.153 |  |  |
|  |  | 105071870 | + | 9600080 | 9600134 | 9599818 | 9599852 | 9601089 | 9601442 | 26 | 41 | 70 | 47 | 47 | 27 | 0.041569479 | 0.267 | 0.461 | -0.194 |  |  |
|  |  | 105064645 | + | 210624 | 210812 | 210178 | 210395 | 211186 | 211225 | 65 | 0 | 43 | 4 | 206 | 32 | 0.041999788 | 1 | 0.625 | 0.375 |  |  |
|  |  | 105072363 | + | 7074985 | 7075039 | 7074375 | 7074483 | 7075720 | 7075788 | 2 | 49 | 16 | 72 | 13 | 27 | 0.042191893 | 0.078 | 0.316 | -0.238 |  |  |
|  |  | 105064800 | - | 1399395 | 1399473 | 1395445 | 1395553 | 1400299 | 1400372 | 6 | 2 | 1 | 6 | 39 | 32 | 0.042728452 | 0.711 | 0.12 | 0.591 |  |  |
|  |  | 105064615 | + | 195683 | 195885 | 195438 | 195582 | 196277 | 196408 | 21 | 7 | 40 | 2 | 312 | 124 | 0.043182305 | 0.544 | 0.888 | -0.344 |  |  |
|  |  | 105074937 | + | 6376765 | 6376871 | 6346189 | 6346345 | 6441728 | 6441797 | 12 | 0 | 10 | 5 | 125 | 62 | 0.043241558 | 1 | 0.498 | 0.502 |  |  |
|  |  | 105078453 | - | 5233907 | 5234242 | 5233660 | 5233779 | 5234425 | 5234484 | 151 | 3 | 199 | 16 | 350 | 29 | 0.043437041 | 0.807 | 0.508 | 0.299 |  |  |
|  |  | 105079851 | - | 1445283 | 1445349 | 1436042 | 1436148 | 1530127 | 1530192 | 18 | 8 | 8 | 15 | 23 | 22 | 0.043787082 | 0.683 | 0.338 | 0.345 |  |  |
|  |  | 105068185 | - | 2103347 | 2103440 | 2102756 | 2103019 | 2103562 | 2103625 | 65 | 54 | 89 | 35 | 93 | 56 | 0.044099254 | 0.42 | 0.605 | -0.185 |  |  |
|  |  | 105070708 | + | 638022 | 638165 | 637634 | 637718 | 638312 | 638424 | 43 | 0 | 7 | 2 | 182 | 47 | 0.044363593 | 1 | 0.475 | 0.525 |  |  |
|  |  | 105069232 | - | 11429675 | 11429772 | 11427784 | 11427926 | 11451901 | 11452027 | 0 | 2 | 5 | 0 | 164 | 119 | 0.045070912 | 0 | 1 | -1 |  |  |
|  |  | 105071974 | + | 8623711 | 8623868 | 8620595 | 8620720 | 8625800 | 8625939 | 5 | 4 | 9 | 0 | 258 | 115 | 0.045189323 | 0.358 | 1 | -0.642 |  |  |
|  |  | 105066977 | + | 127502 | 127615 | 116316 | 116515 | 136415 | 136603 | 76 | 8 | 78 | 0 | 212 | 135 | 0.045326934 | 0.858 | 1 | -0.142 |  |  |
|  |  | 105080331 | + | 1661844 | 1661963 | 1648587 | 1648739 | 1668131 | 1668192 | 56 | 5 | 86 | 0 | 143 | 54 | 0.045510081 | 0.809 | 1 | -0.191 |  |  |
|  |  | 105072191 | + | 5906805 | 5906877 | 5897520 | 5897700 | 5908170 | 5908278 | 0 | 5 | 2 | 0 | 96 | 101 | 0.045720714 | 0 | 1 | -1 |  |  |
|  |  | 105072886 | - | 1228414 | 1228552 | 1224332 | 1224626 | 1230574 | 1230706 | 4 | 10 | 9 | 3 | 252 | 125 | 0.045724878 | 0.166 | 0.598 | -0.432 |  |  |
|  |  | 105067292 | - | 6605072 | 6605175 | 6604291 | 6604423 | 6610668 | 6610842 | 55 | 1 | 41 | 8 | 182 | 125 | 0.045965898 | 0.974 | 0.779 | 0.195 |  |  |
|  |  | 105073738 | + | 8186025 | 8186454 | 8184362 | 8184535 | 8186653 | 8186811 | 334 | 0 | 303 | 10 | 550 | 135 | 0.046283522 | 1 | 0.881 | 0.119 |  |  |
|  |  | 105078397 | - | 4362947 | 4362984 | 4362653 | 4362695 | 4364280 | 4364549 | 0 | 2 | 5 | 0 | 30 | 35 | 0.046389177 | 0 | 1 | -1 |  |  |
|  |  | 105083805 | + | 3595170 | 3595218 | 3594982 | 3595011 | 3595745 | 3595907 | 5 | 1 | 0 | 3 | 41 | 22 | 0.046483074 | 0.728 | 0 | 0.728 |  |  |
|  |  | 105067520 | + | 2006385 | 2006430 | 2003891 | 2004017 | 2006869 | 2006965 | 24 | 15 | 23 | 43 | 22 | 73 | 0.04649761 | 0.841 | 0.64 | 0.201 |  |  |
|  |  | 105066705 | + | 4093523 | 4093623 | 4092579 | 4092729 | 4093945 | 4094082 | 2 | 4 | 5 | 0 | 181 | 130 | 0.046647792 | 0.264 | 1 | -0.736 |  |  |
|  |  | 105083742 | + | 281411 | 281485 | 273390 | 273593 | 284097 | 284250 | 4 | 5 | 11 | 1 | 134 | 135 | 0.046815321 | 0.446 | 0.917 | -0.471 |  |  |
|  |  | 105080125 | + | 794724 | 794841 | 793682 | 793873 | 795448 | 795575 | 97 | 1 | 85 | 10 | 205 | 120 | 0.046892055 | 0.983 | 0.833 | 0.15 |  |  |
|  |  | 105063841 | - | 3750368 | 3750503 | 3750056 | 3750191 | 3752531 | 3752648 | 10 | 55 | 54 | 96 | 224 | 103 | 0.047502764 | 0.077 | 0.205 | -0.128 |  |  |
|  |  | 105069383 | + | 1750862 | 1750990 | 1727994 | 1728106 | 1752174 | 1752528 | 5 | 0 | 1 | 3 | 212 | 105 | 0.047689049 | 1 | 0.142 | 0.858 |  |  |
|  |  | 105081091 | - | 5125752 | 5125957 | 5123323 | 5124074 | 5127853 | 5127940 | 3 | 3 | 9 | 0 | 271 | 80 | 0.04771025 | 0.228 | 1 | -0.772 |  |  |
|  |  | 105072033 | + | 7098417 | 7098454 | 7093318 | 7093357 | 7106595 | 7106733 | 1 | 3 | 5 | 0 | 26 | 28 | 0.047919762 | 0.264 | 1 | -0.736 |  |  |
|  |  | 105073454 | - | 2709660 | 2709757 | 2706492 | 2706611 | 2719944 | 2720014 | 76 | 25 | 78 | 9 | 85 | 40 | 0.047941989 | 0.589 | 0.803 | -0.214 |  |  |
|  |  | 105071565 | + | 4133062 | 4133218 | 4132653 | 4132789 | 4133307 | 4134047 | 32 | 7 | 36 | 25 | 271 | 129 | 0.047943601 | 0.685 | 0.407 | 0.278 |  |  |
|  |  | 105066416 | - | 217179 | 217302 | 213820 | 213923 | 217587 | 217743 | 64 | 4 | 40 | 11 | 193 | 96 | 0.047962991 | 0.888 | 0.644 | 0.244 |  |  |
|  |  | 105081023 | + | 4746544 | 4746576 | 4737761 | 4737854 | 4751188 | 4753720 | 12 | 7 | 9 | 23 | 25 | 86 | 0.048165534 | 0.855 | 0.574 | 0.281 |  |  |
|  |  | 105080852 | + | 483991 | 484057 | 465193 | 465383 | 489793 | 489892 | 1 | 3 | 5 | 0 | 75 | 92 | 0.048286051 | 0.29 | 1 | -0.71 |  |  |
|  |  | 105062002 | + | 605023 | 605120 | 596765 | 596848 | 617226 | 617414 | 141 | 14 | 173 | 2 | 121 | 76 | 0.048636762 | 0.863 | 0.982 | -0.119 |  |  |
|  |  | 105079460 | - | 2789275 | 2789391 | 2784409 | 2784904 | 2812682 | 2812811 | 5 | 10 | 10 | 3 | 205 | 122 | 0.049481088 | 0.229 | 0.665 | -0.436 |  |  |
|  |  | 105065412 | - | 42783 | 42907 | 42172 | 42328 | 43609 | 43672 | 40 | 6 | 68 | 1 | 155 | 56 | 0.049510745 | 0.707 | 0.961 | -0.254 |  |  |
|  |  | 105072372 | - | 8399584 | 8399677 | 8398324 | 8398512 | 8400798 | 8400983 | 465 | 0 | 539 | 30 | 172 | 135 | 0.04967824 | 1 | 0.934 | 0.066 |  |  |
|  | WS | 105078587 | - | 2404126 | 2404185 | 2403576 | 2403722 | 2406419 | 2406466 | 82 | 22 | 448 | 723 | 52 | 40 | 1.86E-12 | 0.741 | 0.323 | 0.418 |  |  |
|  |  | 105079841 | + | 952 | 1056 | 3 | 90 | 2968 | 3072 | 1 | 34 | 32 | 17 | 101 | 42 | 2.62E-08 | 0.012 | 0.439 | -0.427 |  |  |
|  |  | 105082303 | - | 3042777 | 3042846 | 3041884 | 3042026 | 3047152 | 3047215 | 0 | 33 | 96 | 103 | 62 | 56 | 3.04E-08 | 0 | 0.457 | -0.457 |  |  |
|  |  | 105072055 | - | 11256329 | 11256387 | 11246567 | 11246690 | 11273281 | 11273346 | 35 | 40 | 19 | 169 | 32 | 39 | 4.85E-08 | 0.516 | 0.121 | 0.395 |  |  |
|  |  | 105082299 | - | 2906267 | 2906353 | 2898101 | 2898439 | 2906917 | 2907176 | 90 | 0 | 170 | 55 | 158 | 135 | 6.96E-08 | 1 | 0.725 | 0.275 |  |  |
|  |  | 105084011 | - | 649435 | 649517 | 649197 | 649329 | 649813 | 650016 | 8 | 23 | 32 | 3 | 140 | 125 | 2.26E-07 | 0.237 | 0.905 | -0.668 |  |  |
|  |  | 105082307 | - | 3170877 | 3171066 | 3169721 | 3170173 | 3171276 | 3171444 | 104 | 5 | 64 | 34 | 310 | 135 | 3.52E-07 | 0.901 | 0.45 | 0.451 |  |  |
|  |  | 105074461 | + | 5472985 | 5473082 | 5472792 | 5472907 | 5473588 | 5473749 | 53 | 34 | 42 | 0 | 153 | 108 | 6.19E-07 | 0.524 | 1 | -0.476 |  |  |
|  |  | 105063806 | + | 2395924 | 2396016 | 2390502 | 2390693 | 2397575 | 2397653 | 1 | 6 | 51 | 0 | 106 | 71 | 1.25E-06 | 0.1 | 1 | -0.9 |  |  |
|  |  | 105066970 | - | 294784 | 294888 | 293367 | 294658 | 295454 | 295504 | 417 | 13 | 711 | 105 | 102 | 43 | 3.30E-06 | 0.931 | 0.741 | 0.19 |  |  |
|  |  | 105081122 | - | 1224190 | 1224490 | 1214561 | 1214702 | 1229147 | 1229465 | 134 | 2 | 113 | 28 | 420 | 134 | 3.35E-06 | 0.955 | 0.563 | 0.392 |  |  |
|  |  | 105074852 | + | 6959792 | 6959895 | 6959544 | 6959602 | 6966839 | 6966982 | 9 | 0 | 1 | 20 | 108 | 51 | 3.51E-06 | 1 | 0.023 | 0.977 |  |  |
|  |  | 105079730 | - | 3973472 | 3973544 | 3972662 | 3972805 | 3973827 | 3973891 | 14 | 9 | 12 | 122 | 65 | 57 | 4.20E-06 | 0.577 | 0.079 | 0.498 |  |  |
|  |  | 105072701 | - | 8507743 | 8507797 | 8506962 | 8507116 | 8514312 | 8514576 | 28 | 15 | 18 | 74 | 94 | 135 | 4.94E-06 | 0.728 | 0.259 | 0.469 |  |  |
|  |  | 105066632 | - | 8757073 | 8757221 | 8753210 | 8753331 | 8758969 | 8759079 | 135 | 10 | 176 | 68 | 217 | 82 | 5.80E-06 | 0.836 | 0.494 | 0.342 |  |  |
|  |  | 105072055 | - | 11256329 | 11256557 | 11246567 | 11246690 | 11273281 | 11273424 | 52 | 40 | 44 | 169 | 330 | 116 | 6.58E-06 | 0.314 | 0.084 | 0.23 |  |  |
|  |  | 105076509 | - | 10237163 | 10237253 | 10236076 | 10236283 | 10256383 | 10256614 | 36 | 75 | 97 | 51 | 166 | 135 | 6.84E-06 | 0.281 | 0.607 | -0.326 |  |  |
|  |  | 105083092 | - | 3523621 | 3523764 | 3521311 | 3521444 | 3524460 | 3524557 | 0 | 10 | 14 | 1 | 216 | 81 | 7.80E-06 | 0 | 0.84 | -0.84 |  |  |
|  |  | 105074934 | - | 5921357 | 5921766 | 5918041 | 5918260 | 5921967 | 5922125 | 178 | 20 | 200 | 0 | 530 | 135 | 8.55E-06 | 0.694 | 1 | -0.306 |  |  |
|  |  | 105080230 | + | 2158168 | 2158231 | 2157962 | 2158082 | 2158334 | 2158459 | 0 | 13 | 8 | 0 | 73 | 96 | 9.92E-06 | 0 | 1 | -1 |  |  |
|  |  | 105081100 | - | 812264 | 812385 | 808138 | 808366 | 815787 | 815894 | 33 | 17 | 119 | 5 | 193 | 100 | 1.04E-05 | 0.501 | 0.925 | -0.424 |  |  |
|  |  | 105071735 | - | 935119 | 935948 | 929009 | 929102 | 936534 | 936730 | 69 | 0 | 50 | 16 | 901 | 86 | 1.32E-05 | 1 | 0.23 | 0.77 |  |  |
|  |  | 105065299 | - | 1509585 | 1509744 | 1507121 | 1507322 | 1509960 | 1510038 | 14 | 13 | 90 | 6 | 216 | 71 | 1.34E-05 | 0.261 | 0.831 | -0.57 |  |  |
|  |  | 105069161 | - | 6441767 | 6441923 | 6437631 | 6437774 | 6444211 | 6444352 | 84 | 35 | 107 | 6 | 276 | 134 | 1.60E-05 | 0.538 | 0.896 | -0.358 |  |  |
|  |  | 105080613 | + | 5545160 | 5545269 | 5542211 | 5542319 | 5548798 | 5548966 | 7 | 6 | 85 | 0 | 170 | 101 | 2.07E-05 | 0.409 | 1 | -0.591 |  |  |
|  |  | 105062413 | - | 10572337 | 10572430 | 10561754 | 10565181 | 10572760 | 10572853 | 63 | 17 | 40 | 54 | 123 | 86 | 2.12E-05 | 0.722 | 0.341 | 0.381 |  |  |
|  |  | 105076002 | + | 186366 | 186450 | 184581 | 184755 | 196020 | 196207 | 28 | 6 | 0 | 9 | 154 | 135 | 2.18E-05 | 0.804 | 0 | 0.804 |  |  |
|  |  | 105064173 | + | 875726 | 875744 | 874964 | 875108 | 876032 | 876161 | 12 | 1 | 11 | 46 | 11 | 122 | 2.23E-05 | 0.993 | 0.726 | 0.267 |  |  |
|  |  | 105076527 | + | 11895678 | 11895874 | 11894322 | 11894392 | 11898935 | 11899030 | 36 | 10 | 91 | 0 | 198 | 16 | 2.47E-05 | 0.225 | 1 | -0.775 |  |  |
|  |  | 105082013 | - | 487196 | 487390 | 485670 | 485976 | 489005 | 489151 | 4 | 6 | 67 | 1 | 315 | 135 | 2.63E-05 | 0.222 | 0.966 | -0.744 |  |  |
|  |  | 105080718 | + | 1550408 | 1550518 | 1547985 | 1548051 | 1551433 | 1551546 | 16 | 11 | 83 | 3 | 101 | 30 | 2.69E-05 | 0.302 | 0.892 | -0.59 |  |  |
|  |  | 105071724 | + | 1308416 | 1309304 | 1293562 | 1294238 | 1310480 | 1310515 | 197 | 0 | 160 | 15 | 902 | 28 | 2.96E-05 | 1 | 0.249 | 0.751 |  |  |
|  |  | 105067390 | + | 15436658 | 15436808 | 15434479 | 15434632 | 15437339 | 15437480 | 52 | 13 | 100 | 0 | 270 | 134 | 2.98E-05 | 0.665 | 1 | -0.335 |  |  |
|  |  | 105071633 | + | 4209220 | 4209349 | 4208844 | 4208951 | 4211549 | 4212637 | 65 | 20 | 84 | 1 | 209 | 100 | 3.31E-05 | 0.609 | 0.976 | -0.367 |  |  |
|  |  | 105064994 | - | 3827042 | 3827201 | 3823866 | 3824053 | 3832307 | 3832414 | 3 | 8 | 21 | 0 | 245 | 100 | 3.33E-05 | 0.133 | 1 | -0.867 |  |  |
|  |  | 105076953 | + | 8590395 | 8590526 | 8589926 | 8590155 | 8591029 | 8591090 | 4 | 17 | 43 | 13 | 167 | 54 | 3.61E-05 | 0.071 | 0.517 | -0.446 |  |  |
|  |  | 105083775 | + | 2541047 | 2541128 | 2536534 | 2536677 | 2543215 | 2543355 | 22 | 0 | 14 | 16 | 146 | 133 | 3.77E-05 | 1 | 0.444 | 0.556 |  |  |
|  |  | 105066588 | - | 4263138 | 4263240 | 4262701 | 4262854 | 4264112 | 4264205 | 19 | 9 | 204 | 4 | 141 | 86 | 4.29E-05 | 0.563 | 0.969 | -0.406 |  |  |
|  |  | 105065298 | - | 1286501 | 1286621 | 1259701 | 1262736 | 1296229 | 1296280 | 11 | 8 | 101 | 3 | 135 | 44 | 5.13E-05 | 0.309 | 0.916 | -0.607 |  |  |
|  |  | 105063847 | - | 3799692 | 3799774 | 3799286 | 3799388 | 3799880 | 3799986 | 94 | 27 | 84 | 1 | 74 | 59 | 5.86E-05 | 0.735 | 0.985 | -0.25 |  |  |
|  |  | 105082223 | + | 8401631 | 8401835 | 8396260 | 8396438 | 8402586 | 8402769 | 62 | 16 | 197 | 5 | 325 | 135 | 7.34E-05 | 0.617 | 0.942 | -0.325 |  |  |
|  |  | 105079102 | - | 1682019 | 1682395 | 1681371 | 1681455 | 1698850 | 1698989 | 35 | 0 | 14 | 10 | 436 | 74 | 7.38E-05 | 1 | 0.192 | 0.808 |  |  |
|  |  | 105070332 | + | 13378531 | 13378597 | 13377809 | 13377924 | 13378718 | 13378908 | 14 | 11 | 34 | 0 | 91 | 108 | 8.86E-05 | 0.602 | 1 | -0.398 |  |  |
|  |  | 105061593 | - | 7905233 | 7905377 | 7899741 | 7899847 | 7940046 | 7940124 | 3 | 6 | 51 | 2 | 170 | 35 | 9.09E-05 | 0.093 | 0.84 | -0.747 |  |  |
|  |  | 105079018 | - | 7340396 | 7340474 | 7339389 | 7339942 | 7350850 | 7350973 | 4 | 23 | 11 | 2 | 123 | 116 | 0.000106288 | 0.141 | 0.838 | -0.697 |  |  |
|  |  | 105079663 | - | 3280413 | 3280487 | 3278433 | 3278599 | 3281418 | 3281513 | 0 | 10 | 20 | 7 | 87 | 88 | 0.000110759 | 0 | 0.743 | -0.743 |  |  |
|  |  | 105073541 | - | 2986892 | 2986970 | 2967224 | 2967315 | 2990780 | 2990853 | 67 | 3 | 85 | 35 | 22 | 15 | 0.000131995 | 0.938 | 0.623 | 0.315 |  |  |
|  |  | 105081805 | + | 7942711 | 7943434 | 7938113 | 7938226 | 7947986 | 7948131 | 105 | 2 | 41 | 12 | 815 | 106 | 0.000142485 | 0.872 | 0.308 | 0.564 |  |  |
|  |  | 105081023 | + | 4688895 | 4688934 | 4688069 | 4688206 | 4693320 | 4693386 | 13 | 0 | 10 | 18 | 27 | 54 | 0.000151738 | 1 | 0.526 | 0.474 |  |  |
|  |  | 105062674 | - | 1326407 | 1326459 | 1313120 | 1313422 | 1332451 | 1332564 | 0 | 6 | 11 | 0 | 61 | 106 | 0.00015775 | 0 | 1 | -1 |  |  |
|  |  | 105068062 | + | 4587191 | 4587362 | 4577945 | 4578206 | 4594403 | 4594463 | 9 | 2 | 0 | 14 | 210 | 53 | 0.000158072 | 0.532 | 0 | 0.532 |  |  |
|  |  | 105063438 | + | 1319930 | 1319993 | 1319174 | 1319322 | 1321010 | 1321243 | 8 | 22 | 15 | 2 | 112 | 135 | 0.00016153 | 0.305 | 0.9 | -0.595 |  |  |
|  |  | 105067520 | + | 2006385 | 2006430 | 2003891 | 2004017 | 2006869 | 2006965 | 14 | 1 | 23 | 43 | 22 | 73 | 0.000161994 | 0.979 | 0.64 | 0.339 |  |  |
|  |  | 105077924 | - | 4371000 | 4371096 | 4367907 | 4368094 | 4383585 | 4383649 | 8 | 15 | 33 | 5 | 100 | 57 | 0.000172957 | 0.233 | 0.79 | -0.557 |  |  |
|  |  | 105079353 | + | 1119362 | 1119464 | 1118327 | 1118423 | 1120115 | 1120511 | 34 | 4 | 15 | 19 | 144 | 89 | 0.000181029 | 0.84 | 0.328 | 0.512 |  |  |
|  |  | 105075776 | - | 2036230 | 2036335 | 2033751 | 2033853 | 2037142 | 2038097 | 36 | 45 | 119 | 42 | 156 | 95 | 0.000191812 | 0.328 | 0.633 | -0.305 |  |  |
|  |  | 105074722 | + | 14089726 | 14089859 | 14078636 | 14080343 | 14095367 | 14095552 | 41 | 42 | 86 | 22 | 252 | 135 | 0.000201435 | 0.343 | 0.677 | -0.334 |  |  |
|  |  | 105081100 | - | 814927 | 815063 | 808138 | 808366 | 815787 | 815894 | 18 | 17 | 49 | 5 | 223 | 100 | 0.000206081 | 0.322 | 0.815 | -0.493 |  |  |
|  |  | 105076527 | + | 11898061 | 11898215 | 11894322 | 11894392 | 11898935 | 11899030 | 34 | 10 | 60 | 0 | 156 | 16 | 0.000210587 | 0.259 | 1 | -0.741 |  |  |
|  |  | 105073014 | - | 740404 | 740487 | 739437 | 739914 | 740860 | 741005 | 5 | 10 | 21 | 1 | 152 | 135 | 0.000212211 | 0.308 | 0.949 | -0.641 |  |  |
|  |  | 105072914 | + | 8393627 | 8393852 | 8384410 | 8384503 | 8395513 | 8395652 | 21 | 25 | 1 | 43 | 294 | 83 | 0.000222487 | 0.192 | 0.007 | 0.185 |  |  |
|  |  | 105079442 | + | 11039511 | 11039571 | 11037737 | 11038127 | 11042751 | 11043153 | 62 | 32 | 32 | 62 | 106 | 135 | 0.000224339 | 0.712 | 0.397 | 0.315 |  |  |
|  |  | 105063988 | + | 4879324 | 4879439 | 4876333 | 4876525 | 4880570 | 4880709 | 7 | 6 | 65 | 1 | 213 | 132 | 0.000246488 | 0.42 | 0.976 | -0.556 |  |  |
|  |  | 105077813 | - | 265070 | 265139 | 261293 | 263290 | 265667 | 265794 | 79 | 1 | 37 | 14 | 109 | 120 | 0.000258018 | 0.989 | 0.744 | 0.245 |  |  |
|  |  | 105069416 | - | 316012 | 316170 | 311503 | 311661 | 325727 | 325866 | 263 | 36 | 538 | 15 | 276 | 132 | 0.000259579 | 0.777 | 0.945 | -0.168 |  |  |
|  |  | 105068380 | + | 1384261 | 1384525 | 1382543 | 1382807 | 1385663 | 1385892 | 44 | 0 | 9 | 6 | 385 | 135 | 0.000263688 | 1 | 0.345 | 0.655 |  |  |
|  |  | 105073795 | - | 349330 | 349423 | 348234 | 349126 | 349723 | 349936 | 45 | 21 | 76 | 4 | 172 | 135 | 0.000265413 | 0.627 | 0.937 | -0.31 |  |  |
|  |  | 105081608 | - | 11658307 | 11658504 | 11656873 | 11657114 | 11663377 | 11663521 | 4 | 8 | 16 | 0 | 318 | 135 | 0.00027986 | 0.175 | 1 | -0.825 |  |  |
|  |  | 105064246 | - | 4007095 | 4007365 | 4004596 | 4004878 | 4012628 | 4012797 | 17 | 12 | 25 | 0 | 391 | 135 | 0.000300105 | 0.328 | 1 | -0.672 |  |  |
|  |  | 105081955 | - | 7754266 | 7754388 | 7749370 | 7749508 | 7768625 | 7768782 | 0 | 4 | 17 | 0 | 226 | 131 | 0.000300613 | 0 | 1 | -1 |  |  |
|  |  | 105074938 | - | 6632000 | 6632102 | 6630332 | 6630450 | 6635051 | 6635195 | 3 | 6 | 32 | 1 | 166 | 111 | 0.000302672 | 0.251 | 0.955 | -0.704 |  |  |
|  |  | 105067286 | + | 6215071 | 6215175 | 6186341 | 6186442 | 6237618 | 6237736 | 318 | 133 | 125 | 116 | 129 | 70 | 0.00030864 | 0.565 | 0.369 | 0.196 |  |  |
|  |  | 105080051 | + | 1039429 | 1039535 | 1029667 | 1029734 | 1040742 | 1040851 | 2 | 5 | 22 | 0 | 90 | 27 | 0.000310641 | 0.107 | 1 | -0.893 |  |  |
|  |  | 105079690 | + | 13409694 | 13409854 | 13408353 | 13408488 | 13410737 | 13410911 | 93 | 12 | 180 | 0 | 274 | 128 | 0.000311151 | 0.784 | 1 | -0.216 |  |  |
|  |  | 105075026 | + | 4143512 | 4143697 | 4142947 | 4143061 | 4147134 | 4147261 | 15 | 5 | 117 | 0 | 263 | 92 | 0.000314361 | 0.512 | 1 | -0.488 |  |  |
|  |  | 105082952 | + | 13080692 | 13080770 | 13080359 | 13080448 | 13085003 | 13085217 | 34 | 8 | 40 | 52 | 89 | 82 | 0.000323419 | 0.797 | 0.415 | 0.382 |  |  |
|  |  | 105077918 | - | 3149575 | 3149620 | 3146382 | 3146471 | 3150871 | 3151011 | 25 | 52 | 17 | 158 | 36 | 80 | 0.000331301 | 0.517 | 0.193 | 0.324 |  |  |
|  |  | 105079822 | - | 3101156 | 3101292 | 3100073 | 3100615 | 3102661 | 3102744 | 1 | 16 | 40 | 29 | 199 | 76 | 0.000333463 | 0.023 | 0.345 | -0.322 |  |  |
|  |  | 105071434 | - | 8904588 | 8904756 | 8902637 | 8902964 | 8907998 | 8908165 | 6 | 0 | 0 | 9 | 289 | 135 | 0.000348171 | 1 | 0 | 1 |  |  |
|  |  | 105064881 | - | 3083024 | 3083160 | 3082597 | 3082933 | 3083717 | 3083912 | 18 | 4 | 5 | 17 | 258 | 135 | 0.000352953 | 0.702 | 0.133 | 0.569 |  |  |
|  |  | 105072503 | + | 2559673 | 2559814 | 2558359 | 2558584 | 2560304 | 2560414 | 114 | 21 | 132 | 77 | 236 | 103 | 0.000356098 | 0.703 | 0.428 | 0.275 |  |  |
|  |  | 105077573 | - | 290016 | 290082 | 289573 | 289872 | 290305 | 290750 | 39 | 31 | 30 | 2 | 118 | 135 | 0.000358252 | 0.59 | 0.945 | -0.355 |  |  |
|  |  | 105081805 | + | 7944045 | 7944408 | 7938113 | 7938226 | 7947986 | 7948131 | 178 | 2 | 75 | 12 | 455 | 106 | 0.000360435 | 0.954 | 0.593 | 0.361 |  |  |
|  |  | 105066785 | - | 1906339 | 1906463 | 1906102 | 1906253 | 1907854 | 1907947 | 9 | 6 | 42 | 0 | 185 | 86 | 0.000362932 | 0.411 | 1 | -0.589 |  |  |
|  |  | 105081372 | + | 10038097 | 10038183 | 10037672 | 10037816 | 10041958 | 10042068 | 9 | 6 | 50 | 0 | 126 | 103 | 0.000379895 | 0.551 | 1 | -0.449 |  |  |
|  |  | 105074187 | + | 1444842 | 1445063 | 1442836 | 1442956 | 1445763 | 1445874 | 22 | 14 | 59 | 4 | 289 | 82 | 0.000435071 | 0.308 | 0.807 | -0.499 |  |  |
|  |  | 105082299 | - | 2899326 | 2899518 | 2898101 | 2898439 | 2906917 | 2907176 | 13 | 0 | 52 | 55 | 313 | 135 | 0.00044901 | 1 | 0.29 | 0.71 |  |  |
|  |  | 105072812 | - | 6042456 | 6042524 | 6019147 | 6019254 | 6043069 | 6043157 | 23 | 4 | 14 | 24 | 33 | 46 | 0.000455491 | 0.889 | 0.448 | 0.441 |  |  |
|  |  | 105073034 | - | 3121312 | 3121496 | 3100955 | 3101373 | 3122548 | 3122687 | 230 | 2 | 347 | 35 | 302 | 132 | 0.000504336 | 0.98 | 0.813 | 0.167 |  |  |
|  |  | 105062412 | + | 10502973 | 10503046 | 10483675 | 10483974 | 10505895 | 10506080 | 41 | 26 | 49 | 109 | 132 | 135 | 0.000508128 | 0.617 | 0.315 | 0.302 |  |  |
|  |  | 105079730 | - | 3973827 | 3973934 | 3972662 | 3972805 | 3975275 | 3975375 | 20 | 6 | 212 | 0 | 158 | 93 | 0.00051819 | 0.662 | 1 | -0.338 |  |  |
|  |  | 105071417 | - | 5788465 | 5788582 | 5786873 | 5787282 | 5788857 | 5788938 | 171 | 65 | 222 | 189 | 159 | 74 | 0.000535397 | 0.55 | 0.353 | 0.197 |  |  |
|  |  | 105083320 | + | 2832772 | 2832984 | 2828532 | 2828925 | 2840428 | 2843924 | 19 | 16 | 70 | 9 | 333 | 135 | 0.000569977 | 0.325 | 0.759 | -0.434 |  |  |
|  |  | 105081613 | - | 11880021 | 11880170 | 11874661 | 11874818 | 11881791 | 11881934 | 18 | 0 | 23 | 18 | 270 | 135 | 0.000585694 | 1 | 0.39 | 0.61 |  |  |
|  |  | 105080897 | - | 50530 | 50702 | 49210 | 49345 | 63418 | 63482 | 313 | 95 | 242 | 28 | 208 | 50 | 0.000599114 | 0.442 | 0.675 | -0.233 |  |  |
|  |  | 105080967 | - | 1568242 | 1568281 | 1562141 | 1563134 | 1573600 | 1573779 | 7 | 7 | 0 | 23 | 64 | 135 | 0.000601928 | 0.678 | 0 | 0.678 |  |  |
|  |  | 105067063 | - | 2814474 | 2814579 | 2809211 | 2811842 | 2820812 | 2820966 | 11 | 26 | 67 | 32 | 196 | 135 | 0.000614013 | 0.226 | 0.591 | -0.365 |  |  |
|  |  | 105067917 | - | 9350907 | 9351050 | 9349673 | 9349801 | 9352239 | 9352410 | 2 | 4 | 49 | 1 | 256 | 121 | 0.000628981 | 0.191 | 0.959 | -0.768 |  |  |
|  |  | 105081656 | - | 4408553 | 4408703 | 4407127 | 4407179 | 4409809 | 4409995 | 6 | 6 | 50 | 2 | 181 | 45 | 0.000728871 | 0.199 | 0.861 | -0.662 |  |  |
|  |  | 105063871 | + | 5324213 | 5324374 | 5319466 | 5319589 | 5327929 | 5328393 | 4 | 8 | 13 | 0 | 263 | 116 | 0.000734069 | 0.181 | 1 | -0.819 |  |  |
|  |  | 105061854 | + | 298865 | 298905 | 295126 | 295259 | 308078 | 308167 | 0 | 9 | 12 | 4 | 24 | 73 | 0.000734345 | 0 | 0.901 | -0.901 |  |  |
|  |  | 105062889 | + | 1695077 | 1695178 | 1694274 | 1694404 | 1695855 | 1695887 | 132 | 96 | 93 | 21 | 82 | 13 | 0.000748142 | 0.179 | 0.412 | -0.233 |  |  |
|  |  | 105066416 | - | 238916 | 239052 | 235174 | 235287 | 239261 | 239366 | 11 | 8 | 37 | 1 | 192 | 69 | 0.000833225 | 0.331 | 0.93 | -0.599 |  |  |
|  |  | 105066101 | - | 4286357 | 4286495 | 4284489 | 4284969 | 4292412 | 4292584 | 16 | 1 | 4 | 9 | 262 | 135 | 0.000839075 | 0.892 | 0.186 | 0.706 |  |  |
|  |  | 105066885 | + | 613600 | 613674 | 612635 | 612746 | 613788 | 613914 | 32 | 10 | 67 | 0 | 87 | 88 | 0.000851899 | 0.764 | 1 | -0.236 |  |  |
|  |  | 105072599 | - | 620454 | 620478 | 619156 | 619317 | 621044 | 621118 | 8 | 71 | 45 | 91 | 17 | 67 | 0.000874968 | 0.308 | 0.661 | -0.353 |  |  |
|  |  | 105076509 | - | 10252236 | 10252353 | 10236076 | 10236283 | 10256383 | 10256614 | 5 | 75 | 26 | 51 | 220 | 135 | 0.000876767 | 0.039 | 0.238 | -0.199 |  |  |
|  |  | 105064040 | + | 7336510 | 7336644 | 7335281 | 7335471 | 7336815 | 7336936 | 48 | 0 | 59 | 15 | 233 | 114 | 0.000888758 | 1 | 0.658 | 0.342 |  |  |
|  |  | 105078587 | - | 2404126 | 2404185 | 2403576 | 2403722 | 2406437 | 2406467 | 82 | 115 | 448 | 1608 | 52 | 23 | 0.000906012 | 0.24 | 0.11 | 0.13 |  |  |
|  |  | 105081237 | + | 5409238 | 5409392 | 5406781 | 5406838 | 5413683 | 5414059 | 23 | 0 | 6 | 6 | 190 | 50 | 0.00093631 | 1 | 0.208 | 0.792 |  |  |
|  |  | 105077455 | + | 39484827 | 39485000 | 39479219 | 39479655 | 39498818 | 39499004 | 8 | 6 | 29 | 0 | 294 | 135 | 0.000948168 | 0.38 | 1 | -0.62 |  |  |
|  |  | 105062496 | + | 1150334 | 1150658 | 1143974 | 1144038 | 1155875 | 1156039 | 290 | 0 | 248 | 12 | 367 | 57 | 0.001022118 | 1 | 0.762 | 0.238 |  |  |
|  |  | 105075211 | + | 1616939 | 1617060 | 1616579 | 1616820 | 1617214 | 1617363 | 34 | 8 | 80 | 0 | 228 | 135 | 0.00110153 | 0.716 | 1 | -0.284 |  |  |
|  |  | 105072022 | - | 5073378 | 5073433 | 4940940 | 4941125 | 5134355 | 5134469 | 15 | 10 | 27 | 0 | 68 | 107 | 0.001109692 | 0.702 | 1 | -0.298 |  |  |
|  |  | 105076229 | + | 4928496 | 4928616 | 4897325 | 4897424 | 4931026 | 4931127 | 8 | 23 | 56 | 29 | 142 | 51 | 0.001142787 | 0.111 | 0.41 | -0.299 |  |  |
|  |  | 105070214 | + | 2490747 | 2490907 | 2488561 | 2488735 | 2493110 | 2494890 | 52 | 10 | 76 | 0 | 281 | 135 | 0.001163412 | 0.714 | 1 | -0.286 |  |  |
|  |  | 105082910 | + | 6653879 | 6654059 | 6653428 | 6653562 | 6655201 | 6655434 | 60 | 8 | 179 | 0 | 293 | 127 | 0.001181791 | 0.765 | 1 | -0.235 |  |  |
|  |  | 105064745 | - | 234158 | 234420 | 233682 | 233787 | 235382 | 235452 | 0 | 6 | 8 | 0 | 274 | 26 | 0.001229619 | 0 | 1 | -1 |  |  |
|  |  | 105070936 | - | 6133503 | 6133980 | 6109674 | 6109835 | 6136971 | 6137292 | 283 | 15 | 422 | 1 | 598 | 135 | 0.001238497 | 0.81 | 0.99 | -0.18 |  |  |
|  |  | 105082180 | - | 27694634 | 27694816 | 27693990 | 27694147 | 27694983 | 27695051 | 0 | 14 | 23 | 21 | 229 | 61 | 0.001244312 | 0 | 0.226 | -0.226 |  |  |
|  |  | 105076485 | - | 3496860 | 3496925 | 3471218 | 3471301 | 3497526 | 3497654 | 14 | 11 | 4 | 30 | 44 | 62 | 0.00126855 | 0.642 | 0.158 | 0.484 |  |  |
|  |  | 105065118 | + | 690526 | 690613 | 689858 | 690062 | 691370 | 691501 | 58 | 12 | 139 | 1 | 149 | 124 | 0.001291321 | 0.801 | 0.991 | -0.19 |  |  |
|  |  | 105068052 | - | 3690111 | 3690263 | 3688155 | 3688318 | 3691549 | 3691759 | 39 | 19 | 132 | 14 | 273 | 135 | 0.001346198 | 0.504 | 0.823 | -0.319 |  |  |
|  |  | 105081173 | - | 2116771 | 2116845 | 2113782 | 2113850 | 2120935 | 2121039 | 19 | 16 | 39 | 4 | 29 | 23 | 0.001401423 | 0.485 | 0.885 | -0.4 |  |  |
|  |  | 105072221 | + | 8457156 | 8457315 | 8452810 | 8452969 | 8459491 | 8460543 | 28 | 8 | 52 | 0 | 280 | 135 | 0.001441133 | 0.628 | 1 | -0.372 |  |  |
|  |  | 105070040 | + | 811179 | 811365 | 803404 | 803536 | 812006 | 812264 | 11 | 13 | 4 | 76 | 297 | 125 | 0.001480256 | 0.263 | 0.022 | 0.241 |  |  |
|  |  | 105071724 | + | 1309657 | 1309828 | 1293562 | 1294238 | 1310480 | 1310515 | 9 | 0 | 6 | 15 | 185 | 28 | 0.00149618 | 1 | 0.057 | 0.943 |  |  |
|  |  | 105081550 | + | 4203358 | 4203502 | 4201965 | 4202055 | 4206073 | 4206214 | 18 | 26 | 72 | 25 | 217 | 82 | 0.001512889 | 0.207 | 0.521 | -0.314 |  |  |
|  |  | 105070199 | - | 445099 | 445153 | 443981 | 444102 | 446051 | 446136 | 1 | 9 | 19 | 6 | 26 | 57 | 0.001517021 | 0.196 | 0.874 | -0.678 |  |  |
|  |  | 105069469 | - | 12297337 | 12297650 | 12282934 | 12283106 | 12299833 | 12299884 | 230 | 20 | 284 | 4 | 343 | 44 | 0.001542143 | 0.596 | 0.901 | -0.305 |  |  |
|  |  | 105067581 | - | 24671576 | 24671717 | 24667519 | 24667654 | 24673530 | 24673632 | 9 | 0 | 11 | 19 | 221 | 88 | 0.001545548 | 1 | 0.187 | 0.813 |  |  |
|  |  | 105067426 | + | 20693243 | 20693354 | 20690248 | 20690364 | 20693996 | 20694120 | 34 | 9 | 120 | 2 | 164 | 91 | 0.001546642 | 0.677 | 0.971 | -0.294 |  |  |
|  |  | 105072951 | - | 14646251 | 14646422 | 14645151 | 14645299 | 14649746 | 14649808 | 0 | 6 | 6 | 0 | 212 | 55 | 0.001614857 | 0 | 1 | -1 |  |  |
|  |  | 105079146 | - | 349908 | 349979 | 345932 | 346065 | 350830 | 350928 | 6 | 6 | 22 | 0 | 75 | 82 | 0.001706462 | 0.522 | 1 | -0.478 |  |  |
|  |  | 105077852 | + | 460712 | 460763 | 458082 | 458268 | 461770 | 461953 | 12 | 2 | 4 | 13 | 88 | 135 | 0.001719248 | 0.902 | 0.321 | 0.581 |  |  |
|  |  | 105073978 | + | 13023185 | 13023371 | 13020790 | 13020966 | 13023864 | 13024074 | 65 | 12 | 101 | 1 | 307 | 135 | 0.001728119 | 0.704 | 0.978 | -0.274 |  |  |
|  |  | 105073874 | - | 1051467 | 1051545 | 1045663 | 1046084 | 1052790 | 1052868 | 66 | 19 | 146 | 6 | 78 | 71 | 0.001729778 | 0.76 | 0.957 | -0.197 |  |  |
|  |  | 105079760 | - | 257993 | 258135 | 238305 | 238361 | 266494 | 266611 | 41 | 9 | 82 | 1 | 159 | 24 | 0.001746439 | 0.407 | 0.925 | -0.518 |  |  |
|  |  | 105063949 | + | 4922111 | 4922300 | 4921398 | 4921602 | 4935539 | 4935727 | 53 | 132 | 72 | 64 | 310 | 135 | 0.001747219 | 0.149 | 0.329 | -0.18 |  |  |
|  |  | 105062400 | + | 8991112 | 8991228 | 8977790 | 8978152 | 8993996 | 8994904 | 3 | 0 | 0 | 25 | 218 | 135 | 0.001799517 | 1 | 0 | 1 |  |  |
|  |  | 105079180 | + | 1496357 | 1496432 | 1481760 | 1482019 | 1504574 | 1504756 | 2 | 22 | 6 | 2 | 136 | 135 | 0.001814514 | 0.083 | 0.749 | -0.666 |  |  |
|  |  | 105074573 | - | 17054 | 17090 | 15348 | 15510 | 18252 | 18375 | 0 | 9 | 16 | 9 | 39 | 116 | 0.001825627 | 0 | 0.841 | -0.841 |  |  |
|  |  | 105064336 | - | 1865062 | 1865195 | 1854659 | 1854847 | 1875689 | 1875799 | 16 | 6 | 139 | 3 | 220 | 103 | 0.001853772 | 0.555 | 0.956 | -0.401 |  |  |
|  |  | 105083908 | + | 3030446 | 3030550 | 3028790 | 3028952 | 3031251 | 3031330 | 79 | 19 | 278 | 14 | 131 | 72 | 0.001919982 | 0.696 | 0.916 | -0.22 |  |  |
|  |  | 105065416 | + | 355614 | 355868 | 343560 | 343609 | 361294 | 361528 | 179 | 24 | 189 | 5 | 282 | 42 | 0.002065068 | 0.526 | 0.849 | -0.323 |  |  |
|  |  | 105079119 | + | 923645 | 923717 | 923313 | 923408 | 925302 | 926728 | 10 | 10 | 1 | 22 | 83 | 88 | 0.002083613 | 0.515 | 0.046 | 0.469 |  |  |
|  |  | 105080218 | - | 1903071 | 1903163 | 1902663 | 1902757 | 1903489 | 1903819 | 2 | 4 | 19 | 0 | 122 | 87 | 0.002106695 | 0.263 | 1 | -0.737 |  |  |
|  |  | 105081995 | + | 12079479 | 12079512 | 12079113 | 12079287 | 12081112 | 12081573 | 13 | 39 | 6 | 115 | 52 | 135 | 0.00212767 | 0.464 | 0.119 | 0.345 |  |  |
|  |  | 105062496 | + | 1153376 | 1153646 | 1143974 | 1144038 | 1155875 | 1156039 | 92 | 0 | 110 | 12 | 313 | 57 | 0.002143344 | 1 | 0.625 | 0.375 |  |  |
|  |  | 105075816 | - | 6621458 | 6621473 | 6617838 | 6618054 | 6623404 | 6623515 | 57 | 245 | 29 | 325 | 8 | 104 | 0.002148921 | 0.752 | 0.537 | 0.215 |  |  |
|  |  | 105081764 | + | 5695830 | 5695985 | 5695344 | 5695501 | 5696661 | 5696768 | 19 | 6 | 50 | 0 | 241 | 100 | 0.002221681 | 0.568 | 1 | -0.432 |  |  |
|  |  | 105064913 | - | 1970927 | 1971090 | 1970449 | 1970721 | 1971218 | 1971258 | 2 | 8 | 8 | 0 | 182 | 33 | 0.002224981 | 0.043 | 1 | -0.957 |  |  |
|  |  | 105082989 | + | 354134 | 354223 | 336484 | 336669 | 367747 | 368637 | 12 | 0 | 12 | 13 | 164 | 135 | 0.002231449 | 1 | 0.432 | 0.568 |  |  |
|  |  | 105077678 | + | 2424924 | 2425077 | 2420885 | 2421109 | 2425988 | 2426141 | 12 | 6 | 75 | 2 | 274 | 135 | 0.002239306 | 0.496 | 0.949 | -0.453 |  |  |
|  |  | 105076601 | + | 23881210 | 23881333 | 23879647 | 23879727 | 23907361 | 23907511 | 0 | 11 | 15 | 12 | 170 | 73 | 0.002311761 | 0 | 0.349 | -0.349 |  |  |
|  |  | 105071932 | - | 12355973 | 12356263 | 12354868 | 12354928 | 12360032 | 12360163 | 103 | 15 | 68 | 35 | 318 | 42 | 0.002363626 | 0.476 | 0.204 | 0.272 |  |  |
|  |  | 105072384 | + | 9314478 | 9314600 | 9314251 | 9314398 | 9317183 | 9318051 | 8 | 0 | 0 | 4 | 230 | 135 | 0.002414249 | 1 | 0 | 1 |  |  |
|  |  | 105081811 | - | 8269625 | 8269788 | 8269328 | 8269478 | 8271641 | 8271861 | 35 | 0 | 49 | 14 | 284 | 135 | 0.002425705 | 1 | 0.625 | 0.375 |  |  |
|  |  | 105063808 | - | 1663939 | 1664019 | 1659842 | 1660530 | 1665223 | 1665609 | 5 | 0 | 0 | 6 | 146 | 135 | 0.002526298 | 1 | 0 | 1 |  |  |
|  |  | 105077508 | + | 488571 | 488637 | 485532 | 487429 | 488820 | 488953 | 51 | 16 | 137 | 5 | 109 | 126 | 0.00257078 | 0.787 | 0.969 | -0.182 |  |  |
|  |  | 105070827 | - | 633809 | 633914 | 628372 | 628505 | 638234 | 638495 | 5 | 21 | 15 | 7 | 187 | 126 | 0.00262101 | 0.138 | 0.591 | -0.453 |  |  |
|  |  | 105065631 | + | 4731535 | 4731604 | 4727570 | 4727738 | 4732610 | 4732756 | 30 | 37 | 47 | 15 | 124 | 135 | 0.002646699 | 0.469 | 0.773 | -0.304 |  |  |
|  |  | 105066268 | + | 17203530 | 17203594 | 17197556 | 17197643 | 17207364 | 17207547 | 2 | 6 | 15 | 1 | 59 | 80 | 0.002787856 | 0.311 | 0.953 | -0.642 |  |  |
|  |  | 105075354 | - | 8283919 | 8284081 | 8280710 | 8280804 | 8287275 | 8287417 | 108 | 15 | 69 | 0 | 235 | 87 | 0.002907275 | 0.727 | 1 | -0.273 |  |  |
|  |  | 105071029 | + | 14780017 | 14780215 | 14778490 | 14778718 | 14780712 | 14780880 | 43 | 16 | 25 | 0 | 319 | 135 | 0.002932384 | 0.532 | 1 | -0.468 |  |  |
|  |  | 105069531 | + | 2019085 | 2019424 | 2018292 | 2018568 | 2022580 | 2022685 | 93 | 11 | 135 | 1 | 423 | 98 | 0.002936085 | 0.662 | 0.969 | -0.307 |  |  |
|  |  | 105066632 | - | 8753210 | 8753331 | 8752080 | 8752145 | 8758969 | 8759079 | 27 | 4 | 162 | 0 | 119 | 26 | 0.003037196 | 0.596 | 1 | -0.404 |  |  |
|  |  | 105079962 | + | 12877024 | 12877091 | 12873398 | 12873529 | 12878127 | 12879859 | 2 | 14 | 10 | 4 | 109 | 124 | 0.003048777 | 0.14 | 0.74 | -0.6 |  |  |
|  |  | 105074848 | - | 6897698 | 6897850 | 6897395 | 6897572 | 6898500 | 6898559 | 1102 | 105 | 1022 | 179 | 190 | 52 | 0.00319303 | 0.742 | 0.61 | 0.132 |  |  |
|  |  | 105063335 | + | 9701127 | 9701196 | 9693353 | 9693439 | 9703903 | 9703967 | 10 | 0 | 6 | 10 | 6 | 1 | 0.003217982 | 1 | 0.091 | 0.909 |  |  |
|  |  | 105063457 | - | 1903797 | 1903932 | 1897373 | 1898212 | 1905626 | 1905708 | 42 | 0 | 41 | 10 | 196 | 75 | 0.003247996 | 1 | 0.611 | 0.389 |  |  |
|  |  | 105081314 | + | 2540886 | 2541004 | 2539013 | 2539172 | 2542536 | 2542679 | 53 | 8 | 161 | 0 | 222 | 135 | 0.003299832 | 0.801 | 1 | -0.199 |  |  |
|  |  | 105070935 | + | 6075347 | 6075554 | 6054228 | 6054315 | 6076442 | 6076631 | 38 | 6 | 89 | 0 | 273 | 80 | 0.003348408 | 0.65 | 1 | -0.35 |  |  |
|  |  | 105077330 | - | 37404560 | 37404629 | 37402710 | 37402853 | 37406798 | 37407132 | 7 | 93 | 35 | 101 | 124 | 135 | 0.003367063 | 0.076 | 0.274 | -0.198 |  |  |
|  |  | 105064043 | + | 7367654 | 7367757 | 7366438 | 7366561 | 7367950 | 7368021 | 0 | 4 | 11 | 1 | 102 | 45 | 0.003401631 | 0 | 0.829 | -0.829 |  |  |
|  |  | 105064040 | + | 7336040 | 7336260 | 7335281 | 7335471 | 7336815 | 7336936 | 109 | 0 | 160 | 15 | 320 | 114 | 0.003432134 | 1 | 0.792 | 0.208 |  |  |
|  |  | 105081536 | - | 1230539 | 1230564 | 1227733 | 1227975 | 1231902 | 1231934 | 10 | 1 | 13 | 23 | 18 | 25 | 0.003512462 | 0.933 | 0.44 | 0.493 |  |  |
|  |  | 105063841 | - | 3750368 | 3750503 | 3750056 | 3750191 | 3752531 | 3752648 | 15 | 103 | 54 | 96 | 224 | 103 | 0.003654626 | 0.063 | 0.205 | -0.142 |  |  |
|  |  | 105080460 | + | 3644660 | 3644841 | 3642161 | 3642325 | 3645573 | 3645698 | 221 | 99 | 241 | 50 | 285 | 118 | 0.003654762 | 0.48 | 0.666 | -0.186 |  |  |
|  |  | 105080238 | - | 2306751 | 2306836 | 2306448 | 2306540 | 2307222 | 2307308 | 29 | 6 | 94 | 0 | 50 | 29 | 0.003659506 | 0.737 | 1 | -0.263 |  |  |
|  |  | 105083155 | + | 4401115 | 4401232 | 4399874 | 4399929 | 4411237 | 4411364 | 6 | 0 | 1 | 7 | 118 | 33 | 0.003726049 | 1 | 0.038 | 0.962 |  |  |
|  |  | 105068918 | - | 4256707 | 4256878 | 4252676 | 4252972 | 4262882 | 4263018 | 20 | 8 | 28 | 0 | 286 | 129 | 0.003780955 | 0.53 | 1 | -0.47 |  |  |
|  |  | 105067832 | - | 2414977 | 2415139 | 2409477 | 2409696 | 2415819 | 2415905 | 1 | 24 | 6 | 3 | 227 | 79 | 0.003806862 | 0.014 | 0.41 | -0.396 |  |  |
|  |  | 105081439 | - | 413268 | 413363 | 367317 | 367390 | 422502 | 422745 | 4 | 6 | 12 | 0 | 107 | 66 | 0.003844365 | 0.291 | 1 | -0.709 |  |  |
|  |  | 105082012 | + | 406276 | 406600 | 394748 | 394814 | 414488 | 414618 | 176 | 17 | 269 | 5 | 357 | 47 | 0.003855921 | 0.577 | 0.876 | -0.299 |  |  |
|  |  | 105070814 | - | 1177608 | 1177826 | 1177354 | 1177533 | 1178122 | 1178376 | 33 | 8 | 45 | 0 | 339 | 135 | 0.003915584 | 0.622 | 1 | -0.378 |  |  |
|  |  | 105064522 | - | 1799271 | 1799319 | 1793509 | 1793616 | 1808493 | 1808620 | 8 | 7 | 7 | 52 | 32 | 85 | 0.003970042 | 0.752 | 0.263 | 0.489 |  |  |
|  |  | 105062440 | + | 8731698 | 8731769 | 8729541 | 8729637 | 8732245 | 8732340 | 2 | 4 | 16 | 0 | 35 | 42 | 0.004000214 | 0.375 | 1 | -0.625 |  |  |
|  |  | 105073346 | - | 2937367 | 2937533 | 2934555 | 2934787 | 2944990 | 2945140 | 0 | 16 | 9 | 10 | 287 | 135 | 0.00410305 | 0 | 0.297 | -0.297 |  |  |
|  |  | 105064733 | + | 241556 | 241632 | 227891 | 228039 | 245629 | 245702 | 26 | 7 | 87 | 0 | 69 | 66 | 0.004125544 | 0.78 | 1 | -0.22 |  |  |
|  |  | 105070927 | - | 5686613 | 5686722 | 5684267 | 5684445 | 5687869 | 5687974 | 218 | 25 | 223 | 3 | 167 | 98 | 0.004155322 | 0.837 | 0.978 | -0.141 |  |  |
|  |  | 105083805 | + | 3585788 | 3585953 | 3576981 | 3577126 | 3592442 | 3592540 | 20 | 0 | 17 | 9 | 242 | 91 | 0.004221331 | 1 | 0.415 | 0.585 |  |  |
|  |  | 105082155 | - | 26616209 | 26616305 | 26613834 | 26613918 | 26616534 | 26616618 | 15 | 6 | 31 | 0 | 62 | 19 | 0.00423305 | 0.434 | 1 | -0.566 |  |  |
|  |  | 105065204 | - | 2692712 | 2692799 | 2691150 | 2691787 | 2696837 | 2696968 | 40 | 8 | 237 | 1 | 149 | 124 | 0.004284893 | 0.806 | 0.995 | -0.189 |  |  |
|  |  | 105074499 | - | 6637619 | 6637734 | 6634851 | 6635048 | 6647377 | 6647519 | 107 | 21 | 91 | 2 | 216 | 135 | 0.004317358 | 0.761 | 0.966 | -0.205 |  |  |
|  |  | 105077288 | + | 31139296 | 31139455 | 31134546 | 31134627 | 31148920 | 31149455 | 48 | 10 | 123 | 3 | 219 | 74 | 0.004356991 | 0.619 | 0.933 | -0.314 |  |  |
|  |  | 105073124 | + | 6585917 | 6586008 | 6575888 | 6576054 | 6586508 | 6586584 | 2 | 5 | 10 | 0 | 102 | 69 | 0.004390379 | 0.213 | 1 | -0.787 |  |  |
|  |  | 105072822 | - | 6333346 | 6333480 | 6332135 | 6332320 | 6333725 | 6333854 | 54 | 0 | 82 | 14 | 241 | 122 | 0.004403926 | 1 | 0.748 | 0.252 |  |  |
|  |  | 105074178 | - | 1156324 | 1156515 | 1155674 | 1156206 | 1157070 | 1157636 | 66 | 15 | 163 | 7 | 312 | 135 | 0.004546836 | 0.656 | 0.91 | -0.254 |  |  |
|  |  | 105080704 | - | 420285 | 420554 | 411977 | 413617 | 424216 | 424908 | 139 | 1 | 69 | 9 | 390 | 135 | 0.004562874 | 0.98 | 0.726 | 0.254 |  |  |
|  |  | 105064745 | - | 234163 | 234420 | 233682 | 233787 | 235382 | 235452 | 0 | 6 | 6 | 0 | 269 | 26 | 0.004700012 | 0 | 1 | -1 |  |  |
|  |  | 105082303 | - | 3042766 | 3042846 | 3041884 | 3042026 | 3047152 | 3047215 | 0 | 33 | 29 | 103 | 73 | 56 | 0.004729043 | 0 | 0.178 | -0.178 |  |  |
|  |  | 105067521 | - | 3139775 | 3139945 | 3135541 | 3135730 | 3143554 | 3144116 | 24 | 8 | 71 | 2 | 291 | 135 | 0.004750961 | 0.582 | 0.943 | -0.361 |  |  |
|  |  | 105083961 | + | 9112395 | 9113249 | 9110012 | 9110207 | 9141638 | 9142010 | 432 | 9 | 602 | 43 | 975 | 135 | 0.00477222 | 0.869 | 0.66 | 0.209 |  |  |
|  |  | 105069945 | + | 4121078 | 4121654 | 4084536 | 4084670 | 4133585 | 4133681 | 16 | 10 | 26 | 1 | 643 | 81 | 0.004791934 | 0.168 | 0.766 | -0.598 |  |  |
|  |  | 105079605 | + | 949886 | 949924 | 947052 | 947413 | 951039 | 951098 | 14 | 135 | 36 | 105 | 31 | 52 | 0.004832179 | 0.148 | 0.365 | -0.217 |  |  |
|  |  | 105065158 | - | 1518145 | 1518240 | 1514405 | 1514473 | 1525089 | 1525233 | 1 | 16 | 7 | 4 | 102 | 61 | 0.004851801 | 0.036 | 0.511 | -0.475 |  |  |
|  |  | 105064078 | + | 13636342 | 13636375 | 13632742 | 13632901 | 13637916 | 13637976 | 8 | 9 | 4 | 42 | 26 | 53 | 0.004853092 | 0.644 | 0.163 | 0.481 |  |  |
|  |  | 105072931 | + | 10232046 | 10232319 | 10230601 | 10230793 | 10233441 | 10233624 | 102 | 142 | 438 | 312 | 394 | 135 | 0.00490963 | 0.198 | 0.325 | -0.127 |  |  |
|  |  | 105073927 | - | 5088952 | 5089148 | 5081933 | 5082148 | 5097896 | 5098117 | 122 | 14 | 138 | 1 | 317 | 135 | 0.004924108 | 0.788 | 0.983 | -0.195 |  |  |
|  |  | 105082913 | - | 6726661 | 6726794 | 6721252 | 6721594 | 6730371 | 6730829 | 34 | 7 | 113 | 1 | 252 | 135 | 0.004947685 | 0.722 | 0.984 | -0.262 |  |  |
|  |  | 105061950 | - | 951193 | 951261 | 948833 | 948990 | 951661 | 951722 | 5 | 0 | 7 | 21 | 61 | 54 | 0.005014366 | 1 | 0.228 | 0.772 |  |  |
|  |  | 105077805 | + | 5945722 | 5945829 | 5933733 | 5933841 | 5953545 | 5953659 | 44 | 1 | 98 | 26 | 138 | 73 | 0.00509796 | 0.959 | 0.666 | 0.293 |  |  |
|  |  | 105077021 | - | 14178290 | 14178344 | 14177733 | 14177836 | 14178540 | 14178597 | 24 | 2 | 28 | 21 | 8 | 11 | 0.005287598 | 0.943 | 0.647 | 0.296 |  |  |
|  |  | 105077681 | - | 2715032 | 2715155 | 2714392 | 2714524 | 2716340 | 2716504 | 2 | 5 | 31 | 4 | 222 | 125 | 0.005381175 | 0.184 | 0.814 | -0.63 |  |  |
|  |  | 105076212 | - | 1452232 | 1452364 | 1426896 | 1427057 | 1466031 | 1466167 | 85 | 14 | 54 | 0 | 244 | 129 | 0.005476121 | 0.762 | 1 | -0.238 |  |  |
|  |  | 105063788 | + | 218663 | 218860 | 209594 | 209730 | 247567 | 247707 | 10 | 12 | 14 | 1 | 310 | 127 | 0.005716507 | 0.255 | 0.852 | -0.597 |  |  |
|  |  | 105076557 | - | 3510404 | 3510479 | 3509316 | 3509364 | 3512604 | 3514920 | 0 | 7 | 5 | 1 | 68 | 41 | 0.005835743 | 0 | 0.751 | -0.751 |  |  |
|  |  | 105075539 | + | 14166748 | 14166822 | 14165537 | 14165693 | 14167367 | 14169179 | 114 | 0 | 236 | 30 | 134 | 135 | 0.005853293 | 1 | 0.888 | 0.112 |  |  |
|  |  | 105067430 | + | 20889130 | 20889286 | 20881995 | 20882175 | 20891837 | 20892435 | 58 | 9 | 53 | 31 | 277 | 135 | 0.00597321 | 0.759 | 0.455 | 0.304 |  |  |
|  |  | 105082155 | - | 26680717 | 26680909 | 26680073 | 26680286 | 26682137 | 26682304 | 79 | 20 | 195 | 13 | 313 | 135 | 0.006000472 | 0.63 | 0.866 | -0.236 |  |  |
|  |  | 105063093 | + | 9930052 | 9930192 | 9926204 | 9926312 | 9932217 | 9932409 | 56 | 12 | 127 | 4 | 232 | 101 | 0.006110555 | 0.67 | 0.933 | -0.263 |  |  |
|  |  | 105083560 | + | 2106449 | 2106551 | 2103248 | 2103807 | 2109212 | 2109406 | 0 | 2 | 31 | 0 | 190 | 135 | 0.006240019 | 0 | 1 | -1 |  |  |
|  |  | 105076289 | - | 6873306 | 6873488 | 6871809 | 6872290 | 6880192 | 6880322 | 12 | 0 | 2 | 4 | 291 | 123 | 0.00628947 | 1 | 0.174 | 0.826 |  |  |
|  |  | 105072401 | - | 9982199 | 9982244 | 9979223 | 9979339 | 9983622 | 9984156 | 7 | 4 | 2 | 18 | 50 | 109 | 0.006321615 | 0.792 | 0.195 | 0.597 |  |  |
|  |  | 105078749 | - | 741566 | 741784 | 740640 | 741480 | 742064 | 742183 | 244 | 20 | 438 | 6 | 316 | 112 | 0.006386381 | 0.812 | 0.963 | -0.151 |  |  |
|  |  | 105073519 | + | 2045071 | 2045156 | 2039570 | 2039765 | 2068206 | 2068335 | 21 | 30 | 53 | 22 | 143 | 122 | 0.006515649 | 0.374 | 0.673 | -0.299 |  |  |
|  |  | 105081766 | - | 5866584 | 5866706 | 5842353 | 5844167 | 5884743 | 5884843 | 23 | 0 | 19 | 8 | 188 | 93 | 0.006527989 | 1 | 0.54 | 0.46 |  |  |
|  |  | 105063015 | - | 619893 | 619933 | 617015 | 617095 | 638710 | 638835 | 1 | 4 | 17 | 0 | 16 | 56 | 0.006562901 | 0.467 | 1 | -0.533 |  |  |
|  |  | 105071932 | - | 12355973 | 12356218 | 12354868 | 12354928 | 12360032 | 12360163 | 62 | 15 | 41 | 35 | 273 | 42 | 0.006643456 | 0.389 | 0.153 | 0.236 |  |  |
|  |  | 105081023 | + | 3688645 | 3688828 | 3687632 | 3687785 | 3715147 | 3715342 | 46 | 6 | 132 | 0 | 304 | 135 | 0.006741086 | 0.773 | 1 | -0.227 |  |  |
|  |  | 105073667 | - | 2486232 | 2486334 | 2483619 | 2485377 | 2486623 | 2486986 | 8 | 21 | 34 | 19 | 190 | 135 | 0.006792652 | 0.213 | 0.56 | -0.347 |  |  |
|  |  | 105083574 | - | 18984987 | 18985140 | 18982774 | 18983034 | 18986746 | 18986922 | 16 | 45 | 26 | 18 | 274 | 135 | 0.006999182 | 0.149 | 0.416 | -0.267 |  |  |
|  |  | 105067032 | - | 978336 | 978451 | 967783 | 967953 | 987794 | 987957 | 51 | 0 | 60 | 11 | 216 | 135 | 0.007135465 | 1 | 0.773 | 0.227 |  |  |
|  |  | 105068146 | + | 1119729 | 1119779 | 1116055 | 1117747 | 1125876 | 1126417 | 17 | 0 | 7 | 6 | 86 | 135 | 0.007144032 | 1 | 0.647 | 0.353 |  |  |
|  |  | 105063593 | + | 175716 | 175992 | 175058 | 175256 | 176964 | 177087 | 26 | 15 | 3 | 14 | 378 | 116 | 0.007149722 | 0.347 | 0.062 | 0.285 |  |  |
|  |  | 105071388 | + | 2204139 | 2204313 | 2200857 | 2200970 | 2205670 | 2205719 | 92 | 5 | 199 | 0 | 173 | 13 | 0.00715264 | 0.58 | 1 | -0.42 |  |  |
|  |  | 105070994 | + | 10294877 | 10294935 | 10290701 | 10290786 | 10295547 | 10295666 | 12 | 0 | 3 | 5 | 28 | 55 | 0.007215221 | 1 | 0.541 | 0.459 |  |  |
|  |  | 105065472 | + | 637470 | 637622 | 636257 | 636419 | 640415 | 640943 | 199 | 0 | 272 | 19 | 273 | 135 | 0.007268783 | 1 | 0.876 | 0.124 |  |  |
|  |  | 105077135 | - | 7747004 | 7747140 | 7744458 | 7744574 | 7747906 | 7747966 | 22 | 0 | 9 | 5 | 150 | 27 | 0.007300136 | 1 | 0.245 | 0.755 |  |  |
|  |  | 105082732 | - | 856842 | 856935 | 854710 | 855105 | 857312 | 857448 | 1 | 38 | 6 | 9 | 166 | 129 | 0.007317257 | 0.02 | 0.341 | -0.321 |  |  |
|  |  | 105064139 | - | 3181167 | 3181304 | 3159119 | 3167967 | 3187428 | 3187631 | 20 | 6 | 37 | 0 | 260 | 135 | 0.007346786 | 0.634 | 1 | -0.366 |  |  |
|  |  | 105062366 | - | 1008176 | 1008311 | 1001241 | 1001364 | 1009972 | 1010078 | 14 | 7 | 101 | 7 | 201 | 80 | 0.007699216 | 0.443 | 0.852 | -0.409 |  |  |
|  |  | 105067859 | + | 5161498 | 5161684 | 5161194 | 5161334 | 5162825 | 5162921 | 0 | 11 | 3 | 0 | 259 | 87 | 0.007713302 | 0 | 1 | -1 |  |  |
|  |  | 105065998 | - | 1033369 | 1033558 | 1032225 | 1032323 | 1033885 | 1034001 | 86 | 23 | 83 | 5 | 240 | 65 | 0.007736687 | 0.503 | 0.818 | -0.315 |  |  |
|  |  | 105077324 | + | 36461657 | 36461831 | 36457306 | 36457395 | 36469110 | 36469247 | 14 | 5 | 32 | 0 | 237 | 77 | 0.0077706 | 0.476 | 1 | -0.524 |  |  |
|  |  | 105069444 | + | 3137706 | 3137799 | 3133124 | 3133958 | 3143127 | 3143238 | 12 | 8 | 15 | 0 | 141 | 104 | 0.007836304 | 0.525 | 1 | -0.475 |  |  |
|  |  | 105062430 | - | 601822 | 601873 | 595961 | 596095 | 623773 | 623889 | 4 | 0 | 5 | 19 | 54 | 101 | 0.007838365 | 1 | 0.33 | 0.67 |  |  |
|  |  | 105075577 | + | 14782478 | 14782556 | 14780355 | 14780466 | 14784978 | 14785164 | 14 | 6 | 154 | 5 | 111 | 104 | 0.007905312 | 0.686 | 0.967 | -0.281 |  |  |
|  |  | 105076064 | + | 176778 | 176802 | 176433 | 176623 | 179127 | 179375 | 318 | 634 | 203 | 661 | 34 | 135 | 0.007983648 | 0.666 | 0.549 | 0.117 |  |  |
|  |  | 105080473 | + | 5442300 | 5442440 | 5442134 | 5442200 | 5449280 | 5449398 | 19 | 6 | 90 | 3 | 166 | 35 | 0.008197128 | 0.4 | 0.863 | -0.463 |  |  |
|  |  | 105063278 | - | 6553369 | 6553580 | 6544012 | 6547403 | 6555470 | 6555656 | 245 | 24 | 464 | 10 | 332 | 135 | 0.008217672 | 0.806 | 0.95 | -0.144 |  |  |
|  |  | 105082117 | - | 22869233 | 22869368 | 22865665 | 22865784 | 22869759 | 22869921 | 0 | 4 | 5 | 0 | 233 | 112 | 0.008233598 | 0 | 1 | -1 |  |  |
|  |  | 105077133 | - | 7309625 | 7309714 | 7305895 | 7305964 | 7311823 | 7312002 | 10 | 7 | 15 | 0 | 91 | 62 | 0.008239459 | 0.493 | 1 | -0.507 |  |  |
|  |  | 105082302 | - | 3012366 | 3012535 | 2997683 | 2998092 | 3012932 | 3012993 | 31 | 6 | 34 | 30 | 209 | 54 | 0.008291573 | 0.572 | 0.226 | 0.346 |  |  |
|  |  | 105063457 | - | 1899552 | 1900674 | 1897373 | 1898212 | 1905626 | 1905708 | 121 | 0 | 171 | 10 | 1183 | 75 | 0.008341945 | 1 | 0.52 | 0.48 |  |  |
|  |  | 105081613 | - | 11903112 | 11903275 | 11901031 | 11901161 | 11903508 | 11903620 | 2 | 7 | 37 | 12 | 242 | 93 | 0.008402459 | 0.099 | 0.542 | -0.443 |  |  |
|  |  | 105083627 | - | 1831608 | 1831731 | 1816687 | 1816778 | 1900569 | 1900717 | 8 | 3 | 0 | 6 | 181 | 84 | 0.008490062 | 0.553 | 0 | 0.553 |  |  |
|  |  | 105071764 | + | 1600919 | 1601041 | 1600429 | 1600520 | 1601274 | 1601335 | 60 | 52 | 46 | 5 | 98 | 3 | 0.008548679 | 0.034 | 0.22 | -0.186 |  |  |
|  |  | 105072963 | - | 15928466 | 15928508 | 15921842 | 15921943 | 15929232 | 15929379 | 4 | 1 | 5 | 32 | 35 | 94 | 0.00861499 | 0.915 | 0.296 | 0.619 |  |  |
|  |  | 105080793 | + | 11080105 | 11080246 | 11078705 | 11078877 | 11087564 | 11087708 | 26 | 75 | 2 | 60 | 268 | 135 | 0.008670039 | 0.149 | 0.017 | 0.132 |  |  |
|  |  | 105068705 | + | 7392753 | 7392853 | 7388442 | 7388568 | 7399447 | 7399492 | 0 | 3 | 7 | 0 | 77 | 22 | 0.008692284 | 0 | 1 | -1 |  |  |
|  |  | 105071166 | + | 10376175 | 10376205 | 10364855 | 10364978 | 10377366 | 10377562 | 0 | 64 | 5 | 30 | 27 | 116 | 0.008697606 | 0 | 0.417 | -0.417 |  |  |
|  |  | 105069232 | - | 11429675 | 11429772 | 11427784 | 11427926 | 11451901 | 11452027 | 1 | 6 | 5 | 0 | 164 | 119 | 0.008801423 | 0.108 | 1 | -0.892 |  |  |
|  |  | 105078911 | + | 337274 | 337352 | 285138 | 285370 | 339379 | 339466 | 14 | 13 | 79 | 16 | 87 | 80 | 0.008903942 | 0.498 | 0.82 | -0.322 |  |  |
|  |  | 105069084 | - | 1341850 | 1341964 | 1339337 | 1339436 | 1342848 | 1342967 | 27 | 6 | 47 | 0 | 148 | 69 | 0.00907811 | 0.677 | 1 | -0.323 |  |  |
|  |  | 105072322 | - | 1717772 | 1717934 | 1717366 | 1717475 | 1718021 | 1718180 | 118 | 17 | 49 | 0 | 250 | 102 | 0.009083568 | 0.739 | 1 | -0.261 |  |  |
|  |  | 105065318 | + | 2874573 | 2874716 | 2871369 | 2871537 | 2878934 | 2879148 | 18 | 4 | 90 | 0 | 270 | 135 | 0.009124295 | 0.692 | 1 | -0.308 |  |  |
|  |  | 105063809 | - | 2585729 | 2585825 | 2585111 | 2585183 | 2586812 | 2586917 | 3 | 3 | 22 | 0 | 71 | 28 | 0.009156896 | 0.283 | 1 | -0.717 |  |  |
|  |  | 105061921 | - | 4648151 | 4648211 | 4635158 | 4635305 | 4650357 | 4650548 | 6 | 0 | 10 | 17 | 106 | 135 | 0.009197422 | 1 | 0.428 | 0.572 |  |  |
|  |  | 105080144 | - | 852227 | 852320 | 851395 | 851518 | 855182 | 855287 | 15 | 7 | 19 | 44 | 116 | 79 | 0.009315693 | 0.593 | 0.227 | 0.366 |  |  |
|  |  | 105062423 | + | 12102698 | 12102800 | 12097333 | 12097487 | 12104233 | 12104308 | 50 | 0 | 50 | 9 | 123 | 68 | 0.009376874 | 1 | 0.754 | 0.246 |  |  |
|  |  | 105064294 | + | 490327 | 490429 | 489055 | 489163 | 491360 | 491462 | 106 | 5 | 210 | 42 | 116 | 61 | 0.009403707 | 0.918 | 0.724 | 0.194 |  |  |
|  |  | 105083219 | + | 739131 | 739359 | 725940 | 726063 | 747523 | 747738 | 8 | 7 | 12 | 0 | 330 | 116 | 0.009425847 | 0.287 | 1 | -0.713 |  |  |
|  |  | 105081656 | - | 4418958 | 4419053 | 4415638 | 4415758 | 4422246 | 4422372 | 1 | 3 | 12 | 0 | 138 | 97 | 0.009533874 | 0.19 | 1 | -0.81 |  |  |
|  |  | 105083048 | + | 4868680 | 4868882 | 4867931 | 4867992 | 4868978 | 4869105 | 227 | 8 | 428 | 0 | 227 | 39 | 0.009558689 | 0.83 | 1 | -0.17 |  |  |
|  |  | 105072740 | + | 755698 | 755861 | 754346 | 754405 | 763308 | 764869 | 13 | 0 | 13 | 9 | 201 | 52 | 0.009667423 | 1 | 0.272 | 0.728 |  |  |
|  |  | 105067444 | - | 21002882 | 21002927 | 21002643 | 21002743 | 21003606 | 21003746 | 4 | 20 | 11 | 7 | 36 | 91 | 0.009734894 | 0.336 | 0.799 | -0.463 |  |  |
|  |  | 105066588 | - | 4262701 | 4262854 | 4261304 | 4261430 | 4263138 | 4263240 | 38 | 5 | 348 | 2 | 218 | 79 | 0.009764427 | 0.734 | 0.984 | -0.25 |  |  |
|  |  | 105083310 | - | 2082286 | 2082467 | 2078537 | 2079711 | 2082940 | 2083042 | 243 | 146 | 432 | 147 | 262 | 95 | 0.009921999 | 0.376 | 0.516 | -0.14 |  |  |
|  |  | 105078453 | - | 5233907 | 5234242 | 5233660 | 5233779 | 5234425 | 5234484 | 76 | 0 | 199 | 16 | 350 | 29 | 0.010074873 | 1 | 0.508 | 0.492 |  |  |
|  |  | 105066927 | - | 365182 | 365298 | 363670 | 363960 | 385553 | 385775 | 67 | 40 | 173 | 42 | 218 | 135 | 0.010247737 | 0.509 | 0.718 | -0.209 |  |  |
|  |  | 105063558 | + | 3043268 | 3043871 | 3035404 | 3035524 | 3047153 | 3047738 | 72 | 33 | 80 | 103 | 702 | 113 | 0.010270331 | 0.26 | 0.111 | 0.149 |  |  |
|  |  | 105069345 | - | 1649586 | 1649718 | 1647328 | 1647445 | 1650121 | 1650263 | 37 | 10 | 82 | 3 | 225 | 110 | 0.010332375 | 0.644 | 0.93 | -0.286 |  |  |
|  |  | 105069073 | + | 1119012 | 1119067 | 1114265 | 1114634 | 1119894 | 1121241 | 15 | 22 | 8 | 54 | 96 | 135 | 0.010437385 | 0.489 | 0.172 | 0.317 |  |  |
|  |  | 105064019 | - | 6856233 | 6856398 | 6854568 | 6855393 | 6857453 | 6858053 | 36 | 18 | 14 | 0 | 286 | 135 | 0.010455707 | 0.486 | 1 | -0.514 |  |  |
|  |  | 105081612 | - | 11839307 | 11839430 | 11838058 | 11838172 | 11841306 | 11841432 | 42 | 16 | 55 | 62 | 188 | 91 | 0.01065967 | 0.56 | 0.3 | 0.26 |  |  |
|  |  | 105079851 | - | 1445286 | 1445349 | 1436042 | 1436148 | 1530127 | 1530192 | 5 | 6 | 0 | 15 | 20 | 22 | 0.010805768 | 0.478 | 0 | 0.478 |  |  |
|  |  | 105073586 | - | 5000592 | 5000694 | 4998430 | 4998657 | 5001496 | 5001661 | 8 | 9 | 23 | 3 | 190 | 135 | 0.011247778 | 0.387 | 0.845 | -0.458 |  |  |
|  |  | 105073650 | - | 3309747 | 3309919 | 3309265 | 3309378 | 3310098 | 3310218 | 46 | 6 | 77 | 0 | 242 | 84 | 0.011259089 | 0.727 | 1 | -0.273 |  |  |
|  |  | 105067339 | + | 12127217 | 12127425 | 12121866 | 12122418 | 12133448 | 12133582 | 2 | 6 | 6 | 0 | 321 | 127 | 0.011581964 | 0.117 | 1 | -0.883 |  |  |
|  |  | 105063577 | - | 6036243 | 6036300 | 6035261 | 6035364 | 6036940 | 6037027 | 0 | 22 | 5 | 11 | 11 | 41 | 0.011592112 | 0 | 0.629 | -0.629 |  |  |
|  |  | 105081379 | + | 11640102 | 11640159 | 11631043 | 11631255 | 11642597 | 11642677 | 33 | 7 | 46 | 39 | 50 | 73 | 0.011653061 | 0.873 | 0.633 | 0.24 |  |  |
|  |  | 105061972 | - | 987128 | 987200 | 984663 | 984762 | 990106 | 990217 | 0 | 2 | 19 | 0 | 56 | 61 | 0.011685097 | 0 | 1 | -1 |  |  |
|  |  | 105063093 | + | 9928047 | 9928139 | 9926204 | 9926312 | 9932217 | 9932409 | 5 | 12 | 14 | 4 | 136 | 101 | 0.011720999 | 0.236 | 0.722 | -0.486 |  |  |
|  |  | 105071096 | + | 16893055 | 16893280 | 16888242 | 16888576 | 16893963 | 16894146 | 131 | 12 | 89 | 0 | 346 | 135 | 0.011921047 | 0.81 | 1 | -0.19 |  |  |
|  |  | 105078688 | - | 3821745 | 3821861 | 3819063 | 3819187 | 3825033 | 3825260 | 0 | 11 | 7 | 7 | 200 | 117 | 0.011985919 | 0 | 0.369 | -0.369 |  |  |
|  |  | 105067488 | + | 672105 | 672244 | 670801 | 670886 | 672474 | 672658 | 0 | 3 | 6 | 0 | 207 | 78 | 0.011996099 | 0 | 1 | -1 |  |  |
|  |  | 105075937 | + | 556067 | 556267 | 534881 | 535034 | 557514 | 557725 | 14 | 0 | 4 | 4 | 321 | 135 | 0.012145921 | 1 | 0.296 | 0.704 |  |  |
|  |  | 105067792 | + | 1158130 | 1158286 | 1156914 | 1156999 | 1158369 | 1158898 | 26 | 6 | 68 | 1 | 220 | 78 | 0.012227894 | 0.606 | 0.96 | -0.354 |  |  |
|  |  | 105075812 | - | 4865528 | 4867567 | 4859962 | 4860788 | 4867819 | 4867932 | 421 | 14 | 1225 | 11 | 2131 | 106 | 0.012375178 | 0.599 | 0.847 | -0.248 |  |  |
|  |  | 105080823 | + | 868467 | 868538 | 868148 | 868209 | 868801 | 871778 | 18 | 11 | 21 | 1 | 64 | 54 | 0.012442726 | 0.58 | 0.947 | -0.367 |  |  |
|  |  | 105077113 | - | 5849857 | 5850025 | 5849290 | 5849374 | 5852525 | 5853497 | 54 | 17 | 99 | 8 | 231 | 77 | 0.012454342 | 0.514 | 0.805 | -0.291 |  |  |
|  |  | 105083771 | + | 2364597 | 2364705 | 2363943 | 2364093 | 2365364 | 2365502 | 12 | 0 | 7 | 6 | 198 | 131 | 0.012475583 | 1 | 0.436 | 0.564 |  |  |
|  |  | 105070050 | + | 6112 | 6303 | 2513 | 2667 | 7520 | 7585 | 169 | 8 | 530 | 0 | 235 | 58 | 0.012495145 | 0.839 | 1 | -0.161 |  |  |
|  |  | 105068276 | - | 1004412 | 1004488 | 998131 | 999238 | 1028262 | 1028470 | 2 | 12 | 12 | 7 | 138 | 135 | 0.012549161 | 0.14 | 0.626 | -0.486 |  |  |
|  |  | 105071315 | + | 13374194 | 13374372 | 13373972 | 13374115 | 13374462 | 13374823 | 38 | 6 | 64 | 0 | 299 | 135 | 0.012561703 | 0.741 | 1 | -0.259 |  |  |
|  |  | 105073487 | - | 334778 | 334908 | 322985 | 323098 | 339818 | 340028 | 4 | 3 | 24 | 0 | 217 | 106 | 0.012580177 | 0.394 | 1 | -0.606 |  |  |
|  |  | 105073632 | - | 3253324 | 3253352 | 3248828 | 3248980 | 3255288 | 3255361 | 17 | 43 | 8 | 79 | 21 | 66 | 0.012677167 | 0.554 | 0.241 | 0.313 |  |  |
|  |  | 105065263 | + | 1581236 | 1581341 | 1580767 | 1580860 | 1582014 | 1583627 | 118 | 963 | 214 | 739 | 147 | 86 | 0.012754771 | 0.067 | 0.145 | -0.078 |  |  |
|  |  | 105080101 | - | 369885 | 370057 | 369313 | 369420 | 370311 | 370461 | 0 | 12 | 4 | 2 | 258 | 100 | 0.012839312 | 0 | 0.437 | -0.437 |  |  |
|  |  | 105081826 | + | 9294725 | 9294764 | 9292558 | 9292641 | 9296867 | 9296992 | 4 | 0 | 7 | 20 | 15 | 59 | 0.0129455 | 1 | 0.579 | 0.421 |  |  |
|  |  | 105062139 | - | 796917 | 796977 | 796536 | 796685 | 797880 | 798009 | 11 | 24 | 1 | 27 | 93 | 122 | 0.01299827 | 0.375 | 0.046 | 0.329 |  |  |
|  |  | 105063192 | + | 554594 | 554748 | 545591 | 545750 | 560072 | 560211 | 129 | 0 | 128 | 11 | 272 | 132 | 0.013099127 | 1 | 0.85 | 0.15 |  |  |
|  |  | 105081379 | + | 11606481 | 11606646 | 11588942 | 11589034 | 11609320 | 11609382 | 89 | 4 | 205 | 0 | 156 | 5 | 0.013294941 | 0.416 | 1 | -0.584 |  |  |
|  |  | 105063968 | + | 142602 | 142800 | 141685 | 141776 | 143762 | 143898 | 33 | 12 | 175 | 17 | 262 | 78 | 0.013457841 | 0.45 | 0.754 | -0.304 |  |  |
|  |  | 105083924 | - | 9088296 | 9088516 | 9081022 | 9081173 | 9093927 | 9094071 | 8 | 4 | 23 | 0 | 341 | 135 | 0.013503489 | 0.442 | 1 | -0.558 |  |  |
|  |  | 105080105 | + | 429795 | 429839 | 427392 | 427443 | 437176 | 437283 | 0 | 72 | 11 | 120 | 2 | 9 | 0.013651238 | 0 | 0.292 | -0.292 |  |  |
|  |  | 105074560 | + | 226280 | 226679 | 224735 | 225239 | 227251 | 227803 | 77 | 5 | 9 | 5 | 520 | 135 | 0.013837753 | 0.8 | 0.318 | 0.482 |  |  |
|  |  | 105075030 | - | 5137487 | 5137681 | 5134633 | 5136135 | 5141337 | 5141479 | 60 | 6 | 213 | 0 | 315 | 135 | 0.013962017 | 0.811 | 1 | -0.189 |  |  |
|  |  | 105072703 | + | 9593513 | 9593609 | 9586986 | 9587118 | 9596445 | 9596548 | 0 | 4 | 4 | 0 | 129 | 86 | 0.014071006 | 0 | 1 | -1 |  |  |
|  |  | 105064679 | + | 973848 | 974028 | 973578 | 973735 | 974513 | 974606 | 234 | 24 | 132 | 2 | 252 | 86 | 0.014127547 | 0.769 | 0.957 | -0.188 |  |  |
|  |  | 105069902 | - | 1063170 | 1063283 | 1062315 | 1062405 | 1064528 | 1064694 | 8 | 1 | 13 | 22 | 160 | 83 | 0.014195182 | 0.806 | 0.235 | 0.571 |  |  |
|  |  | 105078799 | - | 3751031 | 3751099 | 3750587 | 3750651 | 3753237 | 3753563 | 44 | 0 | 16 | 5 | 61 | 57 | 0.014219176 | 1 | 0.749 | 0.251 |  |  |
|  |  | 105073449 | - | 2067395 | 2067608 | 2065922 | 2066122 | 2069485 | 2069523 | 10 | 4 | 26 | 0 | 230 | 31 | 0.014304824 | 0.252 | 1 | -0.748 |  |  |
|  |  | 105063382 | - | 1008771 | 1008823 | 1006273 | 1006354 | 1011594 | 1011742 | 7 | 5 | 40 | 1 | 45 | 74 | 0.014356503 | 0.697 | 0.985 | -0.288 |  |  |
|  |  | 105065987 | + | 843057 | 843292 | 841824 | 842074 | 844629 | 844720 | 10 | 14 | 0 | 42 | 305 | 84 | 0.014513757 | 0.164 | 0 | 0.164 |  |  |
|  |  | 105064081 | - | 542706 | 542796 | 542257 | 542439 | 542949 | 543008 | 10 | 6 | 15 | 0 | 83 | 52 | 0.014740878 | 0.511 | 1 | -0.489 |  |  |
|  |  | 105072064 | - | 7800962 | 7801075 | 7798690 | 7798776 | 7807468 | 7807714 | 249 | 30 | 618 | 22 | 156 | 79 | 0.014804949 | 0.808 | 0.934 | -0.126 |  |  |
|  |  | 105062728 | - | 3415517 | 3415630 | 3414126 | 3414253 | 3417006 | 3417219 | 10 | 4 | 30 | 0 | 197 | 120 | 0.014845354 | 0.604 | 1 | -0.396 |  |  |
|  |  | 105071082 | - | 16711386 | 16711711 | 16710500 | 16710799 | 16713216 | 16713353 | 4 | 7 | 18 | 3 | 441 | 130 | 0.014852191 | 0.144 | 0.639 | -0.495 |  |  |
|  |  | 105063926 | + | 2493541 | 2493606 | 2491038 | 2491199 | 2498431 | 2498533 | 25 | 0 | 58 | 17 | 76 | 95 | 0.015004177 | 1 | 0.81 | 0.19 |  |  |
|  |  | 105072132 | - | 2816805 | 2816904 | 2815116 | 2816189 | 2820529 | 2820595 | 11 | 0 | 5 | 5 | 108 | 59 | 0.015106482 | 1 | 0.353 | 0.647 |  |  |
|  |  | 105077677 | + | 2360381 | 2360426 | 2355949 | 2356055 | 2361222 | 2361386 | 23 | 33 | 89 | 43 | 40 | 99 | 0.015148154 | 0.633 | 0.837 | -0.204 |  |  |
|  |  | 105061721 | - | 204608 | 204710 | 204197 | 204330 | 212784 | 212877 | 14 | 33 | 3 | 58 | 132 | 77 | 0.015271183 | 0.198 | 0.029 | 0.169 |  |  |
|  |  | 105063593 | + | 176964 | 177087 | 175058 | 175256 | 177899 | 178195 | 22 | 20 | 14 | 48 | 232 | 135 | 0.015283195 | 0.39 | 0.145 | 0.245 |  |  |
|  |  | 105064853 | - | 2599552 | 2599621 | 2598023 | 2598154 | 2600303 | 2600507 | 0 | 38 | 4 | 10 | 113 | 124 | 0.015381351 | 0 | 0.305 | -0.305 |  |  |
|  |  | 105067855 | - | 4704432 | 4704595 | 4701767 | 4701858 | 4705514 | 4705704 | 27 | 15 | 116 | 19 | 233 | 84 | 0.015393911 | 0.394 | 0.688 | -0.294 |  |  |
|  |  | 105080598 | - | 4583770 | 4583875 | 4581812 | 4581912 | 4584915 | 4585031 | 56 | 7 | 86 | 0 | 128 | 67 | 0.015422355 | 0.807 | 1 | -0.193 |  |  |
|  |  | 105066587 | - | 4226280 | 4226400 | 4224570 | 4224673 | 4229083 | 4229145 | 16 | 4 | 38 | 0 | 107 | 16 | 0.015440534 | 0.374 | 1 | -0.626 |  |  |
|  |  | 105078648 | - | 2053152 | 2053409 | 2050795 | 2050922 | 2060811 | 2060951 | 64 | 0 | 104 | 11 | 361 | 118 | 0.015467741 | 1 | 0.756 | 0.244 |  |  |
|  |  | 105082741 | + | 1470957 | 1471020 | 1469108 | 1469165 | 1478358 | 1478469 | 27 | 1 | 19 | 9 | 25 | 19 | 0.015486487 | 0.954 | 0.616 | 0.338 |  |  |
|  |  | 105075326 | + | 2223360 | 2223759 | 2220117 | 2220245 | 2231267 | 2231455 | 41 | 0 | 57 | 9 | 506 | 121 | 0.015498219 | 1 | 0.602 | 0.398 |  |  |
|  |  | 105067859 | + | 5162825 | 5162921 | 5161194 | 5161334 | 5163573 | 5163752 | 11 | 42 | 0 | 37 | 176 | 133 | 0.015582333 | 0.165 | 0 | 0.165 |  |  |
|  |  | 105063408 | - | 347781 | 347844 | 343264 | 343441 | 352375 | 352617 | 19 | 176 | 40 | 130 | 112 | 135 | 0.015794683 | 0.115 | 0.271 | -0.156 |  |  |
|  |  | 105066457 | - | 2493050 | 2493210 | 2488038 | 2488151 | 2496852 | 2496957 | 52 | 6 | 219 | 2 | 215 | 69 | 0.015985642 | 0.736 | 0.972 | -0.236 |  |  |
|  |  | 105067292 | - | 6605072 | 6605175 | 6604291 | 6604423 | 6610668 | 6610842 | 43 | 0 | 41 | 8 | 182 | 125 | 0.016285749 | 1 | 0.779 | 0.221 |  |  |
|  |  | 105062531 | - | 1969937 | 1970012 | 1968330 | 1969584 | 1970877 | 1971082 | 14 | 16 | 2 | 18 | 136 | 135 | 0.016352282 | 0.465 | 0.099 | 0.366 |  |  |
|  |  | 105074379 | + | 1707981 | 1708089 | 1707794 | 1707870 | 1708176 | 1708353 | 7 | 7 | 9 | 0 | 136 | 69 | 0.016839461 | 0.337 | 1 | -0.663 |  |  |
|  |  | 105078884 | - | 7965257 | 7965473 | 7961652 | 7962263 | 7966286 | 7966572 | 90 | 9 | 285 | 3 | 337 | 135 | 0.016851864 | 0.8 | 0.974 | -0.174 |  |  |
|  |  | 105072884 | - | 924694 | 924825 | 920475 | 920685 | 930901 | 931053 | 2 | 2 | 44 | 0 | 248 | 135 | 0.016893102 | 0.352 | 1 | -0.648 |  |  |
|  |  | 105066567 | + | 9614599 | 9614707 | 9607198 | 9607346 | 9617792 | 9617917 | 7 | 1 | 19 | 34 | 185 | 118 | 0.016929407 | 0.817 | 0.263 | 0.554 |  |  |
|  |  | 105082033 | - | 7412315 | 7412393 | 7411910 | 7412027 | 7412973 | 7413113 | 67 | 0 | 94 | 14 | 115 | 108 | 0.016998474 | 1 | 0.863 | 0.137 |  |  |
|  |  | 105078953 | + | 1016715 | 1016841 | 993027 | 993153 | 1020228 | 1020372 | 63 | 5 | 59 | 20 | 222 | 119 | 0.017286832 | 0.871 | 0.613 | 0.258 |  |  |
|  |  | 105074700 | - | 11613777 | 11613880 | 11612216 | 11612962 | 11614492 | 11614711 | 70 | 0 | 44 | 7 | 192 | 135 | 0.017430198 | 1 | 0.815 | 0.185 |  |  |
|  |  | 105076164 | - | 2627428 | 2627529 | 2624121 | 2624429 | 2627984 | 2628082 | 3 | 4 | 10 | 0 | 144 | 91 | 0.017534446 | 0.322 | 1 | -0.678 |  |  |
|  |  | 105070278 | + | 9265757 | 9265814 | 9246080 | 9246283 | 9277643 | 9277777 | 166 | 125 | 179 | 68 | 92 | 127 | 0.017545888 | 0.647 | 0.784 | -0.137 |  |  |
|  |  | 105068634 | - | 828942 | 829218 | 825629 | 827166 | 831519 | 831706 | 62 | 53 | 33 | 75 | 397 | 135 | 0.017731797 | 0.285 | 0.13 | 0.155 |  |  |
|  |  | 105076490 | - | 3925996 | 3926056 | 3925465 | 3925536 | 3927228 | 3927785 | 5 | 0 | 0 | 3 | 53 | 64 | 0.017892713 | 1 | 0 | 1 |  |  |
|  |  | 105072880 | + | 385762 | 385930 | 382480 | 383143 | 398986 | 399035 | 12 | 29 | 47 | 30 | 196 | 42 | 0.018350732 | 0.081 | 0.251 | -0.17 |  |  |
|  |  | 105064980 | + | 1219910 | 1219972 | 1219652 | 1219730 | 1221618 | 1221720 | 2 | 6 | 5 | 0 | 15 | 31 | 0.01836706 | 0.408 | 1 | -0.592 |  |  |
|  |  | 105083845 | - | 6081684 | 6081804 | 6081320 | 6081530 | 6084026 | 6084197 | 0 | 16 | 7 | 12 | 226 | 135 | 0.01847009 | 0 | 0.258 | -0.258 |  |  |
|  |  | 105062030 | + | 1965552 | 1965792 | 1964444 | 1964646 | 1971809 | 1972833 | 43 | 10 | 46 | 1 | 361 | 135 | 0.018472289 | 0.617 | 0.945 | -0.328 |  |  |
|  |  | 105081771 | - | 6209881 | 6209902 | 6209587 | 6209732 | 6211312 | 6211384 | 7 | 12 | 1 | 22 | 14 | 65 | 0.018604127 | 0.73 | 0.174 | 0.556 |  |  |
|  |  | 105070251 | + | 4995386 | 4996360 | 4986933 | 4986986 | 5009884 | 5010332 | 115 | 0 | 158 | 8 | 1006 | 46 | 0.018624548 | 1 | 0.475 | 0.525 |  |  |
|  |  | 105074971 | + | 9995026 | 9995205 | 9987242 | 9987363 | 10004831 | 10004947 | 127 | 8 | 212 | 0 | 253 | 88 | 0.018747835 | 0.847 | 1 | -0.153 |  |  |
|  |  | 105078142 | - | 859925 | 860124 | 859782 | 859854 | 860265 | 860429 | 76 | 3 | 20 | 6 | 250 | 65 | 0.019102557 | 0.868 | 0.464 | 0.404 |  |  |
|  |  | 105067259 | - | 1022667 | 1022837 | 1020887 | 1021025 | 1023000 | 1023145 | 19 | 4 | 52 | 0 | 287 | 131 | 0.019344058 | 0.684 | 1 | -0.316 |  |  |
|  |  | 105067979 | - | 3791812 | 3791993 | 3788340 | 3788508 | 3831102 | 3831206 | 19 | 4 | 97 | 1 | 264 | 97 | 0.019363123 | 0.636 | 0.973 | -0.337 |  |  |
|  |  | 105074937 | + | 6376765 | 6376871 | 6346189 | 6346345 | 6441728 | 6441797 | 17 | 0 | 10 | 5 | 125 | 62 | 0.019706338 | 1 | 0.498 | 0.502 |  |  |
|  |  | 105082149 | - | 25299017 | 25299182 | 25291142 | 25291359 | 25325331 | 25325514 | 0 | 6 | 5 | 2 | 286 | 135 | 0.019720291 | 0 | 0.541 | -0.541 |  |  |
|  |  | 105065732 | - | 7954154 | 7954289 | 7951062 | 7951098 | 7956276 | 7956426 | 21 | 2 | 15 | 11 | 150 | 29 | 0.019817464 | 0.67 | 0.209 | 0.461 |  |  |
|  |  | 105079401 | - | 3531379 | 3531562 | 3529010 | 3529162 | 3533089 | 3533243 | 36 | 17 | 86 | 12 | 304 | 135 | 0.019911497 | 0.485 | 0.761 | -0.276 |  |  |
|  |  | 105062811 | - | 1609724 | 1609820 | 1514391 | 1514489 | 1642956 | 1643100 | 7 | 3 | 38 | 0 | 134 | 91 | 0.020034152 | 0.613 | 1 | -0.387 |  |  |
|  |  | 105078982 | - | 4900717 | 4900896 | 4899118 | 4899240 | 4902502 | 4903341 | 16 | 0 | 39 | 14 | 280 | 115 | 0.020163863 | 1 | 0.534 | 0.466 |  |  |
|  |  | 105083068 | + | 4435667 | 4435771 | 4426004 | 4426092 | 4440114 | 4440261 | 10 | 4 | 25 | 0 | 140 | 81 | 0.020457998 | 0.591 | 1 | -0.409 |  |  |
|  |  | 105070726 | + | 2172566 | 2172839 | 2171681 | 2171954 | 2175612 | 2175885 | 14 | 0 | 28 | 12 | 394 | 135 | 0.020505405 | 1 | 0.444 | 0.556 |  |  |
|  |  | 105064101 | + | 7614436 | 7614553 | 7613919 | 7614125 | 7614653 | 7614831 | 28 | 11 | 27 | 1 | 220 | 135 | 0.020639253 | 0.61 | 0.943 | -0.333 |  |  |
|  |  | 105079160 | - | 202572 | 202668 | 187205 | 187391 | 204427 | 204579 | 7 | 6 | 10 | 0 | 178 | 135 | 0.020735036 | 0.469 | 1 | -0.531 |  |  |
|  |  | 105068300 | + | 370320 | 370491 | 369804 | 370019 | 370926 | 371136 | 21 | 0 | 13 | 5 | 292 | 135 | 0.02075563 | 1 | 0.546 | 0.454 |  |  |
|  |  | 105064160 | - | 998249 | 998388 | 997289 | 997436 | 998602 | 998759 | 7 | 3 | 31 | 0 | 264 | 135 | 0.020988727 | 0.544 | 1 | -0.456 |  |  |
|  |  | 105066000 | - | 1085054 | 1085144 | 1084893 | 1084943 | 1085373 | 1085533 | 0 | 5 | 7 | 3 | 83 | 43 | 0.021067913 | 0 | 0.547 | -0.547 |  |  |
|  |  | 105072129 | - | 779835 | 779946 | 771541 | 772630 | 780719 | 780983 | 7 | 1 | 1 | 5 | 208 | 135 | 0.021087435 | 0.82 | 0.115 | 0.705 |  |  |
|  |  | 105075016 | - | 2155157 | 2155240 | 2154383 | 2154488 | 2157315 | 2157411 | 176 | 29 | 423 | 20 | 69 | 52 | 0.021259858 | 0.821 | 0.941 | -0.12 |  |  |
|  |  | 105071213 | - | 70522 | 70543 | 69824 | 70015 | 74564 | 74621 | 7 | 8 | 2 | 21 | 14 | 50 | 0.021330716 | 0.758 | 0.254 | 0.504 |  |  |
|  |  | 105064037 | - | 7332529 | 7332680 | 7332304 | 7332365 | 7332934 | 7333101 | 20 | 8 | 38 | 2 | 191 | 54 | 0.021358558 | 0.414 | 0.843 | -0.429 |  |  |
|  |  | 105081656 | - | 4448720 | 4448821 | 4446264 | 4446393 | 4449994 | 4450058 | 6 | 0 | 9 | 12 | 97 | 44 | 0.0214692 | 1 | 0.254 | 0.746 |  |  |
|  |  | 105074560 | + | 223662 | 223833 | 219335 | 219659 | 224735 | 225239 | 14 | 12 | 8 | 0 | 292 | 135 | 0.021643459 | 0.35 | 1 | -0.65 |  |  |
|  |  | 105082658 | + | 12581457 | 12581508 | 12563172 | 12563254 | 12592171 | 12592345 | 3 | 7 | 17 | 4 | 44 | 75 | 0.021788618 | 0.422 | 0.879 | -0.457 |  |  |
|  |  | 105067108 | - | 4264992 | 4265157 | 4209225 | 4209824 | 4401392 | 4401519 | 10 | 10 | 50 | 11 | 271 | 120 | 0.021892545 | 0.307 | 0.668 | -0.361 |  |  |
|  |  | 105074562 | + | 400162 | 400314 | 399892 | 400085 | 400536 | 400650 | 97 | 0 | 28 | 4 | 245 | 107 | 0.021941618 | 1 | 0.754 | 0.246 |  |  |
|  |  | 105073298 | - | 5580742 | 5580962 | 5574871 | 5575026 | 5581409 | 5581470 | 40 | 11 | 82 | 5 | 260 | 54 | 0.02194986 | 0.43 | 0.773 | -0.343 |  |  |
|  |  | 105081023 | + | 3684790 | 3684928 | 3682307 | 3682430 | 3687632 | 3687785 | 9 | 11 | 67 | 20 | 243 | 116 | 0.022234178 | 0.281 | 0.615 | -0.334 |  |  |
|  |  | 105069130 | + | 4605683 | 4605772 | 4599124 | 4599199 | 4641859 | 4642455 | 4 | 0 | 4 | 11 | 97 | 68 | 0.022296709 | 1 | 0.203 | 0.797 |  |  |
|  |  | 105082307 | - | 3171276 | 3171444 | 3170877 | 3171066 | 3171613 | 3171995 | 69 | 0 | 59 | 7 | 289 | 135 | 0.022522303 | 1 | 0.797 | 0.203 |  |  |
|  |  | 105083395 | - | 11390986 | 11391037 | 11390642 | 11390869 | 11391120 | 11391158 | 7 | 6 | 17 | 1 | 44 | 31 | 0.022603434 | 0.451 | 0.923 | -0.472 |  |  |
|  |  | 105079863 | - | 3185831 | 3185940 | 3144847 | 3144917 | 3193612 | 3193713 | 76 | 7 | 72 | 0 | 91 | 22 | 0.022673732 | 0.724 | 1 | -0.276 |  |  |
|  |  | 105080789 | - | 10598868 | 10599135 | 10597932 | 10598010 | 10621073 | 10621356 | 46 | 22 | 62 | 9 | 324 | 71 | 0.022676211 | 0.314 | 0.602 | -0.288 |  |  |
|  |  | 105072087 | - | 60304 | 60328 | 60029 | 60193 | 60558 | 60647 | 7 | 80 | 8 | 19 | 17 | 82 | 0.022684385 | 0.297 | 0.67 | -0.373 |  |  |
|  |  | 105069608 | - | 439819 | 439957 | 433812 | 433938 | 503891 | 504059 | 156 | 11 | 297 | 0 | 246 | 119 | 0.022740071 | 0.873 | 1 | -0.127 |  |  |
|  |  | 105078430 | + | 1229314 | 1231411 | 1227898 | 1228404 | 1232720 | 1232846 | 209 | 1 | 318 | 13 | 2202 | 119 | 0.022805966 | 0.919 | 0.569 | 0.35 |  |  |
|  |  | 105081058 | - | 109439 | 109530 | 103204 | 103388 | 115970 | 116113 | 28 | 0 | 75 | 16 | 168 | 135 | 0.022922047 | 1 | 0.79 | 0.21 |  |  |
|  |  | 105069059 | + | 360921 | 361021 | 347954 | 348093 | 366672 | 366849 | 63 | 8 | 173 | 0 | 183 | 132 | 0.02304377 | 0.85 | 1 | -0.15 |  |  |
|  |  | 105064052 | - | 7574546 | 7574822 | 7573668 | 7573938 | 7575001 | 7575289 | 23 | 0 | 6 | 3 | 397 | 135 | 0.023072912 | 1 | 0.405 | 0.595 |  |  |
|  |  | 105072110 | + | 1457498 | 1457690 | 1448723 | 1449054 | 1477613 | 1478694 | 21 | 8 | 29 | 1 | 313 | 135 | 0.023166887 | 0.531 | 0.926 | -0.395 |  |  |
|  |  | 105066425 | - | 691574 | 691716 | 676089 | 676283 | 704786 | 704930 | 56 | 16 | 75 | 5 | 270 | 135 | 0.023254172 | 0.636 | 0.882 | -0.246 |  |  |
|  |  | 105069956 | + | 2985761 | 2985818 | 2960175 | 2960307 | 2992266 | 2992355 | 0 | 22 | 5 | 14 | 40 | 72 | 0.023465436 | 0 | 0.391 | -0.391 |  |  |
|  |  | 105073786 | + | 113527 | 113644 | 109956 | 110115 | 114524 | 115591 | 43 | 0 | 16 | 4 | 220 | 135 | 0.023660925 | 1 | 0.711 | 0.289 |  |  |
|  |  | 105084208 | + | 7504373 | 7504468 | 7499861 | 7500025 | 7505694 | 7505787 | 27 | 0 | 27 | 7 | 127 | 86 | 0.024007938 | 1 | 0.723 | 0.277 |  |  |
|  |  | 105079847 | - | 886051 | 886180 | 885637 | 885872 | 888462 | 888684 | 2 | 2 | 28 | 0 | 244 | 135 | 0.024117788 | 0.356 | 1 | -0.644 |  |  |
|  |  | 105064080 | + | 13781806 | 13781854 | 13779705 | 13779798 | 13783693 | 13783852 | 9 | 85 | 2 | 200 | 41 | 86 | 0.024802035 | 0.182 | 0.021 | 0.161 |  |  |
|  |  | 105072696 | - | 6698801 | 6698903 | 6695520 | 6695688 | 6702888 | 6703077 | 0 | 2 | 8 | 0 | 190 | 135 | 0.024893712 | 0 | 1 | -1 |  |  |
|  |  | 105081805 | + | 7942711 | 7943434 | 7938113 | 7938226 | 7944045 | 7944408 | 116 | 15 | 43 | 17 | 815 | 106 | 0.024897672 | 0.501 | 0.248 | 0.253 |  |  |
|  |  | 105063656 | + | 498207 | 498283 | 497826 | 497946 | 498718 | 498783 | 0 | 2 | 8 | 0 | 47 | 36 | 0.025110952 | 0 | 1 | -1 |  |  |
|  |  | 105074299 | - | 304474 | 304666 | 303908 | 303971 | 305697 | 305785 | 939 | 48 | 296 | 4 | 180 | 2 | 0.025116824 | 0.179 | 0.451 | -0.272 |  |  |
|  |  | 105075834 | + | 3374251 | 3374353 | 3358615 | 3358720 | 3375413 | 3375515 | 5 | 0 | 1 | 4 | 113 | 58 | 0.025152609 | 1 | 0.114 | 0.886 |  |  |
|  |  | 105077666 | - | 86133 | 86253 | 76000 | 76279 | 101141 | 101276 | 77 | 9 | 137 | 1 | 219 | 128 | 0.025641876 | 0.833 | 0.988 | -0.155 |  |  |
|  |  | 105076525 | - | 11697835 | 11697898 | 11697253 | 11697547 | 11698050 | 11698212 | 81 | 75 | 112 | 198 | 112 | 135 | 0.02571061 | 0.566 | 0.405 | 0.161 |  |  |
|  |  | 105077704 | + | 4183481 | 4183544 | 4161291 | 4161347 | 4187692 | 4187914 | 4 | 0 | 11 | 22 | 56 | 49 | 0.025770066 | 1 | 0.304 | 0.696 |  |  |
|  |  | 105070882 | + | 2087105 | 2087261 | 2086029 | 2086139 | 2088778 | 2088902 | 6 | 5 | 25 | 2 | 227 | 85 | 0.025924736 | 0.31 | 0.824 | -0.514 |  |  |
|  |  | 105078698 | - | 4244843 | 4245035 | 4243468 | 4243595 | 4245852 | 4245946 | 43 | 5 | 65 | 0 | 250 | 72 | 0.025964677 | 0.712 | 1 | -0.288 |  |  |
|  |  | 105069988 | - | 356351 | 356470 | 350901 | 351061 | 360851 | 360982 | 48 | 0 | 27 | 5 | 213 | 124 | 0.026256494 | 1 | 0.759 | 0.241 |  |  |
|  |  | 105072886 | - | 1126376 | 1126460 | 1126068 | 1126152 | 1126746 | 1126830 | 0 | 3 | 4 | 0 | 38 | 19 | 0.026437909 | 0 | 1 | -1 |  |  |
|  |  | 105064731 | + | 2582013 | 2582106 | 2575146 | 2575316 | 2583298 | 2583388 | 0 | 20 | 14 | 43 | 120 | 83 | 0.026638262 | 0 | 0.184 | -0.184 |  |  |
|  |  | 105066042 | - | 2005523 | 2005609 | 2002326 | 2004312 | 2008950 | 2009073 | 60 | 16 | 114 | 7 | 139 | 116 | 0.026877961 | 0.758 | 0.931 | -0.173 |  |  |
|  |  | 105067524 | + | 3901325 | 3901520 | 3900530 | 3900675 | 3904053 | 3904233 | 0 | 5 | 10 | 6 | 316 | 135 | 0.026958066 | 0 | 0.416 | -0.416 |  |  |
|  |  | 105074471 | + | 185258 | 185385 | 181272 | 181363 | 192856 | 194565 | 9 | 6 | 34 | 3 | 189 | 84 | 0.027012053 | 0.4 | 0.834 | -0.434 |  |  |
|  |  | 105067676 | - | 10184005 | 10184170 | 10181005 | 10181137 | 10186332 | 10186605 | 106 | 18 | 158 | 7 | 276 | 125 | 0.027618253 | 0.727 | 0.911 | -0.184 |  |  |
|  |  | 105083815 | + | 3699491 | 3699657 | 3696101 | 3696373 | 3701799 | 3701929 | 28 | 0 | 51 | 10 | 275 | 123 | 0.02775448 | 1 | 0.695 | 0.305 |  |  |
|  |  | 105078612 | + | 6266589 | 6266752 | 6262490 | 6262684 | 6267679 | 6267803 | 0 | 4 | 3 | 0 | 266 | 117 | 0.027832793 | 0 | 1 | -1 |  |  |
|  |  | 105073627 | + | 3531003 | 3531211 | 3530794 | 3530909 | 3531286 | 3532591 | 9 | 7 | 9 | 0 | 302 | 108 | 0.027944092 | 0.315 | 1 | -0.685 |  |  |
|  |  | 105083742 | + | 174467 | 174594 | 173714 | 173911 | 177162 | 177398 | 17 | 0 | 8 | 4 | 240 | 135 | 0.028028138 | 1 | 0.529 | 0.471 |  |  |
|  |  | 105069531 | + | 2014672 | 2015002 | 2011265 | 2011413 | 2019085 | 2019424 | 77 | 8 | 202 | 3 | 451 | 135 | 0.028273686 | 0.742 | 0.953 | -0.211 |  |  |
|  |  | 105080002 | + | 5087184 | 5087363 | 5085759 | 5085842 | 5088767 | 5088878 | 18 | 2 | 4 | 5 | 210 | 45 | 0.02859642 | 0.659 | 0.146 | 0.513 |  |  |
|  |  | 105071546 | + | 3511524 | 3511619 | 3511221 | 3511439 | 3511955 | 3511998 | 9 | 3 | 31 | 0 | 88 | 36 | 0.028869318 | 0.551 | 1 | -0.449 |  |  |
|  |  | 105074830 | + | 6646865 | 6646978 | 6645501 | 6645618 | 6666678 | 6667468 | 29 | 189 | 23 | 46 | 187 | 110 | 0.0291119 | 0.083 | 0.227 | -0.144 |  |  |
|  |  | 105079448 | + | 896695 | 896832 | 887169 | 887268 | 909260 | 909353 | 7 | 3 | 45 | 1 | 168 | 43 | 0.029224391 | 0.374 | 0.92 | -0.546 |  |  |
|  |  | 105064650 | + | 409535 | 409561 | 404667 | 404813 | 427878 | 428156 | 19 | 48 | 57 | 55 | 38 | 135 | 0.029250307 | 0.584 | 0.786 | -0.202 |  |  |
|  |  | 105062366 | - | 998071 | 998194 | 993277 | 993372 | 999998 | 1000115 | 43 | 6 | 97 | 1 | 160 | 63 | 0.029372351 | 0.738 | 0.974 | -0.236 |  |  |
|  |  | 105062992 | - | 1025696 | 1025735 | 1023885 | 1024064 | 1026243 | 1026355 | 12 | 10 | 11 | 38 | 34 | 105 | 0.029464402 | 0.787 | 0.472 | 0.315 |  |  |
|  |  | 105071285 | + | 5291814 | 5291895 | 4928226 | 4928430 | 5301071 | 5301224 | 7 | 4 | 16 | 0 | 148 | 135 | 0.029570524 | 0.615 | 1 | -0.385 |  |  |
|  |  | 105082568 | + | 1437169 | 1437376 | 1436492 | 1436737 | 1437710 | 1437937 | 164 | 0 | 54 | 5 | 328 | 135 | 0.029632701 | 1 | 0.816 | 0.184 |  |  |
|  |  | 105080681 | + | 8525488 | 8525611 | 8525303 | 8525368 | 8527288 | 8527402 | 11 | 0 | 12 | 7 | 127 | 30 | 0.029763405 | 1 | 0.288 | 0.712 |  |  |
|  |  | 105061722 | + | 429836 | 429899 | 423627 | 423714 | 432399 | 432624 | 53 | 136 | 91 | 120 | 57 | 80 | 0.029993517 | 0.354 | 0.516 | -0.162 |  |  |
|  |  | 105068997 | + | 10386433 | 10386517 | 10384929 | 10385260 | 10387754 | 10387939 | 5 | 0 | 3 | 6 | 154 | 135 | 0.030209927 | 1 | 0.305 | 0.695 |  |  |
|  |  | 105081110 | + | 970286 | 970437 | 969054 | 969158 | 972537 | 972752 | 65 | 6 | 98 | 0 | 234 | 97 | 0.030377019 | 0.818 | 1 | -0.182 |  |  |
|  |  | 105081091 | - | 5125752 | 5125957 | 5123323 | 5124074 | 5127853 | 5127940 | 2 | 3 | 9 | 0 | 271 | 80 | 0.030536273 | 0.164 | 1 | -0.836 |  |  |
|  |  | 105072398 | + | 9811730 | 9811857 | 9809389 | 9809542 | 9812227 | 9812374 | 16 | 6 | 17 | 0 | 240 | 135 | 0.030611064 | 0.6 | 1 | -0.4 |  |  |
|  |  | 105073291 | - | 1997586 | 1997736 | 1996964 | 1997138 | 1998593 | 1998642 | 126 | 6 | 219 | 0 | 178 | 42 | 0.031090567 | 0.832 | 1 | -0.168 |  |  |
|  |  | 105077726 | + | 6686164 | 6686248 | 6684293 | 6684751 | 6690933 | 6691111 | 14 | 6 | 18 | 32 | 154 | 135 | 0.031497464 | 0.672 | 0.33 | 0.342 |  |  |
|  |  | 105076451 | - | 1349703 | 1349892 | 1348553 | 1348762 | 1350415 | 1350495 | 88 | 17 | 125 | 7 | 248 | 73 | 0.031675455 | 0.604 | 0.84 | -0.236 |  |  |
|  |  | 105066927 | - | 363670 | 363960 | 361608 | 361680 | 385553 | 385775 | 362 | 223 | 315 | 346 | 341 | 65 | 0.031770816 | 0.236 | 0.148 | 0.088 |  |  |
|  |  | 105076047 | - | 83791 | 83911 | 81991 | 82168 | 88690 | 88871 | 2 | 4 | 6 | 0 | 226 | 135 | 0.032250656 | 0.23 | 1 | -0.77 |  |  |
|  |  | 105081805 | + | 8008337 | 8009111 | 8006451 | 8006646 | 8019810 | 8019869 | 92 | 33 | 26 | 35 | 812 | 52 | 0.032280555 | 0.151 | 0.045 | 0.106 |  |  |
|  |  | 105079766 | + | 1642350 | 1642506 | 1641138 | 1641368 | 1644113 | 1644761 | 58 | 27 | 133 | 25 | 277 | 135 | 0.032380281 | 0.511 | 0.722 | -0.211 |  |  |
|  |  | 105073818 | - | 956106 | 956244 | 955899 | 956028 | 956408 | 956581 | 172 | 5 | 185 | 23 | 249 | 122 | 0.03238703 | 0.944 | 0.798 | 0.146 |  |  |
|  |  | 105069520 | + | 1689901 | 1690006 | 1689070 | 1689223 | 1690186 | 1690264 | 70 | 12 | 123 | 4 | 132 | 71 | 0.032528985 | 0.758 | 0.943 | -0.185 |  |  |
|  |  | 105076691 | - | 31548016 | 31548114 | 31545042 | 31547929 | 31548421 | 31548547 | 20 | 2 | 3 | 4 | 166 | 119 | 0.032744536 | 0.878 | 0.35 | 0.528 |  |  |
|  |  | 105081627 | + | 12420895 | 12422422 | 12418900 | 12419112 | 12422794 | 12423012 | 2339 | 26 | 3427 | 85 | 1648 | 135 | 0.03321585 | 0.881 | 0.768 | 0.113 |  |  |
|  |  | 105082774 | + | 6031503 | 6031560 | 6030921 | 6031259 | 6031916 | 6032540 | 632 | 224 | 890 | 503 | 100 | 135 | 0.03355638 | 0.792 | 0.705 | 0.087 |  |  |
|  |  | 105071070 | - | 16487774 | 16487829 | 16487533 | 16487696 | 16488619 | 16488790 | 15 | 49 | 27 | 31 | 96 | 135 | 0.03361666 | 0.301 | 0.551 | -0.25 |  |  |
|  |  | 105081933 | - | 291874 | 292120 | 284089 | 284275 | 311072 | 311156 | 12 | 12 | 43 | 11 | 309 | 77 | 0.033690292 | 0.199 | 0.493 | -0.294 |  |  |
|  |  | 105082710 | + | 133394 | 133492 | 132290 | 132472 | 142826 | 142930 | 0 | 6 | 4 | 2 | 144 | 97 | 0.033799946 | 0 | 0.574 | -0.574 |  |  |
|  |  | 105081720 | + | 3138563 | 3138722 | 3136575 | 3136860 | 3139388 | 3140002 | 257 | 19 | 558 | 7 | 280 | 135 | 0.033900081 | 0.867 | 0.975 | -0.108 |  |  |
|  |  | 105075683 | - | 1355331 | 1355421 | 1351358 | 1351441 | 1363064 | 1363207 | 6 | 1 | 0 | 3 | 107 | 76 | 0.034047856 | 0.81 | 0 | 0.81 |  |  |
|  |  | 105070726 | + | 2187784 | 2188057 | 2163016 | 2163280 | 2193434 | 2193557 | 106 | 10 | 146 | 37 | 375 | 116 | 0.034237506 | 0.766 | 0.55 | 0.216 |  |  |
|  |  | 105079039 | + | 11965333 | 11965503 | 11962974 | 11963177 | 11970703 | 11970972 | 67 | 6 | 130 | 0 | 291 | 135 | 0.03441798 | 0.838 | 1 | -0.162 |  |  |
|  |  | 105070272 | + | 6925971 | 6926198 | 6923133 | 6923308 | 6926481 | 6926844 | 43 | 1 | 66 | 13 | 348 | 135 | 0.034519224 | 0.943 | 0.663 | 0.28 |  |  |
|  |  | 105083436 | - | 735052 | 735136 | 734211 | 734258 | 739171 | 739327 | 19 | 27 | 24 | 10 | 77 | 40 | 0.034736768 | 0.268 | 0.555 | -0.287 |  |  |
|  |  | 105069401 | + | 2593665 | 2593689 | 2592954 | 2593078 | 2594744 | 2594968 | 4 | 1 | 1 | 8 | 17 | 117 | 0.034830207 | 0.965 | 0.462 | 0.503 |  |  |
|  |  | 105083851 | + | 6455103 | 6455325 | 6453742 | 6453813 | 6455532 | 6455625 | 19 | 3 | 48 | 0 | 223 | 15 | 0.034966555 | 0.299 | 1 | -0.701 |  |  |
|  |  | 105078719 | - | 3029057 | 3029238 | 3028746 | 3028889 | 3030302 | 3030568 | 56 | 16 | 105 | 9 | 302 | 135 | 0.034987993 | 0.61 | 0.839 | -0.229 |  |  |
|  |  | 105073856 | - | 977830 | 977976 | 977584 | 977737 | 978529 | 978644 | 26 | 0 | 3 | 2 | 243 | 108 | 0.035028077 | 1 | 0.4 | 0.6 |  |  |
|  |  | 105070450 | - | 1569362 | 1569562 | 1567936 | 1568289 | 1570499 | 1570642 | 31 | 0 | 39 | 7 | 321 | 135 | 0.03531453 | 1 | 0.701 | 0.299 |  |  |
|  |  | 105075001 | + | 945205 | 945244 | 941891 | 941995 | 945543 | 945668 | 0 | 3 | 4 | 0 | 15 | 80 | 0.035393505 | 0 | 1 | -1 |  |  |
|  |  | 105076975 | - | 9540206 | 9540344 | 9539555 | 9540004 | 9547780 | 9547982 | 92 | 0 | 149 | 12 | 262 | 135 | 0.035628537 | 1 | 0.865 | 0.135 |  |  |
|  |  | 105074971 | + | 10050800 | 10050869 | 10049193 | 10049341 | 10053046 | 10053121 | 1 | 15 | 9 | 13 | 62 | 68 | 0.03572293 | 0.068 | 0.432 | -0.364 |  |  |
|  |  | 105069544 | + | 2212299 | 2212367 | 2210075 | 2210317 | 2212604 | 2212668 | 77 | 16 | 251 | 12 | 61 | 57 | 0.036023075 | 0.818 | 0.951 | -0.133 |  |  |
|  |  | 105064584 | - | 3073306 | 3073453 | 3072264 | 3072408 | 3075550 | 3075694 | 25 | 8 | 43 | 2 | 270 | 135 | 0.036068434 | 0.61 | 0.915 | -0.305 |  |  |
|  |  | 105065318 | + | 2878555 | 2878719 | 2871369 | 2871537 | 2878934 | 2879148 | 30 | 4 | 81 | 0 | 285 | 135 | 0.036321407 | 0.78 | 1 | -0.22 |  |  |
|  |  | 105080112 | - | 492499 | 492671 | 486247 | 486863 | 492961 | 493079 | 85 | 10 | 45 | 0 | 269 | 111 | 0.036381244 | 0.778 | 1 | -0.222 |  |  |
|  |  | 105082880 | + | 7045489 | 7045516 | 7044331 | 7044543 | 7046258 | 7046447 | 12 | 0 | 27 | 16 | 40 | 135 | 0.036536946 | 1 | 0.851 | 0.149 |  |  |
|  |  | 105075793 | + | 2922553 | 2922706 | 2920726 | 2920807 | 2933357 | 2933455 | 3 | 5 | 14 | 2 | 169 | 30 | 0.036562813 | 0.096 | 0.554 | -0.458 |  |  |
|  |  | 105082598 | - | 2235348 | 2235454 | 2235204 | 2235272 | 2235667 | 2235758 | 17 | 4 | 27 | 0 | 73 | 10 | 0.036765054 | 0.368 | 1 | -0.632 |  |  |
|  |  | 105071139 | + | 5898886 | 5898995 | 5898433 | 5898715 | 5899070 | 5899147 | 5 | 4 | 10 | 0 | 139 | 70 | 0.036777729 | 0.386 | 1 | -0.614 |  |  |
|  |  | 105072614 | - | 2078867 | 2078938 | 2068162 | 2068695 | 2082680 | 2082746 | 2 | 0 | 0 | 6 | 64 | 59 | 0.037054601 | 1 | 0 | 1 |  |  |
|  |  | 105066695 | - | 19471939 | 19472081 | 19467826 | 19468004 | 19490205 | 19490356 | 14 | 0 | 11 | 5 | 270 | 135 | 0.037203412 | 1 | 0.524 | 0.476 |  |  |
|  |  | 105070375 | - | 1433997 | 1434050 | 1433412 | 1433558 | 1436723 | 1436850 | 54 | 43 | 24 | 46 | 77 | 120 | 0.037854709 | 0.662 | 0.448 | 0.214 |  |  |
|  |  | 105072386 | + | 9431659 | 9431734 | 9429680 | 9429702 | 9434674 | 9435170 | 7 | 6 | 1 | 19 | 68 | 15 | 0.037877033 | 0.205 | 0.011 | 0.194 |  |  |
|  |  | 105071661 | + | 222851 | 223160 | 218517 | 218630 | 233438 | 233684 | 214 | 40 | 400 | 137 | 401 | 106 | 0.038058859 | 0.586 | 0.436 | 0.15 |  |  |
|  |  | 105070845 | + | 307945 | 308047 | 307758 | 307812 | 311374 | 312668 | 9 | 17 | 54 | 31 | 102 | 47 | 0.038067669 | 0.196 | 0.445 | -0.249 |  |  |
|  |  | 105083140 | - | 3001632 | 3001785 | 2999711 | 2999808 | 3005214 | 3005885 | 57 | 22 | 64 | 8 | 229 | 90 | 0.038334028 | 0.505 | 0.759 | -0.254 |  |  |
|  |  | 105065795 | + | 4517902 | 4518046 | 4517151 | 4517215 | 4518408 | 4518518 | 16 | 3 | 40 | 0 | 160 | 25 | 0.038618011 | 0.455 | 1 | -0.545 |  |  |
|  |  | 105071216 | + | 351587 | 351675 | 351240 | 351517 | 352482 | 352570 | 9 | 11 | 6 | 38 | 108 | 81 | 0.038657063 | 0.38 | 0.106 | 0.274 |  |  |
|  |  | 105077924 | - | 4367907 | 4368094 | 4367666 | 4367804 | 4371000 | 4371096 | 25 | 3 | 106 | 0 | 258 | 85 | 0.038846996 | 0.733 | 1 | -0.267 |  |  |
|  |  | 105074141 | + | 4241 | 4417 | 3898 | 4121 | 8411 | 8581 | 24 | 5 | 90 | 2 | 297 | 135 | 0.039089656 | 0.686 | 0.953 | -0.267 |  |  |
|  |  | 105071451 | + | 9932505 | 9932682 | 9931652 | 9931771 | 9933264 | 9933355 | 35 | 4 | 62 | 0 | 224 | 61 | 0.039165819 | 0.704 | 1 | -0.296 |  |  |
|  |  | 105071719 | - | 767802 | 767908 | 767490 | 767622 | 768303 | 768519 | 18 | 4 | 39 | 0 | 188 | 125 | 0.039288024 | 0.75 | 1 | -0.25 |  |  |
|  |  | 105073361 | + | 168773 | 168870 | 166866 | 167004 | 186722 | 186850 | 243 | 20 | 121 | 0 | 162 | 117 | 0.039410993 | 0.898 | 1 | -0.102 |  |  |
|  |  | 105065793 | - | 860272 | 860355 | 859439 | 859563 | 861700 | 862165 | 23 | 27 | 8 | 32 | 134 | 117 | 0.039417479 | 0.427 | 0.179 | 0.248 |  |  |
|  |  | 105070882 | + | 2087147 | 2087261 | 2086029 | 2086139 | 2088778 | 2088902 | 8 | 5 | 28 | 2 | 164 | 85 | 0.039486853 | 0.453 | 0.879 | -0.426 |  |  |
|  |  | 105080772 | - | 8483065 | 8483109 | 8482448 | 8482640 | 8489188 | 8489327 | 14 | 6 | 21 | 0 | 71 | 132 | 0.039949304 | 0.813 | 1 | -0.187 |  |  |
|  |  | 105074101 | + | 831577 | 831668 | 831416 | 831492 | 831899 | 832043 | 10 | 8 | 13 | 1 | 102 | 69 | 0.040084609 | 0.458 | 0.898 | -0.44 |  |  |
|  |  | 105062313 | + | 12158293 | 12158335 | 12151419 | 12151620 | 12161712 | 12161870 | 0 | 14 | 10 | 29 | 70 | 135 | 0.040201452 | 0 | 0.399 | -0.399 |  |  |
|  |  | 105062060 | + | 2919201 | 2919326 | 2911431 | 2911544 | 2921146 | 2921253 | 131 | 3 | 106 | 13 | 172 | 71 | 0.040403147 | 0.947 | 0.771 | 0.176 |  |  |
|  |  | 105069898 | - | 327260 | 327380 | 326600 | 326755 | 327749 | 327931 | 147 | 28 | 56 | 2 | 226 | 135 | 0.040567603 | 0.758 | 0.944 | -0.186 |  |  |
|  |  | 105067466 | - | 22506267 | 22506413 | 22499225 | 22499391 | 22519941 | 22520092 | 65 | 6 | 121 | 0 | 270 | 135 | 0.041000677 | 0.844 | 1 | -0.156 |  |  |
|  |  | 105079021 | + | 10701248 | 10701437 | 10696379 | 10696518 | 10705540 | 10705687 | 81 | 0 | 80 | 7 | 307 | 132 | 0.041101761 | 1 | 0.831 | 0.169 |  |  |
|  |  | 105069534 | - | 2123124 | 2123247 | 2122452 | 2122615 | 2123472 | 2123581 | 68 | 15 | 279 | 20 | 199 | 102 | 0.041293992 | 0.699 | 0.877 | -0.178 |  |  |
|  |  | 105080785 | + | 10029559 | 10029713 | 10024609 | 10024721 | 10030700 | 10030874 | 54 | 6 | 192 | 2 | 245 | 105 | 0.041736479 | 0.794 | 0.976 | -0.182 |  |  |
|  |  | 105078739 | - | 824040 | 824220 | 822029 | 822189 | 826498 | 826590 | 140 | 252 | 231 | 911 | 251 | 85 | 0.041829855 | 0.158 | 0.079 | 0.079 |  |  |
|  |  | 105082252 | - | 22580685 | 22580895 | 22579923 | 22580496 | 22581121 | 22581177 | 11 | 5 | 13 | 0 | 245 | 49 | 0.042205924 | 0.306 | 1 | -0.694 |  |  |
|  |  | 105083170 | - | 6158584 | 6158822 | 6145480 | 6145675 | 6164476 | 6164588 | 126 | 8 | 101 | 0 | 329 | 105 | 0.042244728 | 0.834 | 1 | -0.166 |  |  |
|  |  | 105078955 | + | 1139845 | 1139986 | 1116959 | 1117183 | 1163605 | 1163727 | 0 | 2 | 10 | 1 | 248 | 115 | 0.042340505 | 0 | 0.823 | -0.823 |  |  |
|  |  | 105070783 | - | 5200115 | 5200196 | 5166507 | 5166632 | 5202656 | 5202765 | 10 | 0 | 10 | 6 | 98 | 85 | 0.042444288 | 1 | 0.591 | 0.409 |  |  |
|  |  | 105075877 | - | 192608 | 192704 | 190051 | 190218 | 213642 | 213689 | 1 | 18 | 19 | 35 | 89 | 40 | 0.042569535 | 0.024 | 0.196 | -0.172 |  |  |
|  |  | 105068809 | - | 1471895 | 1471991 | 1471425 | 1471731 | 1472532 | 1472643 | 2 | 6 | 6 | 1 | 147 | 104 | 0.042630992 | 0.191 | 0.809 | -0.618 |  |  |
|  |  | 105061762 | - | 3322759 | 3322887 | 3308113 | 3308247 | 3342003 | 3342186 | 27 | 4 | 65 | 0 | 234 | 127 | 0.043277713 | 0.786 | 1 | -0.214 |  |  |
|  |  | 105072344 | + | 1787680 | 1787818 | 1785998 | 1786136 | 1788266 | 1788401 | 18 | 0 | 11 | 4 | 251 | 124 | 0.043427116 | 1 | 0.576 | 0.424 |  |  |
|  |  | 105073421 | - | 2344737 | 2344931 | 2338661 | 2338779 | 2345822 | 2346128 | 2 | 2 | 15 | 0 | 291 | 111 | 0.043549706 | 0.276 | 1 | -0.724 |  |  |
|  |  | 105065301 | + | 1613952 | 1614202 | 1612871 | 1613014 | 1614817 | 1614913 | 113 | 6 | 158 | 0 | 325 | 89 | 0.043590572 | 0.838 | 1 | -0.162 |  |  |
|  |  | 105077423 | + | 20352572 | 20352836 | 20351727 | 20351834 | 20357206 | 20357387 | 2 | 0 | 0 | 11 | 350 | 100 | 0.043699459 | 1 | 0 | 1 |  |  |
|  |  | 105069469 | - | 12231282 | 12231492 | 12228732 | 12228921 | 12244139 | 12244589 | 39 | 7 | 28 | 0 | 331 | 135 | 0.04373797 | 0.694 | 1 | -0.306 |  |  |
|  |  | 105067834 | - | 2483155 | 2483251 | 2481640 | 2482237 | 2483818 | 2483951 | 17 | 4 | 36 | 0 | 169 | 126 | 0.043831163 | 0.76 | 1 | -0.24 |  |  |
|  |  | 105077111 | + | 5820217 | 5820322 | 5819991 | 5820124 | 5820506 | 5820660 | 52 | 21 | 57 | 7 | 187 | 126 | 0.044191381 | 0.625 | 0.846 | -0.221 |  |  |
|  |  | 105080681 | + | 8519239 | 8519320 | 8514460 | 8514608 | 8522312 | 8522437 | 28 | 6 | 33 | 0 | 131 | 118 | 0.04425635 | 0.808 | 1 | -0.192 |  |  |
|  |  | 105071763 | + | 1554107 | 1554261 | 1548029 | 1548092 | 1574763 | 1574913 | 8 | 0 | 6 | 5 | 196 | 56 | 0.044424122 | 1 | 0.255 | 0.745 |  |  |
|  |  | 105071672 | - | 2989350 | 2989579 | 2986727 | 2986901 | 2992023 | 2992542 | 34 | 0 | 109 | 14 | 350 | 135 | 0.044434089 | 1 | 0.75 | 0.25 |  |  |
|  |  | 105063558 | + | 3037030 | 3037651 | 3035404 | 3035524 | 3047153 | 3047738 | 49 | 33 | 52 | 103 | 720 | 113 | 0.044582319 | 0.189 | 0.073 | 0.116 |  |  |
|  |  | 105078854 | - | 1552654 | 1552723 | 1550935 | 1551020 | 1554792 | 1554908 | 8 | 15 | 31 | 16 | 41 | 52 | 0.044681176 | 0.403 | 0.711 | -0.308 |  |  |
|  |  | 105071762 | - | 1503253 | 1503388 | 1502723 | 1502837 | 1505718 | 1505829 | 17 | 4 | 83 | 2 | 197 | 76 | 0.044934855 | 0.621 | 0.941 | -0.32 |  |  |
|  |  | 105074881 | - | 400708 | 400793 | 377548 | 377689 | 435970 | 436101 | 158 | 26 | 86 | 34 | 144 | 123 | 0.044973945 | 0.838 | 0.684 | 0.154 |  |  |
|  |  | 105062081 | + | 86868 | 87015 | 82994 | 83168 | 90271 | 90428 | 42 | 23 | 53 | 10 | 270 | 135 | 0.045001584 | 0.477 | 0.726 | -0.249 |  |  |
|  |  | 105073653 | - | 469150 | 469282 | 448827 | 449080 | 489561 | 489638 | 26 | 0 | 4 | 2 | 185 | 70 | 0.045082688 | 1 | 0.431 | 0.569 |  |  |
|  |  | 105069345 | - | 1649586 | 1649715 | 1647328 | 1647445 | 1650121 | 1650263 | 39 | 10 | 60 | 3 | 219 | 110 | 0.04517853 | 0.662 | 0.909 | -0.247 |  |  |
|  |  | 105074406 | + | 2932130 | 2932226 | 2927292 | 2927419 | 2935941 | 2937884 | 20 | 7 | 15 | 0 | 163 | 120 | 0.045265046 | 0.678 | 1 | -0.322 |  |  |
|  |  | 105067832 | - | 2413988 | 2414135 | 2409477 | 2409696 | 2414977 | 2415139 | 29 | 0 | 4 | 2 | 270 | 135 | 0.045282525 | 1 | 0.5 | 0.5 |  |  |
|  |  | 105069129 | - | 4561896 | 4562020 | 4554925 | 4555096 | 4569405 | 4569502 | 4 | 6 | 50 | 14 | 189 | 90 | 0.045442593 | 0.241 | 0.63 | -0.389 |  |  |
|  |  | 105080215 | - | 1589436 | 1589518 | 1584887 | 1585058 | 1592887 | 1592988 | 5 | 0 | 0 | 2 | 109 | 94 | 0.045465305 | 1 | 0 | 1 |  |  |
|  |  | 105062604 | - | 3936099 | 3936172 | 3935882 | 3935983 | 3937626 | 3937734 | 3 | 3 | 10 | 0 | 57 | 60 | 0.045518264 | 0.513 | 1 | -0.487 |  |  |
|  |  | 105063955 | + | 5250079 | 5250206 | 5245764 | 5246601 | 5251225 | 5251399 | 5 | 4 | 9 | 0 | 240 | 135 | 0.045619622 | 0.413 | 1 | -0.587 |  |  |
|  |  | 105081766 | - | 5866584 | 5866706 | 5842353 | 5844167 | 5898698 | 5898739 | 18 | 0 | 12 | 4 | 129 | 34 | 0.045759063 | 1 | 0.442 | 0.558 |  |  |
|  |  | 105065177 | + | 158851 | 159047 | 155349 | 155669 | 160537 | 160631 | 15 | 22 | 43 | 21 | 269 | 87 | 0.04610669 | 0.181 | 0.398 | -0.217 |  |  |
|  |  | 105081591 | - | 10305922 | 10306240 | 10301768 | 10302050 | 10313845 | 10314159 | 31 | 0 | 63 | 9 | 439 | 135 | 0.046747386 | 1 | 0.683 | 0.317 |  |  |
|  |  | 105067524 | + | 3921686 | 3921754 | 3911067 | 3911196 | 3926321 | 3926561 | 13 | 0 | 4 | 3 | 109 | 122 | 0.046757378 | 1 | 0.599 | 0.401 |  |  |
|  |  | 105083022 | - | 883060 | 883141 | 879791 | 880155 | 884389 | 884487 | 5 | 3 | 15 | 0 | 104 | 91 | 0.046856453 | 0.593 | 1 | -0.407 |  |  |
|  |  | 105064733 | + | 241532 | 241632 | 227891 | 228039 | 245629 | 245702 | 77 | 7 | 173 | 0 | 117 | 66 | 0.046960935 | 0.861 | 1 | -0.139 |  |  |
|  |  | 105080530 | + | 504986 | 505211 | 504308 | 504428 | 505310 | 505456 | 82 | 14 | 24 | 0 | 324 | 113 | 0.046976931 | 0.671 | 1 | -0.329 |  |  |
|  |  | 105069988 | - | 281877 | 281894 | 278768 | 278913 | 307890 | 307932 | 8 | 0 | 0 | 2 | 10 | 35 | 0.047035397 | 1 | 0 | 1 |  |  |
|  |  | 105072884 | - | 924694 | 924828 | 920359 | 920685 | 930901 | 931053 | 6 | 2 | 89 | 0 | 254 | 135 | 0.047072332 | 0.615 | 1 | -0.385 |  |  |
|  |  | 105070229 | - | 3214364 | 3215009 | 3211986 | 3212025 | 3215503 | 3215620 | 112 | 0 | 148 | 6 | 638 | 7 | 0.047135645 | 1 | 0.213 | 0.787 |  |  |
|  |  | 105069706 | + | 858023 | 858105 | 855745 | 855837 | 859307 | 861757 | 27 | 1 | 11 | 5 | 100 | 85 | 0.047149513 | 0.958 | 0.652 | 0.306 |  |  |
|  |  | 105063427 | + | 1043778 | 1043845 | 1039507 | 1039587 | 1046433 | 1046570 | 2 | 2 | 36 | 0 | 55 | 68 | 0.04727038 | 0.553 | 1 | -0.447 |  |  |
|  |  | 105071561 | - | 4105353 | 4105512 | 4105011 | 4105170 | 4105609 | 4105773 | 42 | 6 | 40 | 0 | 280 | 135 | 0.047347989 | 0.771 | 1 | -0.229 |  |  |
|  |  | 105080837 | - | 12459 | 12513 | 7885 | 9668 | 14933 | 14969 | 22 | 4 | 45 | 0 | 47 | 29 | 0.047523643 | 0.772 | 1 | -0.228 |  |  |
|  |  | 105070564 | + | 526511 | 528140 | 520081 | 520708 | 544375 | 544558 | 873 | 1 | 1014 | 13 | 1750 | 135 | 0.047554898 | 0.985 | 0.857 | 0.128 |  |  |
|  |  | 105061780 | + | 4063775 | 4063877 | 4061608 | 4061722 | 4064841 | 4066484 | 0 | 5 | 2 | 0 | 162 | 107 | 0.047660635 | 0 | 1 | -1 |  |  |
|  |  | 105080493 | + | 8624350 | 8624359 | 8621659 | 8621887 | 8626648 | 8626749 | 4 | 23 | 0 | 34 | 2 | 94 | 0.047688906 | 0.891 | 0 | 0.891 |  |  |
|  |  | 105082139 | - | 24559363 | 24559463 | 24552565 | 24552655 | 24567058 | 24567169 | 68 | 6 | 140 | 0 | 103 | 52 | 0.047823704 | 0.851 | 1 | -0.149 |  |  |
|  |  | 105074119 | - | 244828 | 245051 | 208138 | 209032 | 263674 | 263844 | 14 | 4 | 21 | 0 | 344 | 135 | 0.047842414 | 0.579 | 1 | -0.421 |  |  |
|  |  | 105074924 | + | 3754716 | 3754901 | 3749155 | 3749338 | 3758755 | 3758930 | 24 | 0 | 126 | 21 | 306 | 135 | 0.047870935 | 1 | 0.726 | 0.274 |  |  |
|  |  | 105066554 | + | 2822405 | 2822441 | 2817478 | 2817667 | 2824135 | 2824534 | 4 | 0 | 6 | 10 | 58 | 135 | 0.047884379 | 1 | 0.583 | 0.417 |  |  |
|  |  | 105077678 | + | 2450067 | 2450277 | 2445551 | 2445737 | 2451121 | 2451324 | 64 | 0 | 149 | 12 | 331 | 135 | 0.047953202 | 1 | 0.835 | 0.165 |  |  |
|  |  | 105075191 | + | 1061556 | 1061595 | 1061212 | 1061392 | 1066620 | 1066723 | 3 | 2 | 0 | 7 | 32 | 96 | 0.047983156 | 0.818 | 0 | 0.818 |  |  |
|  |  | 105075121 | + | 5353969 | 5354005 | 5351115 | 5351250 | 5354752 | 5354865 | 3 | 0 | 0 | 3 | 22 | 99 | 0.048023695 | 1 | 0 | 1 |  |  |
|  |  | 105063065 | + | 5667390 | 5667472 | 5667011 | 5667118 | 5667958 | 5668042 | 22 | 4 | 64 | 0 | 57 | 42 | 0.04821195 | 0.802 | 1 | -0.198 |  |  |
|  |  | 105080606 | + | 5397643 | 5397772 | 5396478 | 5396518 | 5409690 | 5409975 | 0 | 3 | 7 | 2 | 142 | 33 | 0.048375888 | 0 | 0.449 | -0.449 |  |  |
|  |  | 105064321 | - | 453869 | 453959 | 452306 | 452405 | 456904 | 456987 | 21 | 0 | 19 | 5 | 64 | 33 | 0.048765544 | 1 | 0.662 | 0.338 |  |  |
|  |  | 105066520 | - | 4406590 | 4406710 | 4405218 | 4405338 | 4411519 | 4411642 | 60 | 6 | 292 | 2 | 185 | 94 | 0.048776262 | 0.836 | 0.987 | -0.151 |  |  |
|  |  | 105064973 | + | 31821 | 31999 | 31122 | 31259 | 34640 | 34729 | 50 | 0 | 44 | 5 | 241 | 77 | 0.048804793 | 1 | 0.738 | 0.262 |  |  |
|  |  | 105083574 | - | 18991752 | 18991827 | 18986746 | 18986922 | 18998548 | 18998657 | 1 | 2 | 10 | 0 | 103 | 102 | 0.049141033 | 0.331 | 1 | -0.669 |  |  |
|  |  | 105074724 | + | 14458456 | 14458603 | 14431383 | 14431493 | 14477058 | 14477115 | 9 | 8 | 12 | 1 | 153 | 18 | 0.049182107 | 0.117 | 0.585 | -0.468 |  |  |
|  |  | 105073407 | + | 130409 | 130715 | 121719 | 121788 | 141428 | 141695 | 87 | 6 | 71 | 0 | 354 | 62 | 0.049228005 | 0.717 | 1 | -0.283 |  |  |
|  |  | 105063641 | - | 179438 | 179537 | 178798 | 178905 | 180332 | 180441 | 95 | 19 | 159 | 69 | 116 | 67 | 0.049365617 | 0.743 | 0.571 | 0.172 |  |  |
|  |  | 105063680 | + | 1652674 | 1652759 | 1640026 | 1640168 | 1658687 | 1658819 | 59 | 10 | 133 | 3 | 146 | 125 | 0.049429708 | 0.835 | 0.974 | -0.139 |  |  |
|  |  | 105074070 | + | 433399 | 433504 | 432818 | 433071 | 433844 | 433957 | 14 | 9 | 14 | 1 | 167 | 106 | 0.04943642 | 0.497 | 0.899 | -0.402 |  |  |
|  |  | 105072599 | - | 621044 | 621118 | 619156 | 619317 | 623181 | 623235 | 76 | 0 | 97 | 10 | 67 | 47 | 0.049582096 | 1 | 0.872 | 0.128 |  |  |
|  |  | 105083397 | + | 11595545 | 11595777 | 11592799 | 11592978 | 11600512 | 11600598 | 27 | 3 | 74 | 0 | 297 | 79 | 0.049894037 | 0.705 | 1 | -0.295 |  |  |
|  |  | 105072879 | + | 822405 | 822480 | 817966 | 818059 | 827783 | 827949 | 3 | 2 | 0 | 7 | 87 | 86 | 0.049894421 | 0.597 | 0 | 0.597 |  |  |
| **AS category** | **Stress** | **GeneID** | **strand** | **riExonStart_0base** | **riExonEnd** | **upstreamES** | **upstreamEE** | **downstreamES** | **downstreamEE** | **IC_SAMPLE_1** | **SC_SAMPLE_1** | **IC_SAMPLE_2** | **SC_SAMPLE_2** | **IncFormLen** | **SkipFormLen** | **PValue** | **IncLevel1** | **IncLevel2** | **IncLevelDifference** |  |  |
| RI | SS | 105068759 | + | 479483 | 480757 | 479483 | 479787 | 480579 | 480757 | 219 | 34 | 255 | 6 | 913 | 135 | 2.25E-05 | 0.488 | 0.863 | -0.375 |  |  |
|  |  | 105070681 | - | 1427537 | 1428930 | 1427537 | 1427850 | 1428790 | 1428930 | 154 | 7 | 83 | 22 | 1059 | 133 | 0.000247038 | 0.734 | 0.321 | 0.413 |  |  |
|  |  | 105062041 | + | 2174343 | 2176086 | 2174343 | 2174548 | 2174698 | 2176086 | 29 | 21 | 50 | 6 | 271 | 135 | 0.001222116 | 0.408 | 0.806 | -0.398 |  |  |
|  |  | 105069546 | - | 2229328 | 2230167 | 2229328 | 2229603 | 2229954 | 2230167 | 155 | 34 | 81 | 50 | 472 | 135 | 0.001391243 | 0.566 | 0.317 | 0.249 |  |  |
|  |  | 105064620 | + | 362364 | 364513 | 362364 | 362558 | 364078 | 364513 | 1486 | 188 | 1301 | 102 | 1641 | 135 | 0.030874897 | 0.394 | 0.512 | -0.118 |  |  |
|  |  | 105079662 | - | 3173916 | 3174466 | 3173916 | 3174077 | 3174198 | 3174466 | 128 | 0 | 106 | 9 | 228 | 135 | 0.045944994 | 1 | 0.875 | 0.125 |  |  |
|  |  | 105080723 | + | 1823848 | 1824693 | 1823848 | 1824124 | 1824237 | 1824693 | 23 | 0 | 14 | 4 | 212 | 135 | 0.04951456 | 1 | 0.69 | 0.31 |  |  |
|  | WS | 105082776 | - | 6074373 | 6075030 | 6074373 | 6074578 | 6074875 | 6075030 | 73 | 10 | 28 | 50 | 418 | 135 | 2.52E-10 | 0.702 | 0.153 | 0.549 |  |  |
|  |  | 105063701 | + | 2833940 | 2834435 | 2833940 | 2834132 | 2834360 | 2834435 | 61 | 10 | 18 | 28 | 282 | 68 | 2.91E-06 | 0.595 | 0.134 | 0.461 |  |  |
|  |  | 105069546 | - | 2229328 | 2230167 | 2229328 | 2229603 | 2229954 | 2230167 | 116 | 14 | 81 | 50 | 472 | 135 | 5.57E-06 | 0.703 | 0.317 | 0.386 |  |  |
|  |  | 105064043 | + | 7368837 | 7371128 | 7368837 | 7369044 | 7370165 | 7371128 | 337 | 28 | 208 | 52 | 1242 | 135 | 0.000269902 | 0.567 | 0.303 | 0.264 |  |  |
|  |  | 105077946 | - | 9468133 | 9468589 | 9468133 | 9468397 | 9468499 | 9468589 | 50 | 2 | 49 | 23 | 138 | 83 | 0.000332854 | 0.938 | 0.562 | 0.376 |  |  |
|  |  | 105068797 | + | 1398706 | 1399074 | 1398706 | 1398717 | 1398867 | 1399074 | 196 | 13 | 207 | 1 | 140 | 4 | 0.00225918 | 0.301 | 0.855 | -0.554 |  |  |
| **AS category** | **Stress** | **GeneID** | **strand** | **1stExonStart_0base** | **1stExonEnd** | **2ndExonStart_0base** | **2ndExonEnd** | **upstreamES** | **upstreamEE** | **downstreamES** | **downstreamEE** | **IC_SAMPLE_1** | **SC_SAMPLE_1** | **IC_SAMPLE_2** | **SC_SAMPLE_2** | **IncFormLen** | **SkipFormLen** | **PValue** | **IncLevel1** | **IncLevel2** | **IncLevelDifference** |
| MXE | SS | 105064863 | + | 2699168 | 2699342 | 2699426 | 2699586 | 2698804 | 2698912 | 2699809 | 2699901 | 4480 | 11603 | 5794 | 8823 | 211 | 197 | 0 | 0.265 | 0.38 | -0.115 |
|  |  | 105066664 | - | 14599228 | 14599342 | 14601038 | 14601158 | 14594702 | 14594787 | 14601247 | 14601379 | 1210 | 476 | 993 | 843 | 159 | 147 | 3.11E-15 | 0.702 | 0.521 | 0.181 |
|  |  | 105075625 | - | 1977394 | 1978408 | 1979488 | 1979632 | 1976836 | 1976977 | 1980167 | 1980257 | 451 | 4082 | 341 | 6805 | 217 | 1082 | 4.44E-13 | 0.355 | 0.2 | 0.155 |
|  |  | 105079710 | + | 1074433 | 1074642 | 1075171 | 1075281 | 1072802 | 1072990 | 1075430 | 1075603 | 8559 | 8702 | 14861 | 10829 | 330 | 206 | 9.39E-11 | 0.38 | 0.461 | -0.081 |
|  |  | 105079347 | + | 72833 | 72928 | 82507 | 82645 | 55814 | 57208 | 102384 | 102489 | 792 | 425 | 796 | 874 | 139 | 225 | 2.00E-10 | 0.751 | 0.596 | 0.155 |
|  |  | 105078825 | - | 7171068 | 7171166 | 7181103 | 7181200 | 7170551 | 7170829 | 7181864 | 7181993 | 236 | 75 | 151 | 171 | 167 | 169 | 3.95E-10 | 0.761 | 0.472 | 0.289 |
|  |  | 105069522 | - | 3669248 | 3669437 | 3670929 | 3671070 | 3666157 | 3666467 | 3675530 | 3675561 | 321 | 1268 | 180 | 1834 | 157 | 199 | 1.90E-09 | 0.243 | 0.111 | 0.132 |
|  |  | 105069244 | - | 803772 | 803913 | 804626 | 804794 | 803277 | 803359 | 804921 | 805032 | 2432 | 1153 | 2361 | 1822 | 198 | 177 | 3.23E-09 | 0.653 | 0.537 | 0.116 |
|  |  | 105065267 | + | 901541 | 901657 | 903312 | 903498 | 899230 | 899357 | 904804 | 905008 | 474 | 1809 | 632 | 1332 | 203 | 292 | 1.53E-07 | 0.274 | 0.406 | -0.132 |
|  |  | 105077486 | - | 234112 | 234197 | 234320 | 234485 | 233636 | 234016 | 235300 | 235944 | 1998 | 494 | 2248 | 948 | 286 | 156 | 3.26E-07 | 0.688 | 0.564 | 0.124 |
|  |  | 105077806 | + | 6214872 | 6214999 | 6215743 | 6215902 | 6211852 | 6211978 | 6218602 | 6218768 | 121 | 31 | 69 | 74 | 224 | 264 | 2.44E-06 | 0.821 | 0.524 | 0.297 |
|  |  | 105066281 | + | 18803864 | 18804026 | 18809146 | 18809341 | 18777927 | 18778054 | 18818604 | 18818710 | 203 | 71 | 184 | 173 | 232 | 265 | 6.87E-06 | 0.766 | 0.549 | 0.217 |
|  |  | 105073945 | - | 9303304 | 9303466 | 9304857 | 9304933 | 9302894 | 9302998 | 9307103 | 9307253 | 71 | 536 | 220 | 708 | 100 | 245 | 9.54E-06 | 0.245 | 0.432 | -0.187 |
|  |  | 105081628 | - | 12410022 | 12410870 | 12412851 | 12412885 | 12406164 | 12406383 | 12413715 | 12413797 | 25 | 1451 | 1 | 1416 | 27 | 909 | 9.91E-06 | 0.367 | 0.023 | 0.344 |
|  |  | 105070102 | - | 1698587 | 1698744 | 1700255 | 1700464 | 1697195 | 1697951 | 1702731 | 1702930 | 476 | 115 | 371 | 200 | 330 | 278 | 2.60E-05 | 0.777 | 0.61 | 0.167 |
|  |  | 105079029 | + | 11346523 | 11346687 | 11354214 | 11354383 | 11338645 | 11338875 | 11369936 | 11370067 | 226 | 86 | 167 | 157 | 274 | 279 | 3.48E-05 | 0.728 | 0.52 | 0.208 |
|  |  | 105064031 | - | 7270953 | 7271068 | 7271222 | 7271346 | 7269781 | 7270035 | 7271681 | 7271863 | 67 | 30 | 26 | 53 | 234 | 216 | 3.53E-05 | 0.673 | 0.312 | 0.361 |
|  |  | 105081677 | - | 1236064 | 1236171 | 1238948 | 1239044 | 1235090 | 1235308 | 1241748 | 1241971 | 114 | 786 | 262 | 813 | 178 | 200 | 4.87E-05 | 0.14 | 0.266 | -0.126 |
|  |  | 105073855 | - | 944140 | 944292 | 944375 | 944456 | 943910 | 944057 | 944541 | 944684 | 70 | 27 | 23 | 44 | 148 | 273 | 4.98E-05 | 0.827 | 0.491 | 0.336 |
|  |  | 105072394 | - | 9637915 | 9638021 | 9639529 | 9639676 | 9636915 | 9637374 | 9639965 | 9640077 | 277 | 249 | 756 | 369 | 240 | 168 | 0.000151694 | 0.438 | 0.589 | -0.151 |
|  |  | 105064080 | + | 13773405 | 13773525 | 13775514 | 13775724 | 13766456 | 13766610 | 13776809 | 13776990 | 322 | 118 | 394 | 303 | 226 | 331 | 0.000160114 | 0.8 | 0.656 | 0.144 |
|  |  | 105077112 | - | 5829103 | 5829259 | 5829357 | 5829462 | 5828359 | 5828460 | 5829803 | 5829908 | 272 | 195 | 163 | 239 | 118 | 199 | 0.00027315 | 0.702 | 0.535 | 0.167 |
|  |  | 105076527 | + | 11895678 | 11895874 | 11898061 | 11898215 | 11894322 | 11894392 | 11898935 | 11899030 | 42 | 84 | 91 | 60 | 198 | 156 | 0.000283585 | 0.283 | 0.544 | -0.261 |
|  |  | 105076019 | + | 325092 | 325231 | 327199 | 327291 | 322571 | 322716 | 328098 | 328258 | 133 | 156 | 197 | 104 | 264 | 170 | 0.000401992 | 0.354 | 0.55 | -0.196 |
|  |  | 105076528 | - | 3440969 | 3441076 | 3443592 | 3443714 | 3440413 | 3440611 | 3443839 | 3443939 | 56 | 33 | 31 | 64 | 188 | 158 | 0.000614213 | 0.588 | 0.289 | 0.299 |
|  |  | 105078859 | - | 4951144 | 4951216 | 4955325 | 4955441 | 4947098 | 4947472 | 4962019 | 4962371 | 130 | 89 | 84 | 137 | 218 | 130 | 0.000811408 | 0.466 | 0.268 | 0.198 |
|  |  | 105073953 | + | 9714516 | 9714645 | 9714822 | 9714897 | 9714278 | 9714425 | 9715027 | 9715144 | 99 | 38 | 104 | 10 | 219 | 111 | 0.001418252 | 0.569 | 0.841 | -0.272 |
|  | WS | 105064785 | - | 3135861 | 3135990 | 3137974 | 3138170 | 3126623 | 3126842 | 3142400 | 3142540 | 65 | 638 | 304 | 514 | 315 | 242 | 0 | 0.073 | 0.312 | -0.239 |
|  |  | 105076629 | + | 28256828 | 28257033 | 28257187 | 28257399 | 28256260 | 28256519 | 28257486 | 28257608 | 1684 | 793 | 2355 | 2316 | 306 | 313 | 0 | 0.685 | 0.51 | 0.175 |
|  |  | 105081796 | + | 17927 | 18086 | 20643 | 20802 | 17409 | 17571 | 21148 | 21304 | 48 | 355 | 114 | 127 | 280 | 280 | 0 | 0.119 | 0.473 | -0.354 |
|  |  | 105079614 | + | 1530023 | 1530263 | 1535513 | 1535630 | 1527164 | 1527295 | 1535766 | 1535880 | 2635 | 461 | 2209 | 822 | 322 | 181 | 3.33E-16 | 0.763 | 0.602 | 0.161 |
|  |  | 105080211 | + | 1477690 | 1477792 | 1492452 | 1492705 | 1477080 | 1477154 | 1496972 | 1497211 | 133 | 82 | 27 | 133 | 122 | 306 | 2.11E-15 | 0.803 | 0.337 | 0.466 |
|  |  | 105072394 | - | 9637915 | 9638021 | 9639529 | 9639676 | 9636915 | 9637374 | 9639965 | 9640077 | 171 | 245 | 756 | 369 | 240 | 168 | 6.78E-14 | 0.328 | 0.589 | -0.261 |
|  |  | 105067286 | + | 6237618 | 6237736 | 6242011 | 6242200 | 6215071 | 6215175 | 6251617 | 6251754 | 288 | 136 | 77 | 154 | 179 | 267 | 5.33E-13 | 0.76 | 0.427 | 0.333 |
|  |  | 105081813 | - | 8331902 | 8332044 | 8336873 | 8337050 | 8328827 | 8329015 | 8339299 | 8339460 | 97 | 362 | 352 | 416 | 298 | 270 | 1.39E-12 | 0.195 | 0.434 | -0.239 |
|  |  | 105071291 | + | 7922877 | 7923079 | 7925420 | 7925635 | 7915872 | 7918079 | 7926713 | 7926866 | 379 | 149 | 404 | 442 | 323 | 336 | 2.07E-12 | 0.726 | 0.487 | 0.239 |
|  |  | 105072333 | + | 1953299 | 1953443 | 1953754 | 1953892 | 1952946 | 1953056 | 1954138 | 1954228 | 213 | 553 | 846 | 945 | 186 | 178 | 4.53E-12 | 0.269 | 0.461 | -0.192 |
|  |  | 105078518 | + | 1545079 | 1545152 | 1548626 | 1548765 | 1532485 | 1532742 | 1563108 | 1563689 | 43 | 915 | 156 | 806 | 132 | 264 | 8.87E-11 | 0.086 | 0.279 | -0.193 |
|  |  | 105065267 | + | 901541 | 901657 | 903312 | 903498 | 899230 | 899357 | 904804 | 905008 | 238 | 1039 | 632 | 1332 | 203 | 292 | 2.85E-09 | 0.248 | 0.406 | -0.158 |
|  |  | 105078429 | + | 1101209 | 1101324 | 1102031 | 1102133 | 1100571 | 1100735 | 1102264 | 1102412 | 68 | 347 | 219 | 356 | 216 | 190 | 7.00E-09 | 0.147 | 0.351 | -0.204 |
|  |  | 105067249 | - | 161445 | 161551 | 163344 | 163477 | 160800 | 161112 | 164310 | 164437 | 260 | 461 | 674 | 564 | 237 | 183 | 1.26E-08 | 0.303 | 0.48 | -0.177 |
|  |  | 105067938 | - | 12627298 | 12627434 | 12630435 | 12630641 | 12615194 | 12615463 | 12633137 | 12633249 | 354 | 485 | 942 | 654 | 297 | 228 | 2.78E-08 | 0.359 | 0.525 | -0.166 |
|  |  | 105079769 | - | 1721785 | 1721941 | 1723035 | 1723178 | 1721040 | 1721574 | 1723905 | 1724023 | 584 | 1340 | 1172 | 1562 | 246 | 253 | 1.23E-07 | 0.309 | 0.436 | -0.127 |
|  |  | 105073938 | + | 6969258 | 6969396 | 6992857 | 6992948 | 6955892 | 6956052 | 6994183 | 6994361 | 102 | 393 | 272 | 394 | 262 | 168 | 2.00E-07 | 0.143 | 0.307 | -0.164 |
|  |  | 105070713 | + | 1563032 | 1563221 | 1563342 | 1563418 | 1562342 | 1562498 | 1563692 | 1563857 | 55 | 234 | 108 | 117 | 310 | 138 | 2.55E-07 | 0.095 | 0.291 | -0.196 |
|  |  | 105069531 | + | 2014672 | 2015002 | 2018292 | 2018568 | 2011265 | 2011413 | 2022580 | 2022685 | 81 | 124 | 198 | 97 | 414 | 360 | 5.04E-07 | 0.362 | 0.64 | -0.278 |
|  |  | 105072021 | + | 4531578 | 4531746 | 4532140 | 4532317 | 4530569 | 4530674 | 4538334 | 4538425 | 226 | 33 | 454 | 221 | 201 | 210 | 8.27E-07 | 0.877 | 0.682 | 0.195 |
|  |  | 105075892 | - | 394905 | 395096 | 395211 | 395292 | 394398 | 394659 | 395474 | 395633 | 32 | 316 | 70 | 181 | 148 | 312 | 1.04E-06 | 0.176 | 0.449 | -0.273 |
|  |  | 105083392 | + | 11223074 | 11223216 | 11227101 | 11227243 | 11220790 | 11220941 | 11227946 | 11228054 | 48 | 80 | 226 | 109 | 236 | 236 | 1.29E-06 | 0.375 | 0.675 | -0.3 |
|  |  | 105071661 | + | 222851 | 223160 | 233438 | 233684 | 218517 | 218630 | 235102 | 235225 | 186 | 122 | 318 | 494 | 382 | 319 | 1.35E-06 | 0.56 | 0.35 | 0.21 |
|  |  | 105077612 | - | 1308105 | 1308181 | 1335931 | 1336135 | 1286039 | 1286149 | 1338563 | 1341012 | 767 | 28 | 402 | 62 | 293 | 106 | 1.42E-06 | 0.908 | 0.701 | 0.207 |
|  |  | 105083714 | + | 5585446 | 5585580 | 5592974 | 5593217 | 5574461 | 5574584 | 5593472 | 5593583 | 66 | 162 | 302 | 287 | 204 | 314 | 5.25E-06 | 0.385 | 0.618 | -0.233 |
|  |  | 105077298 | - | 32709739 | 32709882 | 32711119 | 32711227 | 32706504 | 32706678 | 32713735 | 32713828 | 6 | 59 | 60 | 81 | 153 | 221 | 9.11E-06 | 0.128 | 0.517 | -0.389 |
|  |  | 105072196 | - | 6097581 | 6097628 | 6104822 | 6104956 | 6095833 | 6095947 | 6112746 | 6112887 | 71 | 32 | 412 | 46 | 225 | 51 | 1.29E-05 | 0.335 | 0.67 | -0.335 |
|  |  | 105067762 | + | 148323 | 148449 | 148740 | 149127 | 148104 | 148232 | 149450 | 149544 | 9 | 77 | 30 | 36 | 176 | 446 | 1.32E-05 | 0.229 | 0.679 | -0.45 |
|  |  | 105083805 | + | 3576981 | 3577126 | 3585788 | 3585953 | 3576480 | 3576518 | 3592442 | 3592540 | 0 | 16 | 15 | 6 | 122 | 138 | 1.38E-05 | 0 | 0.739 | -0.739 |
|  |  | 105083322 | + | 2949496 | 2949595 | 2952170 | 2952361 | 2949011 | 2949245 | 2965540 | 2965640 | 1 | 51 | 25 | 48 | 142 | 270 | 1.57E-05 | 0.036 | 0.498 | -0.462 |
|  |  | 105062184 | - | 631572 | 631699 | 632636 | 632746 | 630610 | 631105 | 633837 | 634004 | 30 | 353 | 150 | 542 | 206 | 240 | 2.30E-05 | 0.09 | 0.244 | -0.154 |
|  |  | 105064863 | + | 2699168 | 2699342 | 2699426 | 2699586 | 2698804 | 2698912 | 2699809 | 2699901 | 3600 | 7647 | 5794 | 8823 | 211 | 197 | 2.47E-05 | 0.305 | 0.38 | -0.075 |
|  |  | 105083014 | + | 3524246 | 3524345 | 3531409 | 3531526 | 3508886 | 3509148 | 3533425 | 3533583 | 8 | 63 | 69 | 98 | 184 | 220 | 4.31E-05 | 0.132 | 0.457 | -0.325 |
|  |  | 105063841 | - | 3750056 | 3750191 | 3750368 | 3750503 | 3748220 | 3748293 | 3752531 | 3752648 | 9 | 147 | 54 | 154 | 162 | 162 | 4.77E-05 | 0.058 | 0.26 | -0.202 |
|  |  | 105069531 | + | 2014672 | 2015002 | 2018292 | 2018568 | 2011265 | 2011413 | 2019085 | 2019424 | 77 | 117 | 202 | 119 | 451 | 397 | 5.58E-05 | 0.367 | 0.599 | -0.232 |
|  |  | 105066281 | + | 18803864 | 18804026 | 18809146 | 18809341 | 18777927 | 18778054 | 18818604 | 18818710 | 168 | 62 | 184 | 173 | 232 | 265 | 6.27E-05 | 0.756 | 0.549 | 0.207 |
|  |  | 105078825 | - | 7171068 | 7171166 | 7181103 | 7181200 | 7170551 | 7170829 | 7181864 | 7181993 | 189 | 90 | 151 | 171 | 167 | 169 | 8.60E-05 | 0.68 | 0.472 | 0.208 |
|  |  | 105070935 | + | 6054228 | 6054315 | 6075347 | 6075554 | 6052239 | 6052345 | 6076442 | 6076631 | 25 | 31 | 11 | 82 | 124 | 292 | 9.20E-05 | 0.655 | 0.24 | 0.415 |
|  |  | 105067286 | + | 6035758 | 6035863 | 6078028 | 6078142 | 5995669 | 5996011 | 6114606 | 6114750 | 137 | 106 | 198 | 342 | 196 | 214 | 0.00012404 | 0.585 | 0.387 | 0.198 |
|  |  | 105082302 | - | 2995443 | 2995549 | 2997683 | 2998092 | 2993672 | 2993808 | 3014982 | 3015045 | 782 | 111 | 1154 | 314 | 445 | 113 | 0.000127833 | 0.641 | 0.483 | 0.158 |
|  |  | 105063767 | + | 154590 | 154814 | 156284 | 156456 | 153030 | 153219 | 157610 | 157801 | 147 | 182 | 513 | 314 | 345 | 293 | 0.000131332 | 0.407 | 0.581 | -0.174 |
|  |  | 105081455 | - | 88878 | 89017 | 89619 | 89978 | 88240 | 88420 | 90664 | 90770 | 413 | 112 | 203 | 122 | 444 | 228 | 0.000157797 | 0.654 | 0.461 | 0.193 |
|  |  | 105066616 | + | 5163339 | 5163493 | 5170657 | 5170693 | 5160983 | 5161241 | 5171388 | 5171483 | 257 | 21 | 593 | 8 | 228 | 29 | 0.000168516 | 0.609 | 0.904 | -0.295 |
|  |  | 105083817 | + | 6297284 | 6297443 | 6299333 | 6299508 | 6294498 | 6294639 | 6303021 | 6303208 | 97 | 51 | 219 | 287 | 279 | 295 | 0.00020602 | 0.668 | 0.447 | 0.221 |
|  |  | 105077089 | - | 3395048 | 3395143 | 3420674 | 3420837 | 3369558 | 3369676 | 3432950 | 3433029 | 6 | 26 | 88 | 59 | 197 | 89 | 0.000220074 | 0.094 | 0.403 | -0.309 |
|  |  | 105070796 | - | 714573 | 714747 | 716346 | 716479 | 713710 | 713905 | 716548 | 716885 | 108 | 29 | 85 | 74 | 252 | 295 | 0.000249571 | 0.813 | 0.573 | 0.24 |
|  |  | 105064785 | - | 3071938 | 3072055 | 3075881 | 3076091 | 3069929 | 3070325 | 3094946 | 3095078 | 165 | 58 | 225 | 186 | 321 | 210 | 0.000251097 | 0.65 | 0.442 | 0.208 |
|  |  | 105081422 | + | 3585601 | 3585714 | 3591906 | 3592034 | 3577622 | 3577786 | 3601208 | 3601363 | 7 | 56 | 27 | 33 | 212 | 242 | 0.000251205 | 0.125 | 0.483 | -0.358 |
|  |  | 105080953 | + | 189821 | 190001 | 204015 | 204195 | 189040 | 189193 | 213913 | 214011 | 19 | 39 | 75 | 37 | 257 | 257 | 0.000297268 | 0.328 | 0.67 | -0.342 |
|  |  | 105069868 | + | 314895 | 314980 | 315216 | 315384 | 314707 | 314821 | 315907 | 316098 | 215 | 199 | 251 | 102 | 128 | 261 | 0.00032762 | 0.688 | 0.834 | -0.146 |
|  |  | 105072346 | + | 3253114 | 3253237 | 3329814 | 3330507 | 3224196 | 3224309 | 3415769 | 3415896 | 256 | 967 | 147 | 1015 | 188 | 770 | 0.000332923 | 0.52 | 0.372 | 0.148 |
|  |  | 105068762 | - | 567069 | 567214 | 587025 | 587571 | 565468 | 565592 | 588636 | 588711 | 697 | 157 | 749 | 306 | 582 | 185 | 0.000342182 | 0.585 | 0.438 | 0.147 |
|  |  | 105063300 | - | 1534533 | 1534638 | 1541613 | 1541751 | 1532358 | 1532944 | 1547100 | 1547310 | 476 | 150 | 624 | 365 | 262 | 196 | 0.000342558 | 0.704 | 0.561 | 0.143 |
|  |  | 105069597 | - | 121599 | 121832 | 137006 | 137186 | 116393 | 116523 | 145608 | 145800 | 52 | 15 | 31 | 41 | 289 | 342 | 0.000388347 | 0.804 | 0.472 | 0.332 |
|  |  | 105080727 | + | 2309274 | 2309372 | 2310242 | 2310357 | 2307433 | 2307655 | 2312878 | 2312986 | 88 | 156 | 262 | 216 | 148 | 182 | 0.000442299 | 0.41 | 0.599 | -0.189 |
|  |  | 105081865 | - | 13765700 | 13765834 | 13773914 | 13774012 | 13744492 | 13745488 | 13777407 | 13777605 | 33 | 21 | 97 | 216 | 182 | 254 | 0.000523706 | 0.687 | 0.385 | 0.302 |
|  |  | 105083738 | - | 5698903 | 5699116 | 5701442 | 5701593 | 5692617 | 5692831 | 5702560 | 5702772 | 60 | 278 | 173 | 356 | 272 | 334 | 0.000549091 | 0.21 | 0.374 | -0.164 |
|  |  | 105073444 | - | 1786820 | 1787017 | 1792293 | 1792486 | 1782643 | 1784406 | 1819268 | 1820604 | 266 | 218 | 431 | 630 | 314 | 318 | 0.000643177 | 0.553 | 0.409 | 0.144 |
|  |  | 105065263 | + | 1579693 | 1579808 | 1580767 | 1580860 | 1579409 | 1579541 | 1581236 | 1581341 | 341 | 6 | 807 | 91 | 169 | 125 | 0.000834256 | 0.977 | 0.868 | 0.109 |
|  |  | 105076002 | + | 186366 | 186450 | 195419 | 195527 | 184581 | 184755 | 196020 | 196207 | 28 | 13 | 0 | 8 | 154 | 202 | 0.000836602 | 0.739 | 0 | 0.739 |
|  |  | 105076292 | + | 6885385 | 6885503 | 6885781 | 6885909 | 6885097 | 6885170 | 6886303 | 6886505 | 12 | 3 | 12 | 34 | 153 | 173 | 0.001035527 | 0.819 | 0.285 | 0.534 |
|  |  | 105064679 | + | 964277 | 964461 | 966595 | 966764 | 963958 | 964049 | 967235 | 967394 | 60 | 157 | 121 | 135 | 254 | 239 | 0.001103945 | 0.264 | 0.458 | -0.194 |
|  |  | 105072300 | - | 34202 | 34375 | 34648 | 34782 | 33961 | 34076 | 35110 | 35324 | 101 | 381 | 137 | 248 | 227 | 267 | 0.001175781 | 0.238 | 0.394 | -0.156 |
|  |  | 105081726 | + | 3240525 | 3240620 | 3279398 | 3279464 | 3235452 | 3235585 | 3290632 | 3290773 | 16 | 34 | 24 | 9 | 166 | 108 | 0.001612595 | 0.234 | 0.634 | -0.4 |
|  |  | 105064420 | - | 11532868 | 11532997 | 11534611 | 11534731 | 11531685 | 11531778 | 11541497 | 11543046 | 168 | 168 | 541 | 289 | 177 | 195 | 0.001620027 | 0.524 | 0.673 | -0.149 |
|  |  | 105073945 | - | 9303304 | 9303466 | 9304857 | 9304933 | 9302894 | 9302998 | 9307103 | 9307253 | 24 | 189 | 220 | 708 | 100 | 245 | 0.00162348 | 0.237 | 0.432 | -0.195 |
|  |  | 105083714 | + | 5574461 | 5574584 | 5585446 | 5585580 | 5563131 | 5563298 | 5592974 | 5593217 | 42 | 74 | 245 | 180 | 232 | 254 | 0.001699315 | 0.383 | 0.598 | -0.215 |
|  |  | 105068777 | - | 849657 | 849862 | 864954 | 865147 | 848647 | 848799 | 865389 | 865777 | 262 | 1005 | 188 | 392 | 314 | 326 | 0.002106197 | 0.213 | 0.332 | -0.119 |
|  |  | 105064420 | - | 11532868 | 11533078 | 11534611 | 11534731 | 11531685 | 11531778 | 11541497 | 11543046 | 168 | 323 | 541 | 604 | 177 | 282 | 0.002124677 | 0.453 | 0.588 | -0.135 |
|  |  | 105071552 | - | 3877368 | 3877582 | 3883797 | 3883946 | 3876911 | 3877094 | 3885178 | 3885333 | 226 | 68 | 227 | 149 | 270 | 335 | 0.002153562 | 0.805 | 0.654 | 0.151 |
|  |  | 105072175 | - | 5166855 | 5167078 | 5167485 | 5167595 | 5166396 | 5166527 | 5168463 | 5168594 | 8 | 87 | 24 | 56 | 184 | 322 | 0.002313223 | 0.139 | 0.429 | -0.29 |
|  |  | 105063879 | - | 5920299 | 5920456 | 5920550 | 5920638 | 5913199 | 5913307 | 5923367 | 5923442 | 68 | 47 | 149 | 251 | 61 | 177 | 0.00234389 | 0.808 | 0.633 | 0.175 |
|  |  | 105082307 | - | 3170877 | 3171066 | 3171276 | 3171444 | 3169721 | 3170173 | 3171613 | 3171995 | 30 | 60 | 69 | 47 | 289 | 310 | 0.002545425 | 0.349 | 0.612 | -0.263 |
|  |  | 105083657 | + | 1969879 | 1969981 | 1977608 | 1977716 | 1969469 | 1969615 | 1983782 | 1983902 | 164 | 20 | 120 | 48 | 168 | 180 | 0.002926435 | 0.898 | 0.728 | 0.17 |
|  |  | 105075018 | + | 2340353 | 2341133 | 2349422 | 2349545 | 2320551 | 2320760 | 2355038 | 2355786 | 638 | 43 | 330 | 54 | 901 | 232 | 0.002960994 | 0.793 | 0.611 | 0.182 |
|  |  | 105071393 | + | 2618437 | 2618538 | 2619093 | 2619231 | 2602802 | 2602884 | 2621814 | 2621990 | 15 | 364 | 45 | 286 | 128 | 202 | 0.002989352 | 0.061 | 0.199 | -0.138 |
|  |  | 105078304 | + | 5529214 | 5529292 | 5536180 | 5536234 | 5527191 | 5527293 | 5538224 | 5539027 | 15 | 42 | 43 | 31 | 102 | 54 | 0.003028366 | 0.159 | 0.423 | -0.264 |
|  |  | 105082159 | - | 26797966 | 26798013 | 26798214 | 26798308 | 26796974 | 26797189 | 26798593 | 26799046 | 30 | 24 | 117 | 27 | 174 | 80 | 0.003126071 | 0.365 | 0.666 | -0.301 |
|  |  | 105081656 | - | 4446264 | 4446393 | 4448720 | 4448821 | 4443491 | 4443657 | 4449994 | 4450058 | 6 | 4 | 6 | 55 | 110 | 166 | 0.003150565 | 0.694 | 0.141 | 0.553 |
|  |  | 105080725 | + | 2025132 | 2025254 | 2026258 | 2026472 | 2024672 | 2024837 | 2028856 | 2028960 | 156 | 86 | 244 | 268 | 192 | 297 | 0.003252756 | 0.737 | 0.585 | 0.152 |
|  |  | 105082972 | - | 2896384 | 2896504 | 2901678 | 2901786 | 2889354 | 2889487 | 2903305 | 2903399 | 4 | 48 | 53 | 121 | 145 | 169 | 0.003373198 | 0.089 | 0.338 | -0.249 |
|  |  | 105063944 | - | 4452335 | 4452603 | 4460216 | 4460514 | 4445981 | 4446315 | 4461562 | 4461670 | 245 | 52 | 285 | 133 | 385 | 355 | 0.003476274 | 0.813 | 0.664 | 0.149 |
|  |  | 105077455 | + | 39484827 | 39485000 | 39498818 | 39499004 | 39479219 | 39479655 | 39499687 | 39499834 | 6 | 36 | 30 | 36 | 294 | 307 | 0.003718114 | 0.148 | 0.465 | -0.317 |
|  |  | 105065705 | + | 7639285 | 7639351 | 7648973 | 7649141 | 7625982 | 7626131 | 7665139 | 7665323 | 3 | 70 | 14 | 44 | 118 | 289 | 0.003781806 | 0.095 | 0.438 | -0.343 |
|  |  | 105078862 | + | 4983240 | 4983407 | 5001525 | 5001679 | 4969080 | 4969998 | 5009209 | 5009455 | 9 | 45 | 23 | 25 | 288 | 275 | 0.004686265 | 0.16 | 0.468 | -0.308 |
|  |  | 105084143 | - | 1765914 | 1766262 | 1767563 | 1767684 | 1761294 | 1761576 | 1768177 | 1768367 | 82 | 187 | 52 | 255 | 228 | 469 | 0.005781496 | 0.474 | 0.296 | 0.178 |
|  |  | 105063022 | - | 1292034 | 1292163 | 1294866 | 1295013 | 1290997 | 1291093 | 1296335 | 1297724 | 40 | 1 | 32 | 12 | 224 | 198 | 0.006227438 | 0.972 | 0.702 | 0.27 |
| **AS category** | **Stress** | **GeneID** | **strand** | **longExonStart_0base** | **longExonEnd** | **shortES** | **shortEE** | **flankingES** | **flankingEE** | **IC_SAMPLE_1** | **SC_SAMPLE_1** | **IC_SAMPLE_2** | **SC_SAMPLE_2** | **IncFormLen** | **SkipFormLen** | **PValue** | **IncLevel1** | **IncLevel2** | **IncLevelDifference** |  |  |
| A5SS | SS | 105076998 | + | 11821971 | 11822139 | 11821971 | 11822127 | 11823911 | 11826253 | 1 | 14 | 9 | 6 | 140 | 135 | 0.005199042 | 0.064 | 0.591 | -0.527 |  |  |
|  |  | 105073907 | + | 1717611 | 1717704 | 1717611 | 1717700 | 1718894 | 1719018 | 11 | 5 | 4 | 18 | 68 | 64 | 0.005708687 | 0.674 | 0.173 | 0.501 |  |  |
|  |  | 105080193 | - | 1087639 | 1087816 | 1087657 | 1087816 | 1083779 | 1083989 | 3 | 20 | 13 | 10 | 146 | 135 | 0.006606353 | 0.122 | 0.546 | -0.424 |  |  |
|  |  | 105072380 | - | 9187639 | 9189180 | 9188617 | 9189180 | 9180495 | 9180601 | 348 | 18 | 520 | 6 | 1063 | 99 | 0.006982622 | 0.643 | 0.89 | -0.247 |  |  |
|  |  | 105078425 | - | 485797 | 485901 | 485821 | 485901 | 484752 | 484829 | 1 | 5 | 13 | 2 | 32 | 8 | 0.008747881 | 0.048 | 0.619 | -0.571 |  |  |
|  |  | 105079183 | + | 2163406 | 2163643 | 2163406 | 2163555 | 2165413 | 2165628 | 3 | 8 | 9 | 1 | 216 | 135 | 0.009229053 | 0.19 | 0.849 | -0.659 |  |  |
|  |  | 105074963 | - | 8631133 | 8631414 | 8631172 | 8631414 | 8629219 | 8629381 | 4 | 9 | 9 | 1 | 167 | 135 | 0.010559888 | 0.264 | 0.879 | -0.615 |  |  |
|  |  | 105083854 | - | 7408990 | 7409202 | 7409010 | 7409202 | 7405122 | 7405547 | 12 | 21 | 21 | 8 | 148 | 135 | 0.015549065 | 0.343 | 0.705 | -0.362 |  |  |
|  |  | 105072719 | + | 522562 | 522812 | 522562 | 522657 | 523530 | 523647 | 19 | 1 | 23 | 13 | 204 | 63 | 0.016834437 | 0.854 | 0.353 | 0.501 |  |  |
|  |  | 105078592 | + | 3835194 | 3835321 | 3835194 | 3835315 | 3837548 | 3837670 | 48 | 0 | 25 | 6 | 100 | 94 | 0.019118509 | 1 | 0.797 | 0.203 |  |  |
|  |  | 105080239 | - | 2415681 | 2415945 | 2415719 | 2415945 | 2402357 | 2402467 | 3 | 12 | 12 | 6 | 134 | 103 | 0.01966674 | 0.161 | 0.606 | -0.445 |  |  |
|  |  | 105076595 | - | 22991311 | 22991430 | 22991331 | 22991430 | 22990542 | 22990680 | 8 | 11 | 20 | 5 | 108 | 88 | 0.028348239 | 0.372 | 0.765 | -0.393 |  |  |
|  |  | 105063083 | + | 9058896 | 9059065 | 9058896 | 9059019 | 9059759 | 9059896 | 16 | 3 | 8 | 10 | 150 | 111 | 0.029286976 | 0.798 | 0.372 | 0.426 |  |  |
|  |  | 105079094 | + | 1942344 | 1942606 | 1942344 | 1942501 | 1943678 | 1943847 | 0 | 11 | 5 | 6 | 233 | 135 | 0.02985327 | 0 | 0.326 | -0.326 |  |  |
|  |  | 105064626 | - | 731367 | 731507 | 731373 | 731507 | 729963 | 730078 | 0 | 10 | 11 | 19 | 106 | 100 | 0.031032456 | 0 | 0.353 | -0.353 |  |  |
|  |  | 105067032 | - | 880994 | 881137 | 881006 | 881137 | 880562 | 880731 | 49 | 45 | 75 | 30 | 135 | 124 | 0.037813062 | 0.5 | 0.697 | -0.197 |  |  |
|  |  | 105080239 | - | 2415681 | 2415945 | 2415752 | 2415945 | 2402357 | 2402467 | 3 | 4 | 12 | 1 | 167 | 103 | 0.044846052 | 0.316 | 0.881 | -0.565 |  |  |
|  | WS | 105061635 | - | 14891190 | 14893485 | 14892987 | 14893485 | 14886267 | 14890193 | 2656 | 12 | 1163 | 34 | 1918 | 135 | 3.03E-06 | 0.94 | 0.707 | 0.233 |  |  |
|  |  | 105082092 | + | 21335483 | 21335657 | 21335483 | 21335571 | 21360184 | 21360506 | 109 | 24 | 294 | 7 | 160 | 81 | 1.09E-05 | 0.697 | 0.955 | -0.258 |  |  |
|  |  | 105064215 | + | 3546430 | 3548255 | 3546430 | 3548030 | 3548364 | 3548465 | 113 | 33 | 124 | 5 | 305 | 94 | 4.45E-05 | 0.513 | 0.884 | -0.371 |  |  |
|  |  | 105065987 | + | 850730 | 850901 | 850730 | 850880 | 851848 | 852009 | 50 | 7 | 28 | 30 | 149 | 135 | 5.25E-05 | 0.866 | 0.458 | 0.408 |  |  |
|  |  | 105079809 | - | 1191273 | 1191585 | 1191284 | 1191585 | 1190859 | 1190993 | 4 | 16 | 41 | 17 | 131 | 127 | 0.000417648 | 0.195 | 0.7 | -0.505 |  |  |
|  |  | 105066250 | + | 15461778 | 15462023 | 15461778 | 15461885 | 15463177 | 15463377 | 239 | 5 | 187 | 26 | 231 | 100 | 0.000899623 | 0.954 | 0.757 | 0.197 |  |  |
|  |  | 105064964 | + | 330840 | 331075 | 330840 | 330943 | 333694 | 334069 | 0 | 27 | 14 | 22 | 221 | 96 | 0.001316614 | 0 | 0.217 | -0.217 |  |  |
|  |  | 105068196 | - | 2273375 | 2273852 | 2273445 | 2273852 | 2273073 | 2273248 | 13 | 0 | 4 | 7 | 198 | 135 | 0.001432088 | 1 | 0.28 | 0.72 |  |  |
|  |  | 105066370 | - | 2377826 | 2378011 | 2377871 | 2378011 | 2377282 | 2377369 | 2 | 13 | 20 | 9 | 116 | 78 | 0.00143245 | 0.094 | 0.599 | -0.505 |  |  |
|  |  | 105064043 | + | 7368837 | 7369957 | 7368837 | 7369044 | 7370165 | 7371128 | 249 | 28 | 173 | 52 | 1034 | 135 | 0.00199603 | 0.537 | 0.303 | 0.234 |  |  |
|  |  | 105077726 | + | 6723724 | 6723960 | 6723724 | 6723915 | 6725162 | 6725297 | 3 | 11 | 23 | 7 | 166 | 128 | 0.002038334 | 0.174 | 0.717 | -0.543 |  |  |
|  |  | 105071538 | - | 2922299 | 2922420 | 2922305 | 2922420 | 2916804 | 2916892 | 14 | 2 | 42 | 54 | 60 | 54 | 0.003180914 | 0.863 | 0.412 | 0.451 |  |  |
|  |  | 105074711 | + | 13171219 | 13171367 | 13171219 | 13171363 | 13172253 | 13172398 | 273 | 148 | 348 | 95 | 135 | 135 | 0.004392808 | 0.648 | 0.786 | -0.138 |  |  |
|  |  | 105082187 | - | 27772640 | 27773033 | 27772698 | 27773033 | 27770213 | 27770452 | 3 | 4 | 16 | 0 | 186 | 135 | 0.006023289 | 0.352 | 1 | -0.648 |  |  |
|  |  | 105068895 | + | 162346 | 162725 | 162346 | 162452 | 162868 | 163030 | 64 | 6 | 65 | 26 | 358 | 99 | 0.006173421 | 0.747 | 0.409 | 0.338 |  |  |
|  |  | 105073927 | - | 5099586 | 5099728 | 5099595 | 5099728 | 5099094 | 5099343 | 2 | 18 | 31 | 35 | 135 | 126 | 0.006561751 | 0.094 | 0.453 | -0.359 |  |  |
|  |  | 105077205 | - | 18885000 | 18886333 | 18885009 | 18886333 | 18882061 | 18882646 | 3 | 6 | 33 | 5 | 137 | 135 | 0.007081427 | 0.33 | 0.867 | -0.537 |  |  |
|  |  | 105063949 | + | 4979536 | 4980462 | 4979536 | 4980033 | 4982045 | 4982128 | 50 | 3 | 27 | 11 | 491 | 76 | 0.008769333 | 0.721 | 0.275 | 0.446 |  |  |
|  |  | 105069980 | - | 12654 | 13090 | 12659 | 13090 | 12267 | 12470 | 0 | 34 | 12 | 45 | 135 | 135 | 0.009005495 | 0 | 0.211 | -0.211 |  |  |
|  |  | 105065844 | + | 1534538 | 1535301 | 1534538 | 1534687 | 1535920 | 1536378 | 245 | 90 | 218 | 158 | 735 | 135 | 0.009585881 | 0.333 | 0.202 | 0.131 |  |  |
|  |  | 105067471 | + | 22968503 | 22968617 | 22968503 | 22968607 | 22972894 | 22973110 | 48 | 0 | 34 | 8 | 107 | 97 | 0.010177735 | 1 | 0.794 | 0.206 |  |  |
|  |  | 105069076 | + | 1183741 | 1183918 | 1183741 | 1183841 | 1185841 | 1186171 | 22 | 7 | 28 | 38 | 163 | 93 | 0.010766133 | 0.642 | 0.296 | 0.346 |  |  |
|  |  | 105073318 | + | 3138382 | 3138645 | 3138382 | 3138575 | 3139061 | 3139109 | 4 | 1 | 2 | 23 | 104 | 41 | 0.01088909 | 0.612 | 0.033 | 0.579 |  |  |
|  |  | 105072829 | + | 6540445 | 6540772 | 6540445 | 6540702 | 6543419 | 6543586 | 9 | 0 | 10 | 10 | 198 | 135 | 0.011641794 | 1 | 0.405 | 0.595 |  |  |
|  |  | 105076595 | - | 22991311 | 22991430 | 22991331 | 22991430 | 22986855 | 22987009 | 24 | 0 | 63 | 18 | 112 | 92 | 0.011959433 | 1 | 0.742 | 0.258 |  |  |
|  |  | 105062803 | + | 469438 | 469933 | 469438 | 469840 | 470201 | 470343 | 85 | 16 | 80 | 2 | 221 | 135 | 0.015753144 | 0.764 | 0.961 | -0.197 |  |  |
|  |  | 105069383 | + | 1727005 | 1727160 | 1727005 | 1727155 | 1727994 | 1728106 | 1 | 6 | 4 | 0 | 105 | 105 | 0.016466621 | 0.143 | 1 | -0.857 |  |  |
|  |  | 105083771 | + | 2361880 | 2362036 | 2361880 | 2361957 | 2363943 | 2364093 | 17 | 2 | 3 | 5 | 142 | 70 | 0.019376836 | 0.807 | 0.228 | 0.579 |  |  |
|  |  | 105066790 | + | 2332703 | 2332838 | 2332703 | 2332832 | 2336039 | 2336177 | 81 | 48 | 40 | 55 | 124 | 118 | 0.020500528 | 0.616 | 0.409 | 0.207 |  |  |
|  |  | 105067832 | - | 2422979 | 2423351 | 2422988 | 2423351 | 2421952 | 2422033 | 2 | 3 | 11 | 0 | 76 | 74 | 0.023636615 | 0.394 | 1 | -0.606 |  |  |
|  |  | 105074341 | + | 4023481 | 4024054 | 4023481 | 4024042 | 4046447 | 4046642 | 5 | 1 | 0 | 4 | 140 | 135 | 0.024657869 | 0.828 | 0 | 0.828 |  |  |
|  |  | 105080744 | + | 4618422 | 4618629 | 4618422 | 4618587 | 4619316 | 4619418 | 2 | 9 | 23 | 14 | 130 | 95 | 0.025784709 | 0.14 | 0.546 | -0.406 |  |  |
|  |  | 105069180 | - | 7642163 | 7642295 | 7642169 | 7642295 | 7638131 | 7638340 | 18 | 0 | 15 | 6 | 125 | 119 | 0.029205172 | 1 | 0.704 | 0.296 |  |  |
|  |  | 105070873 | - | 1357745 | 1357972 | 1357790 | 1357972 | 1355258 | 1356026 | 13 | 14 | 19 | 4 | 173 | 135 | 0.030593402 | 0.42 | 0.788 | -0.368 |  |  |
|  |  | 105083288 | + | 719707 | 719871 | 719707 | 719866 | 721027 | 721189 | 33 | 4 | 26 | 15 | 135 | 135 | 0.031818196 | 0.892 | 0.634 | 0.258 |  |  |
|  |  | 105083805 | + | 3585788 | 3585953 | 3585788 | 3585918 | 3592442 | 3592540 | 16 | 2 | 5 | 6 | 107 | 79 | 0.033172828 | 0.855 | 0.381 | 0.474 |  |  |
|  |  | 105068370 | - | 2180256 | 2180506 | 2180295 | 2180506 | 2179889 | 2179973 | 2 | 0 | 0 | 6 | 109 | 77 | 0.038431287 | 1 | 0 | 1 |  |  |
|  |  | 105079962 | + | 12866826 | 12866988 | 12866826 | 12866961 | 12871202 | 12871253 | 3 | 11 | 7 | 3 | 57 | 37 | 0.045146473 | 0.15 | 0.602 | -0.452 |  |  |
|  |  | 105064806 | - | 1546168 | 1546587 | 1546237 | 1546587 | 1545517 | 1545665 | 10 | 3 | 32 | 0 | 197 | 135 | 0.046893189 | 0.696 | 1 | -0.304 |  |  |
|  |  | 105078810 | + | 5854644 | 5855040 | 5854644 | 5854732 | 5857803 | 5858228 | 109 | 0 | 111 | 6 | 375 | 81 | 0.048429075 | 1 | 0.8 | 0.2 |  |  |
| **AS category** | **Stress** | **GeneID** | **strand** | **longExonStart_0base** | **longExonEnd** | **shortES** | **shortEE** | **flankingES** | **flankingEE** | **IC_SAMPLE_1** | **SC_SAMPLE_1** | **IC_SAMPLE_2** | **SC_SAMPLE_2** | **IncFormLen** | **SkipFormLen** | **PValue** | **IncLevel1** | **IncLevel2** | **IncLevelDifference** |  |  |
| A3SS | SS | 105065903 | - | 1134979 | 1135193 | 1134979 | 1135190 | 1138268 | 1138500 | 1 | 24 | 14 | 9 | 135 | 135 | 6.80E-05 | 0.04 | 0.609 | -0.569 |  |  |
|  |  | 105076162 | + | 2386832 | 2388749 | 2387086 | 2388749 | 2365985 | 2366158 | 10 | 16 | 14 | 0 | 375 | 135 | 0.00019363 | 0.184 | 1 | -0.816 |  |  |
|  |  | 105079018 | - | 7339389 | 7339942 | 7339389 | 7339873 | 7340396 | 7340474 | 14 | 27 | 0 | 44 | 133 | 71 | 0.000407852 | 0.217 | 0 | 0.217 |  |  |
|  |  | 105073791 | + | 310314 | 311032 | 310772 | 311032 | 309303 | 309796 | 159 | 81 | 73 | 94 | 579 | 135 | 0.001809969 | 0.314 | 0.153 | 0.161 |  |  |
|  |  | 105083094 | + | 3925356 | 3925493 | 3925359 | 3925493 | 3918778 | 3918927 | 52 | 2 | 43 | 17 | 130 | 127 | 0.002908942 | 0.962 | 0.712 | 0.25 |  |  |
|  |  | 105062454 | - | 120723 | 120878 | 120723 | 120875 | 120976 | 121230 | 30 | 38 | 20 | 4 | 135 | 135 | 0.003072152 | 0.441 | 0.833 | -0.392 |  |  |
|  |  | 105065675 | + | 5605348 | 5605523 | 5605351 | 5605523 | 5605068 | 5605268 | 33 | 11 | 22 | 32 | 135 | 135 | 0.003658848 | 0.75 | 0.407 | 0.343 |  |  |
|  |  | 105079018 | - | 7339389 | 7339942 | 7339389 | 7339873 | 7350850 | 7350973 | 22 | 65 | 2 | 66 | 178 | 116 | 0.003800132 | 0.181 | 0.019 | 0.162 |  |  |
|  |  | 105069868 | + | 315066 | 315384 | 315093 | 315384 | 314895 | 314980 | 19 | 2 | 6 | 9 | 98 | 78 | 0.004279378 | 0.883 | 0.347 | 0.536 |  |  |
|  |  | 105075433 | - | 977081 | 977306 | 977081 | 977303 | 982630 | 982759 | 12 | 14 | 2 | 23 | 122 | 122 | 0.007466534 | 0.462 | 0.08 | 0.382 |  |  |
|  |  | 105070205 | - | 1101522 | 1101712 | 1101522 | 1101707 | 1108017 | 1108188 | 23 | 12 | 36 | 2 | 135 | 135 | 0.008446329 | 0.657 | 0.947 | -0.29 |  |  |
|  |  | 105078760 | - | 1183408 | 1186446 | 1183408 | 1186382 | 1189421 | 1189625 | 20 | 5 | 11 | 17 | 192 | 135 | 0.008645195 | 0.738 | 0.313 | 0.425 |  |  |
|  |  | 105079157 | + | 7995885 | 7996089 | 7995908 | 7996089 | 7995569 | 7995652 | 12 | 2 | 4 | 9 | 92 | 76 | 0.009950392 | 0.832 | 0.269 | 0.563 |  |  |
|  |  | 105072812 | - | 5981834 | 5981978 | 5981834 | 5981975 | 5982443 | 5982639 | 214 | 212 | 145 | 252 | 135 | 134 | 0.011773167 | 0.5 | 0.364 | 0.136 |  |  |
|  |  | 105066447 | + | 2000315 | 2000533 | 2000327 | 2000533 | 1999268 | 1999384 | 12 | 58 | 0 | 48 | 114 | 109 | 0.011993359 | 0.165 | 0 | 0.165 |  |  |
|  |  | 105066743 | - | 533755 | 534184 | 533755 | 534181 | 535277 | 535964 | 2 | 8 | 10 | 3 | 135 | 135 | 0.017188357 | 0.2 | 0.769 | -0.569 |  |  |
|  |  | 105069804 | + | 1304063 | 1304236 | 1304066 | 1304236 | 1303648 | 1303856 | 60 | 84 | 97 | 64 | 135 | 135 | 0.018106198 | 0.417 | 0.602 | -0.185 |  |  |
|  |  | 105077195 | + | 17711631 | 17712677 | 17711664 | 17712677 | 17707273 | 17707458 | 77 | 17 | 82 | 48 | 161 | 135 | 0.019129548 | 0.792 | 0.589 | 0.203 |  |  |
|  |  | 105072878 | + | 628946 | 629132 | 628952 | 629132 | 628056 | 628179 | 15 | 60 | 40 | 56 | 116 | 116 | 0.019478831 | 0.2 | 0.417 | -0.217 |  |  |
|  |  | 105081132 | - | 498302 | 498485 | 498302 | 498455 | 499425 | 499623 | 8 | 18 | 23 | 12 | 158 | 135 | 0.022732552 | 0.275 | 0.621 | -0.346 |  |  |
|  |  | 105081440 | - | 987168 | 987303 | 987168 | 987300 | 989728 | 989876 | 4 | 0 | 0 | 3 | 128 | 125 | 0.02665525 | 1 | 0 | 1 |  |  |
|  |  | 105083488 | + | 4888379 | 4888519 | 4888400 | 4888519 | 4882909 | 4883084 | 10 | 30 | 2 | 53 | 133 | 112 | 0.027008384 | 0.219 | 0.031 | 0.188 |  |  |
|  |  | 105065204 | - | 2696837 | 2696968 | 2696837 | 2696947 | 2697576 | 2697682 | 139 | 22 | 202 | 7 | 88 | 67 | 0.027452141 | 0.828 | 0.956 | -0.128 |  |  |
|  |  | 105068073 | - | 5651155 | 5651310 | 5651155 | 5651307 | 5662211 | 5662358 | 10 | 0 | 2 | 3 | 135 | 135 | 0.027865803 | 1 | 0.4 | 0.6 |  |  |
|  |  | 105073493 | - | 851889 | 852146 | 851889 | 852143 | 852617 | 852785 | 67 | 26 | 85 | 10 | 135 | 135 | 0.029837477 | 0.72 | 0.895 | -0.175 |  |  |
|  |  | 105080659 | - | 7290326 | 7290639 | 7290326 | 7290636 | 7291919 | 7292024 | 69 | 50 | 48 | 75 | 98 | 98 | 0.030475267 | 0.58 | 0.39 | 0.19 |  |  |
|  |  | 105069345 | - | 1649586 | 1649718 | 1649586 | 1649715 | 1650121 | 1650263 | 15 | 21 | 39 | 17 | 125 | 122 | 0.031970789 | 0.411 | 0.691 | -0.28 |  |  |
|  |  | 105074766 | - | 3140415 | 3140622 | 3140415 | 3140563 | 3141238 | 3141622 | 10 | 0 | 11 | 7 | 187 | 135 | 0.032152498 | 1 | 0.531 | 0.469 |  |  |
|  |  | 105069196 | - | 7925596 | 7925698 | 7925596 | 7925695 | 7927094 | 7927349 | 9 | 8 | 1 | 10 | 95 | 92 | 0.036939176 | 0.521 | 0.088 | 0.433 |  |  |
|  |  | 105078564 | + | 1955318 | 1955597 | 1955399 | 1955597 | 1952756 | 1952922 | 72 | 6 | 47 | 15 | 209 | 135 | 0.04008547 | 0.886 | 0.669 | 0.217 |  |  |
|  |  | 105080748 | - | 5059683 | 5059855 | 5059683 | 5059852 | 5059938 | 5060202 | 16 | 8 | 10 | 20 | 135 | 135 | 0.042495403 | 0.667 | 0.333 | 0.334 |  |  |
|  |  | 105063249 | + | 2295315 | 2295480 | 2295318 | 2295480 | 2294526 | 2294744 | 13 | 4 | 28 | 0 | 135 | 135 | 0.047149786 | 0.765 | 1 | -0.235 |  |  |
|  |  | 105066934 | - | 441549 | 442197 | 441549 | 442177 | 442939 | 443025 | 4 | 10 | 0 | 20 | 92 | 79 | 0.049499279 | 0.256 | 0 | 0.256 |  |  |
|  | WS | 105079018 | - | 7339389 | 7339942 | 7339389 | 7339873 | 7350850 | 7350973 | 31 | 35 | 2 | 66 | 178 | 116 | 6.28E-08 | 0.366 | 0.019 | 0.347 |  |  |
|  |  | 105064993 | - | 3756203 | 3756385 | 3756203 | 3756314 | 3756929 | 3756993 | 234 | 80 | 83 | 83 | 90 | 26 | 2.31E-05 | 0.458 | 0.224 | 0.234 |  |  |
|  |  | 105083566 | - | 18390382 | 18390666 | 18390382 | 18390552 | 18391700 | 18391827 | 19 | 12 | 0 | 18 | 227 | 120 | 2.60E-05 | 0.456 | 0 | 0.456 |  |  |
|  |  | 105074785 | - | 3437721 | 3438665 | 3437721 | 3438569 | 3444168 | 3444304 | 14 | 48 | 63 | 46 | 218 | 129 | 0.00014516 | 0.147 | 0.448 | -0.301 |  |  |
|  |  | 105083395 | - | 11379158 | 11379437 | 11379158 | 11379434 | 11385340 | 11385492 | 0 | 11 | 10 | 3 | 135 | 135 | 0.000246035 | 0 | 0.769 | -0.769 |  |  |
|  |  | 105081550 | + | 4203358 | 4203502 | 4203427 | 4203502 | 4201965 | 4202055 | 1 | 12 | 19 | 6 | 83 | 16 | 0.000381456 | 0.016 | 0.379 | -0.363 |  |  |
|  |  | 105066447 | + | 2000315 | 2000533 | 2000327 | 2000533 | 1999268 | 1999384 | 42 | 156 | 0 | 48 | 114 | 109 | 0.000425494 | 0.205 | 0 | 0.205 |  |  |
|  |  | 105068670 | - | 2169368 | 2169872 | 2169368 | 2169824 | 2180992 | 2181147 | 4 | 12 | 21 | 4 | 176 | 135 | 0.000658907 | 0.204 | 0.801 | -0.597 |  |  |
|  |  | 105075433 | - | 977081 | 977306 | 977081 | 977303 | 982630 | 982759 | 21 | 19 | 2 | 23 | 122 | 122 | 0.000699556 | 0.525 | 0.08 | 0.445 |  |  |
|  |  | 105067682 | - | 10582161 | 10582391 | 10582161 | 10582388 | 10584110 | 10584247 | 42 | 2 | 36 | 20 | 130 | 130 | 0.000828479 | 0.955 | 0.643 | 0.312 |  |  |
|  |  | 105064189 | - | 2252718 | 2253362 | 2252718 | 2252952 | 2253754 | 2253934 | 12 | 1 | 11 | 22 | 531 | 135 | 0.001272443 | 0.753 | 0.113 | 0.64 |  |  |
|  |  | 105069868 | + | 315907 | 316098 | 315910 | 316098 | 315066 | 315384 | 160 | 95 | 77 | 105 | 135 | 135 | 0.001429067 | 0.627 | 0.423 | 0.204 |  |  |
|  |  | 105064993 | - | 3756203 | 3756408 | 3756203 | 3756314 | 3756929 | 3756993 | 154 | 80 | 60 | 83 | 113 | 26 | 0.00148043 | 0.307 | 0.143 | 0.164 |  |  |
|  |  | 105079798 | - | 12328 | 12466 | 12328 | 12463 | 15719 | 15859 | 36 | 0 | 36 | 12 | 129 | 126 | 0.002658888 | 1 | 0.746 | 0.254 |  |  |
|  |  | 105073493 | - | 851889 | 852146 | 851889 | 852143 | 852617 | 852785 | 77 | 37 | 85 | 10 | 135 | 135 | 0.002664607 | 0.675 | 0.895 | -0.22 |  |  |
|  |  | 105080897 | - | 24452 | 24543 | 24452 | 24539 | 25397 | 25589 | 2 | 9 | 20 | 6 | 84 | 80 | 0.003219905 | 0.175 | 0.76 | -0.585 |  |  |
|  |  | 105079164 | - | 833803 | 833907 | 833803 | 833895 | 834065 | 834180 | 16 | 9 | 56 | 146 | 70 | 58 | 0.003454764 | 0.596 | 0.241 | 0.355 |  |  |
|  |  | 105076654 | + | 30238785 | 30239142 | 30238824 | 30239142 | 30233875 | 30234024 | 5 | 14 | 21 | 7 | 167 | 135 | 0.003563478 | 0.224 | 0.708 | -0.484 |  |  |
|  |  | 105076223 | + | 4399554 | 4399788 | 4399662 | 4399788 | 4398761 | 4398874 | 55 | 22 | 144 | 16 | 191 | 90 | 0.004568335 | 0.541 | 0.809 | -0.268 |  |  |
|  |  | 105075887 | + | 334531 | 334680 | 334534 | 334680 | 334224 | 334424 | 32 | 11 | 15 | 24 | 135 | 135 | 0.00495441 | 0.744 | 0.385 | 0.359 |  |  |
|  |  | 105075369 | + | 9371701 | 9371786 | 9371704 | 9371786 | 9364993 | 9365071 | 6 | 0 | 0 | 4 | 14 | 11 | 0.0052014 | 1 | 0 | 1 |  |  |
|  |  | 105065479 | - | 1122984 | 1123581 | 1122984 | 1123578 | 1127936 | 1128040 | 0 | 3 | 8 | 0 | 97 | 97 | 0.007284579 | 0 | 1 | -1 |  |  |
|  |  | 105069808 | - | 2371412 | 2371566 | 2371412 | 2371485 | 2373594 | 2373751 | 154 | 68 | 220 | 44 | 140 | 66 | 0.008025338 | 0.516 | 0.702 | -0.186 |  |  |
|  |  | 105071763 | + | 1581297 | 1581525 | 1581431 | 1581525 | 1579360 | 1579549 | 28 | 8 | 30 | 0 | 214 | 87 | 0.008630139 | 0.587 | 1 | -0.413 |  |  |
|  |  | 105076451 | - | 1350415 | 1350495 | 1350415 | 1350490 | 1351249 | 1351530 | 55 | 11 | 54 | 0 | 73 | 68 | 0.009076746 | 0.823 | 1 | -0.177 |  |  |
|  |  | 105071052 | + | 16257076 | 16257246 | 16257088 | 16257246 | 16256893 | 16256993 | 103 | 191 | 100 | 371 | 98 | 93 | 0.009446607 | 0.339 | 0.204 | 0.135 |  |  |
|  |  | 105071336 | - | 77141 | 77262 | 77141 | 77259 | 77886 | 78012 | 24 | 3 | 30 | 23 | 98 | 95 | 0.010075428 | 0.886 | 0.558 | 0.328 |  |  |
|  |  | 105081674 | + | 1203948 | 1205456 | 1203966 | 1205456 | 1203303 | 1203362 | 5 | 23 | 8 | 4 | 63 | 52 | 0.011639713 | 0.152 | 0.623 | -0.471 |  |  |
|  |  | 105080459 | - | 3518360 | 3518513 | 3518360 | 3518486 | 3520343 | 3520692 | 4 | 0 | 1 | 7 | 139 | 119 | 0.011724792 | 1 | 0.109 | 0.891 |  |  |
|  |  | 105082033 | - | 7419963 | 7420342 | 7419963 | 7420039 | 7422706 | 7422805 | 540 | 3 | 454 | 15 | 315 | 26 | 0.011926365 | 0.937 | 0.714 | 0.223 |  |  |
|  |  | 105082109 | - | 22530058 | 22530245 | 22530058 | 22530239 | 22530549 | 22530745 | 105 | 28 | 161 | 11 | 135 | 135 | 0.0136008 | 0.789 | 0.936 | -0.147 |  |  |
|  |  | 105069808 | - | 2371412 | 2371566 | 2371412 | 2371485 | 2373591 | 2373751 | 10 | 10 | 39 | 7 | 140 | 66 | 0.013854389 | 0.32 | 0.724 | -0.404 |  |  |
|  |  | 105065014 | + | 5160135 | 5160234 | 5160138 | 5160234 | 5159751 | 5159872 | 8 | 1 | 3 | 8 | 71 | 68 | 0.014020359 | 0.885 | 0.264 | 0.621 |  |  |
|  |  | 105074700 | - | 11637333 | 11637610 | 11637333 | 11637607 | 11638101 | 11638134 | 1 | 4 | 24 | 4 | 26 | 26 | 0.014489802 | 0.2 | 0.857 | -0.657 |  |  |
|  |  | 105080233 | - | 2176770 | 2176915 | 2176770 | 2176912 | 2176994 | 2177064 | 36 | 13 | 80 | 5 | 63 | 63 | 0.01563191 | 0.735 | 0.941 | -0.206 |  |  |
|  |  | 105081796 | + | 10208 | 10370 | 10211 | 10370 | 9705 | 9820 | 9 | 6 | 2 | 14 | 108 | 108 | 0.01573405 | 0.6 | 0.125 | 0.475 |  |  |
|  |  | 105062454 | - | 120723 | 120878 | 120723 | 120875 | 120976 | 121230 | 81 | 71 | 20 | 4 | 135 | 135 | 0.016336172 | 0.533 | 0.833 | -0.3 |  |  |
|  |  | 105078784 | - | 2229222 | 2230480 | 2229222 | 2230420 | 2231194 | 2231364 | 24 | 83 | 219 | 332 | 188 | 135 | 0.016806385 | 0.172 | 0.321 | -0.149 |  |  |
|  |  | 105063249 | + | 2295315 | 2295480 | 2295318 | 2295480 | 2294526 | 2294744 | 75 | 18 | 28 | 0 | 135 | 135 | 0.017103731 | 0.806 | 1 | -0.194 |  |  |
|  |  | 105077820 | - | 7576622 | 7576853 | 7576622 | 7576850 | 7577519 | 7577744 | 0 | 4 | 8 | 2 | 135 | 135 | 0.017134723 | 0 | 0.8 | -0.8 |  |  |
|  |  | 105082618 | - | 2684521 | 2684631 | 2684521 | 2684628 | 2687009 | 2687610 | 21 | 0 | 31 | 10 | 103 | 100 | 0.02146607 | 1 | 0.751 | 0.249 |  |  |
|  |  | 105064425 | - | 11821698 | 11821849 | 11821698 | 11821846 | 11824255 | 11824356 | 15 | 4 | 35 | 43 | 94 | 94 | 0.022129992 | 0.789 | 0.449 | 0.34 |  |  |
|  |  | 105071932 | - | 12355973 | 12356263 | 12355973 | 12356218 | 12360032 | 12360163 | 39 | 0 | 34 | 7 | 162 | 124 | 0.02375366 | 1 | 0.788 | 0.212 |  |  |
|  |  | 105076237 | - | 5106156 | 5106934 | 5106156 | 5106931 | 5107367 | 5107419 | 15 | 12 | 18 | 2 | 45 | 45 | 0.027236577 | 0.556 | 0.9 | -0.344 |  |  |
|  |  | 105067247 | - | 200930 | 201097 | 200930 | 201069 | 201668 | 201795 | 12 | 2 | 2 | 5 | 138 | 117 | 0.027411775 | 0.836 | 0.253 | 0.583 |  |  |
|  |  | 105072557 | + | 1111252 | 1111327 | 1111255 | 1111327 | 1104201 | 1104412 | 10 | 0 | 10 | 7 | 68 | 65 | 0.027700813 | 1 | 0.577 | 0.423 |  |  |
|  |  | 105073791 | + | 310314 | 311032 | 310772 | 311032 | 309303 | 309796 | 73 | 40 | 73 | 94 | 579 | 135 | 0.028655887 | 0.299 | 0.153 | 0.146 |  |  |
|  |  | 105072197 | - | 6363610 | 6363778 | 6363610 | 6363734 | 6364778 | 6364844 | 2 | 5 | 5 | 0 | 78 | 41 | 0.028942795 | 0.174 | 1 | -0.826 |  |  |
|  |  | 105075086 | - | 689645 | 690306 | 689645 | 689813 | 691789 | 691936 | 46 | 13 | 35 | 30 | 614 | 135 | 0.029236115 | 0.438 | 0.204 | 0.234 |  |  |
|  |  | 105072115 | - | 1718968 | 1719073 | 1718968 | 1719070 | 1720943 | 1721055 | 28 | 13 | 79 | 9 | 68 | 65 | 0.029875232 | 0.673 | 0.894 | -0.221 |  |  |
|  |  | 105076868 | - | 1709651 | 1709764 | 1709651 | 1709761 | 1714176 | 1714326 | 4 | 11 | 20 | 10 | 106 | 103 | 0.030555581 | 0.261 | 0.66 | -0.399 |  |  |
|  |  | 105070867 | - | 1039243 | 1039311 | 1039243 | 1039308 | 1040167 | 1040276 | 3 | 0 | 2 | 9 | 28 | 25 | 0.031773305 | 1 | 0.166 | 0.834 |  |  |
|  |  | 105080489 | + | 8436732 | 8436852 | 8436735 | 8436852 | 8433091 | 8434937 | 16 | 7 | 18 | 30 | 113 | 110 | 0.035556753 | 0.69 | 0.369 | 0.321 |  |  |
|  |  | 105061947 | + | 820266 | 821216 | 820524 | 821216 | 820010 | 820142 | 7 | 6 | 8 | 0 | 369 | 125 | 0.036045472 | 0.283 | 1 | -0.717 |  |  |
|  |  | 105083952 | + | 8177709 | 8177907 | 8177715 | 8177907 | 8153072 | 8153261 | 6 | 0 | 0 | 2 | 135 | 135 | 0.036742589 | 1 | 0 | 1 |  |  |
|  |  | 105083959 | + | 8627972 | 8628154 | 8628031 | 8628154 | 8626715 | 8626847 | 0 | 4 | 7 | 3 | 158 | 106 | 0.036913009 | 0 | 0.61 | -0.61 |  |  |
|  |  | 105083437 | - | 829826 | 829955 | 829826 | 829952 | 833139 | 833262 | 20 | 0 | 27 | 8 | 103 | 100 | 0.037894085 | 1 | 0.766 | 0.234 |  |  |
|  |  | 105078953 | + | 943008 | 943236 | 943167 | 943236 | 940204 | 940372 | 8 | 14 | 30 | 13 | 207 | 62 | 0.038936576 | 0.146 | 0.409 | -0.263 |  |  |
|  |  | 105067879 | + | 2963726 | 2963830 | 2963729 | 2963830 | 2958386 | 2958453 | 0 | 4 | 4 | 1 | 22 | 19 | 0.039379748 | 0 | 0.776 | -0.776 |  |  |
|  |  | 105062215 | - | 1460838 | 1461007 | 1460838 | 1460955 | 1462304 | 1462409 | 0 | 3 | 29 | 12 | 118 | 73 | 0.03986489 | 0 | 0.599 | -0.599 |  |  |
|  |  | 105071451 | + | 9952906 | 9953036 | 9952909 | 9953036 | 9950900 | 9951022 | 36 | 0 | 34 | 7 | 103 | 100 | 0.040021451 | 1 | 0.825 | 0.175 |  |  |
|  |  | 105080590 | - | 4155121 | 4155288 | 4155121 | 4155276 | 4158825 | 4158957 | 19 | 105 | 41 | 90 | 130 | 125 | 0.040091875 | 0.148 | 0.305 | -0.157 |  |  |
|  |  | 105075243 | - | 1607261 | 1607428 | 1607261 | 1607425 | 1608545 | 1608642 | 8 | 14 | 1 | 18 | 90 | 90 | 0.041908372 | 0.364 | 0.053 | 0.311 |  |  |
|  |  | 105075253 | + | 2614604 | 2615441 | 2614745 | 2615441 | 2611398 | 2611575 | 35 | 56 | 82 | 60 | 269 | 135 | 0.043154163 | 0.239 | 0.407 | -0.168 |  |  |
|  |  | 105073514 | - | 1867906 | 1868315 | 1867906 | 1868312 | 1872960 | 1873156 | 16 | 11 | 15 | 35 | 135 | 135 | 0.043890273 | 0.593 | 0.3 | 0.293 |  |  |
|  |  | 105078839 | + | 7788938 | 7789532 | 7789004 | 7789532 | 7786436 | 7786625 | 13 | 7 | 21 | 42 | 194 | 135 | 0.044622256 | 0.564 | 0.258 | 0.306 |  |  |
|  |  | 105076442 | + | 1200226 | 1203985 | 1203499 | 1203985 | 1199971 | 1200138 | 1734 | 60 | 1532 | 91 | 3394 | 135 | 0.045239608 | 0.535 | 0.401 | 0.134 |  |  |
|  |  | 105069180 | - | 7592745 | 7593110 | 7592745 | 7593040 | 7595121 | 7595550 | 5 | 2 | 0 | 4 | 198 | 135 | 0.046270878 | 0.63 | 0 | 0.63 |  |  |
|  |  | 105063581 | - | 6362324 | 6362540 | 6362324 | 6362537 | 6365119 | 6365242 | 49 | 0 | 32 | 6 | 116 | 116 | 0.049945441 | 1 | 0.842 | 0.158 |  |  |
|  |  | 105083094 | + | 3925356 | 3925493 | 3925359 | 3925493 | 3918778 | 3918927 | 11 | 0 | 43 | 17 | 130 | 127 | 0.049957434 | 1 | 0.712 | 0.288 |  |  |

Note: SS and WS indicates salt stress and water-deprivation stress, respectively.

Table S3. The differential alternative splicing events of liver under salt stress and water-deprivation stress

| **AS category** | **Stress** | **GeneID** | **strand** | **exonStart_0base** | **exonEnd** | **upstreamES** | **upstreamEE** | **downstreamES** | **downstreamEE** | **IC_SAMPLE_1** | **SC_SAMPLE_1** | **IC_SAMPLE_2** | **SC_SAMPLE_2** | **IncFormLen** | **SkipFormLen** | **PValue** | **IncLevel1** | **IncLevel2** | **IncLevelDifference** |  |  |
| --- | --- | --- | --- | --- | --- | --- | --- | --- | --- | --- | --- | --- | --- | --- | --- | --- | --- | --- | --- | --- | --- |
| SE | SS | 105064294 | + | 490327 | 490429 | 489055 | 489163 | 491360 | 491462 | 162 | 3 | 116 | 54 | 116 | 61 | 1.56E-12 | 0.966 | 0.53 | 0.436 |  |  |
|  |  | 105071974 | + | 8618643 | 8618932 | 8612562 | 8612633 | 8625800 | 8625939 | 41 | 24 | 5 | 48 | 336 | 61 | 2.75E-06 | 0.237 | 0.019 | 0.218 |  |  |
|  |  | 105063909 | - | 14408 | 14628 | 12161 | 12652 | 15478 | 15566 | 1901 | 331 | 3716 | 356 | 287 | 81 | 9.20E-06 | 0.618 | 0.747 | -0.129 |  |  |
|  |  | 105076014 | - | 1110839 | 1110908 | 1107509 | 1107706 | 1113550 | 1113619 | 0 | 42 | 11 | 15 | 62 | 62 | 3.33E-05 | 0 | 0.423 | -0.423 |  |  |
|  |  | 105078375 | - | 2339474 | 2339552 | 2336410 | 2336752 | 2340951 | 2341075 | 1 | 22 | 8 | 3 | 124 | 117 | 0.000211061 | 0.041 | 0.716 | -0.675 |  |  |
|  |  | 105082179 | + | 27648460 | 27649550 | 27637511 | 27637600 | 27654473 | 27654535 | 3481 | 6 | 2958 | 28 | 1078 | 2 | 0.000330862 | 0.518 | 0.164 | 0.354 |  |  |
|  |  | 105069413 | + | 198337 | 198375 | 196635 | 197050 | 199161 | 199263 | 27 | 207 | 18 | 625 | 31 | 95 | 0.000390845 | 0.286 | 0.081 | 0.205 |  |  |
|  |  | 105082303 | - | 3042777 | 3042846 | 3041884 | 3042026 | 3047152 | 3047215 | 109 | 145 | 282 | 175 | 62 | 56 | 0.000397044 | 0.404 | 0.593 | -0.189 |  |  |
|  |  | 105066748 | + | 910393 | 910440 | 908845 | 909041 | 911087 | 911220 | 39 | 565 | 102 | 543 | 71 | 126 | 0.000561066 | 0.109 | 0.25 | -0.141 |  |  |
|  |  | 105080044 | + | 876241 | 876366 | 875716 | 876080 | 877072 | 877308 | 163 | 36 | 269 | 14 | 236 | 135 | 0.000618987 | 0.721 | 0.917 | -0.196 |  |  |
|  |  | 105069318 | - | 35836 | 35930 | 24963 | 25073 | 40465 | 40659 | 17 | 0 | 3 | 6 | 142 | 103 | 0.00063676 | 1 | 0.266 | 0.734 |  |  |
|  |  | 105075293 | - | 6753531 | 6753672 | 6749996 | 6751546 | 6755163 | 6755258 | 175 | 21 | 69 | 30 | 221 | 88 | 0.000785794 | 0.768 | 0.478 | 0.29 |  |  |
|  |  | 105073067 | + | 4224419 | 4225302 | 4222562 | 4222685 | 4226667 | 4226866 | 616 | 0 | 468 | 13 | 985 | 116 | 0.000894576 | 1 | 0.809 | 0.191 |  |  |
|  |  | 105083785 | + | 3178766 | 3178981 | 3178523 | 3178633 | 3185366 | 3185429 | 409 | 26 | 538 | 7 | 225 | 24 | 0.000915211 | 0.627 | 0.891 | -0.264 |  |  |
|  |  | 105067855 | - | 4703707 | 4703792 | 4701767 | 4701858 | 4705514 | 4705573 | 48 | 2 | 30 | 17 | 27 | 1 | 0.001109558 | 0.471 | 0.061 | 0.41 |  |  |
|  |  | 105070480 | + | 308057 | 308237 | 305865 | 306000 | 309739 | 310221 | 10 | 0 | 0 | 4 | 294 | 128 | 0.001281272 | 1 | 0 | 1 |  |  |
|  |  | 105072404 | + | 10141769 | 10141841 | 10130171 | 10130321 | 10145264 | 10145313 | 0 | 11 | 5 | 1 | 65 | 42 | 0.001617278 | 0 | 0.764 | -0.764 |  |  |
|  |  | 105073433 | + | 2132210 | 2132371 | 2131456 | 2131581 | 2133733 | 2133944 | 139 | 1 | 83 | 12 | 265 | 118 | 0.001874605 | 0.984 | 0.755 | 0.229 |  |  |
|  |  | 105080389 | - | 4782203 | 4782301 | 4779413 | 4779628 | 4785343 | 4785476 | 9 | 0 | 4 | 9 | 173 | 126 | 0.002034474 | 1 | 0.245 | 0.755 |  |  |
|  |  | 105063679 | - | 1624852 | 1624981 | 1619671 | 1619816 | 1625617 | 1625754 | 30 | 9 | 28 | 40 | 239 | 130 | 0.002043682 | 0.645 | 0.276 | 0.369 |  |  |
|  |  | 105078478 | + | 11166737 | 11166919 | 11144392 | 11144557 | 11171000 | 11171058 | 21 | 15 | 16 | 0 | 219 | 51 | 0.00222966 | 0.246 | 1 | -0.754 |  |  |
|  |  | 105080454 | + | 3065455 | 3065526 | 3055987 | 3056081 | 3066705 | 3066826 | 44 | 13 | 46 | 51 | 59 | 66 | 0.002351151 | 0.791 | 0.502 | 0.289 |  |  |
|  |  | 105069739 | - | 2790344 | 2793969 | 2788485 | 2788622 | 2797893 | 2798068 | 1138 | 0 | 414 | 6 | 3741 | 130 | 0.002527749 | 1 | 0.706 | 0.294 |  |  |
|  |  | 105080368 | - | 3185588 | 3185609 | 3180044 | 3180155 | 3186999 | 3187211 | 32 | 236 | 11 | 282 | 14 | 104 | 0.002645224 | 0.502 | 0.225 | 0.277 |  |  |
|  |  | 105069555 | - | 2271218 | 2271364 | 2271010 | 2271127 | 2273295 | 2273426 | 124 | 27 | 184 | 100 | 234 | 99 | 0.002812515 | 0.66 | 0.438 | 0.222 |  |  |
|  |  | 105065207 | - | 3917865 | 3917918 | 3916736 | 3916947 | 3919131 | 3919191 | 15 | 0 | 3 | 5 | 46 | 53 | 0.002980572 | 1 | 0.409 | 0.591 |  |  |
|  |  | 105068762 | - | 590215 | 590399 | 587025 | 587571 | 591721 | 591786 | 15 | 2 | 6 | 12 | 228 | 58 | 0.003247155 | 0.656 | 0.113 | 0.543 |  |  |
|  |  | 105062323 | + | 12831137 | 12831226 | 12817331 | 12817410 | 12832116 | 12832277 | 37 | 24 | 76 | 12 | 101 | 72 | 0.003266306 | 0.524 | 0.819 | -0.295 |  |  |
|  |  | 105073080 | + | 5083794 | 5083920 | 5061732 | 5061799 | 5121651 | 5126007 | 62 | 5 | 49 | 21 | 163 | 60 | 0.003317581 | 0.82 | 0.462 | 0.358 |  |  |
|  |  | 105078604 | + | 6037418 | 6038973 | 6036802 | 6036918 | 6070718 | 6070947 | 223 | 0 | 146 | 7 | 1650 | 109 | 0.003573701 | 1 | 0.579 | 0.421 |  |  |
|  |  | 105080170 | + | 538989 | 539085 | 534799 | 534885 | 539880 | 539972 | 13 | 0 | 3 | 5 | 72 | 29 | 0.003733606 | 1 | 0.195 | 0.805 |  |  |
|  |  | 105064345 | - | 2636590 | 2636659 | 2630760 | 2630934 | 2636879 | 2637209 | 49 | 64 | 26 | 98 | 124 | 135 | 0.003794825 | 0.455 | 0.224 | 0.231 |  |  |
|  |  | 105067855 | - | 4704432 | 4704595 | 4701767 | 4701858 | 4705514 | 4705704 | 68 | 2 | 69 | 17 | 233 | 84 | 0.00395284 | 0.925 | 0.594 | 0.331 |  |  |
|  |  | 105082091 | - | 21419449 | 21419764 | 21418410 | 21418746 | 21421898 | 21422401 | 74 | 12 | 55 | 0 | 436 | 135 | 0.004158812 | 0.656 | 1 | -0.344 |  |  |
|  |  | 105068146 | + | 1119729 | 1119779 | 1116055 | 1117747 | 1125876 | 1126417 | 11 | 0 | 1 | 4 | 86 | 135 | 0.004513475 | 1 | 0.282 | 0.718 |  |  |
|  |  | 105074848 | - | 6897698 | 6897850 | 6897395 | 6897572 | 6898500 | 6898559 | 268 | 67 | 220 | 111 | 190 | 52 | 0.005292049 | 0.523 | 0.352 | 0.171 |  |  |
|  |  | 105072055 | - | 11256329 | 11256387 | 11246567 | 11246690 | 11273281 | 11273346 | 19 | 88 | 36 | 53 | 32 | 39 | 0.005389765 | 0.208 | 0.453 | -0.245 |  |  |
|  |  | 105074168 | + | 1413119 | 1413199 | 1404725 | 1404860 | 1417294 | 1417453 | 1 | 5 | 7 | 0 | 139 | 128 | 0.005550124 | 0.156 | 1 | -0.844 |  |  |
|  |  | 105081091 | - | 5127853 | 5127940 | 5125421 | 5125957 | 5130682 | 5130763 | 1 | 44 | 3 | 1 | 99 | 74 | 0.00568965 | 0.017 | 0.692 | -0.675 |  |  |
|  |  | 105076014 | - | 1109392 | 1109428 | 1107509 | 1107706 | 1110839 | 1110908 | 45 | 0 | 22 | 10 | 29 | 62 | 0.005691137 | 1 | 0.825 | 0.175 |  |  |
|  |  | 105078478 | + | 11167844 | 11167897 | 11144392 | 11144557 | 11171000 | 11171058 | 30 | 15 | 17 | 0 | 46 | 51 | 0.007116535 | 0.689 | 1 | -0.311 |  |  |
|  |  | 105066187 | + | 6757164 | 6757320 | 6693673 | 6693892 | 6773242 | 6774594 | 6 | 13 | 18 | 5 | 277 | 135 | 0.007869427 | 0.184 | 0.637 | -0.453 |  |  |
|  |  | 105073632 | - | 3253324 | 3253352 | 3248828 | 3248980 | 3255288 | 3255361 | 7 | 30 | 0 | 39 | 21 | 66 | 0.007966828 | 0.423 | 0 | 0.423 |  |  |
|  |  | 105066290 | - | 19382099 | 19382241 | 19378248 | 19378402 | 19383122 | 19383313 | 0 | 4 | 5 | 0 | 270 | 135 | 0.008225054 | 0 | 1 | -1 |  |  |
|  |  | 105070236 | - | 3490080 | 3490211 | 3485665 | 3487138 | 3494221 | 3494390 | 347 | 24 | 203 | 0 | 248 | 135 | 0.0083447 | 0.887 | 1 | -0.113 |  |  |
|  |  | 105062383 | - | 6154720 | 6154818 | 6152488 | 6152647 | 6155436 | 6155503 | 6 | 1 | 1 | 8 | 107 | 60 | 0.008421976 | 0.771 | 0.066 | 0.705 |  |  |
|  |  | 105082732 | - | 858347 | 858474 | 857658 | 857787 | 858792 | 858876 | 166 | 5 | 117 | 18 | 169 | 64 | 0.008441004 | 0.926 | 0.711 | 0.215 |  |  |
|  |  | 105069808 | - | 2414145 | 2414271 | 2412525 | 2413328 | 2428529 | 2428676 | 135 | 125 | 116 | 51 | 238 | 135 | 0.008908262 | 0.38 | 0.563 | -0.183 |  |  |
|  |  | 105083193 | - | 8603135 | 8603185 | 8582509 | 8582659 | 8603331 | 8603477 | 0 | 4 | 8 | 1 | 86 | 135 | 0.009032225 | 0 | 0.926 | -0.926 |  |  |
|  |  | 105074705 | + | 12752540 | 12752953 | 12737996 | 12738067 | 12765727 | 12765984 | 27 | 0 | 13 | 5 | 463 | 64 | 0.009554635 | 1 | 0.264 | 0.736 |  |  |
|  |  | 105081843 | - | 11905349 | 11905460 | 11903750 | 11903948 | 11907338 | 11907515 | 19 | 0 | 8 | 5 | 208 | 135 | 0.009867922 | 1 | 0.509 | 0.491 |  |  |
|  |  | 105062974 | - | 404626 | 404761 | 402482 | 403752 | 427155 | 427328 | 11 | 0 | 9 | 8 | 256 | 135 | 0.01003804 | 1 | 0.372 | 0.628 |  |  |
|  |  | 105066151 | + | 4384136 | 4384354 | 4383790 | 4384013 | 4388191 | 4388308 | 23 | 0 | 7 | 4 | 314 | 110 | 0.010099195 | 1 | 0.38 | 0.62 |  |  |
|  |  | 105082128 | + | 23936390 | 23936519 | 23933593 | 23933711 | 23937814 | 23940592 | 23 | 11 | 16 | 0 | 220 | 111 | 0.010583981 | 0.513 | 1 | -0.487 |  |  |
|  |  | 105064345 | - | 2636590 | 2636654 | 2630760 | 2630934 | 2636879 | 2637239 | 41 | 64 | 23 | 98 | 114 | 135 | 0.011041175 | 0.431 | 0.217 | 0.214 |  |  |
|  |  | 105079442 | + | 11039511 | 11039571 | 11037737 | 11038127 | 11042751 | 11043153 | 3 | 12 | 4 | 0 | 106 | 135 | 0.011067743 | 0.242 | 1 | -0.758 |  |  |
|  |  | 105082910 | + | 6627004 | 6627120 | 6623168 | 6623314 | 6631134 | 6631313 | 46 | 8 | 56 | 0 | 218 | 135 | 0.011280177 | 0.781 | 1 | -0.219 |  |  |
|  |  | 105078056 | + | 869962 | 870104 | 867417 | 867547 | 877712 | 878602 | 10 | 0 | 10 | 9 | 258 | 123 | 0.011741655 | 1 | 0.346 | 0.654 |  |  |
|  |  | 105063678 | - | 1522577 | 1524084 | 1521105 | 1521375 | 1534880 | 1535158 | 261 | 1 | 108 | 6 | 1628 | 135 | 0.011796572 | 0.956 | 0.599 | 0.357 |  |  |
|  |  | 105072912 | - | 7664827 | 7664895 | 7629297 | 7629384 | 7683155 | 7683422 | 92 | 28 | 138 | 10 | 67 | 80 | 0.011809057 | 0.797 | 0.943 | -0.146 |  |  |
|  |  | 105074944 | + | 787054 | 787273 | 784097 | 784233 | 788495 | 788687 | 131 | 0 | 110 | 9 | 334 | 129 | 0.011848201 | 1 | 0.825 | 0.175 |  |  |
|  |  | 105083404 | - | 11951684 | 11951921 | 11947765 | 11947860 | 11957559 | 11957656 | 60 | 6 | 82 | 0 | 266 | 43 | 0.012231609 | 0.618 | 1 | -0.382 |  |  |
|  |  | 105074722 | + | 14089726 | 14089859 | 14078636 | 14080343 | 14095367 | 14095552 | 69 | 6 | 24 | 11 | 252 | 135 | 0.012328759 | 0.86 | 0.539 | 0.321 |  |  |
|  |  | 105078304 | + | 5536180 | 5536234 | 5529214 | 5529292 | 5538224 | 5539027 | 23 | 19 | 17 | 49 | 47 | 71 | 0.012420998 | 0.646 | 0.344 | 0.302 |  |  |
|  |  | 105077697 | + | 1755904 | 1756022 | 1754730 | 1755099 | 1758456 | 1759253 | 21 | 0 | 10 | 5 | 222 | 135 | 0.01251042 | 1 | 0.549 | 0.451 |  |  |
|  |  | 105063265 | + | 3633235 | 3633361 | 3621648 | 3621861 | 3647611 | 3647753 | 14 | 0 | 4 | 4 | 238 | 135 | 0.012549014 | 1 | 0.362 | 0.638 |  |  |
|  |  | 105080897 | - | 50530 | 50702 | 49210 | 49345 | 63418 | 63482 | 95 | 11 | 64 | 24 | 208 | 50 | 0.012919354 | 0.675 | 0.391 | 0.284 |  |  |
|  |  | 105065416 | + | 355614 | 355868 | 343560 | 343609 | 361294 | 361528 | 150 | 11 | 150 | 1 | 282 | 42 | 0.013393539 | 0.67 | 0.957 | -0.287 |  |  |
|  |  | 105061950 | - | 951193 | 951261 | 948833 | 948990 | 951661 | 951722 | 3 | 16 | 11 | 7 | 61 | 54 | 0.013490437 | 0.142 | 0.582 | -0.44 |  |  |
|  |  | 105063680 | + | 1646027 | 1646112 | 1640026 | 1640168 | 1658687 | 1658819 | 8 | 0 | 4 | 6 | 146 | 125 | 0.013504283 | 1 | 0.363 | 0.637 |  |  |
|  |  | 105078576 | - | 1331837 | 1331941 | 1322143 | 1322239 | 1425186 | 1425338 | 129 | 15 | 70 | 0 | 148 | 89 | 0.013992582 | 0.838 | 1 | -0.162 |  |  |
|  |  | 105083623 | + | 340359 | 340431 | 339044 | 339150 | 347540 | 347666 | 18 | 7 | 2 | 8 | 78 | 83 | 0.014578907 | 0.732 | 0.21 | 0.522 |  |  |
|  |  | 105066190 | + | 7419324 | 7419555 | 7415601 | 7415767 | 7422683 | 7422756 | 5 | 5 | 26 | 2 | 283 | 66 | 0.014777279 | 0.189 | 0.752 | -0.563 |  |  |
|  |  | 105083721 | + | 6271645 | 6271696 | 6271193 | 6271279 | 6271944 | 6272151 | 5 | 15 | 0 | 29 | 44 | 79 | 0.014869528 | 0.374 | 0 | 0.374 |  |  |
|  |  | 105066364 | - | 2107660 | 2107871 | 2106762 | 2106846 | 2108591 | 2108758 | 111 | 11 | 67 | 0 | 274 | 77 | 0.015118661 | 0.739 | 1 | -0.261 |  |  |
|  |  | 105083574 | - | 18937123 | 18937165 | 18934628 | 18934780 | 18938199 | 18938355 | 10 | 2 | 2 | 7 | 70 | 135 | 0.015436789 | 0.906 | 0.355 | 0.551 |  |  |
|  |  | 105073541 | - | 2986892 | 2986970 | 2967224 | 2967315 | 2990736 | 2990797 | 29 | 1 | 9 | 5 | 20 | 3 | 0.015486421 | 0.813 | 0.213 | 0.6 |  |  |
|  |  | 105072415 | + | 10709115 | 10709761 | 10704951 | 10705098 | 10719583 | 10719678 | 19 | 0 | 7 | 4 | 720 | 88 | 0.015568744 | 1 | 0.176 | 0.824 |  |  |
|  |  | 105077842 | + | 11961258 | 11961335 | 11960372 | 11960576 | 11962735 | 11962927 | 11 | 6 | 17 | 0 | 140 | 135 | 0.01568039 | 0.639 | 1 | -0.361 |  |  |
|  |  | 105064522 | - | 1799271 | 1799319 | 1793509 | 1793616 | 1808493 | 1808620 | 66 | 258 | 27 | 237 | 32 | 85 | 0.015733875 | 0.405 | 0.232 | 0.173 |  |  |
|  |  | 105064215 | + | 3547424 | 3548255 | 3546201 | 3546420 | 3548364 | 3548465 | 274 | 40 | 320 | 96 | 911 | 94 | 0.015853259 | 0.414 | 0.256 | 0.158 |  |  |
|  |  | 105081390 | + | 564613 | 564667 | 560711 | 560837 | 564930 | 565011 | 92 | 87 | 38 | 80 | 31 | 58 | 0.015894339 | 0.664 | 0.471 | 0.193 |  |  |
|  |  | 105076508 | + | 10171821 | 10172055 | 10164361 | 10164488 | 10176867 | 10177062 | 962 | 95 | 489 | 91 | 340 | 120 | 0.015902898 | 0.781 | 0.655 | 0.126 |  |  |
|  |  | 105066407 | - | 38075 | 38151 | 30040 | 30329 | 39907 | 40079 | 19 | 23 | 34 | 11 | 138 | 135 | 0.016535921 | 0.447 | 0.751 | -0.304 |  |  |
|  |  | 105070087 | + | 747988 | 748071 | 747031 | 747252 | 749432 | 749570 | 7 | 0 | 7 | 9 | 148 | 131 | 0.016660517 | 1 | 0.408 | 0.592 |  |  |
|  |  | 105076595 | - | 22990542 | 22990680 | 22986855 | 22987009 | 22991331 | 22991430 | 47 | 26 | 93 | 18 | 219 | 92 | 0.017504959 | 0.432 | 0.685 | -0.253 |  |  |
|  |  | 105079546 | - | 1192790 | 1192877 | 1180139 | 1180231 | 1205687 | 1205820 | 7 | 10 | 10 | 1 | 101 | 76 | 0.017730783 | 0.345 | 0.883 | -0.538 |  |  |
|  |  | 105078982 | - | 4900717 | 4900896 | 4899118 | 4899240 | 4902502 | 4903341 | 25 | 12 | 22 | 1 | 280 | 115 | 0.017929012 | 0.461 | 0.9 | -0.439 |  |  |
|  |  | 105061921 | - | 4573301 | 4573391 | 4572064 | 4572265 | 4575149 | 4575256 | 6 | 0 | 2 | 5 | 131 | 100 | 0.018078132 | 1 | 0.234 | 0.766 |  |  |
|  |  | 105063954 | - | 5217499 | 5217693 | 5215697 | 5215810 | 5226727 | 5226843 | 96 | 0 | 82 | 7 | 260 | 80 | 0.018917716 | 1 | 0.783 | 0.217 |  |  |
|  |  | 105076451 | - | 1349020 | 1349287 | 1348553 | 1348762 | 1349703 | 1349892 | 328 | 29 | 445 | 84 | 388 | 135 | 0.020466991 | 0.797 | 0.648 | 0.149 |  |  |
|  |  | 105075112 | - | 4004064 | 4004263 | 4001685 | 4001817 | 4007762 | 4007900 | 56 | 0 | 22 | 4 | 306 | 121 | 0.021232332 | 1 | 0.685 | 0.315 |  |  |
|  |  | 105064800 | - | 1399395 | 1399473 | 1395445 | 1395553 | 1400299 | 1400372 | 0 | 7 | 5 | 3 | 39 | 32 | 0.02164537 | 0 | 0.578 | -0.578 |  |  |
|  |  | 105079554 | + | 4165258 | 4165451 | 4163911 | 4164232 | 4166430 | 4166715 | 299 | 48 | 292 | 91 | 314 | 135 | 0.022877669 | 0.728 | 0.58 | 0.148 |  |  |
|  |  | 105070813 | + | 1165592 | 1165691 | 1165246 | 1165296 | 1165807 | 1165924 | 38 | 33 | 117 | 41 | 67 | 18 | 0.023002617 | 0.236 | 0.434 | -0.198 |  |  |
|  |  | 105062852 | + | 749544 | 749632 | 746171 | 746246 | 751146 | 751273 | 7 | 0 | 2 | 4 | 80 | 53 | 0.023087613 | 1 | 0.249 | 0.751 |  |  |
|  |  | 105064215 | + | 3547435 | 3548255 | 3546201 | 3546420 | 3548364 | 3548465 | 246 | 40 | 292 | 96 | 900 | 94 | 0.023210753 | 0.391 | 0.241 | 0.15 |  |  |
|  |  | 105078375 | - | 2337040 | 2337124 | 2336410 | 2336752 | 2340951 | 2341075 | 23 | 22 | 17 | 3 | 136 | 117 | 0.023327242 | 0.474 | 0.83 | -0.356 |  |  |
|  |  | 105076675 | - | 31160398 | 31160531 | 31159980 | 31160076 | 31161884 | 31161973 | 2 | 4 | 7 | 0 | 153 | 36 | 0.024339759 | 0.105 | 1 | -0.895 |  |  |
|  |  | 105075963 | - | 167915 | 168062 | 155424 | 155577 | 177328 | 177420 | 19 | 6 | 21 | 0 | 220 | 85 | 0.024957657 | 0.55 | 1 | -0.45 |  |  |
|  |  | 105068283 | + | 1826834 | 1826917 | 1825405 | 1825644 | 1828935 | 1829103 | 10 | 13 | 9 | 1 | 152 | 135 | 0.025523018 | 0.406 | 0.889 | -0.483 |  |  |
|  |  | 105078511 | + | 157229 | 157418 | 135598 | 135716 | 171055 | 171212 | 17 | 1 | 12 | 8 | 286 | 111 | 0.025991738 | 0.868 | 0.368 | 0.5 |  |  |
|  |  | 105070045 | + | 2268763 | 2269027 | 2268180 | 2268309 | 2269383 | 2269524 | 80 | 28 | 30 | 28 | 371 | 121 | 0.026043594 | 0.482 | 0.259 | 0.223 |  |  |
|  |  | 105073514 | - | 1912621 | 1912810 | 1911908 | 1912088 | 1913519 | 1913633 | 130 | 0 | 54 | 5 | 282 | 107 | 0.026088732 | 1 | 0.804 | 0.196 |  |  |
|  |  | 105081550 | + | 4212822 | 4212858 | 4207382 | 4207482 | 4214452 | 4214660 | 2 | 80 | 7 | 36 | 29 | 93 | 0.02617854 | 0.074 | 0.384 | -0.31 |  |  |
|  |  | 105076445 | - | 1242155 | 1242260 | 1241954 | 1242044 | 1242644 | 1242769 | 5 | 4 | 22 | 1 | 127 | 66 | 0.026449424 | 0.394 | 0.92 | -0.526 |  |  |
|  |  | 105074416 | - | 3072034 | 3072157 | 3068164 | 3071382 | 3072691 | 3072818 | 10 | 0 | 4 | 4 | 217 | 120 | 0.026467998 | 1 | 0.356 | 0.644 |  |  |
|  |  | 105066000 | - | 1085054 | 1085144 | 1084893 | 1084943 | 1085373 | 1085533 | 13 | 1 | 15 | 12 | 83 | 43 | 0.026611828 | 0.871 | 0.393 | 0.478 |  |  |
|  |  | 105072962 | - | 15794534 | 15794701 | 15791066 | 15791229 | 15798365 | 15798485 | 38 | 0 | 50 | 8 | 266 | 113 | 0.026653912 | 1 | 0.726 | 0.274 |  |  |
|  |  | 105080144 | - | 852227 | 852320 | 851395 | 851518 | 855182 | 855287 | 11 | 15 | 3 | 27 | 116 | 79 | 0.026823543 | 0.333 | 0.07 | 0.263 |  |  |
|  |  | 105071712 | + | 635762 | 635871 | 634654 | 634830 | 637357 | 637648 | 46 | 1 | 10 | 4 | 204 | 135 | 0.027261696 | 0.968 | 0.623 | 0.345 |  |  |
|  |  | 105071507 | - | 1677268 | 1677376 | 1675124 | 1675211 | 1680102 | 1680279 | 51 | 1 | 30 | 7 | 147 | 80 | 0.028469087 | 0.965 | 0.7 | 0.265 |  |  |
|  |  | 105078324 | - | 5916381 | 5916500 | 5916056 | 5916283 | 5918009 | 5918133 | 14 | 1 | 8 | 7 | 206 | 117 | 0.029431009 | 0.888 | 0.394 | 0.494 |  |  |
|  |  | 105077135 | - | 7747004 | 7747140 | 7744458 | 7744574 | 7747906 | 7747966 | 5 | 1 | 1 | 8 | 150 | 27 | 0.029582886 | 0.474 | 0.022 | 0.452 |  |  |
|  |  | 105075042 | - | 1077801 | 1077864 | 1076512 | 1076721 | 1078273 | 1078363 | 23 | 8 | 43 | 1 | 60 | 83 | 0.029668554 | 0.799 | 0.983 | -0.184 |  |  |
|  |  | 105067099 | + | 761338 | 761353 | 760480 | 760721 | 761691 | 761805 | 5 | 12 | 0 | 17 | 8 | 107 | 0.029697632 | 0.848 | 0 | 0.848 |  |  |
|  |  | 105071735 | - | 935119 | 935948 | 929009 | 929102 | 936534 | 936730 | 35 | 2 | 34 | 12 | 901 | 86 | 0.029745237 | 0.626 | 0.213 | 0.413 |  |  |
|  |  | 105066529 | - | 7682369 | 7682538 | 7680862 | 7680979 | 7683024 | 7683136 | 30 | 20 | 45 | 86 | 235 | 80 | 0.029984915 | 0.338 | 0.151 | 0.187 |  |  |
|  |  | 105077696 | - | 3816132 | 3816192 | 3813690 | 3813802 | 3817815 | 3817940 | 13 | 0 | 10 | 6 | 59 | 88 | 0.03032071 | 1 | 0.713 | 0.287 |  |  |
|  |  | 105062628 | + | 4275346 | 4275420 | 4274485 | 4274675 | 4276684 | 4276822 | 3 | 5 | 6 | 0 | 130 | 131 | 0.030478375 | 0.377 | 1 | -0.623 |  |  |
|  |  | 105077330 | - | 37540158 | 37540299 | 37533446 | 37533614 | 37541374 | 37541569 | 104 | 136 | 88 | 58 | 268 | 135 | 0.030644485 | 0.278 | 0.433 | -0.155 |  |  |
|  |  | 105064019 | - | 6862017 | 6862201 | 6860138 | 6860452 | 6862435 | 6862457 | 11 | 7 | 11 | 0 | 185 | 15 | 0.031837959 | 0.113 | 1 | -0.887 |  |  |
|  |  | 105062308 | + | 11913644 | 11913758 | 11911137 | 11911280 | 11916283 | 11916397 | 42 | 62 | 79 | 53 | 186 | 107 | 0.031868732 | 0.28 | 0.462 | -0.182 |  |  |
|  |  | 105066143 | - | 3713874 | 3713972 | 3713489 | 3713554 | 3716489 | 3716588 | 5 | 0 | 2 | 5 | 62 | 15 | 0.032130435 | 1 | 0.088 | 0.912 |  |  |
|  |  | 105077211 | + | 20973291 | 20973366 | 20972811 | 20972923 | 20974755 | 20974915 | 4 | 59 | 9 | 22 | 106 | 105 | 0.032552743 | 0.063 | 0.288 | -0.225 |  |  |
|  |  | 105082008 | - | 11868117 | 11868344 | 11856477 | 11856651 | 11875319 | 11875539 | 7 | 6 | 0 | 7 | 348 | 135 | 0.032738484 | 0.312 | 0 | 0.312 |  |  |
|  |  | 105065282 | + | 827468 | 827534 | 822820 | 822903 | 836351 | 836544 | 2 | 4 | 6 | 0 | 59 | 76 | 0.033155865 | 0.392 | 1 | -0.608 |  |  |
|  |  | 105063642 | - | 383493 | 383600 | 381602 | 381693 | 385376 | 385536 | 23 | 0 | 17 | 5 | 149 | 84 | 0.033268201 | 1 | 0.657 | 0.343 |  |  |
|  |  | 105062081 | + | 86868 | 87015 | 82994 | 83168 | 90271 | 90428 | 39 | 5 | 12 | 8 | 270 | 135 | 0.033582894 | 0.796 | 0.429 | 0.367 |  |  |
|  |  | 105078082 | + | 101911 | 102138 | 101484 | 101713 | 102650 | 102847 | 51 | 0 | 59 | 7 | 348 | 135 | 0.034325885 | 1 | 0.766 | 0.234 |  |  |
|  |  | 105063387 | - | 1202494 | 1202667 | 1202207 | 1202395 | 1207374 | 1207574 | 38 | 0 | 28 | 5 | 294 | 135 | 0.035161549 | 1 | 0.72 | 0.28 |  |  |
|  |  | 105066756 | - | 1086122 | 1086190 | 1083850 | 1085863 | 1086287 | 1088792 | 5 | 6 | 0 | 9 | 122 | 135 | 0.035378933 | 0.48 | 0 | 0.48 |  |  |
|  |  | 105081550 | + | 4192444 | 4192573 | 4181587 | 4181725 | 4198130 | 4198286 | 75 | 56 | 65 | 21 | 240 | 131 | 0.036276169 | 0.422 | 0.628 | -0.206 |  |  |
|  |  | 105076948 | + | 8480093 | 8480153 | 8479793 | 8479919 | 8480422 | 8480502 | 7 | 0 | 13 | 11 | 37 | 57 | 0.036763536 | 1 | 0.645 | 0.355 |  |  |
|  |  | 105066512 | - | 3948116 | 3948291 | 3941833 | 3942033 | 3952046 | 3952153 | 16 | 10 | 1 | 7 | 261 | 100 | 0.037128348 | 0.38 | 0.052 | 0.328 |  |  |
|  |  | 105072299 | + | 2287 | 2308 | 1696 | 1783 | 2886 | 3063 | 17 | 87 | 7 | 115 | 14 | 80 | 0.037815062 | 0.528 | 0.258 | 0.27 |  |  |
|  |  | 105071767 | - | 2198573 | 2198714 | 2197355 | 2197436 | 2205317 | 2205581 | 46 | 21 | 15 | 21 | 207 | 74 | 0.038118185 | 0.439 | 0.203 | 0.236 |  |  |
|  |  | 105077460 | + | 43780549 | 43780666 | 43779621 | 43779718 | 43783972 | 43784073 | 19 | 6 | 5 | 9 | 134 | 49 | 0.03849507 | 0.537 | 0.169 | 0.368 |  |  |
|  |  | 105062410 | - | 9824639 | 9824701 | 9821385 | 9821438 | 9827880 | 9827993 | 4 | 8 | 0 | 22 | 26 | 17 | 0.03858 | 0.246 | 0 | 0.246 |  |  |
|  |  | 105063498 | - | 3434002 | 3434044 | 3430243 | 3430354 | 3434397 | 3434542 | 1 | 26 | 6 | 15 | 39 | 104 | 0.039328859 | 0.093 | 0.516 | -0.423 |  |  |
|  |  | 105067177 | + | 96244 | 96371 | 89039 | 89129 | 102796 | 105637 | 1 | 4 | 7 | 1 | 188 | 83 | 0.039748469 | 0.099 | 0.756 | -0.657 |  |  |
|  |  | 105074220 | + | 96479 | 96652 | 91350 | 91508 | 96864 | 96894 | 11 | 2 | 63 | 0 | 182 | 23 | 0.041163753 | 0.41 | 1 | -0.59 |  |  |
|  |  | 105075245 | - | 2174459 | 2174580 | 2158527 | 2159162 | 2191132 | 2191218 | 11 | 4 | 37 | 1 | 172 | 79 | 0.041449973 | 0.558 | 0.944 | -0.386 |  |  |
|  |  | 105077732 | - | 1996071 | 1996161 | 1992909 | 1993062 | 1996701 | 1996802 | 42 | 0 | 26 | 5 | 125 | 94 | 0.041558433 | 1 | 0.796 | 0.204 |  |  |
|  |  | 105065992 | + | 947597 | 947612 | 947056 | 947219 | 949377 | 949509 | 7 | 111 | 1 | 134 | 8 | 125 | 0.041720731 | 0.496 | 0.104 | 0.392 |  |  |
|  |  | 105071434 | - | 8904588 | 8904756 | 8902637 | 8902964 | 8907998 | 8908165 | 0 | 6 | 2 | 0 | 289 | 135 | 0.042446051 | 0 | 1 | -1 |  |  |
|  |  | 105074924 | + | 3744161 | 3744323 | 3741189 | 3741246 | 3747971 | 3748073 | 53 | 0 | 24 | 3 | 158 | 10 | 0.043339814 | 1 | 0.336 | 0.664 |  |  |
|  |  | 105061839 | - | 10052 | 10079 | 9312 | 9782 | 10376 | 10477 | 6 | 1 | 1 | 5 | 20 | 94 | 0.043747574 | 0.966 | 0.485 | 0.481 |  |  |
|  |  | 105067496 | - | 24167368 | 24167493 | 24163824 | 24163937 | 24167619 | 24167723 | 2 | 2 | 15 | 0 | 169 | 68 | 0.043836763 | 0.287 | 1 | -0.713 |  |  |
|  |  | 105067524 | + | 3901325 | 3901520 | 3900530 | 3900675 | 3904053 | 3904233 | 7 | 6 | 0 | 6 | 316 | 135 | 0.043971747 | 0.333 | 0 | 0.333 |  |  |
|  |  | 105061747 | + | 1949492 | 1949659 | 1947551 | 1947723 | 1951009 | 1951864 | 105 | 0 | 103 | 8 | 288 | 135 | 0.044066733 | 1 | 0.858 | 0.142 |  |  |
|  |  | 105073038 | - | 2503399 | 2503615 | 2502290 | 2502423 | 2507109 | 2507170 | 280 | 0 | 413 | 11 | 247 | 45 | 0.044274467 | 1 | 0.872 | 0.128 |  |  |
|  |  | 105077235 | - | 22949026 | 22949318 | 22947249 | 22947623 | 22950761 | 22950954 | 84 | 2 | 61 | 9 | 413 | 135 | 0.044421421 | 0.932 | 0.689 | 0.243 |  |  |
|  |  | 105077261 | - | 25800066 | 25800177 | 25789656 | 25789784 | 25804184 | 25804302 | 40 | 6 | 42 | 0 | 170 | 97 | 0.044727259 | 0.792 | 1 | -0.208 |  |  |
|  |  | 105066065 | - | 3308871 | 3309030 | 3308341 | 3308512 | 3314522 | 3314620 | 12 | 6 | 37 | 3 | 236 | 91 | 0.04478969 | 0.435 | 0.826 | -0.391 |  |  |
|  |  | 105071497 | + | 1226134 | 1226262 | 1221710 | 1221833 | 1242624 | 1242824 | 24 | 0 | 39 | 8 | 223 | 116 | 0.044831321 | 1 | 0.717 | 0.283 |  |  |
|  |  | 105068659 | + | 3401301 | 3401392 | 3399138 | 3399198 | 3404943 | 3405133 | 10 | 0 | 1 | 2 | 86 | 53 | 0.04496556 | 1 | 0.236 | 0.764 |  |  |
|  |  | 105077295 | + | 31432577 | 31432631 | 31431013 | 31431177 | 31450693 | 31452297 | 0 | 5 | 2 | 0 | 94 | 135 | 0.044984132 | 0 | 1 | -1 |  |  |
|  |  | 105076934 | - | 7783655 | 7783721 | 7777877 | 7777942 | 7790732 | 7790844 | 5 | 0 | 0 | 2 | 29 | 28 | 0.045764945 | 1 | 0 | 1 |  |  |
|  |  | 105081945 | + | 5159865 | 5160446 | 5154579 | 5154695 | 5163962 | 5164566 | 455 | 0 | 394 | 9 | 676 | 109 | 0.045931763 | 1 | 0.876 | 0.124 |  |  |
|  |  | 105079220 | - | 90197 | 90278 | 86261 | 86438 | 91180 | 91281 | 5 | 5 | 0 | 7 | 107 | 94 | 0.046286858 | 0.468 | 0 | 0.468 |  |  |
|  |  | 105068821 | + | 4809923 | 4809956 | 4809645 | 4809783 | 4811783 | 4812148 | 34 | 39 | 62 | 27 | 48 | 131 | 0.046431962 | 0.704 | 0.862 | -0.158 |  |  |
|  |  | 105064040 | + | 7336040 | 7336260 | 7335281 | 7335471 | 7336815 | 7336936 | 70 | 0 | 100 | 8 | 320 | 114 | 0.046669463 | 1 | 0.817 | 0.183 |  |  |
|  |  | 105083627 | - | 1900569 | 1900717 | 1831608 | 1831731 | 1907934 | 1908009 | 13 | 2 | 0 | 2 | 184 | 49 | 0.047329322 | 0.634 | 0 | 0.634 |  |  |
|  |  | 105073911 | + | 3245327 | 3245431 | 3238313 | 3238466 | 3250460 | 3250581 | 1 | 3 | 5 | 0 | 173 | 114 | 0.047501279 | 0.18 | 1 | -0.82 |  |  |
|  |  | 105063387 | - | 1202494 | 1202611 | 1202207 | 1202395 | 1207374 | 1207574 | 25 | 0 | 21 | 5 | 220 | 135 | 0.047517218 | 1 | 0.72 | 0.28 |  |  |
|  |  | 105066423 | + | 598661 | 598766 | 595084 | 595172 | 601895 | 602099 | 16 | 0 | 6 | 3 | 142 | 81 | 0.047635603 | 1 | 0.533 | 0.467 |  |  |
|  |  | 105074591 | + | 1776611 | 1776875 | 1746108 | 1746627 | 1788246 | 1788356 | 76 | 19 | 17 | 13 | 353 | 103 | 0.047752626 | 0.539 | 0.276 | 0.263 |  |  |
|  |  | 105067581 | - | 24671576 | 24671717 | 24667519 | 24667654 | 24673530 | 24673632 | 0 | 4 | 5 | 2 | 221 | 88 | 0.048354849 | 0 | 0.499 | -0.499 |  |  |
|  |  | 105077264 | - | 26030358 | 26030382 | 26024957 | 26025151 | 26043981 | 26044121 | 4 | 11 | 12 | 5 | 32 | 133 | 0.048389504 | 0.602 | 0.909 | -0.307 |  |  |
|  |  | 105073034 | - | 3121312 | 3121496 | 3100955 | 3101373 | 3122548 | 3122687 | 89 | 4 | 88 | 16 | 302 | 132 | 0.048576565 | 0.907 | 0.706 | 0.201 |  |  |
|  |  | 105083640 | + | 3448437 | 3448682 | 3445795 | 3445954 | 3452582 | 3452742 | 35 | 0 | 14 | 3 | 366 | 135 | 0.049314892 | 1 | 0.633 | 0.367 |  |  |
|  |  | 105073053 | + | 3835154 | 3835282 | 3834103 | 3834153 | 3841280 | 3842533 | 27 | 2 | 6 | 4 | 150 | 43 | 0.049745145 | 0.795 | 0.301 | 0.494 |  |  |
|  |  | 105074606 | + | 2481109 | 2481196 | 2478977 | 2479068 | 2484506 | 2484596 | 14 | 22 | 20 | 9 | 57 | 32 | 0.04994161 | 0.263 | 0.555 | -0.292 |  |  |
|  | WS | 105082303 | - | 3042777 | 3042846 | 3041884 | 3042026 | 3047152 | 3047215 | 94 | 224 | 282 | 175 | 62 | 56 | 5.24E-14 | 0.275 | 0.593 | -0.318 |  |  |
|  |  | 105072845 | + | 197184 | 197341 | 192027 | 192235 | 201167 | 201304 | 119 | 69 | 157 | 17 | 273 | 130 | 9.35E-08 | 0.451 | 0.815 | -0.364 |  |  |
|  |  | 105065177 | + | 158851 | 159047 | 155349 | 155669 | 160537 | 160631 | 335 | 42 | 123 | 59 | 269 | 87 | 5.43E-07 | 0.721 | 0.403 | 0.318 |  |  |
|  |  | 105073481 | - | 98079 | 98194 | 96780 | 96909 | 100716 | 100844 | 693 | 27 | 2369 | 0 | 189 | 108 | 2.18E-06 | 0.936 | 1 | -0.064 |  |  |
|  |  | 105073960 | - | 10243669 | 10243891 | 10241519 | 10241762 | 10248891 | 10249003 | 312 | 33 | 863 | 12 | 313 | 105 | 3.58E-06 | 0.76 | 0.96 | -0.2 |  |  |
|  |  | 105076595 | - | 22990542 | 22990680 | 22986855 | 22987009 | 22991311 | 22991430 | 205 | 50 | 117 | 88 | 239 | 112 | 9.27E-06 | 0.658 | 0.384 | 0.274 |  |  |
|  |  | 105067849 | + | 4170535 | 4170631 | 4165952 | 4166125 | 4200736 | 4200868 | 87 | 33 | 185 | 12 | 168 | 125 | 3.13E-05 | 0.662 | 0.92 | -0.258 |  |  |
|  |  | 105064571 | - | 1196969 | 1197038 | 1195827 | 1196003 | 1199084 | 1199216 | 19 | 13 | 35 | 0 | 114 | 125 | 7.22E-05 | 0.616 | 1 | -0.384 |  |  |
|  |  | 105068902 | + | 971202 | 971258 | 970065 | 970329 | 974015 | 974235 | 146 | 237 | 93 | 346 | 98 | 135 | 8.72E-05 | 0.459 | 0.27 | 0.189 |  |  |
|  |  | 105066221 | - | 11943308 | 11943416 | 11940582 | 11940721 | 11943509 | 11943946 | 10 | 23 | 27 | 6 | 199 | 132 | 0.000126121 | 0.224 | 0.749 | -0.525 |  |  |
|  |  | 105063978 | - | 4005730 | 4005817 | 4003856 | 4003943 | 4006082 | 4006169 | 51 | 77 | 74 | 32 | 50 | 25 | 0.000158021 | 0.249 | 0.536 | -0.287 |  |  |
|  |  | 105074848 | - | 6897698 | 6897850 | 6897395 | 6897572 | 6898500 | 6898559 | 210 | 242 | 220 | 111 | 190 | 52 | 0.000162087 | 0.192 | 0.352 | -0.16 |  |  |
|  |  | 105076948 | + | 8480093 | 8480153 | 8479793 | 8479919 | 8480422 | 8480502 | 30 | 0 | 13 | 11 | 37 | 57 | 0.000172345 | 1 | 0.645 | 0.355 |  |  |
|  |  | 105063427 | + | 1052537 | 1052675 | 1046433 | 1046570 | 1058744 | 1058830 | 8 | 0 | 2 | 14 | 201 | 74 | 0.0001732 | 1 | 0.05 | 0.95 |  |  |
|  |  | 105066913 | - | 623364 | 623528 | 622145 | 622170 | 626654 | 626913 | 168 | 8 | 581 | 0 | 168 | 18 | 0.000184037 | 0.692 | 1 | -0.308 |  |  |
|  |  | 105068185 | - | 2103347 | 2103440 | 2102756 | 2103019 | 2103562 | 2103625 | 115 | 90 | 146 | 41 | 93 | 56 | 0.000215369 | 0.435 | 0.682 | -0.247 |  |  |
|  |  | 105066204 | + | 8952493 | 8952597 | 8950962 | 8951068 | 8953336 | 8953524 | 24 | 12 | 5 | 25 | 158 | 99 | 0.000250019 | 0.556 | 0.111 | 0.445 |  |  |
|  |  | 105070236 | - | 3490080 | 3490211 | 3485665 | 3487138 | 3494221 | 3494390 | 123 | 15 | 203 | 0 | 248 | 135 | 0.000277177 | 0.817 | 1 | -0.183 |  |  |
|  |  | 105081782 | - | 6420937 | 6421022 | 6419342 | 6419590 | 6422819 | 6422983 | 9 | 38 | 11 | 3 | 156 | 135 | 0.000340883 | 0.17 | 0.76 | -0.59 |  |  |
|  |  | 105062383 | - | 6154720 | 6154818 | 6152488 | 6152647 | 6155436 | 6155503 | 33 | 8 | 1 | 8 | 107 | 60 | 0.000417591 | 0.698 | 0.066 | 0.632 |  |  |
|  |  | 105069105 | - | 2408307 | 2408434 | 2405888 | 2406016 | 2409588 | 2409671 | 302 | 92 | 164 | 16 | 167 | 62 | 0.000571653 | 0.549 | 0.792 | -0.243 |  |  |
|  |  | 105072835 | + | 6932279 | 6932471 | 6932005 | 6932180 | 6936975 | 6937126 | 32 | 10 | 50 | 0 | 313 | 135 | 0.000573745 | 0.58 | 1 | -0.42 |  |  |
|  |  | 105063438 | + | 1319930 | 1319993 | 1319174 | 1319322 | 1321010 | 1321243 | 11 | 1 | 1 | 8 | 112 | 135 | 0.000660923 | 0.93 | 0.131 | 0.799 |  |  |
|  |  | 105083320 | + | 2832772 | 2832984 | 2828532 | 2828925 | 2840428 | 2843924 | 119 | 56 | 154 | 24 | 333 | 135 | 0.000738323 | 0.463 | 0.722 | -0.259 |  |  |
|  |  | 105066913 | - | 622145 | 622170 | 621432 | 621471 | 626654 | 626913 | 9 | 0 | 0 | 5 | 18 | 32 | 0.000791623 | 1 | 0 | 1 |  |  |
|  |  | 105067591 | + | 1870807 | 1870899 | 1868318 | 1868710 | 1875789 | 1875860 | 24 | 9 | 43 | 0 | 99 | 64 | 0.000913667 | 0.633 | 1 | -0.367 |  |  |
|  |  | 105068790 | + | 1128242 | 1128393 | 1127768 | 1128102 | 1130148 | 1131147 | 37 | 12 | 40 | 0 | 272 | 135 | 0.000976123 | 0.605 | 1 | -0.395 |  |  |
|  |  | 105072814 | - | 6070689 | 6070800 | 6069382 | 6069505 | 6072225 | 6072340 | 14 | 10 | 42 | 2 | 162 | 89 | 0.000977154 | 0.435 | 0.92 | -0.485 |  |  |
|  |  | 105065298 | - | 1286501 | 1286621 | 1259701 | 1262736 | 1305357 | 1305409 | 42 | 28 | 82 | 13 | 136 | 45 | 0.001071575 | 0.332 | 0.676 | -0.344 |  |  |
|  |  | 105069542 | + | 2186416 | 2186550 | 2185723 | 2185978 | 2186717 | 2186843 | 98 | 43 | 132 | 17 | 238 | 119 | 0.001078471 | 0.533 | 0.795 | -0.262 |  |  |
|  |  | 105064052 | - | 7574546 | 7574822 | 7573668 | 7573938 | 7575001 | 7575289 | 2 | 6 | 12 | 0 | 397 | 135 | 0.001085984 | 0.102 | 1 | -0.898 |  |  |
|  |  | 105070813 | + | 1165592 | 1165705 | 1165246 | 1165296 | 1165807 | 1166073 | 300 | 36 | 1136 | 41 | 120 | 43 | 0.001246899 | 0.749 | 0.908 | -0.159 |  |  |
|  |  | 105062911 | + | 1109742 | 1109874 | 1109467 | 1109617 | 1110463 | 1110595 | 0 | 5 | 11 | 1 | 240 | 125 | 0.001297719 | 0 | 0.851 | -0.851 |  |  |
|  |  | 105079822 | - | 3103774 | 3103894 | 3103406 | 3103529 | 3105188 | 3105251 | 86 | 86 | 178 | 73 | 128 | 37 | 0.001337412 | 0.224 | 0.413 | -0.189 |  |  |
|  |  | 105068902 | + | 971192 | 971258 | 970241 | 970329 | 974015 | 974235 | 147 | 237 | 106 | 346 | 64 | 81 | 0.00139776 | 0.44 | 0.279 | 0.161 |  |  |
|  |  | 105076213 | - | 4043391 | 4043508 | 4041786 | 4042707 | 4043611 | 4043735 | 11 | 10 | 39 | 3 | 202 | 117 | 0.001446132 | 0.389 | 0.883 | -0.494 |  |  |
|  |  | 105081954 | - | 7121131 | 7121301 | 7009045 | 7012131 | 7122092 | 7122231 | 22 | 1 | 8 | 9 | 288 | 132 | 0.001480819 | 0.91 | 0.289 | 0.621 |  |  |
|  |  | 105080897 | - | 50530 | 50702 | 49210 | 49345 | 63418 | 63482 | 90 | 7 | 64 | 24 | 208 | 50 | 0.001571868 | 0.756 | 0.391 | 0.365 |  |  |
|  |  | 105065177 | + | 160537 | 160631 | 155349 | 155669 | 163100 | 163208 | 60 | 19 | 93 | 4 | 140 | 101 | 0.001624824 | 0.695 | 0.944 | -0.249 |  |  |
|  |  | 105081318 | - | 2958892 | 2958993 | 2953157 | 2954376 | 2961264 | 2961408 | 60 | 48 | 47 | 9 | 188 | 135 | 0.001702666 | 0.473 | 0.789 | -0.316 |  |  |
|  |  | 105083251 | - | 256993 | 257143 | 255226 | 255379 | 262603 | 262714 | 42 | 9 | 62 | 0 | 240 | 104 | 0.001708625 | 0.669 | 1 | -0.331 |  |  |
|  |  | 105082028 | - | 6868714 | 6868764 | 6863994 | 6864121 | 6869699 | 6869813 | 183 | 23 | 77 | 41 | 43 | 92 | 0.001737597 | 0.945 | 0.801 | 0.144 |  |  |
|  |  | 105084146 | - | 1796573 | 1796738 | 1796229 | 1796250 | 1797223 | 1797409 | 811 | 28 | 480 | 2 | 165 | 14 | 0.001942018 | 0.711 | 0.953 | -0.242 |  |  |
|  |  | 105082303 | - | 3042766 | 3042846 | 3041884 | 3042026 | 3047152 | 3047215 | 31 | 224 | 72 | 175 | 73 | 56 | 0.001942343 | 0.096 | 0.24 | -0.144 |  |  |
|  |  | 105084146 | - | 1796573 | 1796738 | 1796113 | 1796140 | 1797223 | 1797409 | 719 | 19 | 428 | 0 | 171 | 20 | 0.001987011 | 0.816 | 1 | -0.184 |  |  |
|  |  | 105068790 | + | 1128484 | 1129036 | 1127768 | 1128102 | 1130148 | 1131148 | 180 | 12 | 173 | 0 | 673 | 135 | 0.002002626 | 0.751 | 1 | -0.249 |  |  |
|  |  | 105075143 | - | 516932 | 517172 | 515507 | 516295 | 517497 | 517848 | 16 | 11 | 35 | 2 | 361 | 135 | 0.002019799 | 0.352 | 0.867 | -0.515 |  |  |
|  |  | 105074547 | - | 7165329 | 7165491 | 7150461 | 7150539 | 7175289 | 7175449 | 307 | 19 | 151 | 31 | 219 | 71 | 0.002181459 | 0.84 | 0.612 | 0.228 |  |  |
|  |  | 105069318 | - | 35836 | 35930 | 24963 | 25073 | 40465 | 40659 | 12 | 0 | 3 | 6 | 142 | 103 | 0.002210318 | 1 | 0.266 | 0.734 |  |  |
|  |  | 105075245 | - | 2174459 | 2174580 | 2158527 | 2159162 | 2191132 | 2191218 | 14 | 8 | 37 | 1 | 172 | 79 | 0.00227156 | 0.446 | 0.944 | -0.498 |  |  |
|  |  | 105062366 | - | 972327 | 972483 | 971418 | 971796 | 974969 | 975055 | 24 | 6 | 62 | 0 | 221 | 79 | 0.002273768 | 0.588 | 1 | -0.412 |  |  |
|  |  | 105080161 | + | 404712 | 404778 | 404056 | 404194 | 404974 | 405001 | 236 | 289 | 296 | 783 | 55 | 16 | 0.002519588 | 0.192 | 0.099 | 0.093 |  |  |
|  |  | 105068149 | + | 1237448 | 1237628 | 1226705 | 1227444 | 1254306 | 1254401 | 31 | 15 | 48 | 3 | 254 | 88 | 0.00265025 | 0.417 | 0.847 | -0.43 |  |  |
|  |  | 105080789 | - | 10597932 | 10598010 | 10587797 | 10587967 | 10621073 | 10621356 | 93 | 4 | 43 | 15 | 142 | 135 | 0.002865143 | 0.957 | 0.732 | 0.225 |  |  |
|  |  | 105073708 | - | 5257726 | 5257797 | 5256602 | 5256787 | 5258190 | 5258256 | 596 | 660 | 1072 | 781 | 64 | 59 | 0.002967071 | 0.454 | 0.559 | -0.105 |  |  |
|  |  | 105066512 | - | 3948116 | 3948291 | 3941833 | 3942033 | 3952046 | 3952153 | 9 | 1 | 1 | 7 | 261 | 100 | 0.002993633 | 0.775 | 0.052 | 0.723 |  |  |
|  |  | 105066370 | - | 2378525 | 2378664 | 2377826 | 2378011 | 2379176 | 2379307 | 56 | 14 | 143 | 6 | 253 | 124 | 0.003984331 | 0.662 | 0.921 | -0.259 |  |  |
|  |  | 105066407 | - | 38075 | 38151 | 30040 | 30329 | 39907 | 40079 | 30 | 39 | 34 | 11 | 138 | 135 | 0.004140944 | 0.429 | 0.751 | -0.322 |  |  |
|  |  | 105079841 | + | 2968 | 3072 | 3 | 90 | 6030 | 6136 | 65 | 35 | 86 | 14 | 103 | 44 | 0.004393308 | 0.442 | 0.724 | -0.282 |  |  |
|  |  | 105081846 | + | 12246363 | 12246467 | 12236808 | 12237057 | 12253572 | 12253832 | 209 | 30 | 75 | 32 | 194 | 135 | 0.004830678 | 0.829 | 0.62 | 0.209 |  |  |
|  |  | 105080175 | - | 639501 | 639688 | 639038 | 639197 | 640147 | 640259 | 125 | 13 | 209 | 2 | 278 | 105 | 0.005313568 | 0.784 | 0.975 | -0.191 |  |  |
|  |  | 105065207 | - | 3917865 | 3917918 | 3916736 | 3916947 | 3919131 | 3919191 | 12 | 0 | 3 | 5 | 46 | 53 | 0.005449371 | 1 | 0.409 | 0.591 |  |  |
|  |  | 105072173 | + | 5021759 | 5021866 | 5021424 | 5021595 | 5022688 | 5025116 | 4 | 9 | 7 | 0 | 200 | 135 | 0.005576932 | 0.231 | 1 | -0.769 |  |  |
|  |  | 105072173 | + | 5021762 | 5021866 | 5021424 | 5021595 | 5022688 | 5025116 | 3 | 9 | 6 | 0 | 194 | 135 | 0.005907229 | 0.188 | 1 | -0.812 |  |  |
|  |  | 105081766 | - | 5884743 | 5884843 | 5842353 | 5844167 | 5898715 | 5898737 | 9 | 1 | 0 | 4 | 93 | 15 | 0.006375629 | 0.592 | 0 | 0.592 |  |  |
|  |  | 105070278 | + | 9343994 | 9344048 | 9336223 | 9336445 | 9350690 | 9350791 | 50 | 35 | 147 | 32 | 53 | 94 | 0.006411161 | 0.717 | 0.891 | -0.174 |  |  |
|  |  | 105076509 | - | 10236076 | 10236283 | 10233378 | 10233444 | 10256383 | 10256614 | 422 | 79 | 205 | 77 | 252 | 59 | 0.006606671 | 0.556 | 0.384 | 0.172 |  |  |
|  |  | 105078576 | - | 1331837 | 1331941 | 1322143 | 1322239 | 1425186 | 1425338 | 47 | 8 | 70 | 0 | 148 | 89 | 0.006843474 | 0.779 | 1 | -0.221 |  |  |
|  |  | 105069873 | + | 1391645 | 1391757 | 1390425 | 1390590 | 1403350 | 1403547 | 30 | 7 | 50 | 0 | 210 | 135 | 0.006852514 | 0.734 | 1 | -0.266 |  |  |
|  |  | 105082187 | - | 27771971 | 27772198 | 27771657 | 27771841 | 27772640 | 27772779 | 35 | 0 | 48 | 11 | 345 | 132 | 0.007149737 | 1 | 0.625 | 0.375 |  |  |
|  |  | 105066110 | - | 442460 | 442574 | 349603 | 349692 | 456797 | 457105 | 6 | 2 | 0 | 7 | 161 | 82 | 0.007278083 | 0.604 | 0 | 0.604 |  |  |
|  |  | 105063978 | - | 4005730 | 4005817 | 4000440 | 4000515 | 4006082 | 4006169 | 39 | 51 | 55 | 24 | 38 | 13 | 0.007637036 | 0.207 | 0.439 | -0.232 |  |  |
|  |  | 105069296 | + | 1072741 | 1072762 | 1066635 | 1066761 | 1075381 | 1075517 | 10 | 272 | 1 | 345 | 8 | 113 | 0.007985137 | 0.342 | 0.039 | 0.303 |  |  |
|  |  | 105077668 | + | 464789 | 464897 | 458186 | 458296 | 473702 | 473796 | 30 | 20 | 22 | 53 | 122 | 55 | 0.008058211 | 0.403 | 0.158 | 0.245 |  |  |
|  |  | 105075513 | + | 8828105 | 8828165 | 8822200 | 8822275 | 8828245 | 8828391 | 8 | 34 | 1 | 87 | 53 | 68 | 0.008099841 | 0.232 | 0.015 | 0.217 |  |  |
|  |  | 105064554 | + | 53512 | 53566 | 50025 | 52866 | 54041 | 54228 | 5 | 0 | 0 | 4 | 94 | 135 | 0.008369206 | 1 | 0 | 1 |  |  |
|  |  | 105076296 | - | 1659744 | 1659828 | 1658112 | 1658190 | 1660958 | 1662391 | 2 | 5 | 8 | 0 | 90 | 71 | 0.008395138 | 0.24 | 1 | -0.76 |  |  |
|  |  | 105072690 | - | 4116135 | 4116243 | 4111148 | 4111252 | 4116689 | 4116800 | 43 | 21 | 18 | 31 | 133 | 66 | 0.008482725 | 0.504 | 0.224 | 0.28 |  |  |
|  |  | 105078056 | + | 869962 | 870104 | 867417 | 867547 | 877712 | 878602 | 11 | 0 | 10 | 9 | 258 | 123 | 0.008490346 | 1 | 0.346 | 0.654 |  |  |
|  |  | 105073080 | + | 5083794 | 5083920 | 5061732 | 5061799 | 5121651 | 5126007 | 54 | 5 | 49 | 21 | 163 | 60 | 0.00858911 | 0.799 | 0.462 | 0.337 |  |  |
|  |  | 105069860 | + | 271220 | 271286 | 270745 | 270811 | 273680 | 273807 | 4 | 2 | 0 | 11 | 44 | 44 | 0.008610347 | 0.667 | 0 | 0.667 |  |  |
|  |  | 105082012 | + | 406276 | 406600 | 394748 | 394814 | 414488 | 414618 | 72 | 22 | 72 | 5 | 357 | 47 | 0.008962355 | 0.301 | 0.655 | -0.354 |  |  |
|  |  | 105066054 | - | 2948288 | 2948478 | 2947729 | 2948113 | 2950468 | 2950701 | 41 | 6 | 82 | 0 | 311 | 135 | 0.009085163 | 0.748 | 1 | -0.252 |  |  |
|  |  | 105076466 | + | 1892999 | 1893086 | 1868848 | 1869859 | 1897200 | 1897380 | 4 | 11 | 24 | 9 | 160 | 135 | 0.009314363 | 0.235 | 0.692 | -0.457 |  |  |
|  |  | 105083805 | + | 3585788 | 3585953 | 3576981 | 3577126 | 3592442 | 3592540 | 17 | 2 | 2 | 5 | 242 | 91 | 0.00940231 | 0.762 | 0.131 | 0.631 |  |  |
|  |  | 105066004 | - | 1097103 | 1097251 | 1095102 | 1095252 | 1099436 | 1099495 | 225 | 22 | 245 | 5 | 187 | 52 | 0.0095173 | 0.74 | 0.932 | -0.192 |  |  |
|  |  | 105063642 | - | 383493 | 383600 | 381602 | 381693 | 385376 | 385536 | 41 | 0 | 17 | 5 | 149 | 84 | 0.009574534 | 1 | 0.657 | 0.343 |  |  |
|  |  | 105073692 | + | 3401363 | 3401783 | 3399127 | 3399210 | 3403002 | 3403118 | 0 | 2 | 14 | 0 | 456 | 50 | 0.009662026 | 0 | 1 | -1 |  |  |
|  |  | 105074539 | + | 7004275 | 7004386 | 6992787 | 6992855 | 7009805 | 7009931 | 32 | 0 | 15 | 5 | 118 | 45 | 0.009767679 | 1 | 0.534 | 0.466 |  |  |
|  |  | 105064019 | - | 6862017 | 6862201 | 6860138 | 6860452 | 6862435 | 6862457 | 7 | 8 | 11 | 0 | 185 | 15 | 0.009815908 | 0.066 | 1 | -0.934 |  |  |
|  |  | 105066094 | + | 4116440 | 4116536 | 4115962 | 4116085 | 4117817 | 4117942 | 0 | 24 | 6 | 11 | 142 | 99 | 0.010204377 | 0 | 0.276 | -0.276 |  |  |
|  |  | 105063949 | + | 4931014 | 4931203 | 4921398 | 4921602 | 4935539 | 4935727 | 6 | 14 | 16 | 5 | 310 | 135 | 0.010330706 | 0.157 | 0.582 | -0.425 |  |  |
|  |  | 105068821 | + | 4809923 | 4809956 | 4809645 | 4809783 | 4811783 | 4812148 | 37 | 1 | 62 | 27 | 48 | 131 | 0.010439126 | 0.99 | 0.862 | 0.128 |  |  |
|  |  | 105066915 | - | 768868 | 768955 | 764035 | 765767 | 770131 | 770297 | 15 | 15 | 21 | 3 | 160 | 135 | 0.010578276 | 0.458 | 0.855 | -0.397 |  |  |
|  |  | 105079837 | - | 2090448 | 2090556 | 2084377 | 2084512 | 2093612 | 2093743 | 2 | 11 | 7 | 2 | 184 | 117 | 0.010724729 | 0.104 | 0.69 | -0.586 |  |  |
|  |  | 105079018 | - | 7340396 | 7340474 | 7339389 | 7339873 | 7350850 | 7350973 | 37 | 104 | 47 | 52 | 123 | 116 | 0.010769222 | 0.251 | 0.46 | -0.209 |  |  |
|  |  | 105072889 | - | 1775253 | 1775292 | 1771504 | 1771615 | 1794755 | 1794898 | 14 | 6 | 7 | 20 | 33 | 104 | 0.011091135 | 0.88 | 0.524 | 0.356 |  |  |
|  |  | 105081390 | + | 564613 | 564667 | 560711 | 560837 | 564930 | 565011 | 20 | 12 | 38 | 80 | 31 | 58 | 0.011096082 | 0.757 | 0.471 | 0.286 |  |  |
|  |  | 105076986 | - | 9837342 | 9837514 | 9837077 | 9837197 | 9840365 | 9840572 | 54 | 15 | 39 | 1 | 271 | 113 | 0.011197652 | 0.6 | 0.942 | -0.342 |  |  |
|  |  | 105070102 | - | 1705968 | 1706149 | 1703913 | 1704021 | 1706297 | 1706417 | 42 | 35 | 33 | 7 | 246 | 79 | 0.011348541 | 0.278 | 0.602 | -0.324 |  |  |
|  |  | 105083805 | + | 3585788 | 3585918 | 3576981 | 3577126 | 3592442 | 3592540 | 16 | 2 | 2 | 5 | 202 | 91 | 0.011410896 | 0.783 | 0.153 | 0.63 |  |  |
|  |  | 105074560 | + | 224735 | 225239 | 219335 | 219659 | 227251 | 227803 | 11 | 4 | 31 | 0 | 625 | 135 | 0.011684062 | 0.373 | 1 | -0.627 |  |  |
|  |  | 105079159 | - | 164371 | 164519 | 159215 | 159367 | 164971 | 165032 | 69 | 0 | 46 | 6 | 189 | 54 | 0.012134707 | 1 | 0.687 | 0.313 |  |  |
|  |  | 105067849 | + | 4181152 | 4181185 | 4170535 | 4170631 | 4200736 | 4200868 | 15 | 2 | 19 | 25 | 16 | 79 | 0.012590299 | 0.974 | 0.79 | 0.184 |  |  |
|  |  | 105078478 | + | 11167844 | 11167897 | 11144392 | 11144557 | 11171000 | 11172283 | 9 | 6 | 17 | 0 | 92 | 135 | 0.012827858 | 0.688 | 1 | -0.312 |  |  |
|  |  | 105080789 | - | 10598868 | 10599135 | 10597932 | 10598010 | 10621073 | 10621356 | 109 | 31 | 117 | 11 | 324 | 71 | 0.012860374 | 0.435 | 0.7 | -0.265 |  |  |
|  |  | 105063526 | - | 243501 | 243665 | 242256 | 242459 | 249172 | 249301 | 37 | 0 | 25 | 6 | 272 | 122 | 0.01288014 | 1 | 0.651 | 0.349 |  |  |
|  |  | 105067321 | + | 11920371 | 11922465 | 11881948 | 11882203 | 11927057 | 11927687 | 232 | 12 | 450 | 5 | 2215 | 135 | 0.01298267 | 0.541 | 0.846 | -0.305 |  |  |
|  |  | 105073643 | - | 5776982 | 5777113 | 5775083 | 5776007 | 5781842 | 5782009 | 4 | 5 | 10 | 0 | 248 | 135 | 0.013108154 | 0.303 | 1 | -0.697 |  |  |
|  |  | 105075963 | - | 167915 | 168062 | 155424 | 155577 | 177328 | 177420 | 42 | 13 | 21 | 0 | 220 | 85 | 0.013325484 | 0.555 | 1 | -0.445 |  |  |
|  |  | 105078648 | - | 2053152 | 2053409 | 2050795 | 2050922 | 2060811 | 2060951 | 300 | 25 | 405 | 8 | 361 | 118 | 0.013554997 | 0.797 | 0.943 | -0.146 |  |  |
|  |  | 105065282 | + | 850360 | 850564 | 847965 | 848151 | 851753 | 851963 | 723 | 65 | 2220 | 79 | 325 | 135 | 0.013992329 | 0.822 | 0.921 | -0.099 |  |  |
|  |  | 105072448 | - | 507761 | 507935 | 505725 | 505796 | 508335 | 508548 | 157 | 12 | 132 | 30 | 224 | 64 | 0.013999398 | 0.789 | 0.557 | 0.232 |  |  |
|  |  | 105072757 | - | 2774854 | 2774940 | 2773277 | 2773404 | 2777219 | 2777492 | 4 | 0 | 0 | 4 | 143 | 120 | 0.0140267 | 1 | 0 | 1 |  |  |
|  |  | 105068459 | - | 418651 | 418755 | 418019 | 418507 | 418844 | 418979 | 0 | 8 | 14 | 14 | 187 | 128 | 0.014094007 | 0 | 0.406 | -0.406 |  |  |
|  |  | 105064422 | + | 11781583 | 11781699 | 11780089 | 11780277 | 11789611 | 11789715 | 4 | 0 | 0 | 4 | 180 | 97 | 0.014162039 | 1 | 0 | 1 |  |  |
|  |  | 105082506 | - | 2030107 | 2030898 | 2023586 | 2023731 | 2038092 | 2038203 | 250 | 8 | 257 | 0 | 881 | 104 | 0.014790884 | 0.787 | 1 | -0.213 |  |  |
|  |  | 105079119 | + | 923645 | 923717 | 923313 | 923408 | 925302 | 926728 | 0 | 21 | 7 | 17 | 83 | 88 | 0.015286481 | 0 | 0.304 | -0.304 |  |  |
|  |  | 105076595 | - | 22989911 | 22989981 | 22986855 | 22987009 | 22991311 | 22991430 | 66 | 50 | 51 | 88 | 103 | 112 | 0.01548164 | 0.589 | 0.387 | 0.202 |  |  |
|  |  | 105079016 | - | 9212077 | 9212618 | 9207279 | 9207402 | 9213899 | 9214051 | 76 | 51 | 67 | 16 | 643 | 116 | 0.015629165 | 0.212 | 0.43 | -0.218 |  |  |
|  |  | 105080389 | - | 4782203 | 4782301 | 4779413 | 4779628 | 4785343 | 4785476 | 13 | 3 | 4 | 9 | 173 | 126 | 0.016406291 | 0.759 | 0.245 | 0.514 |  |  |
|  |  | 105072315 | + | 898810 | 899374 | 818993 | 819061 | 904909 | 905093 | 195 | 7 | 203 | 0 | 611 | 61 | 0.017554083 | 0.736 | 1 | -0.264 |  |  |
|  |  | 105082014 | + | 554966 | 555026 | 553133 | 553196 | 556582 | 556723 | 3 | 3 | 0 | 18 | 52 | 55 | 0.017868715 | 0.514 | 0 | 0.514 |  |  |
|  |  | 105071932 | - | 12355973 | 12356263 | 12354868 | 12354928 | 12360032 | 12360163 | 141 | 7 | 109 | 19 | 318 | 42 | 0.017943291 | 0.727 | 0.431 | 0.296 |  |  |
|  |  | 105063620 | - | 4173754 | 4173913 | 4141074 | 4141236 | 4193089 | 4193258 | 22 | 0 | 17 | 6 | 280 | 135 | 0.017967612 | 1 | 0.577 | 0.423 |  |  |
|  |  | 105065335 | + | 4265812 | 4266005 | 4260876 | 4260928 | 4300910 | 4301075 | 168 | 26 | 197 | 10 | 224 | 45 | 0.017994398 | 0.565 | 0.798 | -0.233 |  |  |
|  |  | 105067148 | - | 2952874 | 2953011 | 2801744 | 2801905 | 2996395 | 2996423 | 539 | 13 | 360 | 0 | 146 | 21 | 0.01800702 | 0.856 | 1 | -0.144 |  |  |
|  |  | 105076934 | - | 7783655 | 7783721 | 7777877 | 7777942 | 7790732 | 7790844 | 11 | 0 | 0 | 2 | 29 | 28 | 0.018093835 | 1 | 0 | 1 |  |  |
|  |  | 105066065 | - | 3308871 | 3309030 | 3308341 | 3308512 | 3314522 | 3314620 | 17 | 9 | 37 | 3 | 236 | 91 | 0.018788617 | 0.421 | 0.826 | -0.405 |  |  |
|  |  | 105077732 | - | 2083012 | 2083166 | 2060217 | 2060348 | 2087078 | 2087160 | 28 | 7 | 27 | 0 | 204 | 64 | 0.018902304 | 0.557 | 1 | -0.443 |  |  |
|  |  | 105063335 | + | 9701127 | 9701196 | 9693353 | 9693439 | 9703903 | 9703967 | 1 | 4 | 6 | 0 | 6 | 1 | 0.019036136 | 0.04 | 1 | -0.96 |  |  |
|  |  | 105061921 | - | 4648151 | 4648211 | 4635158 | 4635305 | 4650357 | 4650548 | 0 | 10 | 2 | 0 | 106 | 135 | 0.019241313 | 0 | 1 | -1 |  |  |
|  |  | 105072299 | + | 1696 | 1783 | 1281 | 1399 | 2886 | 3063 | 31 | 11 | 135 | 10 | 136 | 111 | 0.019271931 | 0.697 | 0.917 | -0.22 |  |  |
|  |  | 105063949 | + | 4922111 | 4922300 | 4921398 | 4921602 | 4935539 | 4935727 | 20 | 14 | 34 | 5 | 310 | 135 | 0.019303925 | 0.384 | 0.748 | -0.364 |  |  |
|  |  | 105063278 | - | 6553369 | 6553580 | 6544012 | 6547403 | 6555470 | 6555656 | 125 | 17 | 267 | 9 | 332 | 135 | 0.0194594 | 0.749 | 0.923 | -0.174 |  |  |
|  |  | 105079554 | + | 4165258 | 4165451 | 4163911 | 4164232 | 4166532 | 4166715 | 601 | 566 | 419 | 623 | 314 | 135 | 0.019478567 | 0.313 | 0.224 | 0.089 |  |  |
|  |  | 105075338 | - | 4821328 | 4821409 | 4785721 | 4785830 | 4842457 | 4842553 | 5 | 6 | 8 | 0 | 69 | 56 | 0.019912597 | 0.403 | 1 | -0.597 |  |  |
|  |  | 105068836 | - | 653963 | 654021 | 653712 | 653768 | 656295 | 656500 | 32 | 8 | 11 | 13 | 51 | 49 | 0.019991604 | 0.794 | 0.448 | 0.346 |  |  |
|  |  | 105082108 | - | 22473358 | 22473367 | 22470210 | 22470367 | 22477224 | 22477323 | 2 | 0 | 0 | 13 | 2 | 92 | 0.020000103 | 1 | 0 | 1 |  |  |
|  |  | 105067239 | + | 5948538 | 5948732 | 5939613 | 5940171 | 5954092 | 5954465 | 66 | 7 | 83 | 0 | 315 | 135 | 0.020108368 | 0.802 | 1 | -0.198 |  |  |
|  |  | 105062022 | + | 1480760 | 1480851 | 1479443 | 1479666 | 1481247 | 1481314 | 14 | 5 | 24 | 0 | 93 | 60 | 0.020835491 | 0.644 | 1 | -0.356 |  |  |
|  |  | 105067006 | - | 1540195 | 1540279 | 1531582 | 1531692 | 1541002 | 1541162 | 0 | 3 | 17 | 4 | 122 | 103 | 0.022047871 | 0 | 0.782 | -0.782 |  |  |
|  |  | 105064372 | - | 5597650 | 5597683 | 5594950 | 5595047 | 5599850 | 5600017 | 7 | 1 | 2 | 7 | 26 | 90 | 0.022085518 | 0.96 | 0.497 | 0.463 |  |  |
|  |  | 105073045 | + | 3309848 | 3309971 | 3307225 | 3307344 | 3310462 | 3310659 | 83 | 13 | 146 | 4 | 209 | 112 | 0.023255572 | 0.774 | 0.951 | -0.177 |  |  |
|  |  | 105077924 | - | 4371000 | 4371096 | 4367907 | 4368094 | 4383585 | 4383649 | 39 | 1 | 27 | 8 | 100 | 57 | 0.023416722 | 0.957 | 0.658 | 0.299 |  |  |
|  |  | 105067325 | - | 11980914 | 11981043 | 11980521 | 11980751 | 11981288 | 11981359 | 0 | 2 | 8 | 0 | 173 | 64 | 0.023489921 | 0 | 1 | -1 |  |  |
|  |  | 105064212 | - | 3484303 | 3484445 | 3483382 | 3483582 | 3484952 | 3485097 | 0 | 2 | 8 | 0 | 270 | 135 | 0.024070346 | 0 | 1 | -1 |  |  |
|  |  | 105073854 | - | 900611 | 900719 | 900415 | 900529 | 901229 | 901293 | 32 | 4 | 77 | 0 | 96 | 29 | 0.024272188 | 0.707 | 1 | -0.293 |  |  |
|  |  | 105073604 | + | 5681814 | 5681917 | 5622533 | 5622639 | 5690989 | 5691182 | 33 | 0 | 26 | 6 | 156 | 99 | 0.024351348 | 1 | 0.733 | 0.267 |  |  |
|  |  | 105083442 | + | 1474886 | 1475073 | 1474680 | 1474765 | 1476665 | 1477091 | 13 | 4 | 52 | 1 | 251 | 78 | 0.0243571 | 0.502 | 0.942 | -0.44 |  |  |
|  |  | 105066143 | - | 3713489 | 3713554 | 3708454 | 3708610 | 3716489 | 3716588 | 18 | 13 | 15 | 1 | 73 | 92 | 0.024472891 | 0.636 | 0.95 | -0.314 |  |  |
|  |  | 105079442 | + | 11039511 | 11039571 | 11037737 | 11038127 | 11042751 | 11043153 | 1 | 5 | 4 | 0 | 106 | 135 | 0.024485119 | 0.203 | 1 | -0.797 |  |  |
|  |  | 105073702 | + | 5072689 | 5072790 | 5071546 | 5071703 | 5073033 | 5073115 | 114 | 2 | 30 | 6 | 128 | 75 | 0.024716429 | 0.971 | 0.746 | 0.225 |  |  |
|  |  | 105080019 | + | 11797964 | 11798147 | 11762405 | 11762671 | 11809517 | 11810336 | 82 | 8 | 81 | 0 | 304 | 135 | 0.024868294 | 0.82 | 1 | -0.18 |  |  |
|  |  | 105082179 | + | 27648460 | 27649550 | 27637511 | 27637600 | 27654473 | 27654535 | 3575 | 12 | 2958 | 28 | 1078 | 2 | 0.025075939 | 0.356 | 0.164 | 0.192 |  |  |
|  |  | 105073541 | - | 2986892 | 2986970 | 2967224 | 2967315 | 2990736 | 2990797 | 14 | 0 | 9 | 5 | 20 | 3 | 0.025217528 | 1 | 0.213 | 0.787 |  |  |
|  |  | 105082128 | + | 23936390 | 23936519 | 23933593 | 23933711 | 23937814 | 23940592 | 21 | 8 | 16 | 0 | 220 | 111 | 0.025767292 | 0.57 | 1 | -0.43 |  |  |
|  |  | 105063679 | - | 1624852 | 1624981 | 1619671 | 1619816 | 1625617 | 1625754 | 44 | 23 | 28 | 40 | 239 | 130 | 0.026067302 | 0.51 | 0.276 | 0.234 |  |  |
|  |  | 105076527 | + | 11898061 | 11898215 | 11895678 | 11895874 | 11898935 | 11899030 | 31 | 6 | 65 | 1 | 228 | 88 | 0.026434283 | 0.666 | 0.962 | -0.296 |  |  |
|  |  | 105074416 | - | 3072034 | 3072157 | 3068164 | 3071382 | 3072691 | 3072818 | 10 | 0 | 4 | 4 | 217 | 120 | 0.026467998 | 1 | 0.356 | 0.644 |  |  |
|  |  | 105078224 | - | 2938563 | 2938644 | 2937544 | 2937740 | 2939425 | 2939499 | 166 | 43 | 93 | 7 | 80 | 67 | 0.026806019 | 0.764 | 0.918 | -0.154 |  |  |
|  |  | 105063418 | - | 701898 | 702006 | 700849 | 701037 | 702538 | 702620 | 5 | 24 | 15 | 14 | 142 | 75 | 0.027263167 | 0.099 | 0.361 | -0.262 |  |  |
|  |  | 105070082 | - | 942501 | 942705 | 940478 | 940580 | 947021 | 947156 | 134 | 9 | 208 | 1 | 278 | 88 | 0.028312084 | 0.825 | 0.985 | -0.16 |  |  |
|  |  | 105075052 | - | 6850840 | 6850892 | 6836456 | 6837190 | 6852372 | 6852554 | 2 | 0 | 4 | 35 | 90 | 135 | 0.028584423 | 1 | 0.146 | 0.854 |  |  |
|  |  | 105081724 | - | 3307199 | 3307280 | 3301470 | 3301595 | 3311831 | 3311917 | 11 | 5 | 17 | 0 | 75 | 62 | 0.028604616 | 0.645 | 1 | -0.355 |  |  |
|  |  | 105081338 | - | 4628884 | 4628995 | 4624865 | 4625024 | 4629839 | 4630466 | 54 | 11 | 73 | 2 | 208 | 135 | 0.028740536 | 0.761 | 0.959 | -0.198 |  |  |
|  |  | 105065767 | - | 402746 | 402940 | 401765 | 401941 | 406334 | 406481 | 16 | 0 | 15 | 6 | 315 | 135 | 0.028994314 | 1 | 0.517 | 0.483 |  |  |
|  |  | 105067177 | + | 96244 | 96371 | 89039 | 89129 | 102796 | 105637 | 7 | 13 | 7 | 1 | 188 | 83 | 0.029098406 | 0.192 | 0.756 | -0.564 |  |  |
|  |  | 105083442 | + | 1474868 | 1475073 | 1474684 | 1474765 | 1476665 | 1477091 | 11 | 4 | 40 | 1 | 265 | 74 | 0.029194835 | 0.434 | 0.918 | -0.484 |  |  |
|  |  | 105077185 | + | 15395524 | 15395649 | 15394013 | 15394194 | 15397208 | 15397838 | 194 | 16 | 334 | 3 | 236 | 135 | 0.030655482 | 0.874 | 0.985 | -0.111 |  |  |
|  |  | 105061747 | + | 1949492 | 1949659 | 1947551 | 1947723 | 1951009 | 1951864 | 147 | 0 | 103 | 8 | 288 | 135 | 0.031039104 | 1 | 0.858 | 0.142 |  |  |
|  |  | 105082808 | + | 6579661 | 6579785 | 6579334 | 6579421 | 6580092 | 6580227 | 13 | 0 | 1 | 2 | 172 | 73 | 0.031111458 | 1 | 0.175 | 0.825 |  |  |
|  |  | 105080170 | + | 538989 | 539085 | 534799 | 534885 | 539880 | 539972 | 6 | 0 | 3 | 5 | 72 | 29 | 0.031231241 | 1 | 0.195 | 0.805 |  |  |
|  |  | 105074944 | + | 787054 | 787273 | 784097 | 784233 | 788495 | 788687 | 82 | 21 | 110 | 9 | 334 | 129 | 0.03148579 | 0.601 | 0.825 | -0.224 |  |  |
|  |  | 105071923 | + | 11883675 | 11884039 | 11849331 | 11849535 | 11885234 | 11885377 | 120 | 6 | 41 | 9 | 485 | 135 | 0.031863409 | 0.848 | 0.559 | 0.289 |  |  |
|  |  | 105078971 | + | 3870013 | 3870292 | 3863941 | 3864088 | 3875301 | 3875478 | 72 | 16 | 75 | 4 | 400 | 135 | 0.032303703 | 0.603 | 0.864 | -0.261 |  |  |
|  |  | 105070311 | + | 12809215 | 12810677 | 12803470 | 12803896 | 12819059 | 12819128 | 441 | 12 | 622 | 4 | 1510 | 62 | 0.032311196 | 0.601 | 0.865 | -0.264 |  |  |
|  |  | 105066790 | + | 2316693 | 2316930 | 2315324 | 2315621 | 2329095 | 2329363 | 48 | 18 | 103 | 13 | 358 | 135 | 0.032554441 | 0.501 | 0.749 | -0.248 |  |  |
|  |  | 105066204 | + | 8940486 | 8940513 | 8938873 | 8939031 | 8941083 | 8941116 | 6 | 2 | 0 | 4 | 20 | 26 | 0.032592475 | 0.796 | 0 | 0.796 |  |  |
|  |  | 105069866 | + | 1121080 | 1121110 | 1119359 | 1119515 | 1121606 | 1121736 | 2 | 8 | 0 | 77 | 34 | 123 | 0.032645907 | 0.475 | 0 | 0.475 |  |  |
|  |  | 105063641 | - | 179438 | 179537 | 178798 | 178905 | 180332 | 180441 | 7 | 40 | 13 | 16 | 116 | 67 | 0.033014042 | 0.092 | 0.319 | -0.227 |  |  |
|  |  | 105064626 | - | 759308 | 759372 | 735444 | 735646 | 760491 | 760532 | 15 | 0 | 11 | 5 | 57 | 34 | 0.033216566 | 1 | 0.568 | 0.432 |  |  |
|  |  | 105069555 | - | 2271218 | 2271364 | 2271010 | 2271127 | 2273295 | 2273426 | 116 | 115 | 184 | 100 | 234 | 99 | 0.033272964 | 0.299 | 0.438 | -0.139 |  |  |
|  |  | 105073604 | + | 5690989 | 5691182 | 5681814 | 5681917 | 5693045 | 5693141 | 28 | 4 | 48 | 0 | 229 | 50 | 0.0337778 | 0.604 | 1 | -0.396 |  |  |
|  |  | 105062081 | + | 86868 | 87015 | 82994 | 83168 | 90271 | 90428 | 9 | 0 | 12 | 8 | 270 | 135 | 0.033785705 | 1 | 0.429 | 0.571 |  |  |
|  |  | 105074098 | + | 864676 | 864853 | 863867 | 864064 | 865177 | 866080 | 2 | 5 | 11 | 2 | 298 | 135 | 0.033835987 | 0.153 | 0.714 | -0.561 |  |  |
|  |  | 105079995 | + | 12845594 | 12845645 | 12844024 | 12844177 | 12845724 | 12847793 | 25 | 12 | 9 | 17 | 88 | 135 | 0.033892615 | 0.762 | 0.448 | 0.314 |  |  |
|  |  | 105063480 | - | 2459442 | 2459550 | 2458534 | 2458654 | 2459858 | 2459949 | 13 | 3 | 48 | 0 | 129 | 62 | 0.034507208 | 0.676 | 1 | -0.324 |  |  |
|  |  | 105083028 | + | 8732004 | 8732087 | 8731003 | 8731107 | 8733220 | 8733353 | 22 | 0 | 15 | 5 | 105 | 88 | 0.034603658 | 1 | 0.715 | 0.285 |  |  |
|  |  | 105070366 | + | 14349284 | 14349334 | 14348484 | 14348613 | 14350610 | 14350710 | 8 | 1 | 7 | 11 | 31 | 80 | 0.034797803 | 0.954 | 0.622 | 0.332 |  |  |
|  |  | 105066491 | - | 72938 | 73009 | 70730 | 70895 | 73133 | 73224 | 10 | 7 | 17 | 1 | 77 | 84 | 0.034870811 | 0.609 | 0.949 | -0.34 |  |  |
|  |  | 105064285 | - | 249402 | 249552 | 248921 | 249089 | 250919 | 251172 | 0 | 2 | 6 | 0 | 271 | 135 | 0.034999903 | 0 | 1 | -1 |  |  |
|  |  | 105065154 | - | 1223651 | 1223728 | 1213937 | 1214115 | 1226395 | 1226614 | 16 | 56 | 32 | 41 | 140 | 135 | 0.035038493 | 0.216 | 0.429 | -0.213 |  |  |
|  |  | 105065666 | - | 5436960 | 5437104 | 5433980 | 5434808 | 5442520 | 5442595 | 98 | 8 | 75 | 0 | 203 | 68 | 0.035365843 | 0.804 | 1 | -0.196 |  |  |
|  |  | 105079554 | + | 4165258 | 4165451 | 4163911 | 4164232 | 4167138 | 4167301 | 261 | 20 | 241 | 3 | 314 | 135 | 0.035955881 | 0.849 | 0.972 | -0.123 |  |  |
|  |  | 105072376 | + | 8640905 | 8641028 | 8640302 | 8640495 | 8648577 | 8648704 | 47 | 81 | 70 | 56 | 217 | 120 | 0.036459208 | 0.243 | 0.409 | -0.166 |  |  |
|  |  | 105076684 | + | 31356914 | 31356987 | 31343295 | 31343394 | 31371373 | 31378857 | 15 | 2 | 2 | 4 | 89 | 92 | 0.036497564 | 0.886 | 0.341 | 0.545 |  |  |
|  |  | 105062456 | - | 180839 | 180926 | 178134 | 180735 | 182758 | 182812 | 1 | 6 | 3 | 0 | 80 | 47 | 0.036996965 | 0.089 | 1 | -0.911 |  |  |
|  |  | 105066287 | - | 19253571 | 19253696 | 19251290 | 19251422 | 19254887 | 19255044 | 20 | 0 | 11 | 4 | 226 | 125 | 0.037052567 | 1 | 0.603 | 0.397 |  |  |
|  |  | 105079964 | - | 12913033 | 12913138 | 12911191 | 12911361 | 12914424 | 12914605 | 2 | 3 | 16 | 1 | 196 | 135 | 0.037371398 | 0.315 | 0.917 | -0.602 |  |  |
|  |  | 105063553 | - | 2730254 | 2730359 | 2727805 | 2728687 | 2732027 | 2732107 | 24 | 0 | 19 | 5 | 134 | 73 | 0.038488663 | 1 | 0.674 | 0.326 |  |  |
|  |  | 105079649 | + | 13266444 | 13266575 | 13265504 | 13265729 | 13266951 | 13267099 | 50 | 9 | 29 | 0 | 248 | 135 | 0.038851212 | 0.752 | 1 | -0.248 |  |  |
|  |  | 105064347 | + | 584074 | 584120 | 551691 | 551898 | 594648 | 594792 | 39 | 160 | 2 | 41 | 78 | 135 | 0.03919695 | 0.297 | 0.078 | 0.219 |  |  |
|  |  | 105080658 | + | 7227529 | 7227824 | 7221814 | 7222014 | 7229327 | 7231686 | 32 | 5 | 38 | 0 | 416 | 135 | 0.039317916 | 0.675 | 1 | -0.325 |  |  |
|  |  | 105073053 | + | 3835154 | 3835282 | 3834103 | 3834153 | 3841280 | 3842533 | 11 | 0 | 6 | 4 | 150 | 43 | 0.039489967 | 1 | 0.301 | 0.699 |  |  |
|  |  | 105083640 | + | 3448437 | 3448682 | 3445795 | 3445954 | 3452582 | 3452742 | 41 | 0 | 14 | 3 | 366 | 135 | 0.039503935 | 1 | 0.633 | 0.367 |  |  |
|  |  | 105075892 | - | 398865 | 398923 | 398221 | 398575 | 399622 | 399676 | 48 | 57 | 26 | 11 | 51 | 47 | 0.04036907 | 0.437 | 0.685 | -0.248 |  |  |
|  |  | 105077215 | - | 21565592 | 21566159 | 21563773 | 21563896 | 21580163 | 21580336 | 62 | 13 | 113 | 7 | 669 | 116 | 0.040657382 | 0.453 | 0.737 | -0.284 |  |  |
|  |  | 105066943 | + | 623276 | 623400 | 622449 | 622636 | 624505 | 625384 | 2 | 2 | 17 | 0 | 234 | 135 | 0.04068881 | 0.366 | 1 | -0.634 |  |  |
|  |  | 105070333 | + | 13396015 | 13396056 | 13395336 | 13395658 | 13396152 | 13396273 | 8 | 0 | 3 | 4 | 47 | 114 | 0.040936912 | 1 | 0.645 | 0.355 |  |  |
|  |  | 105074339 | - | 967883 | 968025 | 967542 | 967731 | 968260 | 968513 | 35 | 5 | 50 | 0 | 270 | 135 | 0.041116779 | 0.778 | 1 | -0.222 |  |  |
|  |  | 105067485 | - | 23567007 | 23567120 | 23545521 | 23545577 | 23574149 | 23574376 | 4 | 3 | 12 | 0 | 126 | 49 | 0.04127119 | 0.341 | 1 | -0.659 |  |  |
|  |  | 105069469 | - | 12231282 | 12231492 | 12228732 | 12228921 | 12244139 | 12244589 | 15 | 0 | 9 | 4 | 331 | 135 | 0.041556535 | 1 | 0.479 | 0.521 |  |  |
|  |  | 105064851 | + | 4866265 | 4866415 | 4846417 | 4846656 | 4869472 | 4869643 | 8 | 0 | 4 | 4 | 271 | 135 | 0.041875734 | 1 | 0.333 | 0.667 |  |  |
|  |  | 105080718 | + | 1660341 | 1660437 | 1658594 | 1658718 | 1661044 | 1661337 | 18 | 51 | 54 | 62 | 160 | 117 | 0.041888579 | 0.205 | 0.389 | -0.184 |  |  |
|  |  | 105075793 | + | 2922553 | 2922706 | 2910500 | 2910652 | 2933357 | 2933455 | 2 | 10 | 9 | 5 | 230 | 91 | 0.042063092 | 0.073 | 0.416 | -0.343 |  |  |
|  |  | 105066913 | - | 622145 | 622170 | 620342 | 620474 | 626654 | 626913 | 9 | 136 | 1 | 143 | 26 | 125 | 0.042155702 | 0.241 | 0.033 | 0.208 |  |  |
|  |  | 105067766 | - | 11336 | 11453 | 10547 | 10711 | 11848 | 11971 | 114 | 10 | 292 | 2 | 201 | 116 | 0.043088429 | 0.868 | 0.988 | -0.12 |  |  |
|  |  | 105078560 | - | 185093 | 185253 | 181978 | 182050 | 189965 | 190193 | 3 | 2 | 20 | 0 | 211 | 65 | 0.04335495 | 0.316 | 1 | -0.684 |  |  |
|  |  | 105072812 | - | 6042456 | 6042524 | 6019147 | 6019254 | 6043069 | 6043157 | 15 | 37 | 18 | 14 | 33 | 46 | 0.044454225 | 0.361 | 0.642 | -0.281 |  |  |
|  |  | 105078304 | + | 5536180 | 5536234 | 5529214 | 5529292 | 5538224 | 5539027 | 36 | 41 | 17 | 49 | 47 | 71 | 0.044477328 | 0.57 | 0.344 | 0.226 |  |  |
|  |  | 105079446 | - | 728454 | 728571 | 727089 | 727262 | 741122 | 741322 | 4 | 5 | 6 | 0 | 220 | 135 | 0.044794864 | 0.329 | 1 | -0.671 |  |  |
|  |  | 105075892 | - | 398865 | 398934 | 398221 | 398575 | 399622 | 399683 | 49 | 57 | 26 | 11 | 62 | 54 | 0.045415616 | 0.428 | 0.673 | -0.245 |  |  |
|  |  | 105080144 | - | 852227 | 852320 | 851395 | 851518 | 855182 | 855287 | 8 | 11 | 3 | 27 | 116 | 79 | 0.046078975 | 0.331 | 0.07 | 0.261 |  |  |
|  |  | 105071417 | - | 5806438 | 5807002 | 5801552 | 5801606 | 5810337 | 5810504 | 4 | 0 | 0 | 3 | 597 | 47 | 0.046182179 | 1 | 0 | 1 |  |  |
|  |  | 105064499 | + | 18511 | 18667 | 16178 | 16292 | 20365 | 20505 | 250 | 2 | 169 | 12 | 247 | 105 | 0.046485466 | 0.982 | 0.857 | 0.125 |  |  |
|  |  | 105070935 | + | 6054228 | 6054315 | 6052195 | 6052345 | 6075347 | 6075554 | 16 | 6 | 15 | 0 | 160 | 135 | 0.046561375 | 0.692 | 1 | -0.308 |  |  |
|  |  | 105074971 | + | 10050800 | 10050869 | 10049193 | 10049341 | 10053046 | 10053121 | 2 | 5 | 7 | 1 | 62 | 68 | 0.047444245 | 0.305 | 0.885 | -0.58 |  |  |
|  |  | 105079571 | + | 4995702 | 4995806 | 4993708 | 4993897 | 4996985 | 4997190 | 7 | 0 | 2 | 3 | 194 | 135 | 0.048252392 | 1 | 0.317 | 0.683 |  |  |
|  |  | 105062138 | + | 782554 | 782656 | 782231 | 782442 | 782893 | 782994 | 138 | 13 | 138 | 1 | 149 | 94 | 0.048700817 | 0.87 | 0.989 | -0.119 |  |  |
|  |  | 105079285 | - | 200117 | 200228 | 199445 | 199667 | 203524 | 203653 | 7 | 24 | 22 | 21 | 195 | 122 | 0.048726668 | 0.154 | 0.396 | -0.242 |  |  |
|  |  | 105065615 | + | 4279599 | 4279693 | 4278984 | 4279120 | 4280389 | 4280488 | 0 | 9 | 7 | 11 | 125 | 86 | 0.049108179 | 0 | 0.305 | -0.305 |  |  |
|  |  | 105074924 | + | 3744161 | 3744323 | 3741189 | 3741246 | 3747971 | 3748073 | 49 | 0 | 24 | 3 | 158 | 10 | 0.049667183 | 1 | 0.336 | 0.664 |  |  |
| **AS category** | **Stress** | **GeneID** | **strand** | **riExonStart_0base** | **riExonEnd** | **upstreamES** | **upstreamEE** | **downstreamES** | **downstreamEE** | **IC_SAMPLE_1** | **SC_SAMPLE_1** | **IC_SAMPLE_2** | **SC_SAMPLE_2** | **IncFormLen** | **SkipFormLen** | **PValue** | **IncLevel1** | **IncLevel2** | **IncLevelDifference** |  |  |
| RI | SS | 105074813 | + | 6323932 | 6324812 | 6323932 | 6324062 | 6324703 | 6324812 | 60 | 0 | 67 | 11 | 717 | 90 | 0.002334167 | 1 | 0.433 | 0.567 |  |  |
|  |  | 105064043 | + | 7368837 | 7371128 | 7368837 | 7369957 | 7370165 | 7371128 | 25 | 11 | 5 | 14 | 329 | 135 | 0.009084012 | 0.483 | 0.128 | 0.355 |  |  |
|  |  | 105077504 | + | 467011 | 467890 | 467011 | 467164 | 467570 | 467890 | 101 | 1 | 68 | 9 | 527 | 135 | 0.009361246 | 0.963 | 0.659 | 0.304 |  |  |
|  |  | 105062199 | - | 1173965 | 1174857 | 1173965 | 1174242 | 1174506 | 1174857 | 29 | 11 | 35 | 2 | 385 | 135 | 0.023746699 | 0.48 | 0.86 | -0.38 |  |  |
|  |  | 105067427 | + | 20741690 | 20742238 | 20741690 | 20741842 | 20742179 | 20742238 | 49 | 35 | 17 | 2 | 375 | 52 | 0.030974138 | 0.163 | 0.541 | -0.378 |  |  |
|  |  | 105076442 | + | 1199971 | 1203985 | 1199971 | 1200138 | 1203499 | 1203985 | 795 | 95 | 473 | 107 | 3482 | 135 | 0.039937519 | 0.245 | 0.146 | 0.099 |  |  |
|  |  | 105079381 | - | 3106178 | 3106678 | 3106178 | 3106364 | 3106588 | 3106678 | 5 | 1 | 4 | 12 | 293 | 83 | 0.048028098 | 0.586 | 0.086 | 0.5 |  |  |
|  | WS | 105064043 | + | 7368837 | 7371128 | 7368837 | 7369957 | 7370165 | 7371128 | 37 | 21 | 5 | 14 | 329 | 135 | 0.017663545 | 0.42 | 0.128 | 0.292 |  |  |
|  |  | 105077840 | - | 11879024 | 11879710 | 11879024 | 11879247 | 11879349 | 11879710 | 67 | 17 | 34 | 26 | 190 | 135 | 0.018203015 | 0.737 | 0.482 | 0.255 |  |  |
|  |  | 105067375 | - | 14346191 | 14348012 | 14346191 | 14347364 | 14347725 | 14348012 | 75 | 1 | 45 | 7 | 482 | 135 | 0.022349639 | 0.955 | 0.643 | 0.312 |  |  |
| **AS category** | **Stress** | **GeneID** | **strand** | **1stExonStart_0base** | **1stExonEnd** | **2ndExonStart_0base** | **2ndExonEnd** | **upstreamES** | **upstreamEE** | **downstreamES** | **downstreamEE** | **IC_SAMPLE_1** | **SC_SAMPLE_1** | **IC_SAMPLE_2** | **SC_SAMPLE_2** | **IncFormLen** | **SkipFormLen** | **PValue** | **IncLevel1** | **IncLevel2** | **IncLevelDifference** |
| MXE | SS | 105080389 | - | 4783831 | 4784040 | 4786453 | 4786511 | 4780541 | 4780671 | 4787246 | 4787379 | 75463 | 136999 | 143773 | 191929 | 81 | 309 | 0 | 0.678 | 0.741 | -0.063 |
|  |  | 105080389 | - | 4783831 | 4784040 | 4786453 | 4786511 | 4775720 | 4775859 | 4787246 | 4787379 | 75463 | 136999 | 143773 | 191929 | 90 | 318 | 0 | 0.661 | 0.726 | -0.065 |
|  |  | 105080389 | - | 4782203 | 4782301 | 4783831 | 4784040 | 4780541 | 4780671 | 4785343 | 4785476 | 340100 | 43312 | 500968 | 101351 | 309 | 161 | 0 | 0.804 | 0.72 | 0.084 |
|  |  | 105080389 | - | 4779413 | 4779628 | 4782203 | 4782301 | 4778132 | 4778265 | 4783146 | 4783279 | 35081 | 308960 | 115089 | 506434 | 164 | 318 | 0 | 0.18 | 0.306 | -0.126 |
|  |  | 105080389 | - | 4779413 | 4779628 | 4782203 | 4782301 | 4773632 | 4773765 | 4783146 | 4783279 | 35080 | 139016 | 115088 | 190934 | 164 | 318 | 0 | 0.329 | 0.539 | -0.21 |
|  |  | 105080389 | - | 4773632 | 4773765 | 4778132 | 4778265 | 4772954 | 4773022 | 4779413 | 4779628 | 169947 | 55594 | 315504 | 150892 | 178 | 178 | 0 | 0.754 | 0.676 | 0.078 |
|  |  | 105080389 | - | 4780541 | 4780671 | 4783146 | 4783279 | 4779413 | 4779628 | 4783831 | 4784040 | 286986 | 212754 | 577935 | 335261 | 252 | 246 | 0 | 0.568 | 0.627 | -0.059 |
|  |  | 105080389 | - | 4780541 | 4780671 | 4782203 | 4782301 | 4779413 | 4779628 | 4783146 | 4783279 | 35089 | 212755 | 115086 | 335260 | 173 | 237 | 0 | 0.184 | 0.32 | -0.136 |
|  |  | 105077217 | + | 22042991 | 22043064 | 22048239 | 22048399 | 22038960 | 22039272 | 22049604 | 22049699 | 2896 | 4158 | 3182 | 7432 | 85 | 234 | 0 | 0.657 | 0.541 | 0.116 |
|  |  | 105080389 | - | 4772954 | 4773022 | 4775720 | 4775859 | 4771629 | 4771742 | 4776914 | 4777012 | 136946 | 21557 | 170521 | 131197 | 191 | 49 | 0 | 0.62 | 0.25 | 0.37 |
|  |  | 105080389 | - | 4772954 | 4773022 | 4773632 | 4773765 | 4771629 | 4771742 | 4774953 | 4775177 | 139688 | 21566 | 322668 | 131226 | 223 | 93 | 0 | 0.73 | 0.506 | 0.224 |
|  |  | 105080389 | - | 4783146 | 4783279 | 4785343 | 4785476 | 4782203 | 4782301 | 4786453 | 4786511 | 11531 | 35080 | 24416 | 115086 | 124 | 124 | 0 | 0.247 | 0.175 | 0.072 |
|  |  | 105080389 | - | 4775720 | 4775859 | 4776914 | 4777012 | 4774953 | 4775177 | 4778132 | 4778265 | 57835 | 337477 | 122021 | 473037 | 173 | 255 | 0 | 0.202 | 0.275 | -0.073 |
|  |  | 105080389 | - | 4775720 | 4775859 | 4783146 | 4783279 | 4774953 | 4775177 | 4783831 | 4784040 | 286976 | 337477 | 577930 | 473036 | 252 | 264 | 0 | 0.471 | 0.561 | -0.09 |
|  |  | 105080389 | - | 4775720 | 4775859 | 4780541 | 4780671 | 4774953 | 4775177 | 4782203 | 4782301 | 43312 | 337477 | 101349 | 473036 | 202 | 220 | 0 | 0.123 | 0.189 | -0.066 |
|  |  | 105080389 | - | 4774953 | 4775177 | 4782203 | 4782301 | 4773632 | 4773765 | 4783146 | 4783279 | 35080 | 189234 | 115088 | 453446 | 164 | 327 | 0 | 0.27 | 0.336 | -0.066 |
|  |  | 105080389 | - | 4774953 | 4775177 | 4779413 | 4779628 | 4773632 | 4773765 | 4780541 | 4780671 | 351760 | 189234 | 526187 | 453445 | 315 | 324 | 0 | 0.657 | 0.544 | 0.113 |
|  |  | 105080389 | - | 4774953 | 4775177 | 4779413 | 4779628 | 4772954 | 4773022 | 4780541 | 4780671 | 351757 | 49568 | 526186 | 130898 | 250 | 259 | 0 | 0.88 | 0.806 | 0.074 |
|  |  | 105080389 | - | 4774953 | 4775177 | 4775720 | 4775859 | 4771629 | 4771742 | 4776914 | 4777012 | 136946 | 49561 | 170521 | 130878 | 191 | 272 | 0 | 0.797 | 0.65 | 0.147 |
|  |  | 105080389 | - | 4774953 | 4775177 | 4779413 | 4779628 | 4771629 | 4771742 | 4780541 | 4780671 | 351757 | 49559 | 526185 | 130879 | 295 | 304 | 0 | 0.88 | 0.806 | 0.074 |
|  |  | 105064863 | + | 2699168 | 2699342 | 2699426 | 2699586 | 2698804 | 2698912 | 2699809 | 2699901 | 2411 | 6711 | 4589 | 6805 | 211 | 197 | 0 | 0.251 | 0.386 | -0.135 |
|  |  | 105079731 | + | 3987954 | 3988080 | 3988310 | 3988596 | 3987474 | 3987588 | 3989211 | 3989876 | 35627 | 106601 | 51079 | 226529 | 210 | 379 | 0 | 0.376 | 0.289 | 0.087 |
|  |  | 105076516 | - | 10858487 | 10858640 | 10859097 | 10859289 | 10855357 | 10855496 | 10862215 | 10862341 | 17748 | 40366 | 20762 | 28431 | 294 | 255 | 0 | 0.276 | 0.388 | -0.112 |
|  |  | 105062488 | + | 2544487 | 2544612 | 2547075 | 2547211 | 2543533 | 2543623 | 2550259 | 2550513 | 22873 | 30229 | 22788 | 43480 | 184 | 206 | 0 | 0.459 | 0.37 | 0.089 |
|  |  | 105077715 | + | 4784765 | 4784903 | 4787284 | 4787401 | 4782979 | 4783086 | 4789313 | 4790385 | 3171 | 11863 | 6802 | 13496 | 227 | 185 | 0 | 0.179 | 0.291 | -0.112 |
|  |  | 105074273 | - | 2996491 | 2996524 | 2998371 | 2998446 | 2995009 | 2995179 | 3000097 | 3000277 | 402 | 781 | 1696 | 965 | 136 | 52 | 0 | 0.164 | 0.402 | -0.238 |
|  |  | 105068898 | - | 229980 | 230196 | 230527 | 230897 | 229162 | 229300 | 232922 | 233062 | 68335 | 24569 | 48119 | 25822 | 485 | 331 | 0 | 0.655 | 0.56 | 0.095 |
|  |  | 105077372 | - | 43613116 | 43613257 | 43613826 | 43613901 | 43611533 | 43611640 | 43615813 | 43615934 | 3342 | 4537 | 2669 | 5733 | 80 | 212 | 2.48E-14 | 0.661 | 0.552 | 0.109 |
|  |  | 105073481 | - | 96780 | 96909 | 98079 | 98194 | 93259 | 93415 | 100716 | 100870 | 514 | 489 | 1787 | 851 | 216 | 244 | 4.54E-10 | 0.543 | 0.703 | -0.16 |
|  |  | 105063978 | - | 4074123 | 4074218 | 4105664 | 4105780 | 4063843 | 4063948 | 4123136 | 4123280 | 3159 | 460 | 1719 | 516 | 181 | 139 | 2.47E-09 | 0.841 | 0.719 | 0.122 |
|  |  | 105079731 | + | 3986700 | 3986928 | 3988310 | 3988596 | 3985612 | 3985796 | 3989211 | 3989876 | 120798 | 106596 | 203801 | 226521 | 349 | 407 | 1.62E-08 | 0.569 | 0.512 | 0.057 |
|  |  | 105083529 | - | 10960062 | 10960214 | 10981503 | 10981741 | 10956713 | 10956832 | 10983900 | 10984162 | 642 | 1353 | 834 | 957 | 336 | 250 | 1.70E-08 | 0.261 | 0.393 | -0.132 |
|  |  | 105075625 | - | 1977394 | 1978408 | 1979488 | 1979632 | 1976836 | 1976977 | 1980167 | 1980257 | 1991 | 21545 | 1040 | 17968 | 217 | 1082 | 4.07E-08 | 0.315 | 0.224 | 0.091 |
|  |  | 105078135 | - | 769785 | 769896 | 770469 | 770533 | 769025 | 769373 | 770661 | 770807 | 323 | 3463 | 578 | 3202 | 114 | 208 | 2.75E-06 | 0.145 | 0.248 | -0.103 |
|  |  | 105064733 | + | 241532 | 241632 | 245629 | 245702 | 227891 | 228039 | 247746 | 247893 | 2022 | 1764 | 2474 | 1421 | 186 | 132 | 2.84E-06 | 0.449 | 0.553 | -0.104 |
|  |  | 105077217 | + | 22042991 | 22043064 | 22045740 | 22045915 | 22038960 | 22039272 | 22048239 | 22048399 | 3048 | 5576 | 3446 | 8868 | 132 | 296 | 2.98E-06 | 0.551 | 0.466 | 0.085 |
|  |  | 105080389 | - | 4785343 | 4785476 | 4786453 | 4786511 | 4783831 | 4784040 | 4787246 | 4787379 | 75464 | 203101 | 143781 | 309044 | 93 | 243 | 4.92E-06 | 0.493 | 0.549 | -0.056 |
|  |  | 105070110 | - | 2057650 | 2057848 | 2058401 | 2058512 | 2056837 | 2056939 | 2060417 | 2060799 | 1954 | 2931 | 2605 | 2658 | 168 | 279 | 6.73E-06 | 0.525 | 0.619 | -0.094 |
|  |  | 105069414 | + | 220892 | 220994 | 230758 | 230873 | 218503 | 218597 | 232999 | 233528 | 215 | 434 | 348 | 347 | 142 | 168 | 7.05E-06 | 0.37 | 0.543 | -0.173 |
|  |  | 105083676 | + | 374977 | 375149 | 376065 | 376223 | 373690 | 373788 | 376795 | 376971 | 3616 | 2355 | 3466 | 3240 | 249 | 235 | 9.36E-06 | 0.592 | 0.502 | 0.09 |
|  |  | 105068052 | - | 3690111 | 3690263 | 3691549 | 3691759 | 3688155 | 3688318 | 3693210 | 3693308 | 42 | 82 | 49 | 20 | 287 | 229 | 1.76E-05 | 0.29 | 0.662 | -0.372 |
|  |  | 105075253 | + | 2614745 | 2615441 | 2616146 | 2616368 | 2611398 | 2611575 | 2620432 | 2620566 | 489 | 312 | 539 | 175 | 809 | 335 | 2.40E-05 | 0.394 | 0.561 | -0.167 |
|  |  | 105069256 | - | 1228135 | 1228177 | 1229728 | 1229888 | 1224752 | 1224929 | 1231567 | 1231722 | 672 | 202 | 464 | 274 | 281 | 70 | 2.93E-05 | 0.453 | 0.297 | 0.156 |
|  |  | 105067994 | + | 62499 | 62769 | 66554 | 66726 | 59039 | 59234 | 68169 | 68368 | 289 | 265 | 337 | 151 | 391 | 293 | 6.46E-05 | 0.45 | 0.626 | -0.176 |
|  |  | 105066281 | + | 18803864 | 18804026 | 18809146 | 18809341 | 18777927 | 18778054 | 18818604 | 18818710 | 234 | 235 | 236 | 467 | 232 | 265 | 9.84E-05 | 0.532 | 0.366 | 0.166 |
|  |  | 105075043 | + | 6346073 | 6346232 | 6346727 | 6346853 | 6343721 | 6343853 | 6349013 | 6349214 | 1936 | 924 | 1953 | 1401 | 270 | 228 | 0.000152221 | 0.639 | 0.541 | 0.098 |
|  |  | 105075253 | + | 2614604 | 2615441 | 2616146 | 2616368 | 2611398 | 2611575 | 2620432 | 2620566 | 615 | 312 | 634 | 175 | 950 | 335 | 0.000189402 | 0.41 | 0.561 | -0.151 |
|  |  | 105076699 | + | 31640764 | 31640895 | 31640975 | 31641138 | 31636633 | 31636741 | 31641272 | 31641621 | 1715 | 10532 | 4685 | 18673 | 214 | 250 | 0.000218015 | 0.16 | 0.227 | -0.067 |
|  |  | 105062811 | - | 1524403 | 1524475 | 1609724 | 1609820 | 1514391 | 1514489 | 1642956 | 1643100 | 52 | 30 | 109 | 13 | 134 | 86 | 0.000220214 | 0.527 | 0.843 | -0.316 |
|  |  | 105064733 | + | 227891 | 228039 | 241532 | 241632 | 226266 | 226353 | 245629 | 245702 | 2284 | 1342 | 3314 | 1344 | 146 | 62 | 0.00032642 | 0.42 | 0.512 | -0.092 |
|  |  | 105074070 | + | 436768 | 436828 | 436939 | 437083 | 436524 | 436671 | 437160 | 437268 | 44 | 253 | 58 | 123 | 72 | 236 | 0.00047173 | 0.363 | 0.607 | -0.244 |
|  |  | 105075032 | + | 5276900 | 5277045 | 5281363 | 5281513 | 5272646 | 5272804 | 5282684 | 5282906 | 111 | 301 | 139 | 178 | 270 | 271 | 0.000831414 | 0.27 | 0.439 | -0.169 |
|  |  | 105066657 | + | 12008236 | 12008440 | 12014259 | 12014326 | 11982867 | 11982978 | 12025790 | 12025851 | 21 | 0 | 11 | 9 | 213 | 29 | 0.000968048 | 1 | 0.143 | 0.857 |
|  |  | 105066790 | + | 2281603 | 2281782 | 2290878 | 2291045 | 2280522 | 2280631 | 2293000 | 2293271 | 120 | 58 | 45 | 61 | 267 | 255 | 0.001037811 | 0.664 | 0.413 | 0.251 |
|  |  | 105075043 | + | 6343721 | 6343853 | 6346727 | 6346853 | 6341056 | 6341141 | 6349013 | 6349214 | 587 | 913 | 558 | 1366 | 193 | 181 | 0.002091423 | 0.376 | 0.277 | 0.099 |
|  |  | 105070023 | + | 3875458 | 3875638 | 3876800 | 3876983 | 3872695 | 3872872 | 3878369 | 3878570 | 228 | 367 | 723 | 702 | 301 | 304 | 0.002172961 | 0.386 | 0.51 | -0.124 |
|  |  | 105076014 | - | 1109392 | 1109428 | 1110839 | 1110908 | 1107509 | 1107706 | 1113550 | 1113619 | 0 | 45 | 11 | 23 | 62 | 29 | 0.002997761 | 0 | 0.183 | -0.183 |
|  |  | 105073541 | - | 2958074 | 2958216 | 2967224 | 2967315 | 2956771 | 2956896 | 2990780 | 2990842 | 14 | 47 | 3 | 78 | 71 | 173 | 0.003013123 | 0.421 | 0.086 | 0.335 |
|  |  | 105083817 | + | 6294498 | 6294639 | 6297284 | 6297443 | 6293703 | 6293845 | 6299333 | 6299508 | 87 | 118 | 68 | 203 | 268 | 280 | 0.004498437 | 0.435 | 0.259 | 0.176 |
|  |  | 105077639 | - | 149536 | 149638 | 159178 | 159359 | 140712 | 140885 | 164662 | 165912 | 56 | 2 | 36 | 12 | 302 | 190 | 0.00639526 | 0.946 | 0.654 | 0.292 |
|  |  | 105077199 | + | 17796140 | 17796222 | 17796310 | 17796403 | 17792922 | 17792996 | 17796631 | 17796766 | 0 | 15 | 4 | 3 | 75 | 97 | 0.007133719 | 0 | 0.633 | -0.633 |
|  |  | 105062060 | + | 2911431 | 2911544 | 2921146 | 2921253 | 2910131 | 2910322 | 2921445 | 2921588 | 261 | 246 | 276 | 148 | 212 | 200 | 0.007579977 | 0.5 | 0.638 | -0.138 |
|  |  | 105080207 | - | 1295441 | 1295610 | 1308183 | 1308405 | 1291421 | 1292004 | 1316870 | 1317162 | 282 | 78 | 189 | 105 | 343 | 290 | 0.008287167 | 0.753 | 0.603 | 0.15 |
|  |  | 105066110 | - | 480056 | 480117 | 490223 | 490355 | 456797 | 457105 | 494841 | 494979 | 33 | 92 | 37 | 33 | 246 | 104 | 0.008922806 | 0.132 | 0.322 | -0.19 |
|  |  | 105062275 | + | 7836866 | 7837015 | 7862013 | 7862155 | 7832491 | 7832550 | 7864777 | 7864910 | 597 | 4260 | 1204 | 5105 | 178 | 178 | 0.00900658 | 0.123 | 0.191 | -0.068 |
|  |  | 105076796 | - | 25073510 | 25073714 | 25080623 | 25080823 | 25071312 | 25071474 | 25087810 | 25087924 | 135 | 251 | 144 | 144 | 293 | 297 | 0.00912982 | 0.353 | 0.503 | -0.15 |
|  |  | 105072831 | - | 6688342 | 6688458 | 6688952 | 6689103 | 6687625 | 6687904 | 6692459 | 6692672 | 43 | 49 | 25 | 81 | 272 | 218 | 0.009136751 | 0.413 | 0.198 | 0.215 |
|  |  | 105074273 | - | 2976223 | 2976337 | 2978330 | 2978468 | 2975567 | 2975766 | 2979302 | 2979472 | 6694 | 4143 | 5308 | 4324 | 262 | 214 | 0.009749243 | 0.569 | 0.501 | 0.068 |
|  |  | 105074240 | + | 2002584 | 2002674 | 2002833 | 2003002 | 2001554 | 2001728 | 2004685 | 2005182 | 263 | 351 | 290 | 229 | 166 | 290 | 0.009765862 | 0.567 | 0.689 | -0.122 |
|  |  | 105080207 | - | 1308183 | 1308405 | 1316870 | 1317162 | 1295441 | 1295610 | 1319430 | 1319505 | 252 | 217 | 145 | 221 | 346 | 276 | 0.009783981 | 0.481 | 0.344 | 0.137 |
|  |  | 105075937 | + | 534881 | 535034 | 556067 | 556267 | 501390 | 501629 | 557514 | 557725 | 41 | 136 | 14 | 12 | 274 | 321 | 0.010907227 | 0.261 | 0.577 | -0.316 |
|  |  | 105064522 | - | 1840210 | 1840475 | 1870241 | 1870383 | 1817521 | 1817640 | 1873139 | 1873184 | 265 | 251 | 497 | 274 | 150 | 266 | 0.010944981 | 0.652 | 0.763 | -0.111 |
|  |  | 105072021 | + | 4531578 | 4531746 | 4532140 | 4532317 | 4530569 | 4530674 | 4538334 | 4538425 | 127 | 37 | 71 | 50 | 201 | 210 | 0.01440899 | 0.782 | 0.597 | 0.185 |
|  |  | 105073352 | - | 688385 | 688602 | 693616 | 694015 | 682325 | 683065 | 696372 | 696559 | 118 | 136 | 157 | 95 | 520 | 338 | 0.01479344 | 0.361 | 0.518 | -0.157 |
|  |  | 105066375 | + | 2744426 | 2744505 | 2750993 | 2751133 | 2735268 | 2735497 | 2753669 | 2753874 | 68 | 118 | 85 | 71 | 144 | 266 | 0.018427266 | 0.516 | 0.689 | -0.173 |
|  |  | 105083500 | - | 5850979 | 5851081 | 5851247 | 5851321 | 5849857 | 5850034 | 5854156 | 5854255 | 404 | 538 | 413 | 825 | 91 | 147 | 0.021600043 | 0.548 | 0.447 | 0.101 |
|  |  | 105066368 | + | 2409897 | 2410076 | 2411933 | 2411996 | 2409542 | 2409631 | 2413397 | 2414189 | 936 | 66 | 667 | 88 | 247 | 59 | 0.024778858 | 0.772 | 0.644 | 0.128 |
|  |  | 105069110 | + | 2491047 | 2491140 | 2491350 | 2491470 | 2489070 | 2489175 | 2498519 | 2499622 | 85 | 103 | 35 | 90 | 135 | 189 | 0.025071483 | 0.536 | 0.353 | 0.183 |
|  |  | 105070023 | + | 3875458 | 3875638 | 3875726 | 3875912 | 3872695 | 3872872 | 3876800 | 3876983 | 229 | 270 | 722 | 550 | 301 | 307 | 0.026436165 | 0.464 | 0.572 | -0.108 |
|  |  | 105067855 | - | 4701767 | 4701858 | 4703707 | 4703792 | 4699209 | 4700738 | 4704432 | 4704595 | 151 | 104 | 101 | 128 | 156 | 168 | 0.02679808 | 0.61 | 0.459 | 0.151 |
|  |  | 105071052 | + | 16256152 | 16256255 | 16256893 | 16256993 | 16255117 | 16255203 | 16257088 | 16257246 | 42 | 328 | 80 | 273 | 136 | 130 | 0.02682661 | 0.109 | 0.219 | -0.11 |
|  |  | 105075027 | - | 4485061 | 4485214 | 4509933 | 4510032 | 4298344 | 4298452 | 4568770 | 4568877 | 127 | 429 | 74 | 137 | 115 | 205 | 0.027541815 | 0.345 | 0.491 | -0.146 |
|  |  | 105082732 | - | 857658 | 857787 | 858347 | 858474 | 857312 | 857448 | 858792 | 858876 | 62 | 53 | 37 | 71 | 176 | 180 | 0.028100704 | 0.545 | 0.348 | 0.197 |
|  |  | 105075664 | - | 101222 | 101412 | 105785 | 106062 | 100082 | 100208 | 106490 | 106589 | 325 | 334 | 350 | 229 | 339 | 252 | 0.029698476 | 0.42 | 0.532 | -0.112 |
|  |  | 105070082 | - | 942501 | 942705 | 947021 | 947156 | 940478 | 940580 | 948812 | 948975 | 159 | 178 | 320 | 215 | 216 | 285 | 0.032788982 | 0.541 | 0.663 | -0.122 |
|  |  | 105062273 | + | 6950204 | 6950312 | 6960490 | 6960677 | 6942585 | 6942789 | 6963127 | 6963252 | 64 | 139 | 73 | 81 | 185 | 291 | 0.033518156 | 0.42 | 0.586 | -0.166 |
|  |  | 105070480 | + | 308057 | 308237 | 308924 | 309099 | 305865 | 306000 | 309739 | 310221 | 10 | 7 | 0 | 5 | 294 | 289 | 0.033521005 | 0.584 | 0 | 0.584 |
|  |  | 105075043 | + | 6343721 | 6343853 | 6346073 | 6346232 | 6341056 | 6341141 | 6346727 | 6346853 | 596 | 1050 | 581 | 1480 | 177 | 207 | 0.034509447 | 0.399 | 0.315 | 0.084 |
|  |  | 105078375 | - | 2337040 | 2337124 | 2339474 | 2339552 | 2336410 | 2336752 | 2340951 | 2341075 | 1 | 23 | 8 | 17 | 124 | 136 | 0.034738573 | 0.046 | 0.34 | -0.294 |
|  |  | 105062362 | - | 861705 | 861789 | 865837 | 865939 | 861517 | 861625 | 867269 | 867395 | 267 | 233 | 311 | 425 | 140 | 104 | 0.039961478 | 0.46 | 0.352 | 0.108 |
|  |  | 105066580 | - | 2224567 | 2224810 | 2225586 | 2225736 | 2223173 | 2224473 | 2226266 | 2226392 | 23 | 65 | 5 | 52 | 255 | 348 | 0.04104836 | 0.326 | 0.116 | 0.21 |
|  |  | 105077285 | + | 30624278 | 30624434 | 30647235 | 30647337 | 30567107 | 30567297 | 30745045 | 30745161 | 247 | 78 | 394 | 210 | 251 | 164 | 0.04154433 | 0.674 | 0.551 | 0.123 |
|  |  | 105079875 | + | 4193512 | 4193641 | 4195972 | 4196071 | 4190929 | 4191100 | 4199921 | 4200147 | 130 | 17 | 171 | 54 | 244 | 184 | 0.042133115 | 0.852 | 0.705 | 0.147 |
|  |  | 105078304 | + | 5532649 | 5532748 | 5536180 | 5536234 | 5529214 | 5529292 | 5538224 | 5539027 | 6 | 23 | 19 | 17 | 120 | 47 | 0.044193667 | 0.093 | 0.304 | -0.211 |
|  |  | 105067591 | + | 1876267 | 1876359 | 1889055 | 1889164 | 1875789 | 1875860 | 1889249 | 1889354 | 7 | 12 | 3 | 31 | 62 | 96 | 0.045450667 | 0.475 | 0.13 | 0.345 |
|  |  | 105081846 | + | 12246363 | 12246467 | 12251038 | 12251618 | 12236808 | 12237057 | 12253572 | 12253832 | 71 | 440 | 75 | 839 | 194 | 701 | 0.04751561 | 0.368 | 0.244 | 0.124 |
|  |  | 105075113 | - | 4172377 | 4172527 | 4176329 | 4176455 | 4171263 | 4171386 | 4181864 | 4182008 | 232 | 163 | 105 | 126 | 219 | 252 | 0.047675049 | 0.621 | 0.49 | 0.131 |
|  | WS | 105064733 | + | 227891 | 228039 | 241532 | 241632 | 226266 | 226353 | 245629 | 245702 | 3095 | 524 | 3314 | 1344 | 146 | 62 | 0 | 0.715 | 0.512 | 0.203 |
|  |  | 105066443 | - | 1564648 | 1564735 | 1569470 | 1569534 | 1563452 | 1563603 | 1573713 | 1573840 | 1436 | 3690 | 1242 | 1175 | 99 | 145 | 0 | 0.363 | 0.608 | -0.245 |
|  |  | 105080389 | - | 4782203 | 4782301 | 4783831 | 4784040 | 4780541 | 4780671 | 4785343 | 4785476 | 438605 | 155681 | 500968 | 101351 | 309 | 161 | 0 | 0.595 | 0.72 | -0.125 |
|  |  | 105080389 | - | 4779413 | 4779628 | 4782203 | 4782301 | 4778132 | 4778265 | 4783146 | 4783279 | 202101 | 486476 | 115089 | 506434 | 164 | 318 | 0 | 0.446 | 0.306 | 0.14 |
|  |  | 105080389 | - | 4779413 | 4779628 | 4780541 | 4780671 | 4778132 | 4778265 | 4782203 | 4782301 | 155680 | 486476 | 101348 | 506428 | 193 | 283 | 0 | 0.319 | 0.227 | 0.092 |
|  |  | 105080389 | - | 4779413 | 4779628 | 4782203 | 4782301 | 4773632 | 4773765 | 4783146 | 4783279 | 202097 | 158739 | 115088 | 190934 | 164 | 318 | 0 | 0.712 | 0.539 | 0.173 |
|  |  | 105080389 | - | 4780541 | 4780671 | 4783146 | 4783279 | 4779413 | 4779628 | 4783831 | 4784040 | 311385 | 239251 | 577935 | 335261 | 252 | 246 | 0 | 0.56 | 0.627 | -0.067 |
|  |  | 105080389 | - | 4780541 | 4780671 | 4782203 | 4782301 | 4779413 | 4779628 | 4783146 | 4783279 | 202108 | 239248 | 115086 | 335260 | 173 | 237 | 0 | 0.536 | 0.32 | 0.216 |
|  |  | 105080389 | - | 4780541 | 4780671 | 4785343 | 4785476 | 4779413 | 4779628 | 4786453 | 4786511 | 53532 | 239247 | 24422 | 335261 | 168 | 162 | 0 | 0.177 | 0.066 | 0.111 |
|  |  | 105080389 | - | 4772954 | 4773022 | 4775720 | 4775859 | 4771629 | 4771742 | 4776914 | 4777012 | 136027 | 257568 | 170521 | 131197 | 191 | 49 | 0 | 0.119 | 0.25 | -0.131 |
|  |  | 105080389 | - | 4772954 | 4773022 | 4776914 | 4777012 | 4771629 | 4771742 | 4778132 | 4778265 | 169859 | 257568 | 122021 | 131197 | 144 | 84 | 0 | 0.278 | 0.352 | -0.074 |
|  |  | 105080389 | - | 4772954 | 4773022 | 4773632 | 4773765 | 4771629 | 4771742 | 4774953 | 4775177 | 273408 | 257590 | 322668 | 131226 | 223 | 93 | 0 | 0.307 | 0.506 | -0.199 |
|  |  | 105080389 | - | 4785343 | 4785476 | 4786453 | 4786511 | 4783831 | 4784040 | 4787246 | 4787379 | 95369 | 301959 | 143781 | 309044 | 93 | 243 | 0 | 0.452 | 0.549 | -0.097 |
|  |  | 105080389 | - | 4775720 | 4775859 | 4780541 | 4780671 | 4774953 | 4775177 | 4782203 | 4782301 | 155679 | 253378 | 101349 | 473036 | 202 | 220 | 0 | 0.401 | 0.189 | 0.212 |
|  |  | 105080389 | - | 4775720 | 4775859 | 4785343 | 4785476 | 4774953 | 4775177 | 4786453 | 4786511 | 53530 | 253376 | 24428 | 473036 | 168 | 180 | 0 | 0.185 | 0.052 | 0.133 |
|  |  | 105080389 | - | 4775720 | 4775859 | 4776914 | 4777012 | 4774953 | 4775177 | 4778132 | 4778265 | 169855 | 253377 | 122021 | 473037 | 173 | 255 | 0 | 0.497 | 0.275 | 0.222 |
|  |  | 105080389 | - | 4774953 | 4775177 | 4776914 | 4777012 | 4773632 | 4773765 | 4778132 | 4778265 | 169854 | 399582 | 122028 | 453452 | 164 | 327 | 0 | 0.459 | 0.349 | 0.11 |
|  |  | 105080389 | - | 4774953 | 4775177 | 4782203 | 4782301 | 4773632 | 4773765 | 4783146 | 4783279 | 202097 | 399580 | 115088 | 453446 | 164 | 327 | 0 | 0.502 | 0.336 | 0.166 |
|  |  | 105080389 | - | 4774953 | 4775177 | 4776914 | 4777012 | 4771629 | 4771742 | 4778132 | 4778265 | 169859 | 126305 | 122021 | 130886 | 144 | 307 | 0 | 0.741 | 0.665 | 0.076 |
|  |  | 105080389 | - | 4774953 | 4775177 | 4776914 | 4777012 | 4772954 | 4773022 | 4778132 | 4778265 | 169854 | 126320 | 122022 | 130905 | 99 | 262 | 0 | 0.781 | 0.712 | 0.069 |
|  |  | 105079903 | + | 5581653 | 5581927 | 5584621 | 5584772 | 5579928 | 5580582 | 5585808 | 5586167 | 9048 | 6643 | 6104 | 6769 | 395 | 272 | 0 | 0.484 | 0.383 | 0.101 |
|  |  | 105079903 | + | 5579928 | 5580582 | 5581653 | 5581927 | 5577928 | 5578030 | 5584621 | 5584772 | 7820 | 14091 | 19608 | 13405 | 735 | 355 | 0 | 0.211 | 0.414 | -0.203 |
|  |  | 105080295 | + | 3567737 | 3567859 | 3571777 | 3571820 | 3567303 | 3567350 | 3575271 | 3575432 | 262 | 256 | 453 | 75 | 135 | 36 | 0 | 0.214 | 0.617 | -0.403 |
|  |  | 105065368 | + | 10461645 | 10461859 | 10462213 | 10462360 | 10454884 | 10454997 | 10463203 | 10463401 | 1096 | 1128 | 1638 | 494 | 306 | 241 | 0 | 0.434 | 0.723 | -0.289 |
|  |  | 105065368 | + | 10461645 | 10461859 | 10462213 | 10462360 | 10454369 | 10454774 | 10463203 | 10463401 | 642 | 1129 | 852 | 494 | 335 | 270 | 0 | 0.314 | 0.582 | -0.268 |
|  |  | 105073728 | + | 6446760 | 6446894 | 6447349 | 6447469 | 6444332 | 6444498 | 6449653 | 6449771 | 1682 | 829 | 549 | 754 | 230 | 202 | 0 | 0.641 | 0.39 | 0.251 |
|  |  | 105073420 | - | 2042886 | 2042961 | 2043705 | 2043807 | 2041046 | 2041950 | 2044111 | 2044194 | 27892 | 49637 | 16039 | 76934 | 131 | 77 | 0 | 0.248 | 0.109 | 0.139 |
|  |  | 105075418 | - | 358507 | 358693 | 359619 | 359786 | 357711 | 357811 | 367441 | 367587 | 5099 | 5732 | 10102 | 4943 | 246 | 265 | 0 | 0.489 | 0.688 | -0.199 |
|  |  | 105079731 | + | 3987474 | 3987588 | 3987954 | 3988080 | 3986700 | 3986928 | 3988310 | 3988596 | 45606 | 63723 | 53057 | 145464 | 214 | 238 | 0 | 0.443 | 0.289 | 0.154 |
|  |  | 105079731 | + | 3986700 | 3986928 | 3987474 | 3987588 | 3985612 | 3985796 | 3987954 | 3988080 | 85334 | 37871 | 203802 | 51079 | 333 | 198 | 0 | 0.573 | 0.703 | -0.13 |
|  |  | 105081677 | - | 1236064 | 1236171 | 1238948 | 1239044 | 1235090 | 1235308 | 1241748 | 1241971 | 1021 | 1489 | 537 | 2678 | 178 | 200 | 0 | 0.435 | 0.184 | 0.251 |
|  |  | 105062637 | + | 4374361 | 4374616 | 4374738 | 4375014 | 4373966 | 4374053 | 4379230 | 4379500 | 4622 | 5681 | 1313 | 3193 | 321 | 342 | 0 | 0.464 | 0.305 | 0.159 |
|  |  | 105062275 | + | 7836866 | 7837015 | 7862013 | 7862155 | 7832491 | 7832550 | 7864777 | 7864910 | 2252 | 3656 | 1204 | 5105 | 178 | 178 | 0 | 0.381 | 0.191 | 0.19 |
|  |  | 105081292 | - | 2911 | 3027 | 6152 | 6286 | 568 | 1266 | 6820 | 6916 | 3082 | 2997 | 1540 | 2787 | 208 | 172 | 0 | 0.46 | 0.314 | 0.146 |
|  |  | 105077715 | + | 4784765 | 4784903 | 4787284 | 4787401 | 4782979 | 4783086 | 4789313 | 4790385 | 9005 | 11320 | 6802 | 13496 | 227 | 185 | 0 | 0.393 | 0.291 | 0.102 |
|  |  | 105072124 | - | 2201579 | 2201672 | 2203282 | 2203455 | 2199702 | 2199818 | 2205124 | 2205315 | 14493 | 3390 | 17579 | 1216 | 268 | 146 | 0 | 0.7 | 0.887 | -0.187 |
|  |  | 105074273 | - | 2990049 | 2990157 | 2991643 | 2991806 | 2989252 | 2989381 | 2994283 | 2994388 | 3910 | 2865 | 4201 | 1689 | 234 | 152 | 0 | 0.47 | 0.618 | -0.148 |
|  |  | 105081265 | + | 1123269 | 1123376 | 1124869 | 1125086 | 1120662 | 1120791 | 1125515 | 1125655 | 1773 | 4424 | 1355 | 10540 | 185 | 323 | 0 | 0.412 | 0.183 | 0.229 |
|  |  | 105063293 | - | 339928 | 340191 | 342683 | 342776 | 332861 | 332977 | 348090 | 348160 | 339 | 2960 | 605 | 1558 | 74 | 286 | 0 | 0.307 | 0.6 | -0.293 |
|  |  | 105063293 | - | 339928 | 340191 | 342683 | 342776 | 335596 | 335721 | 348090 | 348160 | 338 | 5005 | 612 | 3063 | 83 | 295 | 0 | 0.194 | 0.415 | -0.221 |
|  |  | 105081748 | + | 4455832 | 4455995 | 4463495 | 4463645 | 4453569 | 4453777 | 4464270 | 4464431 | 8720 | 7185 | 11739 | 6503 | 284 | 271 | 0 | 0.537 | 0.633 | -0.096 |
|  |  | 105075418 | - | 357711 | 357811 | 358507 | 358693 | 353641 | 353727 | 359619 | 359786 | 8042 | 1686 | 12836 | 1471 | 251 | 130 | 1.11E-16 | 0.712 | 0.819 | -0.107 |
|  |  | 105074948 | + | 7499688 | 7499798 | 7516354 | 7516403 | 7476029 | 7476161 | 7525237 | 7525380 | 511 | 171 | 319 | 341 | 196 | 74 | 2.22E-16 | 0.53 | 0.261 | 0.269 |
|  |  | 105066131 | - | 2153486 | 2153611 | 2155153 | 2155229 | 2151929 | 2152038 | 2167386 | 2167552 | 1051 | 1818 | 1308 | 4176 | 105 | 203 | 3.33E-16 | 0.528 | 0.377 | 0.151 |
|  |  | 105074038 | - | 164174 | 164249 | 164385 | 164476 | 162685 | 162788 | 164605 | 164792 | 2147 | 909 | 2113 | 1650 | 129 | 97 | 1.11E-15 | 0.64 | 0.491 | 0.149 |
|  |  | 105078823 | - | 6549384 | 6549609 | 6549713 | 6549866 | 6548976 | 6549142 | 6549962 | 6550085 | 2858 | 4947 | 2212 | 6231 | 255 | 327 | 1.44E-15 | 0.426 | 0.313 | 0.113 |
|  |  | 105061985 | - | 2054404 | 2054573 | 2055608 | 2055705 | 2047466 | 2048889 | 2056577 | 2056660 | 130 | 293 | 35 | 451 | 121 | 231 | 1.55E-15 | 0.459 | 0.129 | 0.33 |
|  |  | 105065339 | - | 5701979 | 5702123 | 5782231 | 5782411 | 5684543 | 5684684 | 5811610 | 5811753 | 653 | 430 | 455 | 715 | 300 | 269 | 5.55E-15 | 0.577 | 0.363 | 0.214 |
|  |  | 105080389 | - | 4773632 | 4773765 | 4778132 | 4778265 | 4772954 | 4773022 | 4779413 | 4779628 | 327737 | 119054 | 315504 | 150892 | 178 | 178 | 1.22E-13 | 0.734 | 0.676 | 0.058 |
|  |  | 105077822 | + | 7868756 | 7868907 | 7869795 | 7869919 | 7868044 | 7868172 | 7871799 | 7872341 | 541 | 309 | 581 | 118 | 258 | 220 | 3.29E-11 | 0.599 | 0.808 | -0.209 |
|  |  | 105079229 | + | 8644561 | 8645016 | 8648773 | 8648899 | 8639936 | 8640090 | 8649450 | 8649615 | 44436 | 7792 | 19011 | 4712 | 576 | 238 | 4.24E-09 | 0.702 | 0.625 | 0.077 |
|  |  | 105074863 | + | 7497540 | 7497655 | 7540974 | 7541198 | 7496541 | 7496683 | 7544734 | 7544878 | 104 | 524 | 64 | 1298 | 216 | 345 | 6.45E-09 | 0.241 | 0.073 | 0.168 |
|  |  | 105075043 | + | 6343721 | 6343853 | 6346727 | 6346853 | 6341056 | 6341141 | 6349013 | 6349214 | 741 | 973 | 558 | 1366 | 193 | 181 | 9.20E-09 | 0.417 | 0.277 | 0.14 |
|  |  | 105073019 | - | 844678 | 844839 | 848175 | 848424 | 842407 | 842546 | 890422 | 890504 | 95 | 146 | 237 | 114 | 307 | 219 | 2.31E-08 | 0.317 | 0.597 | -0.28 |
|  |  | 105063293 | - | 324717 | 324921 | 325617 | 325719 | 324381 | 324630 | 328800 | 328905 | 617 | 3834 | 307 | 4004 | 153 | 288 | 3.20E-08 | 0.232 | 0.126 | 0.106 |
|  |  | 105083529 | - | 10960062 | 10960214 | 10981503 | 10981741 | 10956713 | 10956832 | 10983900 | 10984162 | 1116 | 729 | 834 | 957 | 336 | 250 | 6.46E-08 | 0.532 | 0.393 | 0.139 |
|  |  | 105067890 | - | 6617811 | 6618043 | 6622567 | 6622669 | 6613933 | 6614194 | 6625107 | 6625125 | 413 | 645 | 603 | 468 | 95 | 229 | 1.09E-07 | 0.607 | 0.756 | -0.149 |
|  |  | 105064522 | - | 1840210 | 1840475 | 1870241 | 1870383 | 1817521 | 1817640 | 1873139 | 1873184 | 361 | 429 | 497 | 274 | 150 | 266 | 2.03E-07 | 0.599 | 0.763 | -0.164 |
|  |  | 105063293 | - | 332861 | 332977 | 342683 | 342776 | 331796 | 331959 | 348090 | 348160 | 337 | 3247 | 606 | 2875 | 100 | 146 | 2.35E-07 | 0.132 | 0.235 | -0.103 |
|  |  | 105067766 | - | 10547 | 10711 | 11336 | 11453 | 9799 | 9919 | 11848 | 11971 | 115 | 291 | 292 | 302 | 179 | 244 | 2.90E-07 | 0.35 | 0.569 | -0.219 |
|  |  | 105076223 | + | 4397651 | 4397792 | 4398374 | 4398513 | 4396637 | 4396815 | 4398761 | 4398874 | 2460 | 1110 | 1803 | 459 | 239 | 235 | 5.23E-07 | 0.685 | 0.794 | -0.109 |
|  |  | 105066368 | + | 2409897 | 2410076 | 2411933 | 2411996 | 2409542 | 2409631 | 2413397 | 2414189 | 510 | 165 | 667 | 88 | 247 | 59 | 9.56E-07 | 0.425 | 0.644 | -0.219 |
|  |  | 105083801 | + | 3467978 | 3468133 | 3468257 | 3468426 | 3467740 | 3467844 | 3469254 | 3469378 | 118 | 136 | 140 | 52 | 220 | 234 | 4.50E-06 | 0.48 | 0.741 | -0.261 |
|  |  | 105080160 | - | 421645 | 421762 | 422075 | 422201 | 421258 | 421427 | 423163 | 423277 | 1457 | 1595 | 1658 | 1173 | 210 | 192 | 6.88E-06 | 0.455 | 0.564 | -0.109 |
|  |  | 105073604 | + | 5681814 | 5681917 | 5690989 | 5691182 | 5622533 | 5622639 | 5693045 | 5693141 | 24 | 15 | 3 | 31 | 110 | 232 | 1.22E-05 | 0.771 | 0.17 | 0.601 |
|  |  | 105062295 | + | 10594410 | 10594513 | 10596851 | 10596943 | 10593550 | 10593643 | 10597021 | 10597537 | 64 | 175 | 18 | 239 | 143 | 121 | 1.43E-05 | 0.236 | 0.06 | 0.176 |
|  |  | 105070849 | + | 408014 | 408122 | 408837 | 408933 | 405976 | 406060 | 410057 | 410206 | 481 | 550 | 636 | 406 | 144 | 120 | 1.65E-05 | 0.422 | 0.566 | -0.144 |
|  |  | 105070454 | - | 1615486 | 1615675 | 1624477 | 1624615 | 1612362 | 1612525 | 1630330 | 1630431 | 626 | 696 | 299 | 588 | 221 | 269 | 3.69E-05 | 0.523 | 0.382 | 0.141 |
|  |  | 105076223 | + | 4399554 | 4399788 | 4400012 | 4400119 | 4398761 | 4398874 | 4401106 | 4401768 | 3506 | 1557 | 1578 | 1053 | 326 | 171 | 3.72E-05 | 0.542 | 0.44 | 0.102 |
|  |  | 105074948 | + | 7516354 | 7516403 | 7525237 | 7525380 | 7499688 | 7499798 | 7527471 | 7528199 | 67 | 1056 | 33 | 1883 | 52 | 238 | 4.31E-05 | 0.225 | 0.074 | 0.151 |
|  |  | 105083801 | + | 3467978 | 3468133 | 3469254 | 3469378 | 3467740 | 3467844 | 3469662 | 3469772 | 118 | 89 | 140 | 34 | 206 | 164 | 7.23E-05 | 0.514 | 0.766 | -0.252 |
|  |  | 105071052 | + | 16256152 | 16256255 | 16256893 | 16256993 | 16255117 | 16255203 | 16257088 | 16257246 | 134 | 185 | 80 | 273 | 136 | 130 | 7.26E-05 | 0.409 | 0.219 | 0.19 |
|  |  | 105063890 | + | 328588 | 328722 | 330480 | 330567 | 327752 | 327877 | 335292 | 335484 | 391 | 292 | 393 | 148 | 237 | 143 | 7.91E-05 | 0.447 | 0.616 | -0.169 |
|  |  | 105076527 | + | 11895678 | 11895874 | 11898061 | 11898215 | 11894322 | 11894392 | 11898935 | 11899030 | 32 | 13 | 15 | 40 | 198 | 156 | 0.000109059 | 0.66 | 0.228 | 0.432 |
|  |  | 105083322 | + | 2944891 | 2945029 | 2946342 | 2946505 | 2943637 | 2943768 | 2949011 | 2949245 | 1305 | 1008 | 1056 | 1252 | 251 | 273 | 0.000119542 | 0.585 | 0.478 | 0.107 |
|  |  | 105064184 | + | 2028377 | 2028461 | 2034198 | 2034384 | 2025975 | 2026066 | 2036999 | 2037239 | 118 | 958 | 45 | 1015 | 103 | 256 | 0.000160465 | 0.234 | 0.099 | 0.135 |
|  |  | 105065554 | + | 933069 | 933174 | 933355 | 933481 | 929994 | 930132 | 933641 | 933842 | 2041 | 3996 | 2644 | 3678 | 192 | 234 | 0.000234026 | 0.384 | 0.467 | -0.083 |
|  |  | 105081846 | + | 12246363 | 12246467 | 12249248 | 12249392 | 12236808 | 12237057 | 12251038 | 12251618 | 210 | 295 | 71 | 221 | 194 | 270 | 0.000241283 | 0.498 | 0.309 | 0.189 |
|  |  | 105065282 | + | 850360 | 850564 | 851753 | 851963 | 847965 | 848151 | 852053 | 852203 | 471 | 494 | 1472 | 953 | 325 | 331 | 0.000279906 | 0.493 | 0.611 | -0.118 |
|  |  | 105062257 | - | 4052013 | 4052139 | 4055648 | 4055765 | 4050698 | 4050896 | 4064170 | 4064308 | 928 | 722 | 1008 | 483 | 216 | 234 | 0.00029812 | 0.582 | 0.693 | -0.111 |
|  |  | 105080207 | - | 1295441 | 1295610 | 1308183 | 1308405 | 1291421 | 1292004 | 1316870 | 1317162 | 103 | 133 | 189 | 105 | 343 | 290 | 0.000307646 | 0.396 | 0.603 | -0.207 |
|  |  | 105082825 | + | 6584168 | 6584334 | 6588912 | 6589039 | 6579382 | 6579438 | 6592132 | 6592333 | 1163 | 1525 | 1464 | 1284 | 201 | 154 | 0.000317239 | 0.369 | 0.466 | -0.097 |
|  |  | 105074273 | - | 2996491 | 2996524 | 2998371 | 2998446 | 2995009 | 2995179 | 3000097 | 3000277 | 2594 | 990 | 1696 | 965 | 136 | 52 | 0.000331603 | 0.5 | 0.402 | 0.098 |
|  |  | 105076948 | + | 8480093 | 8480153 | 8480422 | 8480502 | 8479793 | 8479919 | 8482847 | 8482929 | 32 | 11 | 13 | 30 | 37 | 70 | 0.000337554 | 0.846 | 0.45 | 0.396 |
|  |  | 105080916 | - | 2962343 | 2962465 | 2967636 | 2967719 | 2957348 | 2957554 | 2974271 | 2974413 | 66 | 66 | 44 | 127 | 152 | 230 | 0.000460814 | 0.602 | 0.344 | 0.258 |
|  |  | 105067766 | - | 11062 | 11202 | 11336 | 11453 | 10547 | 10711 | 11848 | 11971 | 114 | 280 | 292 | 366 | 201 | 247 | 0.000508618 | 0.333 | 0.495 | -0.162 |
|  |  | 105075920 | - | 697924 | 698076 | 698245 | 698420 | 696821 | 697320 | 699281 | 699412 | 174 | 105 | 186 | 235 | 285 | 262 | 0.000602058 | 0.604 | 0.421 | 0.183 |
|  |  | 105071923 | + | 11849658 | 11850320 | 11883675 | 11884039 | 11849331 | 11849535 | 11885234 | 11885377 | 877 | 120 | 880 | 41 | 783 | 485 | 0.000674884 | 0.819 | 0.93 | -0.111 |
|  |  | 105077089 | - | 3369558 | 3369676 | 3395048 | 3395143 | 3352851 | 3353580 | 3420674 | 3420837 | 208 | 184 | 132 | 235 | 176 | 222 | 0.000747948 | 0.588 | 0.415 | 0.173 |
|  |  | 105062362 | - | 861705 | 861789 | 865837 | 865939 | 861517 | 861625 | 867269 | 867395 | 586 | 471 | 311 | 425 | 140 | 104 | 0.000768617 | 0.48 | 0.352 | 0.128 |
|  |  | 105065482 | - | 2439975 | 2440121 | 2440976 | 2441149 | 2437789 | 2437971 | 2447292 | 2447412 | 625 | 439 | 821 | 345 | 272 | 248 | 0.000786575 | 0.565 | 0.685 | -0.12 |
|  |  | 105077834 | + | 11626325 | 11626511 | 11629960 | 11630073 | 11618945 | 11619112 | 11633793 | 11633905 | 346 | 186 | 469 | 130 | 277 | 182 | 0.000830334 | 0.55 | 0.703 | -0.153 |
|  |  | 105073019 | - | 844678 | 844839 | 848175 | 848424 | 836488 | 836936 | 890422 | 890504 | 96 | 18 | 241 | 5 | 310 | 222 | 0.001068501 | 0.793 | 0.972 | -0.179 |
|  |  | 105080386 | - | 4726876 | 4727088 | 4728230 | 4728279 | 4726551 | 4726684 | 4730389 | 4730508 | 183 | 1296 | 97 | 1295 | 52 | 301 | 0.00115893 | 0.45 | 0.302 | 0.148 |
|  |  | 105070941 | - | 6303078 | 6303206 | 6305989 | 6306116 | 6299765 | 6299894 | 6307877 | 6308114 | 104 | 134 | 141 | 80 | 227 | 229 | 0.001170966 | 0.439 | 0.64 | -0.201 |
|  |  | 105065709 | + | 7761451 | 7761589 | 7762018 | 7762176 | 7760899 | 7760972 | 7762264 | 7763277 | 31 | 67 | 42 | 26 | 193 | 210 | 0.001386587 | 0.335 | 0.637 | -0.302 |
|  |  | 105071668 | + | 1566709 | 1566899 | 1569420 | 1569621 | 1561216 | 1561364 | 1571577 | 1571818 | 2099 | 1922 | 1953 | 2505 | 311 | 322 | 0.001742234 | 0.531 | 0.447 | 0.084 |
|  |  | 105069177 | - | 7501358 | 7501458 | 7501719 | 7501783 | 7500807 | 7500935 | 7501876 | 7501996 | 7 | 14 | 12 | 1 | 78 | 150 | 0.002044194 | 0.49 | 0.958 | -0.468 |
|  |  | 105076229 | + | 4938347 | 4938478 | 4941030 | 4941190 | 4931026 | 4931127 | 4946376 | 4947933 | 57 | 68 | 94 | 42 | 207 | 240 | 0.002703673 | 0.493 | 0.722 | -0.229 |
|  |  | 105063149 | - | 18560873 | 18561104 | 18564155 | 18564296 | 18558892 | 18558973 | 18564493 | 18564647 | 186 | 148 | 241 | 92 | 207 | 291 | 0.002926777 | 0.639 | 0.786 | -0.147 |
|  |  | 105062910 | + | 3430908 | 3430966 | 3433041 | 3433236 | 3426517 | 3426689 | 3438020 | 3438216 | 37 | 85 | 26 | 172 | 102 | 316 | 0.00301552 | 0.574 | 0.319 | 0.255 |
|  |  | 105073481 | - | 96780 | 96909 | 98079 | 98194 | 93259 | 93415 | 100716 | 100870 | 500 | 378 | 1787 | 851 | 216 | 244 | 0.003080149 | 0.599 | 0.703 | -0.104 |
|  |  | 105078466 | - | 9319022 | 9319133 | 9321307 | 9321376 | 9318320 | 9318392 | 9327607 | 9327696 | 146 | 143 | 85 | 211 | 9 | 85 | 0.00328124 | 0.906 | 0.792 | 0.114 |
|  |  | 105070522 | - | 1623926 | 1624043 | 1633188 | 1634421 | 1617888 | 1618002 | 1641177 | 1641282 | 301 | 26 | 387 | 9 | 1289 | 155 | 0.00429265 | 0.582 | 0.838 | -0.256 |
|  |  | 105066368 | + | 2404861 | 2404961 | 2405327 | 2405426 | 2396487 | 2396616 | 2407205 | 2407315 | 372 | 286 | 610 | 279 | 141 | 139 | 0.004317711 | 0.562 | 0.683 | -0.121 |
|  |  | 105063923 | + | 2357902 | 2358064 | 2359268 | 2359372 | 2356696 | 2356901 | 2359757 | 2359984 | 305 | 204 | 253 | 294 | 283 | 194 | 0.005094272 | 0.506 | 0.371 | 0.135 |
|  |  | 105077681 | - | 2715032 | 2715155 | 2716340 | 2716504 | 2714392 | 2714524 | 2719918 | 2720714 | 39 | 23 | 55 | 7 | 275 | 222 | 0.005260735 | 0.578 | 0.864 | -0.286 |
|  |  | 105064870 | + | 2836881 | 2836990 | 2839187 | 2839291 | 2835828 | 2835946 | 2839670 | 2839772 | 725 | 398 | 1116 | 928 | 140 | 130 | 0.005752569 | 0.628 | 0.528 | 0.1 |
|  |  | 105083161 | + | 238231 | 238345 | 240208 | 240301 | 236474 | 236767 | 243809 | 243891 | 35 | 22 | 51 | 7 | 154 | 112 | 0.005966085 | 0.536 | 0.841 | -0.305 |
|  |  | 105081091 | - | 5161312 | 5161389 | 5174310 | 5174388 | 5159065 | 5159203 | 5181016 | 5181123 | 184 | 267 | 101 | 73 | 103 | 101 | 0.006111952 | 0.403 | 0.576 | -0.173 |
|  |  | 105066281 | + | 18809146 | 18809341 | 18818604 | 18818710 | 18803864 | 18804026 | 18823708 | 18823815 | 432 | 127 | 688 | 106 | 281 | 163 | 0.007265255 | 0.664 | 0.79 | -0.126 |
|  |  | 105063093 | + | 9928047 | 9928139 | 9930052 | 9930192 | 9926204 | 9926312 | 9932217 | 9932409 | 33 | 91 | 9 | 89 | 136 | 232 | 0.007740722 | 0.382 | 0.147 | 0.235 |
|  |  | 105070941 | - | 6305989 | 6306116 | 6307877 | 6308114 | 6303078 | 6303206 | 6311068 | 6311198 | 208 | 98 | 302 | 71 | 332 | 214 | 0.008610828 | 0.578 | 0.733 | -0.155 |
|  |  | 105081597 | + | 10537985 | 10538128 | 10538325 | 10538442 | 10535674 | 10535949 | 10538825 | 10538931 | 210 | 90 | 155 | 128 | 234 | 184 | 0.009159279 | 0.647 | 0.488 | 0.159 |
|  |  | 105081846 | + | 12246363 | 12246467 | 12251038 | 12251618 | 12236808 | 12237057 | 12253572 | 12253832 | 209 | 1294 | 75 | 839 | 194 | 701 | 0.009609987 | 0.369 | 0.244 | 0.125 |
|  |  | 105080386 | - | 4727793 | 4727926 | 4728230 | 4728279 | 4726876 | 4727088 | 4730389 | 4730508 | 205 | 1049 | 116 | 1011 | 61 | 229 | 0.010208553 | 0.423 | 0.301 | 0.122 |
|  |  | 105075664 | - | 101222 | 101412 | 105785 | 106013 | 100082 | 100208 | 106490 | 106589 | 92 | 181 | 53 | 229 | 290 | 252 | 0.010457516 | 0.306 | 0.167 | 0.139 |
|  |  | 105079766 | + | 1642350 | 1642506 | 1643589 | 1643669 | 1641138 | 1641368 | 1644113 | 1644761 | 191 | 179 | 125 | 220 | 277 | 146 | 0.01097621 | 0.36 | 0.23 | 0.13 |
|  |  | 105076087 | - | 282184 | 282328 | 288433 | 288503 | 278366 | 278723 | 291828 | 291985 | 95 | 219 | 66 | 73 | 126 | 270 | 0.011633119 | 0.482 | 0.66 | -0.178 |
|  |  | 105064785 | - | 3071938 | 3072055 | 3075881 | 3076091 | 3069929 | 3070325 | 3094946 | 3095078 | 79 | 28 | 53 | 48 | 321 | 210 | 0.012750375 | 0.649 | 0.419 | 0.23 |
|  |  | 105064350 | + | 3010119 | 3010324 | 3018481 | 3018680 | 3007229 | 3007394 | 3024469 | 3024639 | 609 | 676 | 583 | 962 | 326 | 320 | 0.012825369 | 0.469 | 0.373 | 0.096 |
|  |  | 105079618 | - | 1803702 | 1803822 | 1808572 | 1808686 | 1801774 | 1802025 | 1817369 | 1817474 | 57 | 82 | 75 | 47 | 177 | 189 | 0.01287742 | 0.426 | 0.63 | -0.204 |
|  |  | 105067668 | - | 9629848 | 9629977 | 9630823 | 9630944 | 9629683 | 9629762 | 9631706 | 9631916 | 609 | 202 | 619 | 106 | 165 | 181 | 0.013576342 | 0.768 | 0.865 | -0.097 |
|  |  | 105082825 | + | 6584168 | 6584334 | 6588912 | 6589039 | 6572382 | 6572453 | 6592132 | 6592333 | 405 | 1523 | 556 | 1285 | 216 | 169 | 0.013869233 | 0.172 | 0.253 | -0.081 |
|  |  | 105073096 | - | 5191341 | 5191500 | 5193029 | 5195378 | 5188317 | 5188493 | 5197580 | 5197922 | 384 | 8 | 375 | 26 | 2470 | 280 | 0.0145992 | 0.845 | 0.62 | 0.225 |
|  |  | 105065818 | + | 6903021 | 6903140 | 6907696 | 6907735 | 6899650 | 6899681 | 6933709 | 6933868 | 70 | 29 | 20 | 26 | 113 | 32 | 0.014685498 | 0.406 | 0.179 | 0.227 |
|  |  | 105064347 | + | 595413 | 595535 | 597441 | 597593 | 594648 | 594792 | 597713 | 597841 | 110 | 144 | 60 | 156 | 216 | 259 | 0.015311877 | 0.478 | 0.316 | 0.162 |
|  |  | 105064733 | + | 226086 | 226144 | 245629 | 245702 | 224696 | 224896 | 247746 | 247893 | 2185 | 2186 | 1955 | 1420 | 102 | 132 | 0.015717523 | 0.564 | 0.641 | -0.077 |
|  |  | 105069531 | + | 2014672 | 2015002 | 2019085 | 2019424 | 2011265 | 2011413 | 2022580 | 2022685 | 48 | 15 | 43 | 40 | 414 | 423 | 0.016289995 | 0.766 | 0.523 | 0.243 |
|  |  | 105084146 | - | 1796229 | 1796250 | 1796573 | 1796738 | 1796113 | 1796140 | 1797223 | 1797409 | 719 | 28 | 428 | 4 | 171 | 14 | 0.01642906 | 0.678 | 0.898 | -0.22 |
|  |  | 105080294 | + | 3586241 | 3586281 | 3586680 | 3586841 | 3585589 | 3585692 | 3587116 | 3587716 | 0 | 90 | 5 | 57 | 33 | 243 | 0.01718215 | 0 | 0.392 | -0.392 |
|  |  | 105069542 | + | 2186089 | 2186193 | 2186416 | 2186550 | 2185723 | 2185978 | 2186717 | 2186843 | 60 | 98 | 36 | 132 | 178 | 238 | 0.017529431 | 0.45 | 0.267 | 0.183 |
|  |  | 105063978 | - | 4003856 | 4003943 | 4005730 | 4005817 | 4000440 | 4000515 | 4006082 | 4006169 | 39 | 114 | 55 | 70 | 38 | 38 | 0.018764876 | 0.255 | 0.44 | -0.185 |
|  |  | 105061686 | - | 2092247 | 2092440 | 2101314 | 2101442 | 2087748 | 2087931 | 2108098 | 2108272 | 58 | 89 | 63 | 43 | 242 | 314 | 0.01940561 | 0.458 | 0.655 | -0.197 |
|  |  | 105067864 | - | 72900 | 73020 | 73198 | 73276 | 71256 | 71397 | 74591 | 74770 | 51 | 44 | 26 | 57 | 141 | 225 | 0.019587208 | 0.649 | 0.421 | 0.228 |
|  |  | 105076509 | - | 10240797 | 10240935 | 10248174 | 10248300 | 10233378 | 10233444 | 10256383 | 10256614 | 0 | 11 | 5 | 6 | 162 | 186 | 0.022438581 | 0 | 0.489 | -0.489 |
|  |  | 105070015 | + | 1714071 | 1714775 | 1715138 | 1715236 | 1710794 | 1711287 | 1716922 | 1717218 | 44 | 4 | 27 | 12 | 825 | 182 | 0.022633018 | 0.708 | 0.332 | 0.376 |
|  |  | 105064350 | + | 3010119 | 3010324 | 3025440 | 3025594 | 3007229 | 3007394 | 3027283 | 3027449 | 611 | 753 | 582 | 1057 | 326 | 275 | 0.023287312 | 0.406 | 0.317 | 0.089 |
|  |  | 105077285 | + | 30745045 | 30745161 | 30780311 | 30780475 | 30647235 | 30647337 | 30786147 | 30786819 | 290 | 531 | 210 | 607 | 178 | 245 | 0.024707624 | 0.429 | 0.323 | 0.106 |
|  |  | 105079815 | + | 1930893 | 1931046 | 1931845 | 1932073 | 1927089 | 1927242 | 1943569 | 1943701 | 217 | 72 | 121 | 81 | 264 | 339 | 0.025201195 | 0.795 | 0.657 | 0.138 |
|  |  | 105071923 | + | 11849658 | 11850320 | 11881986 | 11882081 | 11849331 | 11849535 | 11883675 | 11884039 | 875 | 76 | 876 | 36 | 783 | 176 | 0.026409468 | 0.721 | 0.845 | -0.124 |
|  |  | 105067890 | - | 6621303 | 6621445 | 6622567 | 6622669 | 6617811 | 6618043 | 6625107 | 6625125 | 411 | 162 | 599 | 119 | 95 | 146 | 0.027684304 | 0.796 | 0.886 | -0.09 |
|  |  | 105073808 | - | 721764 | 721902 | 723682 | 723857 | 721547 | 721689 | 724153 | 724229 | 20 | 15 | 19 | 46 | 230 | 196 | 0.030168825 | 0.532 | 0.26 | 0.272 |
|  |  | 105073707 | - | 5188868 | 5188996 | 5190455 | 5190557 | 5187692 | 5187792 | 5190764 | 5190845 | 709 | 797 | 948 | 1505 | 87 | 139 | 0.030193127 | 0.587 | 0.502 | 0.085 |
|  |  | 105075892 | - | 394905 | 395096 | 395211 | 395292 | 394398 | 394659 | 395474 | 395633 | 168 | 272 | 63 | 181 | 148 | 312 | 0.032882474 | 0.566 | 0.423 | 0.143 |
|  |  | 105075027 | - | 4485061 | 4485214 | 4509933 | 4510032 | 4298344 | 4298452 | 4568770 | 4568877 | 84 | 81 | 74 | 137 | 115 | 205 | 0.035119998 | 0.649 | 0.491 | 0.158 |
|  |  | 105064422 | + | 11781583 | 11781699 | 11789611 | 11789715 | 11780089 | 11780277 | 11792755 | 11792825 | 3 | 1 | 0 | 6 | 146 | 122 | 0.035620469 | 0.715 | 0 | 0.715 |
|  |  | 105070082 | - | 945133 | 945190 | 947021 | 947156 | 942501 | 942705 | 948812 | 948975 | 194 | 47 | 319 | 37 | 256 | 100 | 0.037220708 | 0.617 | 0.771 | -0.154 |
|  |  | 105069873 | + | 1390425 | 1390590 | 1391645 | 1391757 | 1388693 | 1389056 | 1403350 | 1403547 | 35 | 20 | 13 | 23 | 286 | 210 | 0.03832711 | 0.562 | 0.293 | 0.269 |
|  |  | 105073604 | + | 5681814 | 5681917 | 5690989 | 5691182 | 5632714 | 5632778 | 5693045 | 5693141 | 4 | 15 | 0 | 25 | 68 | 190 | 0.038431443 | 0.427 | 0 | 0.427 |
|  |  | 105073541 | - | 2958074 | 2958216 | 2967224 | 2967315 | 2956771 | 2956896 | 2990780 | 2990842 | 23 | 137 | 3 | 78 | 71 | 173 | 0.039483593 | 0.29 | 0.086 | 0.204 |
|  |  | 105067766 | - | 14969 | 15156 | 15237 | 15490 | 12278 | 12419 | 15737 | 15810 | 406 | 442 | 237 | 397 | 304 | 238 | 0.043223396 | 0.418 | 0.319 | 0.099 |
|  |  | 105073735 | + | 8019900 | 8019985 | 8023939 | 8024076 | 8017679 | 8017877 | 8027621 | 8027894 | 47 | 50 | 26 | 63 | 156 | 260 | 0.04423229 | 0.61 | 0.408 | 0.202 |
|  |  | 105071668 | + | 1561216 | 1561364 | 1566709 | 1566899 | 1560191 | 1560336 | 1569420 | 1569621 | 2003 | 2280 | 2211 | 1886 | 270 | 311 | 0.046833307 | 0.503 | 0.575 | -0.072 |
|  |  | 105063769 | + | 563240 | 563312 | 566747 | 567060 | 546337 | 546551 | 571625 | 572176 | 46 | 211 | 58 | 139 | 130 | 434 | 0.047381237 | 0.421 | 0.582 | -0.161 |
|  |  | 105082710 | + | 165040 | 165186 | 172599 | 172697 | 159360 | 159506 | 174322 | 174435 | 23 | 30 | 39 | 19 | 241 | 153 | 0.04821006 | 0.327 | 0.566 | -0.239 |
| **AS category** | **Stress** | **GeneID** | **strand** | **longExonStart_0base** | **longExonEnd** | **shortES** | **shortEE** | **flankingES** | **flankingEE** | **IC_SAMPLE_1** | **SC_SAMPLE_1** | **IC_SAMPLE_2** | **SC_SAMPLE_2** | **IncFormLen** | **SkipFormLen** | **PValue** | **IncLevel1** | **IncLevel2** | **IncLevelDifference** |  |  |
| A5SS | SS | 105065844 | + | 1534538 | 1535301 | 1534538 | 1534687 | 1535920 | 1536378 | 151 | 72 | 66 | 227 | 735 | 135 | 1.07E-14 | 0.278 | 0.051 | 0.227 |  |  |
|  |  | 105066370 | - | 2377826 | 2378011 | 2377871 | 2378011 | 2377282 | 2377369 | 24 | 21 | 20 | 0 | 116 | 78 | 0.000139597 | 0.435 | 1 | -0.565 |  |  |
|  |  | 105069555 | - | 2274180 | 2274833 | 2274319 | 2274833 | 2273887 | 2274027 | 205 | 94 | 279 | 257 | 265 | 133 | 0.001075556 | 0.523 | 0.353 | 0.17 |  |  |
|  |  | 105065228 | + | 430503 | 430798 | 430503 | 430683 | 431279 | 431711 | 19 | 22 | 4 | 36 | 243 | 135 | 0.002822969 | 0.324 | 0.058 | 0.266 |  |  |
|  |  | 105068370 | - | 2180256 | 2180506 | 2180295 | 2180506 | 2179889 | 2179973 | 0 | 13 | 6 | 4 | 109 | 77 | 0.003654844 | 0 | 0.514 | -0.514 |  |  |
|  |  | 105065481 | - | 1753835 | 1754002 | 1753838 | 1754002 | 1747839 | 1747956 | 4 | 194 | 17 | 87 | 110 | 110 | 0.005471284 | 0.02 | 0.163 | -0.143 |  |  |
|  |  | 105073907 | + | 1717611 | 1717704 | 1717611 | 1717700 | 1718894 | 1719018 | 8 | 7 | 0 | 11 | 68 | 64 | 0.005858954 | 0.518 | 0 | 0.518 |  |  |
|  |  | 105065202 | + | 1145240 | 1145953 | 1145240 | 1145293 | 1149776 | 1149900 | 185 | 0 | 121 | 6 | 674 | 28 | 0.006673459 | 1 | 0.456 | 0.544 |  |  |
|  |  | 105066151 | + | 4383790 | 4384354 | 4383790 | 4384013 | 4388191 | 4388308 | 56 | 0 | 20 | 4 | 437 | 110 | 0.012306405 | 1 | 0.557 | 0.443 |  |  |
|  |  | 105077504 | + | 467011 | 467164 | 467011 | 467160 | 467570 | 467890 | 1 | 3 | 9 | 0 | 135 | 135 | 0.017200153 | 0.25 | 1 | -0.75 |  |  |
|  |  | 105070058 | - | 232540 | 232943 | 232668 | 232943 | 228878 | 228966 | 367 | 418 | 678 | 491 | 202 | 81 | 0.018461852 | 0.26 | 0.356 | -0.096 |  |  |
|  |  | 105065656 | - | 5328982 | 5329303 | 5329109 | 5329303 | 5328282 | 5328474 | 87 | 11 | 146 | 2 | 255 | 135 | 0.020613047 | 0.807 | 0.975 | -0.168 |  |  |
|  |  | 105082213 | + | 28678392 | 28678518 | 28678392 | 28678458 | 28678624 | 28678732 | 2 | 6 | 5 | 0 | 85 | 25 | 0.020812232 | 0.089 | 1 | -0.911 |  |  |
|  |  | 105079962 | + | 12866826 | 12866988 | 12866826 | 12866961 | 12871202 | 12871253 | 8 | 0 | 3 | 4 | 57 | 37 | 0.028609002 | 1 | 0.327 | 0.673 |  |  |
|  |  | 105065270 | - | 1050524 | 1054130 | 1050635 | 1054130 | 1046756 | 1046905 | 32 | 12 | 32 | 36 | 239 | 135 | 0.030583082 | 0.601 | 0.334 | 0.267 |  |  |
|  |  | 105063083 | + | 9058896 | 9059065 | 9058896 | 9059019 | 9059759 | 9059896 | 14 | 12 | 11 | 36 | 150 | 111 | 0.038809245 | 0.463 | 0.184 | 0.279 |  |  |
|  |  | 105062477 | + | 663504 | 663994 | 663504 | 663866 | 664232 | 664333 | 23 | 10 | 10 | 17 | 215 | 94 | 0.038851127 | 0.501 | 0.205 | 0.296 |  |  |
|  |  | 105062530 | - | 1962951 | 1963215 | 1962966 | 1963215 | 1962250 | 1962336 | 5 | 0 | 0 | 2 | 87 | 79 | 0.045596949 | 1 | 0 | 1 |  |  |
|  |  | 105071501 | + | 1347693 | 1347913 | 1347693 | 1347744 | 1348032 | 1348167 | 22 | 20 | 68 | 22 | 192 | 37 | 0.049720208 | 0.175 | 0.373 | -0.198 |  |  |
|  | WS | 105070058 | - | 232540 | 232943 | 232668 | 232943 | 228878 | 228966 | 466 | 631 | 678 | 491 | 202 | 81 | 6.70E-06 | 0.228 | 0.356 | -0.128 |  |  |
|  |  | 105062290 | - | 9563417 | 9563649 | 9563522 | 9563649 | 9522638 | 9522849 | 30 | 36 | 5 | 39 | 218 | 120 | 0.001647763 | 0.314 | 0.066 | 0.248 |  |  |
|  |  | 105066370 | - | 2377826 | 2378011 | 2377871 | 2378011 | 2377282 | 2377369 | 11 | 8 | 20 | 0 | 116 | 78 | 0.002132199 | 0.48 | 1 | -0.52 |  |  |
|  |  | 105076595 | - | 22991311 | 22991430 | 22991331 | 22991430 | 22986855 | 22987009 | 50 | 35 | 88 | 18 | 112 | 92 | 0.002903239 | 0.54 | 0.801 | -0.261 |  |  |
|  |  | 105069180 | - | 7642169 | 7642295 | 7642192 | 7642295 | 7638131 | 7638340 | 6 | 6 | 17 | 0 | 119 | 96 | 0.003217496 | 0.447 | 1 | -0.553 |  |  |
|  |  | 105073907 | + | 1717611 | 1717704 | 1717611 | 1717700 | 1718894 | 1719018 | 15 | 19 | 0 | 11 | 68 | 64 | 0.007798223 | 0.426 | 0 | 0.426 |  |  |
|  |  | 105080193 | - | 1087639 | 1087816 | 1087657 | 1087816 | 1083779 | 1083989 | 24 | 21 | 27 | 78 | 146 | 135 | 0.009919071 | 0.514 | 0.242 | 0.272 |  |  |
|  |  | 105064556 | - | 1017732 | 1018020 | 1017766 | 1018020 | 1015655 | 1017503 | 17 | 41 | 4 | 56 | 162 | 135 | 0.014505236 | 0.257 | 0.056 | 0.201 |  |  |
|  |  | 105064806 | - | 1546154 | 1546587 | 1546237 | 1546587 | 1545517 | 1545665 | 3 | 4 | 10 | 0 | 211 | 135 | 0.017551964 | 0.324 | 1 | -0.676 |  |  |
|  |  | 105079366 | + | 2555515 | 2555718 | 2555515 | 2555595 | 2560093 | 2560210 | 69 | 48 | 91 | 26 | 164 | 48 | 0.019186307 | 0.296 | 0.506 | -0.21 |  |  |
|  |  | 105064970 | - | 6736 | 8002 | 7949 | 8002 | 6325 | 6437 | 114 | 5 | 140 | 0 | 1215 | 16 | 0.026078382 | 0.231 | 1 | -0.769 |  |  |
|  |  | 105072620 | - | 35394 | 35909 | 35633 | 35909 | 34953 | 35027 | 5 | 2 | 0 | 6 | 292 | 67 | 0.028098557 | 0.365 | 0 | 0.365 |  |  |
|  |  | 105068953 | + | 7658820 | 7659247 | 7658820 | 7659109 | 7661128 | 7661273 | 0 | 15 | 9 | 18 | 266 | 135 | 0.028959244 | 0 | 0.202 | -0.202 |  |  |
|  |  | 105065270 | - | 1050524 | 1054130 | 1050635 | 1054130 | 1046756 | 1046905 | 22 | 7 | 32 | 36 | 239 | 135 | 0.029941303 | 0.64 | 0.334 | 0.306 |  |  |
|  |  | 105068811 | + | 1482938 | 1483345 | 1482938 | 1483294 | 1483729 | 1483850 | 32 | 6 | 28 | 20 | 158 | 114 | 0.031074604 | 0.794 | 0.503 | 0.291 |  |  |
|  |  | 105083038 | + | 4533160 | 4533412 | 4533160 | 4533349 | 4533951 | 4534422 | 3 | 3 | 11 | 0 | 191 | 135 | 0.035593335 | 0.414 | 1 | -0.586 |  |  |
|  |  | 105069180 | - | 7642163 | 7642295 | 7642192 | 7642295 | 7638131 | 7638340 | 29 | 6 | 32 | 0 | 125 | 96 | 0.042047276 | 0.788 | 1 | -0.212 |  |  |
|  |  | 105083854 | - | 7408990 | 7409202 | 7409010 | 7409202 | 7405122 | 7405547 | 102 | 31 | 99 | 63 | 148 | 135 | 0.047889567 | 0.75 | 0.589 | 0.161 |  |  |
| **AS category** | **Stress** | **GeneID** | **strand** | **longExonStart_0base** | **longExonEnd** | **shortES** | **shortEE** | **flankingES** | **flankingEE** | **IC_SAMPLE_1** | **SC_SAMPLE_1** | **IC_SAMPLE_2** | **SC_SAMPLE_2** | **IncFormLen** | **SkipFormLen** | **PValue** | **IncLevel1** | **IncLevel2** | **IncLevelDifference** |  |  |
| A3SS | SS | 105069338 | - | 710936 | 711387 | 710936 | 711291 | 711699 | 711876 | 349 | 9 | 222 | 44 | 224 | 135 | 1.69E-06 | 0.959 | 0.753 | 0.206 |  |  |
|  |  | 105074813 | + | 6324324 | 6324812 | 6324703 | 6324812 | 6323932 | 6324062 | 27 | 0 | 20 | 11 | 455 | 90 | 0.000703508 | 1 | 0.265 | 0.735 |  |  |
|  |  | 105073067 | + | 4224419 | 4226866 | 4226667 | 4226866 | 4222562 | 4222685 | 1314 | 0 | 1070 | 13 | 2350 | 116 | 0.00092338 | 1 | 0.802 | 0.198 |  |  |
|  |  | 105071215 | + | 121360 | 121635 | 121521 | 121635 | 121201 | 121275 | 63 | 0 | 111 | 15 | 186 | 39 | 0.003328817 | 1 | 0.608 | 0.392 |  |  |
|  |  | 105075326 | + | 2231267 | 2231455 | 2231270 | 2231455 | 2223360 | 2223759 | 2 | 6 | 8 | 0 | 135 | 135 | 0.00442541 | 0.25 | 1 | -0.75 |  |  |
|  |  | 105079822 | - | 3100460 | 3102504 | 3100460 | 3100615 | 3102661 | 3102744 | 2507 | 11 | 1446 | 24 | 1951 | 76 | 0.004811658 | 0.899 | 0.701 | 0.198 |  |  |
|  |  | 105074855 | - | 7114269 | 7114380 | 7114269 | 7114377 | 7114449 | 7114504 | 9 | 9 | 11 | 0 | 17 | 14 | 0.006615746 | 0.452 | 1 | -0.548 |  |  |
|  |  | 105070957 | - | 8414108 | 8414214 | 8414108 | 8414211 | 8420130 | 8420253 | 5 | 12 | 11 | 2 | 80 | 77 | 0.007087906 | 0.286 | 0.841 | -0.555 |  |  |
|  |  | 105076176 | - | 3048375 | 3048539 | 3048375 | 3048535 | 3050992 | 3051147 | 8 | 12 | 29 | 7 | 135 | 135 | 0.009535909 | 0.4 | 0.806 | -0.406 |  |  |
|  |  | 105064650 | + | 449531 | 450543 | 449788 | 450543 | 449170 | 449284 | 239 | 3 | 129 | 12 | 350 | 107 | 0.011951571 | 0.961 | 0.767 | 0.194 |  |  |
|  |  | 105065050 | + | 5952347 | 5952465 | 5952374 | 5952465 | 5952111 | 5952174 | 22 | 1 | 8 | 6 | 32 | 5 | 0.012691605 | 0.775 | 0.172 | 0.603 |  |  |
|  |  | 105062215 | - | 1460838 | 1461007 | 1460838 | 1460955 | 1462304 | 1462409 | 9 | 8 | 10 | 0 | 118 | 73 | 0.013629616 | 0.41 | 1 | -0.59 |  |  |
|  |  | 105082109 | - | 22524043 | 22524720 | 22524043 | 22524462 | 22527162 | 22527353 | 68 | 0 | 68 | 7 | 379 | 135 | 0.025956036 | 1 | 0.776 | 0.224 |  |  |
|  |  | 105073408 | + | 265387 | 265524 | 265390 | 265524 | 264900 | 265043 | 63 | 11 | 38 | 22 | 130 | 127 | 0.026512153 | 0.848 | 0.628 | 0.22 |  |  |
|  |  | 105066374 | + | 2656384 | 2656451 | 2656387 | 2656451 | 2655567 | 2655734 | 32 | 8 | 29 | 0 | 60 | 57 | 0.0267463 | 0.792 | 1 | -0.208 |  |  |
|  |  | 105081629 | + | 12495129 | 12495729 | 12495147 | 12495729 | 12493607 | 12493811 | 46 | 9 | 18 | 14 | 146 | 135 | 0.027289692 | 0.825 | 0.543 | 0.282 |  |  |
|  |  | 105064080 | + | 13783693 | 13783852 | 13783696 | 13783852 | 13779705 | 13779798 | 163 | 46 | 125 | 73 | 86 | 86 | 0.029220477 | 0.78 | 0.631 | 0.149 |  |  |
|  |  | 105080774 | + | 8762664 | 8762881 | 8762684 | 8762881 | 8760462 | 8760586 | 27 | 24 | 11 | 32 | 130 | 117 | 0.029378095 | 0.503 | 0.236 | 0.267 |  |  |
|  |  | 105069125 | - | 4265946 | 4266112 | 4265946 | 4266097 | 4266786 | 4266925 | 15 | 10 | 9 | 0 | 140 | 132 | 0.030642363 | 0.586 | 1 | -0.414 |  |  |
|  |  | 105069166 | + | 868228 | 868307 | 868243 | 868307 | 867525 | 867742 | 19 | 3 | 2 | 4 | 72 | 57 | 0.036079583 | 0.834 | 0.284 | 0.55 |  |  |
|  |  | 105072764 | - | 3064450 | 3064554 | 3064450 | 3064551 | 3065583 | 3065705 | 226 | 257 | 174 | 119 | 77 | 74 | 0.039843527 | 0.458 | 0.584 | -0.126 |  |  |
|  |  | 105082202 | - | 28604196 | 28604355 | 28604196 | 28604280 | 28604525 | 28604610 | 1 | 8 | 8 | 4 | 88 | 20 | 0.041154327 | 0.028 | 0.313 | -0.285 |  |  |
|  |  | 105067905 | - | 7490662 | 7491744 | 7490662 | 7491566 | 7497761 | 7497835 | 48 | 1 | 9 | 3 | 231 | 67 | 0.042578482 | 0.933 | 0.465 | 0.468 |  |  |
|  |  | 105076089 | + | 736379 | 736547 | 736439 | 736547 | 732516 | 732744 | 6 | 5 | 0 | 6 | 154 | 101 | 0.042749537 | 0.44 | 0 | 0.44 |  |  |
|  |  | 105073203 | - | 8070942 | 8071278 | 8070942 | 8071113 | 8073782 | 8073937 | 17 | 6 | 15 | 0 | 286 | 135 | 0.044461026 | 0.572 | 1 | -0.428 |  |  |
|  |  | 105064420 | - | 11532868 | 11533078 | 11532868 | 11532997 | 11534611 | 11534731 | 301 | 86 | 187 | 26 | 174 | 100 | 0.048166832 | 0.668 | 0.805 | -0.137 |  |  |
|  | WS | 105078784 | - | 2229222 | 2230480 | 2229222 | 2230420 | 2231194 | 2231364 | 524 | 245 | 234 | 270 | 188 | 135 | 2.16E-09 | 0.606 | 0.384 | 0.222 |  |  |
|  |  | 105069338 | - | 710936 | 711387 | 710936 | 711291 | 711699 | 711876 | 435 | 9 | 222 | 44 | 224 | 135 | 7.33E-08 | 0.967 | 0.753 | 0.214 |  |  |
|  |  | 105063983 | - | 4600364 | 4600527 | 4600364 | 4600524 | 4602123 | 4602213 | 65 | 0 | 25 | 13 | 83 | 83 | 1.14E-05 | 1 | 0.658 | 0.342 |  |  |
|  |  | 105071717 | - | 737735 | 739860 | 737735 | 739855 | 742848 | 743077 | 11 | 38 | 26 | 10 | 135 | 135 | 3.76E-05 | 0.224 | 0.722 | -0.498 |  |  |
|  |  | 105068022 | - | 49196 | 49451 | 49196 | 49448 | 49805 | 49826 | 11 | 5 | 2 | 15 | 14 | 14 | 0.002486282 | 0.688 | 0.118 | 0.57 |  |  |
|  |  | 105072462 | + | 2678930 | 2679085 | 2678963 | 2679085 | 2677897 | 2678005 | 66 | 11 | 73 | 0 | 107 | 81 | 0.006016633 | 0.82 | 1 | -0.18 |  |  |
|  |  | 105075326 | + | 2231267 | 2231455 | 2231270 | 2231455 | 2223360 | 2223759 | 0 | 3 | 8 | 0 | 135 | 135 | 0.007284579 | 0 | 1 | -1 |  |  |
|  |  | 105080229 | - | 2161891 | 2162079 | 2161891 | 2162002 | 2163971 | 2166886 | 4 | 0 | 0 | 5 | 174 | 104 | 0.008457469 | 1 | 0 | 1 |  |  |
|  |  | 105075775 | - | 1078365 | 1080731 | 1078365 | 1080728 | 1085801 | 1085897 | 0 | 3 | 6 | 0 | 89 | 89 | 0.012576633 | 0 | 1 | -1 |  |  |
|  |  | 105067247 | - | 200930 | 201097 | 200930 | 201069 | 201668 | 201795 | 4 | 0 | 0 | 4 | 138 | 117 | 0.014025657 | 1 | 0 | 1 |  |  |
|  |  | 105065050 | + | 5952347 | 5952465 | 5952374 | 5952465 | 5952111 | 5952174 | 12 | 0 | 8 | 6 | 32 | 5 | 0.017400767 | 1 | 0.172 | 0.828 |  |  |
|  |  | 105064970 | - | 6325 | 6446 | 6325 | 6437 | 7949 | 8002 | 0 | 5 | 3 | 0 | 25 | 16 | 0.018263339 | 0 | 1 | -1 |  |  |
|  |  | 105072549 | + | 929516 | 929650 | 929540 | 929650 | 929296 | 929341 | 11 | 0 | 17 | 10 | 30 | 6 | 0.023415287 | 1 | 0.254 | 0.746 |  |  |
|  |  | 105083161 | + | 232485 | 232701 | 232560 | 232701 | 231479 | 231586 | 32 | 9 | 66 | 3 | 167 | 99 | 0.029710246 | 0.678 | 0.929 | -0.251 |  |  |
|  |  | 105083959 | + | 8627972 | 8628154 | 8628021 | 8628154 | 8626715 | 8626847 | 0 | 20 | 3 | 4 | 158 | 116 | 0.032416178 | 0 | 0.355 | -0.355 |  |  |
|  |  | 105071953 | - | 16102308 | 16102442 | 16102308 | 16102430 | 16104266 | 16104396 | 15 | 1 | 11 | 8 | 115 | 103 | 0.035388971 | 0.931 | 0.552 | 0.379 |  |  |
|  |  | 105076089 | + | 736379 | 736547 | 736439 | 736547 | 732516 | 732744 | 2 | 0 | 0 | 6 | 154 | 101 | 0.038946344 | 1 | 0 | 1 |  |  |
|  |  | 105073390 | - | 338017 | 338164 | 338017 | 338161 | 338761 | 338888 | 41 | 8 | 47 | 29 | 120 | 120 | 0.040385189 | 0.837 | 0.618 | 0.219 |  |  |
|  |  | 105069546 | - | 2229954 | 2230167 | 2229954 | 2230091 | 2230913 | 2230994 | 5 | 9 | 16 | 5 | 138 | 69 | 0.043589841 | 0.217 | 0.615 | -0.398 |  |  |
|  |  | 105082739 | + | 1319235 | 1319621 | 1319469 | 1319621 | 1318300 | 1318484 | 5 | 0 | 0 | 2 | 355 | 135 | 0.043801269 | 1 | 0 | 1 |  |  |
|  |  | 105062215 | - | 1460838 | 1461007 | 1460838 | 1460955 | 1462304 | 1462409 | 8 | 5 | 10 | 0 | 118 | 73 | 0.043889733 | 0.497 | 1 | -0.503 |  |  |
|  |  | 105067441 | - | 21011468 | 21011656 | 21011468 | 21011623 | 21011742 | 21011901 | 4 | 5 | 6 | 0 | 161 | 135 | 0.044861306 | 0.401 | 1 | -0.599 |  |  |
|  |  | 105069166 | + | 868228 | 868307 | 868243 | 868307 | 867525 | 867742 | 5 | 0 | 2 | 4 | 72 | 57 | 0.046595271 | 1 | 0.284 | 0.716 |  |  |
|  |  | 105073512 | - | 1681132 | 1681281 | 1681132 | 1681278 | 1683867 | 1684043 | 1 | 2 | 10 | 0 | 135 | 135 | 0.049271876 | 0.333 | 1 | -0.667 |  |  |

Note: SS and WS indicates salt stress and water-deprivation stress, respectively.

Table S4. The differential mRNAs, miRNAs and lncRNAs of ileum under salt stress and water-deprivation stress

| **Stress** | **RNA type** | **Gene name** | **Stress FPKM (/TPM)** | **Control FPKM (/TPM)** | **log2(foldchange)** | **pvalue** |
| --- | --- | --- | --- | --- | --- | --- |
| SS | mRNA | *RAI14* | 3.27907 | 0.00714902 | 8.84132 | 0.022985 |
|  |  | *ACACA* | 2.28789 | 0.001832 | 10.2864 | 0.0130679 |
|  |  | *FAM13B* | 3.69114 | 0.00753355 | 8.93652 | 0.0166516 |
|  |  | *LOC105084128* | 92.6172 | 0.809926 | 6.83735 | 0.024783 |
|  |  | *HNRNPR* | 5.58753 | 0.00951393 | 9.19795 | 0.00940584 |
|  |  | *FNBP1* | 2.75686 | 0.00858668 | 8.32671 | 0.0497229 |
|  |  | *ZNF668* | 2.85245 | 0.0101933 | 8.12843 | 0.0346653 |
|  |  | *CUL7* | 2.95043 | 0.00393086 | 9.55187 | 0.0138694 |
|  |  | *B4GALNT2* | 43.1401 | 1.53405 | 4.81361 | 0.00452543 |
|  |  | *ZHX3* | 0.00634976 | 1.55636 | -7.93726 | 0.0355154 |
|  |  | *CA2* | 2.56711 | 51.8217 | -4.33534 | 0.0198963 |
|  |  | *MUC6* | 0.0304934 | 76.4545 | -11.2919 | 0.000117086 |
|  |  | *TMPRSS15* | 0.0907298 | 5.74204 | -5.98384 | 0.0241572 |
|  |  | *SEMA4A* | 0.00875591 | 2.83594 | -8.33935 | 0.0330865 |
|  |  | *LOC105078064* | 1.18249 | 1506.2 | -10.3149 | 5.37E-05 |
|  |  | *ACER3* | 0.0230425 | 6.50261 | -8.14057 | 0.0338281 |
|  |  | *NLRC4* | 0.00907041 | 4.64481 | -9.00023 | 0.00703128 |
|  |  | *AQP5* | 0.107261 | 14.1873 | -7.04734 | 0.0231186 |
|  |  | *BPIFB1* | 0.0497848 | 49.4828 | -9.957 | 0.00304657 |
|  |  | *KTN1* | 0.0105043 | 8.64463 | -9.68468 | 0.011513 |
|  |  | *PICALM* | 0.0120277 | 3.73393 | -8.27819 | 0.0278147 |
|  |  | *FABP1* | 3.27915 | 100.924 | -4.9438 | 0.0215946 |
|  | lncRNA | LNC002765 | 49.0524 | 2.56432 | 4.25768 | 0.0371574 |
|  |  | LNC000855 | 15.4463 | 0.424629 | 5.18491 | 0.0146746 |
|  |  | LNC001664 | 25.8025 | 0.213314 | 6.91839 | 0.02774 |
|  | miRNA | miR-100 | 14021.12896 | 5324.859852 | 1.3968 | 0 |
|  |  | miR-10b | 29715.29486 | 12471.87494 | 1.2525 | 0 |
|  |  | miR-143 | 166406.8413 | 409121.8517 | -1.2978 | 0 |
|  |  | miR-191 | 4289.42317 | 2003.602724 | 1.0982 | 0 |
|  |  | miR-200c | 4468.766088 | 1793.834063 | 1.3168 | 0 |
|  |  | miR-215 | 136.8596804 | 3746.074667 | -4.7746 | 0 |
|  |  | miR-26b | 10051.50632 | 4296.657399 | 1.2261 | 0 |
|  |  | miR-99a-5p | 8922.586932 | 3092.287674 | 1.5288 | 0 |
|  |  | novel 1 | 23615.55993 | 74863.89096 | -1.6645 | 0 |
|  |  | miR-374a | 931.8635876 | 209.2886411 | 2.1546 | 3.73E-154 |
|  |  | miR-125b | 1564.545548 | 646.5866961 | 1.2748 | 4.67E-151 |
|  |  | miR-30c | 1833.144779 | 863.5556543 | 1.086 | 7.97E-149 |
|  |  | miR-374b | 876.0956895 | 208.8086212 | 2.0689 | 1.22E-139 |
|  |  | miR-378 | 3099.892519 | 6852.282916 | -1.1444 | 3.44E-137 |
|  |  | miR-20a | 1356.004053 | 602.4248728 | 1.1705 | 9.03E-120 |
|  |  | miR-195 | 880.108764 | 271.6912175 | 1.6957 | 2.13E-115 |
|  |  | miR-99b | 1412.048715 | 663.8674096 | 1.0888 | 2.19E-115 |
|  |  | miR-1246 | 0 | 405.1367272 | -8.7082 | 4.24E-74 |
|  |  | miR-34c | 31.27430514 | 378.7356372 | -3.5981 | 1.16E-60 |
|  |  | miR-450b | 510.9059052 | 202.5683636 | 1.3346 | 4.91E-53 |
|  |  | miR-15b | 259.4660271 | 55.20227918 | 2.2327 | 1.09E-45 |
|  |  | miR-378c | 128.1416219 | 512.6611666 | -2.0003 | 1.73E-36 |
|  |  | miR-29b | 297.1058988 | 115.6847764 | 1.3608 | 3.42E-32 |
|  |  | miR-365-3p | 145.1625933 | 33.60138733 | 2.1111 | 4.79E-25 |
|  |  | miR-499 | 182.2489375 | 62.40257646 | 1.5462 | 4.13E-23 |
|  |  | miR-142-5p | 110.0135955 | 18.24075312 | 2.5924 | 5.88E-23 |
|  |  | miR-214 | 214.215152 | 89.76370614 | 1.2549 | 7.98E-22 |
|  |  | miR-196a | 220.3039548 | 97.92404307 | 1.1698 | 7.09E-21 |
|  |  | miR-16a | 175.744989 | 64.80267556 | 1.4394 | 7.93E-21 |
|  |  | miR-141 | 168.2723675 | 62.88259628 | 1.4201 | 9.58E-20 |
|  |  | miR-196b | 67.94550364 | 4.320178371 | 3.9752 | 4.67E-19 |
|  |  | miR-484 | 108.0762492 | 27.8411495 | 1.9568 | 8.10E-18 |
|  |  | miR-19b | 120.1154728 | 36.48150624 | 1.7192 | 2.01E-17 |
|  |  | miR-450a | 107.3843398 | 28.80118914 | 1.8986 | 3.07E-17 |
|  |  | miR-132 | 118.5932721 | 49.92206117 | 1.2483 | 1.06E-12 |
|  |  | miR-184 | 0 | 38.40158552 | -6.5314 | 1.61E-09 |
|  |  | miR-1248 | 3.321165147 | 44.16182334 | -3.733 | 1.12E-08 |
|  |  | miR-505 | 88.28764017 | 43.68180353 | 1.0152 | 3.93E-08 |
|  |  | miR-664b | 74.31107017 | 33.60138733 | 1.1451 | 7.43E-08 |
|  |  | miR-488 | 18.26640831 | 79.68328995 | -2.1251 | 1.55E-07 |
|  |  | miR-454 | 37.77825355 | 11.04045584 | 1.7748 | 1.29E-06 |
|  |  | miR-95 | 42.48323751 | 15.36063421 | 1.4677 | 3.32E-06 |
|  |  | novel 455 | 0 | 23.52097113 | -7.4091 | 3.55E-06 |
|  |  | miR-2887 | 1.24543693 | 25.4410504 | -4.3524 | 3.88E-06 |
|  |  | miR-424-5p | 27.53799435 | 8.640356741 | 1.6723 | 6.10E-05 |
|  |  | miR-574 | 109.7368317 | 223.2092158 | -1.0243 | 0.00012083 |
|  |  | miR-490 | 17.15935326 | 59.04243773 | -1.7828 | 0.00012258 |
|  |  | miR-335 | 18.54317207 | 4.320178371 | 2.1017 | 0.00022724 |
|  |  | miR-22-5p | 29.19857692 | 11.52047565 | 1.3417 | 0.00023844 |
|  |  | novel 665 | 0 | 14.88061439 | -4.8954 | 0.00025609 |
|  |  | miR-9-3p | 23.52491979 | 8.160336922 | 1.5275 | 0.000409 |
|  |  | miR-424-3p | 15.91391633 | 3.360138733 | 2.2437 | 0.00042935 |
|  |  | miR-320a | 9.548349799 | 37.44154588 | -1.9713 | 0.00076795 |
|  |  | miR-378d | 5.673657127 | 28.32116932 | -2.3195 | 0.00076529 |
|  |  | miR-199c | 60.8880277 | 130.0853709 | -1.0952 | 0.0012453 |
|  |  | miR-19a | 11.34731425 | 2.400099095 | 2.2412 | 0.002959 |
| WS | mRNA | *ACACA* | 2.35572 | 0.001832 | 10.3285 | 0.0128794 |
|  |  | *LOC105075135* | 37.7243 | 1.02228 | 5.20563 | 0.0158072 |
|  |  | *RPH3AL* | 5.14392 | 0.0100504 | 8.99948 | 0.026097 |
|  |  | *FNBP1* | 6.1755 | 0.00858668 | 9.49024 | 0.0195505 |
|  |  | *ZNF668* | 4.23959 | 0.0101933 | 8.70015 | 0.0228413 |
|  |  | *LOC105076960* | 33.3651 | 1.40627 | 4.56839 | 0.0416344 |
|  |  | *LOC105074968* | 19.1172 | 0.848622 | 4.4936 | 0.0402799 |
|  |  | *LOC105069855* | 17.7283 | 0.638504 | 4.79521 | 0.03579 |
|  |  | *JUP* | 40.8352 | 2.8905 | 3.82042 | 0.0446692 |
|  |  | *MOV10* | 13.1909 | 0.862068 | 3.9356 | 0.0478294 |
|  |  | *CIC* | 7.60372 | 0.498513 | 3.931 | 0.045495 |
|  |  | *SUPT6H* | 6.51788 | 0.336444 | 4.27596 | 0.0485091 |
|  |  | *HADHB* | 21.4061 | 1.05489 | 4.34286 | 0.0473013 |
|  |  | *CLK3* | 24.8707 | 1.81925 | 3.77303 | 0.0499357 |
|  |  | *PRUNE2* | 5.59832 | 0.175453 | 4.99584 | 0.0384905 |
|  |  | *HIPK2* | 5.83571 | 0.325831 | 4.16271 | 0.0477221 |
|  |  | *LARP4* | 17.1681 | 0.965866 | 4.15176 | 0.0495211 |
|  |  | *CUL7* | 7.37194 | 0.00393086 | 10.873 | 0.00384734 |
|  |  | *B4GALNT2* | 23.9605 | 1.53405 | 3.96524 | 0.0202717 |
|  |  | *PRRC2C* | 5.44592 | 0.336464 | 4.01665 | 0.0458849 |
|  |  | *ALDOA* | 140.196 | 6.17304 | 4.50531 | 0.0224916 |
|  |  | *ZNF469* | 2.39161 | 0.111995 | 4.41648 | 0.0334111 |
|  |  | *CA2* | 2.72976 | 51.8217 | -4.24671 | 0.0229637 |
|  |  | *LOC105081265* | 0.00711723 | 9.12981 | -10.3251 | 0.0155618 |
|  |  | *MUC6* | 0.457754 | 76.4545 | -7.38389 | 0.000257443 |
|  |  | *ALB* | 1.24954 | 37.9063 | -4.92297 | 0.0110861 |
|  |  | *PPP6R3* | 0.820857 | 20.1892 | -4.62031 | 0.0219135 |
|  |  | *PUM1* | 0.00533123 | 11.3065 | -11.0504 | 0.00289545 |
|  |  | *LOC105070098* | 0.176006 | 10.1084 | -5.84379 | 0.0467118 |
|  |  | *LOC105073959* | 82.4139 | 951.615 | -3.52942 | 0.0366892 |
|  |  | *LOC105078064* | 0.912562 | 1506.2 | -10.6887 | 6.24E-05 |
|  |  | *PGC* | 0.0610927 | 10.8975 | -7.47878 | 0.0298408 |
|  |  | *CDH11* | 0.00935739 | 13.4817 | -10.4926 | 0.00103977 |
|  |  | *TBC1D14* | 0.0148821 | 5.97046 | -8.64813 | 0.045719 |
|  |  | *AQP5* | 0.233467 | 14.1873 | -5.92524 | 0.0324476 |
|  |  | *FGF7* | 0.014292 | 4.93104 | -8.43054 | 0.0356343 |
|  |  | *HNRNPC* | 0.055565 | 19.9838 | -8.49044 | 0.0154246 |
|  |  | *EPS8* | 0.0116573 | 4.99896 | -8.74425 | 0.0304811 |
|  |  | *FABP2* | 11.3 | 141.271 | -3.64407 | 0.0348294 |
|  |  | *LOC105071055* | 0.0266478 | 4.99228 | -7.54954 | 0.0423108 |
|  |  | *LOC105067892* | 0.135987 | 16.965 | -6.96295 | 0.0247453 |
|  |  | *RPLP1* | 8053.12 | 87211.7 | -3.4369 | 0.0276685 |
|  |  | *FGB* | 0.270628 | 10.3012 | -5.25036 | 0.0384953 |
|  | lncRNA | LNC001509 | 0.271115 | 0.0053681 | 5.65835 | 0.0256902 |
|  |  | LNC000105 | 69.4612 | 3.42763 | 4.34092 | 0.0161942 |
|  |  | LNC000948 | 79.0069 | 1.07826 | 6.1952 | 0.0264818 |
|  |  | LNC002464 | 34.7935 | 1.30424 | 4.73754 | 0.0492923 |
|  |  | LNC002465 | 20.3171 | 0.104317 | 7.60557 | 0.0224048 |
|  |  | LNC001684 | 29.7281 | 0.417077 | 6.15537 | 0.038185 |
|  |  | LNC001664 | 39.7746 | 0.213314 | 7.54273 | 0.0154275 |
|  | miRNA | miR-101 | 9832.451379 | 2361.820574 | 2.0577 | 0 |
|  |  | miR-148a | 31996.21473 | 96490.27359 | -1.5925 | 0 |
|  |  | miR-215 | 139.5871415 | 3883.617312 | -4.7982 | 0 |
|  |  | miR-26a | 13988.63762 | 64504.67401 | -2.2051 | 0 |
|  |  | miR-26c | 13987.46743 | 64503.18108 | -2.2052 | 0 |
|  |  | miR-26b | 1126.393005 | 4454.4155 | -1.9835 | 1.76E-262 |
|  |  | miR-451 | 1569.561283 | 679.2846785 | 1.2083 | 7.51E-163 |
|  |  | miR-1246 | 8.358511467 | 420.0119184 | -5.651 | 3.04E-85 |
|  |  | novel 73 | 564.5338645 | 230.9070262 | 1.2897 | 1.24E-63 |
|  |  | let-7e | 373.1239519 | 1284.917978 | -1.7839 | 3.99E-62 |
|  |  | miR-19b | 220.163192 | 37.82097844 | 2.5413 | 8.01E-47 |
|  |  | miR-106b | 315.617393 | 106.9935574 | 1.5607 | 1.75E-43 |
|  |  | miR-29b | 290.8761991 | 119.9323132 | 1.2782 | 2.38E-33 |
|  |  | miR-141 | 201.1057859 | 65.19142336 | 1.6252 | 2.18E-29 |
|  |  | novel 109 | 231.5307676 | 103.5100462 | 1.1614 | 1.35E-24 |
|  |  | miR-142-5p | 103.3112017 | 18.91048922 | 2.4497 | 3.01E-22 |
|  |  | miR-147 | 168.5075912 | 80.12075695 | 1.0726 | 4.62E-17 |
|  |  | miR-486 | 121.8670972 | 44.29035633 | 1.4602 | 9.01E-17 |
|  |  | miR-34c | 131.7301407 | 392.6414735 | -1.5756 | 1.48E-15 |
|  |  | miR-362-5p | 118.8580331 | 46.28093414 | 1.3607 | 2.13E-15 |
|  |  | miR-148b | 384.157187 | 807.6769474 | -1.0721 | 3.64E-11 |
|  |  | miR-1248 | 0 | 45.78328969 | -6.0974 | 8.18E-11 |
|  |  | miR-19a | 28.58610922 | 2.488222266 | 3.5221 | 7.69E-09 |
|  |  | miR-184 | 1.671702293 | 39.81155625 | -4.5738 | 9.45E-09 |
|  |  | miR-96 | 67.03526197 | 30.85395609 | 1.1195 | 6.35E-08 |
|  |  | miR-502a | 51.98994133 | 19.90577812 | 1.385 | 1.20E-07 |
|  |  | miR-199c | 44.96879169 | 134.8616468 | -1.5845 | 2.51E-06 |
|  |  | novel 455 | 0 | 24.3845782 | -7.1885 | 2.73E-06 |
|  |  | miR-18a | 23.06949165 | 5.474088984 | 2.0753 | 1.95E-05 |
|  |  | miR-210 | 32.09668403 | 12.44111133 | 1.3673 | 3.59E-05 |
|  |  | miR-153 | 13.03927789 | 0 | 4.7116 | 3.93E-05 |
|  |  | novel 665 | 0 | 15.42697805 | -4.943 | 0.00025626 |
|  |  | miR-875 | 11.3675756 | 1.492933359 | 2.9287 | 0.00057872 |
|  |  | miR-144 | 8.024171009 | 0 | 4.0044 | 0.0016243 |
|  |  | miR-491 | 21.39778936 | 9.455244609 | 1.1783 | 0.0017226 |
|  |  | miR-136 | 7.188319862 | 0 | 3.8525 | 0.003084 |

Note: SS and WS indicate salt stress and water-deprivation stress, respectively.

Table S5. The differential mRNAs, miRNAs and lncRNAs of liver under salt stress and water-deprivation stress

| **Stress** | **RNA type** | **Gene name** | **Stress FPKM (/TPM)** | **Control FPKM (/TPM)** | **log2(foldchange)** | **pvalue** |
| --- | --- | --- | --- | --- | --- | --- |
| SS | mRNA | *ACSM3* | 33.2666 | 0.737881 | 5.49454 | 0.00933491 |
|  |  | *OCIAD1* | 18.2207 | 0.0278251 | 9.35498 | 0.0135364 |
|  |  | *TLR8* | 1.9656 | 0.00481901 | 8.67202 | 0.0406103 |
|  |  | *TCF4* | 1.4121 | 0.00245515 | 9.16782 | 0.0119036 |
|  |  | *CDH11* | 1.12162 | 0.00492323 | 7.83177 | 0.0201964 |
|  |  | *MAP7* | 1.11452 | 0.00538836 | 7.69236 | 0.0419581 |
|  |  | *PCNX* | 1.76755 | 0.00486601 | 8.50479 | 0.0182906 |
|  |  | *VPS13D* | 4.33802 | 0.189049 | 4.5202 | 0.0142954 |
|  |  | *TENM1* | 2.59988 | 0.00102577 | 11.3075 | 0.00578872 |
|  |  | *LOC105071055* | 5.33548 | 0.00946612 | 9.13863 | 0.0286115 |
|  |  | *PKP4* | 5.46498 | 0.00704166 | 9.60009 | 0.00972754 |
|  |  | *TTPAL* | 0.00342377 | 1.45216 | -8.7284 | 0.0375286 |
|  |  | *SDS* | 1.76171 | 36.1055 | -4.35718 | 0.0209654 |
|  |  | *LOC105061856* | 3.26674 | 107.533 | -5.04079 | 0.00998733 |
|  | lncRNA | LNC001438 | 2.52503 | 0.154357 | 4.03196 | 0.0254415 |
|  |  | LNC003417 | 0.711252 | 0.0506229 | 3.8125 | 0.0481902 |
|  |  | LNC003695 | 3.80583 | 0.335135 | 3.5054 | 0.0444867 |
|  |  | LNC000406 | 3.36931 | 0.36793 | 3.19495 | 0.0357949 |
|  |  | LNC001770 | 1.57267 | 0.139558 | 3.49428 | 0.0251271 |
|  | miRNA | miR-26b | 4943.673657 | 2038.760016 | 1.2779 | 0 |
|  |  | novel 1 | 3813.19163 | 7764.047403 | -1.0258 | 1.53E-237 |
|  |  | miR-195 | 385.5074824 | 171.6424329 | 1.1674 | 4.86E-24 |
|  |  | miR-222 | 54.99445426 | 222.695054 | -2.0177 | 1.15E-22 |
|  |  | miR-23b-3p | 398.9829447 | 189.246785 | 1.0761 | 2.74E-22 |
|  |  | miR-199c | 115.6340346 | 319.3429469 | -1.4655 | 5.29E-20 |
|  |  | miR-365-5p | 109.0784043 | 44.18692375 | 1.3037 | 6.25E-09 |
|  |  | miR-142-5p | 74.11504267 | 27.63883278 | 1.4231 | 3.68E-07 |
|  |  | miR-2889 | 18.75638672 | 0 | 4.4134 | 7.49E-06 |
|  |  | miR-1307 | 28.77193302 | 76.40288808 | -1.409 | 1.47E-05 |
|  |  | miR-331-3p | 25.49411787 | 61.4391888 | -1.269 | 0.00038608 |
|  |  | miR-218 | 37.3306726 | 16.72413449 | 1.1584 | 0.0017511 |
| WS | mRNA | *CORO1C* | 3.9907 | 0.00827055 | 8.91444 | 0.00892906 |
|  |  | *PLIN2* | 69.0454 | 7.57501 | 3.18823 | 0.0327774 |
|  |  | *TCF4* | 1.88279 | 0.00245515 | 9.58285 | 0.0070336 |
|  |  | *PCNX* | 0.953009 | 0.00524363 | 7.50578 | 0.0386617 |
|  |  | *HILPDA* | 60.893 | 5.60918 | 3.44041 | 0.035925 |
|  |  | *SPINK1* | 1084.65 | 80.235 | 3.75685 | 0.0156359 |
|  |  | *TOR3A* | 55.173 | 3.67241 | 3.90916 | 0.0162688 |
|  |  | *PKP4* | 1.575 | 0.00704166 | 7.80522 | 0.0450833 |
|  |  | *SERPINA6* | 4.13361 | 45.6025 | -3.46364 | 0.0373954 |
|  |  | *UPP2* | 2.55952 | 47.5701 | -4.21611 | 0.025006 |
|  |  | *CTNND1* | 0.146318 | 6.46522 | -5.46552 | 0.0351396 |
|  |  | *ASH1L* | 0.00128557 | 1.29061 | -9.97143 | 0.0265173 |
|  |  | *SDS* | 3.37939 | 36.1055 | -3.41738 | 0.0452357 |
|  |  | *LOC105074317* | 0.0088971 | 0.913627 | -6.68213 | 0.0460671 |
|  |  | *ETNPPL* | 8.01767 | 96.8007 | -3.59376 | 0.0390748 |
|  |  | *LOC105061856* | 3.51283 | 107.533 | -4.93601 | 0.00917229 |
|  | lncRNA | XM_010950341.1 | 0.00815546 | 2.41192 | -8.2082 | 0.0200664 |
|  | miRNA | miR-101 | 2562.638925 | 48187.49037 | -4.233 | 0 |
|  |  | miR-148a | 448673.1193 | 175085.5561 | 1.3576 | 0 |
|  |  | miR-26a | 42099.09887 | 19063.84577 | 1.143 | 0 |
|  |  | miR-26b | 7139.478744 | 1711.938199 | 2.0602 | 0 |
|  |  | miR-26c | 42094.12087 | 19062.66319 | 1.1429 | 0 |
|  |  | miR-99a-5p | 18690.66511 | 7546.659954 | 1.3084 | 0 |
|  |  | novel 1 | 2790.768547 | 6519.437905 | -1.2241 | 0 |
|  |  | miR-139 | 1584.204879 | 454.8513805 | 1.8003 | 1.33E-91 |
|  |  | miR-7 | 3643.036098 | 1578.897496 | 1.2062 | 2.67E-87 |
|  |  | novel 22 | 3455.073768 | 1599.149247 | 1.1114 | 2.31E-67 |
|  |  | miR-20a | 330.2644043 | 720.341494 | -1.1251 | 1.15E-60 |
|  |  | miR-125a | 1395.899239 | 545.6147044 | 1.3552 | 3.48E-45 |
|  |  | miR-365-3p | 941.5282005 | 338.3668541 | 1.4764 | 2.73E-37 |
|  |  | miR-450b | 124.9649097 | 318.5585717 | -1.35 | 1.48E-33 |
|  |  | miR-215 | 19.05371563 | 145.901304 | -2.9368 | 1.54E-32 |
|  |  | miR-199c | 95.95519855 | 268.1509276 | -1.4826 | 2.27E-31 |
|  |  | miR-106b | 10.81427104 | 75.53755458 | -2.8043 | 4.61E-17 |
|  |  | miR-335 | 62.99742016 | 157.4314983 | -1.3214 | 4.97E-17 |
|  |  | miR-155 | 295.4184199 | 92.09373092 | 1.6816 | 2.53E-16 |
|  |  | miR-340 | 125.6515301 | 29.71242362 | 2.0803 | 6.97E-11 |
|  |  | miR-19b | 16.3072341 | 59.42484724 | -1.8656 | 6.25E-10 |
|  |  | miR-128 | 198.0899806 | 73.46803253 | 1.431 | 1.58E-08 |
|  |  | miR-339b | 27.63647042 | 67.55511241 | -1.2895 | 5.91E-08 |
|  |  | miR-1 | 19.74033602 | 54.39886514 | -1.4624 | 1.90E-07 |
|  |  | miR-142-5p | 2.574826437 | 23.20821148 | -3.1721 | 1.12E-06 |
|  |  | miR-331-3p | 21.28523188 | 51.59022808 | -1.2772 | 2.44E-06 |
|  |  | miR-29b | 27.46481533 | 56.02491817 | -1.0285 | 1.30E-05 |
|  |  | miR-217 | 20.77026659 | 45.97295396 | -1.1463 | 2.76E-05 |
|  |  | miR-543 | 32.27115801 | 4.582513096 | 2.816 | 3.01E-05 |
|  |  | miR-455-3p | 178.6929547 | 79.97224467 | 1.1599 | 3.08E-05 |
|  |  | miR-34c | 5.32130797 | 20.84304344 | -1.9697 | 0.00016768 |
|  |  | miR-15a | 12.70247709 | 30.59936164 | -1.2684 | 0.00029924 |
|  |  | miR-29c | 7.724479311 | 23.20821148 | -1.5871 | 0.00037931 |
|  |  | miR-370 | 146.5934518 | 70.21592647 | 1.062 | 0.00079842 |
|  |  | miR-365-5p | 85.999203 | 37.10357377 | 1.2128 | 0.0022121 |
|  |  | miR-424-5p | 10.47096084 | 23.20821148 | -1.1482 | 0.0028655 |
|  |  | novel 135 | 9.955995556 | 22.46909647 | -1.1743 | 0.0029746 |

Note: SS and WS indicate salt stress and water-deprivation stress, respectively.

Table S6. Sequence of differential novel lncRNAs in the ileum and liver of camel under salt stress and water-deprivation stress.

| **lncRNA** | **Sequence (5' to 3')** |
| --- | --- |
| LNC002765 | GgTTTGAAGATGTCTCATTTGGATTGGACTAAttccaaattctgttttcttgctttttaatacTAATATACTTTCAGGCTTTATACACATAAATCAGACATTATATTGATGTTATCACAAATACCACTTTATGTCTCCATGTTTGACTTGGGACGCTGGTAGTTtagaggtggagaggagggaattCCATGTATAACATCAACATCTGCATCAAATCTGaactcttctgtctcttctgcaCACAAAACTGGATAATATGATAGTTATGCATGTTTAGAGGAAACCTTCAATTTATAATTATGCTTAgaattttgggaaaatttttaaagatatttatcaGAATTATGGAAATTTGTGATAGTGGGTGAAACAACTGCCTGCCTAGAATTCCATGcacaacaaaaatatctttcaaaaatgactgCAAATTGACACTTTAGATGAAAACTGAGATCAGCACCAGTCAGATCCTCATTAAAGGAGATTCTAAATGATACACCTTACGCAGAGGGAAGGCCTACAATGTGAGAAGTGAAGAGCAAGGGAAATGTATCAATTCTgtgtaaaaagaaagaagcctgaATACTTAAGTATAATGAATGCCAAtgtagtaaaaaacaaacaaacaaaacacccagaACAATTATGTAAGTATAGAAAGAGCAAGTGATGTTAAAGCATTCAGTGGACTTCTGTTACCCAGAAGGGTAAAGTTATAAACTTTTTTGGTAATTTAAACATGTACATTATATTTCCTAGGGTATCCTCTTAAAAAAACATAAACTGAATATTTACTGTCTAACTAGTTGAGGGCACAGGAGCTTCTGGAGTGctagtaatgttttattttttgccctGAATGATGGTTACAAGAaattttgctttgtaattattCGTTAAATGATATgtacttcacattttaaaataatgttatatttcataataaaaataataaaattgattcaTGTGGTTTAACTACAGTAACAGATTAAAGGAAATGGttcatatgattatctcaatatatttagaataagtgttcagtaaaattcaacatccacttatgatttaaaaaaccTCATAGCTGGGGAAAAATGGGAATTTACTTAGATATAAAGGTTTTCTTAACATACATAAATAGACACACATTAATAGAGAGATATTACAGGTATTTTACAGGTAAGATCAGAAATAATATAAGGATGCCCATTGCTATTTCTGGGTGATATTATACTAGAGGTCCCTGCAGAAAATTAAGacatgga |
| LNC000855 | accTGACTACTAATACATTTAATTGTCAGtccaaaatttttaataatattgaattcaatttatttttctttcttttgatattCCTTGgacttctcattttatttttcagatgttacCAGGAAACCAGTTGGTAACTATTCCTGCATTCTTTGCCCCGTAATAACCCTTTAAATGAGTTCTAACTTTCCTGCCTTTGTATCTATATGCCATCAATtggctctttcttcctttcttccctctccaatTTTTAGATCTCTACTGCACCAATATTAATTTCTTCCATATCTTCTTCCCTGATTCCATCaattcctttttatccttttgtattacaaacattttctcctctgtaactttaagggtttttgtttctttctctcttttctttttgccttttttttcgCTTTTTTTCCCAATGTCTCCAGGAAATGGCcctgatttttataaatacaactatactgtttgttctttctcttataCCTTTTCAGtgaaacaatttaatttttattaaatttcaattatattatatattagtaGAGGCAATTTCGTTCACATGGTATTTTCAACTGTTAATGTACCTCTCAATTCTCAGttttttatatgaaatacatGGAAACCATTATCTTGGGATTTTTCCTGAGATGTTGTATTACAATACAAATATAACTCAATTAGAACTTTAACATGCTTTATGTAATTAATATGTTAAATGATAATTTCTATTCCAAAAGGAATTCAATTCAAGTACATGTCAATTTAATGTTTGTATGAAGCCGTTTATGAGTACTGCTATGCCCAGAGTATTCAAAATATGGTGcttgtaatttattctttttctttcttttcaaggaccacttttgtgtgtgtgtgtgtatgagtgtgtcttcattttattctaaCAAAGGTAGAAGgcccaaaacaaaccaacaattGACATATCTCTTATCCATTAACTCAGAGAGAGTGCTGCTCTACTGCCTTGCACTCTGGGATGTCCCTCTTTGTCCCTTTGCCACGTATGGAAGTCAAATAACACTTCCTTTGACTGGTCAGATGGTCTCTAAAATTTGCAGTTTCAATTCTCAGAAGACAAGTCTCAGAAACAGCTATGGAAATGCTTGAAAATTTGTCCTTCTGCTTTCTGACACTGTATCCcattttgaatgaatgatttctttctctcctttagtTAATGACAAACGTGAAGAAGAAAATGGTAAGTCTCATGAATGTACCTCCCTGTGTACCTAAGCATGGTTTCAAATGTACTTATGCTTTTCATTTCAGCTTCCAAGGATCATCTTTCTCAACTCCGATATGTGCTTATTTTACACGACTTCCTCTCCAGTCATATTTTTGCAACCGAAGTTTCCCTCTACATTGGCTATTCAATTATTCCCCCAAAACTGCTCTTTGATTTGTCCTGTAATTGCAATGTTTGCAGGTATTCTGTCGGAGTTAAGTACACTTTACTAATTATATACCTTTGGAATAATGCCTTTTGAAAGTGGTTGAAGTGAAACTATTTCCGAGACATGCTCAGGTCACTTGATTTAGGCTCATCTCACTGCcatttaatacttttttccttttcatggaaattagttaagaaaattttcatttagaCATAGTTGAAAGAGAAATTTACATGCACACTGTTAtgtattcatctattgatgaaagTATTCTTAATAGTGAGACCAAGGGGATATACCGTATGGTACCTCTAGAAGCACTGGTGTAAGATACTTCAATTCATTGTTTGTGGCTGCTTTATATCACTTAGATGTCATAAAAATCCAACTAAATATGCAGATTAAGTAAAGGAAAATTACTTACTGCATCTTACAAACTCAATCAGAAAAGCCTAATTGTATCTAAACTGATGATGAACATCTGAAAGCTTAGCACGAGAACTGCTATTTGTAAAAAAGTTCTCAAACAAGAATATTCactcattttatctttgttttcattttgagggAAACATTTTTCGGATAAAACTGAGCTCTGAACCACTGGacatgtatgagtgtgtgtgtgtgtgtgtgtgtgtgtgtgtgacttcatGCAATGGTATCTGATTTTATCTGTGTGCACATCCACGTGGGttcaaattgaaatataaattctGTGTTAAATTTGAACTACTTTCccattttacattatatttacattgacctctccattttatttttcagaaagaccTCAGAGAACAGTAGatgaacaACGAAGACTatatggtaaatttttttaacGCACATTGTTCCATAACTTTTAAACTATTGCTAACTTTTCCTACCTTTCTATCAATTTACCAGTTCCTGGCTGAaacttctcctcttccctctccagtaTTTGGGATAAGGAACACTCTCATATTAatgtcttccttctgtttttcctgtttcacTCCCTAATTCTCCTTGTTTTTCACTgcatgccttttcttttttttatttttaattcttcagagACTTGCCCtgattgattattatttttgtagAGATGACTGGCATTAGGCTGGTCACTATATCACTTCCATTCTATATATGTTTTCTGGCAAATGACCGCTGAGGTAGAATTTCTGTCACCTTTCAAGTTCTGGGGAGAATAACTATGCAACTGAAACTGATCCAACTTAAGTGAAAAGAAGACTCCAATTTGCCGAGTGAAATTTCTCAACCTTCTGATCTTTCAGGCACGATAAATTGACGTCATTCTTGGTCAAGTGTGAGCTCTAACTACAGTTCACCTTGATCGTATTACCCAATTGTTCCCAAGTAAACAAATGTATTAGCTAATACTTTGTGacctcttttctttcaaaactattAGCTctgcaaaatgtaatttttcttaatgagtttAGAGGGACTTTACCTGCTAAGAGAAGGGCATTTAATTACATATCtatcattaataaaatgttatttcgTTAATGTGAGAAGTGaagttttggggaaaattttttaaagtctttcagAACACACTAATTTAAACACTAACTTTTTACCTCCTCTATCCTCTTGATATTTTCCATCTACATTTTTGTTTCAGGAGGAATTCAGTTTAGCCATGTATCTCTTCACAGTTTTTAGGAGGCTCAAAGTGGTGACTGTTCCATTAGCCCCTGTGCACAGAAGGGccctttactgtatgttttctCATCACTTTTTAAGTATATCTTCCCTATATTCCTTTacattagtattttattttgaacaaacttaGAAGAGCCAACATAAGCCCAATAGGAGCTCCTATTCTACCTCATCTTATGGAAAAGTCTCCAATCTTAGATCCTTTGCAAAATAGAGGTTATGGAATTAAAACTACAATACTTCTCATTGGCTATATGGACTTCAAACTTCAAGGCTTCAAATCTCATAAATGTACGCTCATTTGAACCTAAGAGAAAACTAAGTAAATACCATATATTTTTGAAGCCCTCGATTCCTCCCTTCCTGACATCATCATCTTACATGGAATGAgttgctgttttcttatttttattgatggaTCAGTGTGAAGAGGGAAAAGTCATGTCTCATAAGGTATCTCACTGTGTACTAGAGCgtgctttcatttgtttcttgtaGCTTTGTCTTCAATACTCAATTTCCCTATGCCCAAACAACACTGCACATAGAGCTTACGTGTTTGTCTTGCTATTTAGTCTTTCCAAAGGAAGTTTGCCTTCtccattgttttttaatttctcccatTAGTACTTCTGTGATTCCTCTTCTTGATACTATCTAGGCAGATTTTTTTTGGTCAgtaaatattagtaataatacaTTTCAATGATTTGCCTTTTCCAAGCTTGTGAAGTGTGAGTTTTCTCCAATATATTCTCAGTCCTTGATGTACAAATGCTTTCAAACTAACATTTGGTACTTAAACTCCATCTTTTCATGGGATTTTATTAAGACATCCTTCAGTAGGCTTTAGCTACACTAGAATGTCGTACTACACGAAGTTATTTATTGATCGGTTGAGAGAAATATGAATAGAATCATCCAGGAAAAGAGCCCTGAATTTCCTCTACAAGACTGGTTCATGATGTTAATTCAACATACATGTCTTCTTCACACAACTTAGGTATTACAATAATGAAGTTGATTGCATCTATGTATTAACAGGCAAAGAAGTATTATAGCTAATGAACCCAATAATAATAGCTCTAGTAATgtcaaaataaagaacattttaaaaatgtgcactTCAGAACTGTTATTATGATACTTTTCCTTAATTtagatgatatttatttttattcctattctcCTTTGGGAGAATGTTTTCTTGACAAAATCAGCTCTAAttcactgtgtgtatgtgtgtgtgtgtgtgcccctcTGTGTGCGTGTACTTGCATTCAGATAATTtaactttatatgtatatattttgtaacaactttGGTTTCCTGGTTTTCCATTTCCATATTATAGAcattaatcttccttttttttttttttaagaacctgAGGGAAGAGATGACAAGTATCCTAAAAACTCTGATGACAGGATTCTAAAAAATGGTAAGTATTCTAAAAACTGTAGAATCCCTTTTTCACGGCTGATTTCTTGAGCCCTTAACATTCCCAGTACTTAATCCCtatgcttttctttgtctttcccttCTCCTGGATGTCGACCTTTACCTCTCGCAATTTTACGTATGGACCTCCCCATAAAATGTTCTCTCCATCTAGTCTCCACTGGTACAATCCATTTCAAATCCTTATTTTTCCATCCTgtatcatttctttcctgttaACATTATCTTTGTATGTTTTTAGATACTTTTTATAGATTGTCTAAAAGGTTGATGAGATTATGGACATTAGTATAAAAGGCACTGGGTTCCACTCTGTTTAGCTTCCAGGAAACATCCCTAGTTAGGACCTCCGTTACTTTCCAATTTCAATGGCCCACATTTGTTTTGATAATACTTCCCC |
| LNC001664 | AAAGGAAAGACAGTAGGAGGGAAGTCATTTTCTCCCCGGATTACCCAGCAAGAACATAGAGAATGAAAGCCTTCAGTCACCTCACTCTGCCTGCCCTCCTTTTTGTAATCTCAGATGTTATCATAATTCCTACCATGACTGATAATTGTCCTACCACTGAAGCTACAACACAAACTGCCACGGTTTCTGGGAAGACAATAATCAGTACTTCGAGCATAAAACACTACAATCAACCCTCACACACTACATATGAAACCACCACTGAAATTACATTAAGACACAACTACATACCCACTTCAACTGCTGTGGATTGTACCACAACAACCACTGAGAAGCCTCCTTCGCACTCAGCTCCCACAGATCCACACACTAACAATGAGTCTACTAATGCCGCCTCAGATGACACAATTCCTCCTACGACCAGGGAaaaccctcttccctcctcagctACCACAGTTTTTCTAAAAACCACGGAAAAGCCTACTCCATCCTCAGCCACAATGGCTCCACCGACAACGACGGGGCCACCTACTACCTCCTCAGATGTAATAATGCCTCTTACCACCACAGGGAAGCCTACAGCCACCTCAGCTAACACAGATCCTCCTACAACCACAGTGGAGCCTATAGCCTCCTCAGCTTCCACAGCTCCACCCACTACCACTGAGCCTCCTGCACTCTCCTCAGCTGCCACAATTACTTCTATCA |
| LNC001509 | TGTGGATACTAAAGATGTGGgtaaaaaaatagcaaaactcTCCTAAAAGTTCCTGTCTATACTTACCTattatactaaaaaagaaaaaatcctctCTTTCTCCTACCATAGTTTTGGTTTGTGAGGAACAATAGATTTTCCTTTCTAGAAACGATTATTACACTCTGATAAAGGTTCTCTTGTTCATAAATCCCTCCTATCTTCACTTTTTTCTGAGTGCAGCCTTAGATTTTAGCAGACCTGCTTTTGAAGTTCAAAGAGAGAAAAGTCAGCAGCCTCCAGTGTATATAACAAAAGACAACGCAGTagtatccttctttctttttattgaaaaggAATCATTTCTATTCTAGCGCAaacaatgtatattttacatCATAAAgcaagatataatttacatattacaTTTCTTATGAGATCTTTATATCCTAAATGTCATGTCATTCCCTTTTTTATCCGATCACCTCTTCTGGTTTGATTTAACCTGCCAAGTATCTTACAAGTCAGCATTTTGTTTGGGGAGGGATTGGGGACACAGATAGACAGTTCGTAAGTATTCATCTGTTATCTGGGTATAATGATGGTGATGAACAAGGACATGCCTCTGACCTTTGTGGCTCTGAGGCCCTCTATTGATACCTGGCTTAGGAGCCAGAAGGCTGTTCCTCCAAATCACATCTCAAAcccctattttttaaagaatcaataaCGAGTGAACAAAATTGAGTGCTTGTACCCTGTGCGCACTTCTCCATCGGGTGCTTAGATAATAGATGCGTGTGGTATTGGTTGGCAGTAAAAGCTCCTGTGGCTGACAGCATTGGTTGCTCTGTGATATCCCCAGACAGCACTTGCCCAGGCACTGAGTAAAACTGCCTAGTTTCACCGAGCATTGGGGTTATGGAGTCAGACTTTTCTGTTCACCACTGAAAATGAGTATCAGGATGAAGTTCTCAAATGGTTTGGTTTTACATGACTCTTACTCCCTCTTCTAATATTCCTCGCTTTTGACAAAATCCTTTCATGTATGATTTCTCTCCCCTGAACTATAACTCAGATTCCTCTTTTAGCCGTTCTCTCTGAATTCCAAAATCTCAGCAATTTCTTGGATTGCACTCTTTTCACAAAGAATGTTTTAATCTGTTATAATACACTTTCCTTTGAAAGTTGATAATATGCATCAGCTAGTGGGACAAAAGAATTAGTTGAAGAAAACTTTAGGGAGCAAGTGATGTGTGTTGTTCAGCCAGGCTACAGAAGCCATTCAGTAAGAAGTGTTCTAATAGTTGACTAAAAGATGAGGGAATCAGTTGATGAAAGCAAAATGCATACAATGGTCTTCCTTCTTATTAAGAGAAGTACGAAATCaacatttgaaatacattttcagattGGTGGCTGTTTGACTGAAAAAACACCGCCATTAGCTCAATCTGATATGCCAGGGGAAATAACAGACGTGTACTGAGAGTGCATCTTAAATCCCAAATCCTTGTCTGTCACTTAGAGTTGCCATATTTGGCCAAAATCAATCTAAAGCTTTTCAATCACTGATAACAGAAATTATTAGAACCCAAGTTGGTcaggatgaaaataaaagtcaaataaatgtcttaaattaatttttgatcaAATGATGTGCTTTTTGAGAGGTCTGAAAATATAAACCTATTTCTCATTTCCAAATGTAAGTTTTTACAGCAAGGTAGCATTTCCTTGCTAGACAACAAGGCTTCTGGGTACCGTATCCTTCTATTAACATTGTATACTTTTATAGTCCCTTTCCTCCCATGGCTACAAAGTGCTGGACATACAGAGAATACAGGAAAGGACAAAGTAGCAAATGATTAGAGCCTCACAACacccctgtgaggtaggtatatTGTTTAAACCCATTTTACAAAAAGGTAAACCAAGGCACAGCATGGTAATGTGTTTTGGTCAAGTCTACCTGACAAGTTACAAAAGCATGgccagaaacaaagagaaagaaacaaagaaaaggtcagtcctggctcccagccctaGATActaaactttatttcaaaatattaggaaacaaaaacagcCCACAGTTGGCTTTTAAAGGTTTAGAATTTAGGCAGACTTTTAAGAATTCCACTTAAACATGTGTCTTGCAGATTCCTGTTGAGTGGGAAAAGGGTGGGTGACTAAATGGCTTTCCCTCTTCTAAGTGTAGTTCCCGTGACAGATCAGGATGCATCCGCATTCCACCAGAGCGTGGGCTTCAGGCTTAAGGACATTTTCATAGCCTCAAAtagtttctaatttatttaaagcCCTAATGTGAAAATTGGACCTGTTCTCAAACAGTTGCCCTATCAATTGCTCTGtgtagagaaagaaaggaaacaggatCCCTAATAAAAAGTCTCATCACTGTAGAGACAAGGACACAGGTGATGGTGTGAAATTTGCTCTTTGTAGTGTTTGAAAAACTGCAAACTCAAgcttgcaaaaatattttatatctatatataatatataaatatatacagtatatatataagTGTGCATCTGACTATCCTTAAGGCACCAAAGAGATGTAGGCCATGCCTTTCACGATTCAGTCtttgttttcctagaaaaatgtctcttcctctctcaagtgaaaaatatttcttctatcaAATAGCCCGTGACAGTAAATCCAGAAACAACAAATATCTTTAACTTAAGATGGCTAATTGTTTGGATAGGGGGGAAgggttggtggtgatggtgatattATCCTCTATAGCTTACAGCTTAAATCTAGAGCTTAGATCTAGAGCTTAGAGCTTAGAATTAGCCAGACACAGACAATGACACTGGATCAGACAGGCCAGCCACCAGATGTTGTACACAATTGTTTCCCAGTAGCTGATGGGAAAACCTTACCATGACCAACAGAGATGGAGGCAATGAGAAAGGGAATTATGTCTGGCACAGATTTTTCAGAAAGACTTTGGCTTTTCAGAAGTTCCTAGAGCTTCTATACAGAGAGCTGGGAAGAAGGCTAAGGTAAGGCACTGAAGTTCAGACAGGTCAATGAGGAGAATATAGGCAATTATTCTGGTTTCTTTGGCTGCTGTGGATTCTTTTAAAACCCATGTAATCATGGGGGTGGGTGGGCCGAACTTAACTGAAGAAGCTCAGAGTATATTAAGCAAAGCATATgaataggaagaaaaacaaaattaacacacacagaaacaagtGGTGGTGGTatcccagagggaggaggaacatTTGTTAactaattttcattttgctcctctcctgcttttctctgccaTGGTCCCAATAGtttagttctgttttttgtttgtttgtttttatttttatttttccagcagatgaagaaatgacAGAGCAGGATAGGTGTCTTAtggtttttgctttcttcttaggcctgtttaattatttttttcctcagttgaCTGTTGGCACCTGGTTCCTCTGTAAACTATATTCCTTGGCCTTCAGTCTCAGGTTGGCAATGCTGTTGGCCATGTTGATGCCCTGTGCAGGGCTATTGTTGGCACATGTGGCAGAATAAGTAGCCATGGCGCTGTAGGGAcaaaggagagagcagggagaaTAAAAGGCATTAGTTTCACAAAGCTGCAAGTCTGTGCCCCAGATGGCTGCAACTCTCAggatgggggcaggtgggggcaggtcGGGGCAGGTGGGTTGGACTAAATGACCTCTGAGGTCCCTTACAGCCCCAGATTTCCATTAGTCTACGGCACTTGGAAAAGGGTGGCAGTGTTTTGAAGGGCAGGGAAAAGCAGTGGAGAGTGGAACAAGGCCAGGGAAGAAGTACTTTCTGTGTCGGATATAGTGAGTAATGATTTATTTCGGGGGTGAGAGAAGAAAGTTGCTTATGGTGGATAGCAGGTGTTTCATCAGCTGATcaattaaaacagtatttaatcTCATCCTGCCTACGTCACCAGCTTGTGGGAGCAAAGAGGAGAAGAGGGTatctgaaaagaaactaaagGGCATTTTGCCTTCCTCTCTCTAGGTAGCTGACTTTAATTCTAACACATTTTAGATAGTCAATAAATACCATATTAAATAGTGTACTTGGAGAACCAGTTAAACAGCGTAAGATGACTTTTCTACCTTTCTTGATCCACTGGTTGATAGTAGCAAGCATTGCCAAACCAAGAGTTTCCATCCTGATTGGCCTTGATTTTATAGCTTTTCTTCAAATGAACTTGAGTAGCTCACACTGTCTTTTGGGTcagttattttggaaatgtttaaatCTCCTAGATAAACTAGAGAAGGCTCCAGTTTCCGTGTTTCCGACCATTCTTATTGTTGAGAAAAACCCAGACTTGTTTTTCATTGTCAGAAATTGTAATTAAGACCTGGGACTACCACTGGGGAGAAATTGTAATTGGTGGTTTTCTGTTAATTGCATCCAtgcatttcttttaaaggaaaagtaatgGTTTCCTATACTTGGATGCAAATTATCCATTACTTTATACTAAACAGCTAAAAGGGAACTCATTTTAAtctaaatgaaaatgtttctagTCATTTCTAAGATTAGCAAAATACCTTTCAACTGAGGGCTGGAATGAGATAGActtataaatggaaagacatgAACTCTTCTGGTGCCTGCATTCCTGTGCAATTTTGTCAGCCACACAGGAGTTTGTGGTTCTGGGGATCAAGTGacagaccagaaaaagaaaaaaactaaagctaCTCTACAAATAGAGAGACTAAATCATTAATACTTATTTGGGACACCACCTAGTCAAGATTTACAGTCAAGACTCACAATAGCCTATTTCAGCTCAATTACTAGCAATTAACCCAGGAAAATTGGGTTAAAGAAATGTAATTAGTCCCACATCACTAGGTCTTTCCAGAAATACAAATCCTTAATTTAAAATCCGTACAACACCAGTatatttgtgagtgtgtgtgttatGGAAGGAAAAAGACTCAGAGTGTTCTAAAAGGATCCTatactcttttctgtttcatttttgtgtgcTTCACGCTTACGCCCTTACTCATTTCTGGATACGTGATCTTAATCGTCTGCACAAAGTTAGGTTCACCTATAGCACCCTGTACTTTTCCTTTTAGCTCTTATTTTAATTCCTCGGCTATAAACTCCATAGATACCATATCTGCCTTGTTCACCGCTGTGTCCCCAGGAAGTGCTAACACCATATCTAGCCCATATTCAGCGTCCAATAAAGTATTTAATGAATAGATAACAAACTGAGAAATATCTAGATTTAGTATGATTAAACCGATGGCTTTTCAGTATCAAAGATCTCTGTCATCCTGCTGTGAGTAatgatttacaaaaatattttaccaatatgcttttattttaaggaacCAGTTCTTCCCATTAATTCTTCTATCTTAATAGAATGAGTAGAATCAGAAGAGACATGACAAAAGCAGCGGTTGATGATTGCATATTCTGAATCCATCACAgcttttaacaattaaaaaaagaacaaatattgatGCAGTTTAGATTTACCGAATACACTATTACTTCTACCACTGTCTAtcaaaagacaaaagcagaaaaagaatccCCAGTTCAGGTGAGTTTCAATTAGGAGTGAGATAAATAATCAGACTAGCCTGTTAAAACaggacaagttttttttttttaacaatatatcAAGAAGTCTCACTTATTTTCAAAAACCATGGATGTATTATCATTTATGTTGTATATGAATTACATGCTTCTGTTCAAGATGCTACTAGAAATACATGGAAATCCATAAATCTCATACTCAATAAATTGCTAGGATATACTGAAATCTAAGATTTCAAAGAAAGCAAAGTTCTAAGTAGCTAAGAAAAGAGACAACTGGATTTGGGAAATGTACCTAAGTTATAGATCATGAAATCAACCAAACCAGCCTTTCTCAGCAAGGTTTCTGTGAGAAAATTAAGCCCTAAGGAATTAAGCCCTAAAATGGTACCATTTTAAATGCATAGAACAGAAGCTGGAAATTTGAATTATCTGTATCATTAGTTAAGTACTGAGAATAAAGTTGAATAGCTTCTGAAAATAGGAGGCTAAAACTTCAtgagagagtgaaaagaaaaaaaattctctgatggCAAACTATAGATTGTCATGGAGTATTGTGCTTTTAAGTATTTGGCCCAATTTTTTGAGCGTTATGTTTTCCATCCTTTGAGATTCTGGACAAGCCAAGTCCTGTTTAATAgagattctctctttctctctctttttttttttggatgatcAAAGTGTGGTCCATTTTCCTTAGCAGACAAAGGGAATTAGAACCTTGGGGAATTATTTTAGTTTACTGAAGGTACTGTTAACTGTTTTCTAGAAATGTCTACTATAGTTGTGATTCCATGGTTATTATATAACCATTTTTTGCTGTAGTTACTTCATAGTTTACTAGTATCATCTGGTTTTCTAGTCTGAAGACTCAGGATGGCAACACATTTCTTGGAAATACTGAGATTCTCTAAGAAGGCCAAATGTTAACATCCCAAATGAGATGAATATTGATCCATTTCCCATAGCAaaaccaaattaataaaattagagCTCTATTTTATACAGAAGATTGGATTTTCACAGAGACATTTTTCCTTAAACGGCAGCCTGTGTTTGCAGAGTTGAGAACAGCATCCTTGTCTAATTGTACTGTGGTGCTGTTAGAAAGACACCACTGTGTTAGTGAGACCATGACAATGCCTTACAGGTAGCTCTCAAGTTAGAGGGGTGTGAGAATAAAAAAAGCAGTTGATGTTAAAGAGTGAGTTTTGCAGTTCCCTAGACACTTGATAAGTGATCATGTtttcactcagtaaatgtttggaaaagttataaactttcaaaaattggtctgacttatttcagCATTGCAGAGCCACAAGAAATAGGATAAAAGCACAGAGACGGCCTTAATTCACAGCCTCAAAACACACATGTAGAGTACATAACTCTGTAGGTAGAGATTACAGTGCTACTTTGTGCACATACTAAACAAAACaagtctcctttccctcctgtaGTTTGACATGAGTTTAGTTTCTCAACTAAAAGTGCCTCTTAGGCAAGTAGGGACCAGTCCTCTGACATACCAGTGACAAGAGAGACATGTTATGCATCAGAAAAGACAGAATTAATCTAAGAGCAGCTTAGTCATGCTTTAAAAAGGCATGAGAAGTAAAGTGGAGAGGACCCACTTCCAAAAGTGACAACAGATGCAAGAGAGACTTGAGTGGCTGCCAGTTTGAAAGATTGTTCATGACCACCTGTTTTCCATGTCATCAAAGCTGTTATGGCTCTCtgttaggaaaagaaagagaatcctgagatttttttcacaCAAGACAGAAACCAATGAGGTTAGTAAGCTGTTTTCAACCACTTTTATAGGGCCATTCAAACCATGTAAAATGACCACTCTTCCCAAACAGGATTAAGCTTCCTGGGATAAAAGGGTGAAGTTGAAACTAAGTAAGTTGTACAGAGTAGCACATGAAAACACCAAGAGCCTGCTTACCAACACCCAGAGACCCCGCCCATTGGTCTTGCCTTCTGAGTTAGAACCACCCATCCCTCTAACAGGAGGCAGACGTGAGATGAGCACAGCGACTGGGCTTACAGGGTTTGGCCACAGCAacatttctatttcacttttaagAATGTAAAGTTCTTTGTGTCCATGACCAAGAAACTGCATTGCTAAATAAAGGGCATTAAGGAAttagagataaaaaagaaatgcttgtaTCCCATCTGTTGATTCAGCAGATCTGCTCTGGGACTTGCACAGAGCTGACACATTTAAGTAAGCTTGAAATTTTTCCATCTCCTTATTTGGAGATCCCTAACAGATGGAGTTCAGACATACACAGCAGAATGCTAACCACTGGCAGGGTTAGAGTTCTGTTTTCACTGGGGACAAATCATTTTTGATAATTTCAACCTACCTTGAAAGGATGTCGTGAGGAAGAATGTGTTAAAATGACTGCCAAACACCAAGGAAGTATGGAAAATACTATACATGGGGTGGTTCTAAAGCCAAGAAACGCATGAACATCTCCCCCTCGGCTTTCTAATCACCCAGAGTGCAAATCCCACAAATCACAAGATAATTGAGGAGAGCTAGGGGGAAAATAATACCTGACTCAGTTTTCAActctaactttgttttctatttggcCCTTTTGGACAAATGATGACAAGCTGTTGAAAATTTCACCCCAAAGACAGATCTGTCAGCCCAAGAAGTCTGTGTCTTCCTGAGACCCCAGGGCTTCCCAGCTCGGTATTTTTCCAAGACTGCAGAGCTTGAGGTTTTCATGACCCTGTCCGTGATGTGTAGAGGTTGGCGATCATCTTTGGTTTGTTCTTTTGTCAGTGCAAGAACACTTTAGGTGTGCTTGCTTCATGTAAGAAAATTCAATATGAGTTTTCCTAGCgaaatttacttttaaacaaaGACCTGATAAATCCTTTTAGAAGTGATCTTTTgtgatttaaataattattttcaacttctatttttaagattcagaagttgattttttaaaggtatatgtAGCTCTGAAAGTGGACACATTTGGACCATATTTCATCAGAGAGCCTTCAGTTTGCACTGCACGTTCAGTGATCCCTCTGCCATCCTGAACTTTTCTTCCTAGAGATGTTTTATGTATGAAGCCTTGCATCTTTCTGTTGTCTtaatgatttttgtatttcttttttgaattgcACATTGCTTAACCATTGGTTCCAAATACCCCTGGTGAGATATCAAATAGGGAAACTATCACTAGTTCAATCTTAGATACAAACCTACATAAGAGACTGATGACCCAACTGGCCTGGGGAGCAACAAGAGCCAGGACAAGAACATCGTTCTTCTGACCTCTGTCGCATATCCTACAGTCATCTAAAGCCTCACGATTCAAAGTATGTGCCAAGTGTCAACAGCATGGGCATCACATGGGAGCATGTTAGAGGCGCAGATCCCAGGTCCTgccccagacttactgaatctgAGTCTGTGTTGTAACAGAATCCTGGTGATTCATATGCCCGGTAATCTTTGAAGAGCCCTGCTTTAGGCTATCAGACTACCCTTAACTTTAAGGACATATTTTCTTGTAGCTCTGGTTTCCAATAGTATTCCCCACTTGAGGACCTCACTACTTTCATTTCTACTTTCTTGAACACACTTAATTCCCACTTAAACCCTACCCCCAGcagccaccctcttccccctctgacAAGTTACCTTTTCAGTGTCTTCCGTTAGAATCCGTTATGAAGCCCCTCGTGTAAACAACATCTTGGGAGGGACGAGGATCTATTTTTGAAAGTGTGAAAGGGAGAAATGAGCCAAATGCGTTCAGCAGATGTCAGGCAGCACCGTGACCACGATCAAatgaggggaggagaaggaggagaaggttgggggagggagattGTGAATAAGGATCGCTGGCCTCTCTTTCTTTTGTCATTAGTTTTCTTTgaagcccagccctccctctggaAATGGCACTCCAGACCAGTACTGCAGAGCCTCGTCATTCTTTATCTTTCATAGGAAAGGATGTCTCTGTGGAAAGATTTCTTGACTACTTCATCTAAAACAACAGTCAAAAGTCCAGAACTTGGTTAATCACTGCTACCCAAGAGGAAAAGCACTACAAGGGGAGAGCTGTGGAAGTCAGTAAACTGTCCTCTCTGGCAAAACTGTCTGCTCAATGAACATGGATTGCATGACCAAGCCACATCACTCATCCCGGGGGGCAGCCCACACACCCCAGAACATGGACCTCAGGCACAGGCGTGCTTCACGGATCTGAGACAGACCCTGAATCAAACAGGCATGTTTACGGAGTTTCCCAGTCTTACAAACTTTTCGCTATTTTCAATATCATGTAAAGTTTAATTTGAATGAAAACATAGTTATGTGGCCTAACATGTTGGACAGAGACAGGGATCGTGAGCTCTGGCTCTAATTCTGCTTACTGAAGGCTTAAACTTGGGCAAGTGTTCAGTCTCTTTGGAACCAGATCAAGGACAGAGCTGCCACACAGGAGCATTAACCAAGGATTAACCAAGAGAAATGCCTTTTAATGAATGTCACATGTGAGATAACTAACTTGACATAATGGTACCAACAGAAATAGCGAGTTACTTTTCCATAACACAAAAGGTCTACCGTTTCCTCGGCTTTTCaccacttgtttttcttttatagttccTTAACAAGACTTGTATTTCCTACCGAGcttcaaaatatgaaatgataaCATATTTTGGTTCTAAACATAGCAGAAAACTCTCCAAGTAACAGAATAGCTTTTTGCCACTTtgagaaattaatcaaaatataaacaCTTCAGTCAACATTGGGTCAAAAACATTATTATTGATTAAATTACTGCTTAAACCACCATTTTTCTACTTAAAGCTGGAGATTAATTTATTCTTCATACAGAATATGAACAATCAGATTCTATgaacttttaattaattattttttacaaactCTGTGTCTAAGAgagaaatgatataaaaatataccataaaatgAGTAAGAAGTACTAGGTTATATGATTTTAAGTGAAAAGAGCAGAATACAAAATAGTATATACACTAAATCTGTACTATAAAAAATACTGAACTCtaattgcaaaaataaacaaactaattaTCTCAAGATGGTGGAATTatgaatgtttttgaaaaatgtatctaGTATTTCTACAtaatctttcaaattaaaatagaacacTTCCCCAAAAAGCACACATAAATTGTATGTTAATCGTTTCAGGTAGATGCCCATATACAAAGGATAAATGATGcaggcactggggcttgaactTGCGCTGAGGTCCAGGGAATCTAGCCAGTACTGATTCAGGAAGGATTTCGTCCCTGCTCATGCAGTGTGTTGCCCTCCGCCTCTCATCGTTAAGAACCACATGACAGCTATCTGGGTTATTAGCGCTGGTACGGAAAGCTCCTCACTAGGGCCCAGGAAGAAAAGTAAGCAAAACTTGCTGATTGTGACCAAATTCAAATCAGAGGCCTTGAAACATCAAGCTCCGAAGCACATCCATTTCCCAAGCCATTCAATGAGTCTGTGAACTATCCATATGTATCAGAGAAACAAAGCAATCttccatgtatttaaaaatttaaagattacgTGATTTCACAGTGgtcttcaaaatgtgtttttagtaAATTCTATAGAAGAGGAGTCTAAATTTCAAATAGGACATACAGATTGACTAATCTTTTTGAAATCAGCAAGGCATATTCTATCTGAGAGCAATAGAAATTTCCAATTTCCTTTGCATAGGGTTTCCATGTACAAAGACCAAAGTAATTCAGGCAAATTTTACAataggggattaaaaaaaaaaagtttccaaatgcATTTATGGTGTTTCTTTCAAGGAACTTATTTgaaacagaaagaatatttaGGTGAGGTTTCTTGGCTTTGATTAGAGCAATCAACTTTTGGAAAGTATTTGCATTTCACAAATGTTGTGTTTCATAGCAGGAGTTCATCCCCAGCTGAGAGATAATCTCCTATAATCAGTTTAAAACCTCTCTTTAAACCCTTAATTAACCTTAACAATAAAAAGGTAAACAGAAGCATAATAAATGAATTTGTGAATACTTCGCCTTCCACTGCAGTGAGCCAGAGAGTTGAACCTGGATCTGGTTTCTAAGAGTACTCAGGCTGAGGTTTAAAGAATGACACTTAAGGCTGATGACATTGCCCTAGGCACGTGTGTCTCAGCTTCCCTCTGGCATTAATCTATGTGTTCTCAACAGCATAATAAATGGAGGTTAAAAGCCTTtccaagtgctttttttttttttttgaaatagtcttctatttgcttttttagtatttttcaccttttaaatataaaaatttagtgtaaattatactttgtttttatatatactttttaaaaaacacacacaaactaacTCCCAAAAAACTTCAGATAAGTAGGTAAGAATTAGCAACAGAATAGGAAGCATCTTGGATCATAAGACATCCCCACCTACTAACAAAGAATTGTGGCCAGGAACAGGATCTGAGTgaccttggatttttttttttttcttctcaatctctctctgtctctctcctttttttccttccttccttctttccttttcattcaaaatcagttttactgTGAAGTTTGCCTTTGGTCATACACTTAGAACCTTGTGTGTTAGATTCCTACTTAagtataataaaaacaaatctatctTAAATTTAGAAATGGGGTAAAATTTTGCTCCAGAATGATTGCAAGAGGGACTCTGGGCAGAGCTTAATGATCTTTCCTTTTAGATTCTTCCCCATCTTTGAAACCTTTGCAAGAGTTCCTGAGTCCAAGAGGAGATGGCTGATGGCCTGTCCCTCTCTCTTCTGCCCCACACCCTGCTCCTGGGGCAACTAGCAATCTCCTGTGAAAATCTGGGGATGTCAGCAAGTTGAATGTCTCTGAAGAGGGTAACCCAGTGAGAAATGTCAGCCAGCTGCACAAGCAGCTTTGAACATGACCGACCGCGGAGAAACGTGCGGAAGTGGAGCAAGGGAGAGTGGGCCAGTCATTCACCTGTACGGAGAGGCCGTGCCCCAGGAGAGATAGTCGGTGGGTCTTGGAGCAGGACGAGGTACGATGGGCTGCTCCACAGCAGTCACGTCTCCTGAGTACGATTTGAGGAGGGAAGCGTTTTTATTGGCCAGCATGGCTCTCTCATTCCTGCGGAATTTGGCTCTTCGGTTCTGAAACCACACCTGGAAGATCGTCGGGAGGAGAGAAGGGCATCAGGAGCGagaggagaaacaaagaagaggTTTCATGAATCAAGTCCTGGTTGATTTTCCTATATCATGCCTTCCCGAGGGAAATATAGGCTAAGTATGGAATTCTTTCTAGAACCGTAGATATATGCCGTAATCTTTTCCTAGTGGGCCGTGTGAGAGACCTGTTAAGGACAAAGACTGTTATGACAAGATCATGAGGTACTTCCCCACATCAAGCCACCGGAGATGAGCTCCATGGCTAACTCACGTGGTAGCAAAATATGGATTATAATCCCTGCTGGAATTGtaatttaaagaagaagaagaagaagaaaaatctcactaGGTTTCTGCCACAGATACaatgacattaattttttttcgAAATTCTTCAAAATGCCAAAACTTCatctgttttcaaaatacttattaGATATTTAAGGAAAATTCTCCATCTTCCCCAAAGTAGACCCTGTGATAAGGATTTAAGAAGCAAatagtttatttgggaagtgCAAGGAACACCAGTAGGAGAGTGAGGAAGTAGCAACGGAAGAGCAGCCTCTAACTTGTGTGCTGCTCAGCCAGCTCCCTGGTAGCCCAGTCCTGCAGGGACTCAAAGGACGAATGCAGGACACACACCTCCCACCCGAGGGGGAGAGCGCTATGGATTTGTATGCTGGCTTCTGTCAGTCATTGGTTATTAGTGTTCCAGCACTACTGGCATGTCTCCTGAAGTTCTAGAAAGAAATTTGTGAAACGGAAATGCAGATACTGAAGACTGGAAGTTGACTggagtatgtttaattttaggGTTCCAAGGAGATAGGTGGTGCTGACATCACTGGCTACAGCTCATGATAAACctaccatttattaagtgctGTTAGGGCTAGGAACTTATATGAGCACATTCCATACTTTGTTGCTAGTTCTCAAGACAATTCTACAGTTagattttaatgtttctatttcaAAGTTGAGGAAACAGTTTCAGTGAGATTAAGTAATCTGTTGACGttcacacaaataataaatggctGTTTCTGAGCTCAAGGACATCTGACTCATGATCATACAACTCATCCAGACTGCTGtggaattttattcattcatttactcattcaagaAATCTTTATGGAGTGCCTGCTGTGTTCCGGACCCTGGCTTAAATACTAGGAAGGGAGAAATGAGACATTCTCGGTTTGTAAGCAGCTCACCATCTATTAAAAGAGGACAGacatatacatttgaaaaatggcaAAACAGGAATTAGGTGCAACTATGGGAAAGCATGGCAAGGTGTGGGGGAAAGAAACCCTATGGTGTGGAATGGGACAACTTCACAGCTCTAGAGGGCAACATTCACATAAGATAACCATGGAAAGTATGtggaaggcaggccagggtggaAATCAGTAGAGGCAGAACCTTGCCTGGCTTCACAGACTCTCAGCTCTTTTGTCTCTAACGTGTCTTCCACATTGCTATTAAGgttatcattttaaatgcaaatataattgtATTAGTGCTTCGCTTTACATCATCACTGACTCTCCATCACTCACAGAATTAAATCAAAACGTCTTACCATAGAACACAGACTGTTCAAGAATTAGCTCATTGTCCACCAATCCAGCCACATCCTGTGTCTCTTGGACTTCATGCACTGGCCCTTTATTATTCCAAATCCTACCCTAGGCCCAAGACTCCCAAAATGTATTCAGTTATGTTCCCCCTATGTATATTCACGCCGAGTGGTCTCCCTGCCTGTGATAGTCTCCCACTCTGTCCAGGTGTCAAAGACATGTTTTCTCAAGGTTTAACTTTAACATCACCTTCTTTGTGAAATGTTGGAGTGGAAAGAACATGACTTTAACAGAGTTGGATAAAAATACTGGCTCCACGGTTTTAAGCATTTGCCCTTGGgtagttatttaatttctctgatccTTTGTCTCCCAgtcaaatgaagataataatacctgcttatagggttgttgtgagagtTTCACTGAATGGCTGCTTAATGCGTACGTATTCTCCCCTTTTTCCCTGGACAGAATTTATCATGCCTTCTTCTGGGCTGTCACTAATTCTCGAACATTCCCTTAGTGTAGGAATCATTTTACTCTATATTAACAGTAGGATGTGCCCATCTGTCTTCTTTGCCCTTCAGAAGAGGGCCAGTGTCTTACTCATCCCCAGCAAAGCAAAAACAGCTCCAGGAGAATAACAGATGCTCGATAAATGTTTGAGCGgaaaacaggagaggagagaaaaaaaaagaatttcacaaTGATGATAAGATATGGCTTCCATCCAGGAGAACTTCACAGTCTAGTTGGGGAAGTTGGCCCACATACAGCTCAATATAATACATGTCATGTTGTGATAAATGCCATAAATATAAAGAAGGCCAGAGTCAGGGTGAGGggatagctcagtagtagagtgcacgcttagcatgcacgagatcctgggttcaacccctagtacctccattaaaaattttcttttaaataaagaagtcCAGAGTCAGAGGGAGTGGTTCAGACTGGGAAAGGGTGAGACTTCACGAAGGGAGAAGAAAGGTCATGGAGGGAGAATTCTTTCCTGCTCACATAGGAAATGCTCTTAACTCTAAAAGACTTTAGAACATAGCAGGATGGAAAAGGTGGACGTTAATAGTTTCAACAGATAGGAAAGTCATCTGCTTATTGTTGTGATGGGGCTGAAATAGCTCCCCAACCCAAAATTGCTCTtccacatcttaaaaaaaaaattccagcatgGCCCTGTCCAGGtttctttatttcaaattcaGATCGCCATGGCAAAAGTGGTACTTTCAGGGACTGTGGGTTATTGgcctaaaattatttattataccACTGCAGGCCAAGCCTGAGACATAAAAAATATGGGGGTtttgaaggagagaggaagggatttAAGAGTAGAAACAGAGTGTGTTTGGAGAGCTGTGGTAAGCAGCACGGGGGAAGAGGATGTGGGAAGGCAGACCTCAGTGTTCTGCTTTGGACGGCAGAGCGGAGAGGGCACGACTGAGCTTTGTGCCTGTGGACCAATGTCACCTGAGCCCTGAGGGACTGGAGAGCAGTGATGTACCACGTAACGTACACATCGTCGCGTACGCCTTCTCCTAACCTATCCTTGGAGGCACCACGGTGCCCAGGCAACTATCCCCTTTTGtcactgacatttttttcctccttcctgaccTCTTCTGAATGAGGACCCTGTTTCCCAGGGTGAAAACCCTCACACACCTTCTTTTCAGGCTAAAGAGACCTCGAGGACCCTGTCCCTCAAATTAACAAATGTCTCTCACAGTACAGGGTGTTGGATACAGTTCCTACCTTCCCAGCCCTCTGAGTCTAGCTCAAGCCCCCATCAAGAGAGTTACTATCTTCAGAAAGAAATCCTTCAAGCCCAGACTGAACACAGGTATACAAACAATAATTTCGTGAGGAAGACAAGACAGATTTGACCCACCTCGCTTAGCGAAACAGCAGCCTACCGCCGGCAATATGGAAGATAAATGAATCTTGACTCTCAGCAGAGACAGCAAAGCACTACTTGAAAAAAGCATTTCGCCAGAAGACTCATTTATTTAAAGTCTACTATCGTGCTAGAAAGCACGAACTCTGGATCGGCATAGCCATCTCCAAATCTTAGTTCAGCCACTTCCTAGCTATGTGACGTTGCATGAAGCACTttgacctctgtgtctcagtttcttcccctgAAGATGAGTAATACTGTCCACCTCGTTGAATTGGTGTGCGGATTAGACAGATTAGTTTATGTAACTAAGAACACTCATTGGTGAACAAGGACCCAGCTAGTGTTACTGCATTGTTATCGTCACTGTTATTCCTCATCCCAGACGGCACTGTTTCTAGCTGTCTGACTTGGATTTGCTGAGTCATGGCATTCATGTTAGAGAGAACTGCTGTCCTCTCTTTACCCTGTCTTACGTGTCCCTACCCTTCTTCGCTTCGCAAAATCATCTACTCGAACGTGCTCTTTACGCAGCTCCTAAGTGGAAGGTCTTTGATGCATTAATTTCCCACTCCCATCCGTGtccatgcctgtgtgtgtgtatactatgTTTCTTGAAGATTTGTACAGTCCCAGAGTCGGGGAGGAAACCTGAGGAGGGGGCCGGAGAGTCCATCCCTTTGGCCACCCTGCAGCTAAAGTTGAAAGCTCGTGGGAGAGGGGTGCAGAGTCACCTGCTCAGAGGGACAATGTTTGGAACACGAATCTGGAAGGGTGGGAAGCCCTGGGGAGGGTGAGACCTGCAGCAAGGCACATTCTGCCCTACTTAAAGGCgttcactttgatttttaaaaataccgtGCTGCCAAGAGAAACACAGTTAGAAACGGATTCTGTTCTCAAGCTGCCCATTTATAATCCTTATTAGTACCTGCTCCAGTCTGAAACCTACAACTTGTGTTTTTTAACAGTCTTCAGAGATGTGTCTCTGAGGAGGGGAATACCCTGGTTACCTGCACTCTGGCCTCAGTGAGGTTCACCCGGCGGGCCAGGTCTTCTCGCACAAAAGCATCTGGGTAGTGTGTCCTCTCAAAGACACGCTCCAAAGCCTGCAGCTGGCTGCTGTTGAAGGTTGTCCTGTTCCTTcgctgctttctcttcttcttttcctctgagttcAGTTGGTCATCTGGAATAAAAGAAATTGATGATAAGGAGCTAAATTCACGTTGGAGGTCCCAGATTCTACCATGAGATGGTTTCATTTGCTCATATGCTCGTCAAAGTCTGTGGCTAAATGCCACTTTCACTGCGCAGATGTGGGCACTGGTGCTATCGCGTCTGTGTGCCTAGAACCCCCGATGGTTCTGATGCTTCCTTGTTGCTTCTAAATCTGGGACCGGACCTGAACAATTAGAAAGACATTTCTTGCATGCTCCTTGAAGTTTGTTTTGTCAAAGAAGGCTAAAGGAAAATGTTACTCCTCCCCATATTCTCCGGACTTCTCCAGAGATGGGGGGAGAAAGGCAGACTGCTTTGGACCTCATTTATTCCTACGCTGTCATAACCAAGAGGACCGACTATTCCCTGGTTACTTCACCTCAGCATAGCCATGTAACACAAATACAGCTACAGAGGAAAGACCACGTTCACGCCGACTAGAGGGATGTCCGCTTTGCCATGCATTTCCTGGAAACAGCAAGTAATTGCTAACTCTGTTTGCTCAAGGCCTCATGCTGTTGCCGCAGGCTTCAGGGGCCTCGGCCAAGATCGGGGCTAGTCCTTCAACACCATCAGCCCAGCTGACACTGGCCTGGGACTCACCCTGTGAGAATTCAGGAAGCAGGAAACAGGCCTGAAAGATCTCTGAAATGGTTTCATGCAAAACTGGAGTTGTAGGGCCCTGAGGTCTTAGGAACCTTGGAGGAATCATGACCCCAAGTTTCTACCTGTCAAGATTCCTCCCTGGTTTTCTCTGAGTTCCCCCAAAGGCACCCTGGCGAGCTGATCCCCTAGCCTGCCTGCAGCAGTCAGGGAGTGGCGTGCTCCTGCCCTGCCGCCCCCACTCAGCCCAGGCGGGGATGAGGGCGTGTGAAGCTGAGCGGAAGACCAGCtgtgccatcaccaccaccaagcTGTCCCCCTGTAGCCCAGCCACCGCAGAGCCCGTGCAGCGCTCAGAGATGTGCGGCTTGAGCTGCTGAGCCCAAGTCACACGCTCGGCTTGATGAACTGGACGAGCACTGCCAATCACTCTCCCCTGACGAGGCTCAACGCAAAGGGAAACACGAGGAGTGagactgaggctggagagaaagaagagcgTGGCACTTGCCCCACGTTCAGTGGGAAGTAGTTCTCAAACGTTTTTTCCAACACGGAAAGCCCCCTGCTCCTCTCTGACTGACACAAAACAGTCAAGAAGACTGCTAGCAGGAATCCTGAGGTCCCAGGGAGAGTGGCGCCCACCTGAGCTGCAGTATTATTATTGCAAATTGAGGGCATGTTCTAGATTTGGGTGAAGATTCAAAGTGTAGCTTAAATCCAGGGGTTCCCGGCCCACATCTCTCAGCACCTAACAAGAGGCTCCAAGTCGTTCTGATGAGAAACCCTGTCTTCCTGAGTGCCCTGGGAGCTCGGAATACAGACGGCTGCCTCCCTCCTGACATTTAAAGCACCCAGTAAGTGCTTGTTGAATTTGTTCCCGGGGAAAATGTGAAACCTTTCTTGTCTTTAGAAAACTGTTTCTGTAGAATAAAACCCATAAAAACTCGACCGCATTCCTCTAACTGAGAAGCTGCTGGCCCCCATTCTCTCAGCGTTATGATGGCCCATCTCTCTGGTTTTTTCCATCTGCGGCACCTCACTGAGGAGCCCTGCTTTGAAGGCACTGCTGTGACACTCTCAATAAGAATGTCAGGTCAGCGGCATCTTGAGAATGCAGCCAAATTGCATTATCGTGATTTCTGGGATCTATCAAAGCCTGGAATGTTTCAACCAACATCCCTGATTTTACCATCCCTGATTTAAAAGCAATTAGGAAAATTAGTTCACAATCATGTCAACAGTGGAATTCAGACACCTAGGATCACACATCCTTCAGCTGTTGCCTTGCATCTGCATTCTCCTTCCTATTTGAATGTTTGCAGGCAGTTCCCATTGCTCCCATTCCCTTTCTGCAGTTCTGTATGGCTTGCACTTCAGGCCTCTCTAGGTCACTCAGGCCCTTCTGACAGCACCACACCTGCATTGCATGCTCCCATTTAACACAGGTCTGCAAAGTCTTTAGCTTCCATCCCTACCCAAGAGTGAGACCGATGAGACAGGCCCTAGGCTGTTTTAATCATATAAAGGAAATCTCTTACTAAGCCAATCCCatgatttgctttctttcctcaaagaaaGTGCTTTCTCAGGTATAACCACAAATTCTAAATCCTTTATGCTGTTTGGGAAGCCTGTTTCTTCTTGTCCTGCCAGACTGTGATGGAGAACAAGAATATTTTATGTCTTGTTCTTTGTACTACAACCTGCTGCAGAATCTCTCTCGCTAGTTCTCTGAAATCTTCCACTGTTCACTCAAATTCCACTGACTGTCCACCATAAACCACCAGCTCCCCTGACGTGGGAGTAAGGGAAATGGAGAATTATAGTTTATGACAGAGAATAAATAGCTATGATGACCTGAAATGATTCCAGTGAAAGTTTCCCAGCAAatgttcttgttgtttttaacaaatgaaaacaccAGTCCAGATCCTGCTGAAGGTGAGAAAAGACACTCAAATAACAGTGGTCCAAGGAGTCTGGGTCTGGCAAGTGTCCTGTGGAGCGCGAGTGTAATAGGAAATGACGGCGAGGGGGCAACAGAGACAGCCTGAGCGGCAGCCCTCGGCTCCTCTGTCTTTGCTCACTAATCACCACCTGTGAGCTTCCTGTTTTCCCCCCGGAACACAGAAGACTAGGACCCAGCACAGTCTCACCAGCAGCACGAGAGGAAACCTGAACGTCGTAGAGGTTTACAAACATGGGGCATCTGAAATACCTGGGATAAGGACTTTTATCAGATTCTGGACTCTGGTTGAATATGGGGGAGAGAACACAGCGATGTTATATCATTGGTGAAAGCTCGTCTTTCTTGTAAAAATAGCTACTTGCAAAGGTGGATTTTGAAAGATGCTGTCCAAGGATACTGATGTCTGATGGGCATCAGAAAAGAGCTAATTCTTTGGAATTCTGAAATGGGTAGGATTCACCAAACTGCAGAATAACCACCAGGTACATGGATGCATTTTTATCAGAAAGATGTTTCAAATCCAGAGCGTTTACAGATTTTTACGGACGACTATGTACGTTGTGGACAACTGAGTAAATTGCAAGTATACAGTGATTGCCATGCTTTctaataaaaacaagagaaatgacTCTATTAACAGGTGAATTTTCACTGGGATTCATGAGTCATAAAATACTGGCAGCGCATGAAGCCAGAGAAACTGTCTAAGTTAATCCTCTTATTTTACATGCGGTAAATGAGGCCTCGAAGAATGAAGGGATTTGCCTCAAATCACAATACAAGCTGTGCCTGAGCCAGAGCTAGAAGCCAGGACTTCTAATTCCACTCCACTTTTAAGTGCAAATGACAGTACACATGCTGTGCATGAGAGGGACGAGGATGCAGCAGGGATATACATAAAAACCCACCACCTTGGGAGAGGTTTTCATGGCGAAGACAGGTTTACCCACACCAGCCATACAAAGAGGGTCTCGGGATGACGGTCCACGTGTATCACTGGTATCAGAGGAGTATCATGGGTCGTTACTCCTGCCAGAAGAGCCGTTAAAAAATGCAGCCATGCTCATCCAGGAAAGCTACTTTGATCTGGACGTTCTTGAATTTGCCTTTTCAGCGGCCTCTGTAAGTCTCTGTGTCATCAATTAAATATGTACAAGGTTGCCTTAGCACAGTAGTGAACCCCCGGCTTCCATCTTCTCCCCACATTCATATTAGGACTTCTCTTCATTTCAAGACACAGTTCAAAACCTGTTTCCTATGACATGTCTCAATCCTACTTTCTCTAGAAAACTTTGCTGACAACACCACTCCATACGGATCTCTCCATACCTAAAGTCCCATACATATTTTAGTATAGaatcaaatacttttttaaaattatggttcaATTCTTTCTGTGTAAATATCCTGCCACCTCTGTGCACTcagtatttctataaaaaaagaCATCGGGTCTTTTGATGAATTAAATGACAGTAAACTTTGAAAGAGTTCTATAGTGTTGTGGTATAGAAGCAGTCCCAGAAAATTAGAACCTTGGTGATTTACATGAAGTTTTCAAGGGCTGAATAATTCCCGAATtccaattattcttttttaacagtGTATAGCAAACAGTTAGGTTAACATTATCTTTTGTCCCTGCATTATTTTAcctgtggaggaaaaaaatccttttaccCTGTTGTTTTTTCAAGTAGGTTTCTCACCAGATTTAGAGGTGAGAAAAGGCTCTGTGGTTCTCATTCCTttgtaaatgaaggaatgaattcatccttcatttttattccttaCCAGAAAAGCACATCTAAGATAAATGATTGAAACAAACAGGAACACACAGGAGGAAGAGACGCTAGGAGAATGGTGTTTCCAGTGGGTCAGGATGGTAGAATACTTATGAAGCAGTTCTTCAGGAAAGGAAGCCCTAATTCCTGAGGAGAAGGAGACCCCTCGTGCTTTCAAATAGAAGTTGAACAGAACAATAGCTCTGTCCTTGGCTCTAGCTTGAAATTCACTCTGGACTTCACTTGCGGCTGCTCAAGTTGCACTGAAGACAGGTAGAGAAGACAGGATGGAGAGCCATCCTCCAGCGGGAGGCCCAGGCAGCGCGCCATGATCGCCTTGCACTCATCACACAGGCTCCGGTTAACTCCCCAAGACCCTGACCCTCTCCTGCTCTGTATCTTGTGGGAGAAGCCTTGGAGATTCCAAATCAGTCAGCCAAATGTAATTTAAAGGTACAGCAAAAAcatattttaggaataaaatctgaagaaaaatctGTTTCGTATGCCTGAGTCAcatcaatttgggaagaaatcTGATGCAAGAAGATAAAATGCCAGCTTCGGTAGCAGAAACCAACCGCCACATCAAAGCTTGTCATCTGCCCCTTGGTTGAAAACATGGCAGGCAAGCGGATCTAAAGAGCAGCCCGCTGATCATTTTATGGGCATTGAAGACTGTTTTCAAAGGAACTCATTGCACAACAACTACTTACTTTAATGGGATTTGTGTGGTTCGGGCCCTGTAACCTTTGGAAAATTGAAGGGAAGTATAATTGTAAAAGCCACTAAAAACTGTTTAGCTTTTATTGCAAGGCTTATGAAAAGCAGTGAGATTCAGCTGTGGCATTCATTTGCGTTTCTTTGGTTACACTAAGCTCCGATTGTAATTAGGGCAGCGTCCTTTTAATAAGTGCTCCGATAATTGGGCACGTGAGTTCCTTCATTGGGATGAGTGTAGATTGCACTTAGCACATCCCAGTGACCAAAGCCAAGTTCAGCAGCACATGCACATCTTCTCAACAGCATGCCTCGTATCCTGCACCACTGAATCCCAGCAACTGTCAAGAGTGGCCAGGTCTCAGCCAGTACGGATGCCCAGCTGTGCAGCTGCCAGAATCTGAATGTAGCGTGATGGTCTTTCAtgagtatatttttttcctttaccatcAACTTGGCATAGACATGGTTTATTTTCAGTCAAGCATTAGTCCTAAACCTTAGAAAATAGCATGAAAACACCTTACATGTAATATGGGGGATGTATGTTCTTTGGAGAATAAAATGCTCCACAAAGTGTGAAATAACATTATTTTGAATAGTTTTCACCCAAAAGATGGGTCAGTGGCTCTATGTAAAGCTACAGTGGTTTTATGCAGATACAGCTCAAAGTTTAGCTCTCTCAGTATAGCCAAttctcatttaaaacagaaaacacttaAGAACATGAGGTATCATAATACAATTTGCACTGCTTTTGACTTCATCTCACACATTTCCTTTCACCTCTCTAGGACCAGTATATTTCCATAGAGTCAAAAACTTCAACACAATCTGTGTCACCTTATCTGCTTTAACTCTCTTAAGACATCTTTCTGAAGGGATTTCTGATCAGAACCTTGAGGATGTCTGACATAAAGCAAGCCTTCCCATGCCCTAGTTTTCTTCTGGGACTACCTTGATAATTTCCCCCATGTTGGCATTTTTCCATGTGACTCTGTGTCTATTTTAGATGGTAACTTCAGAGTACAAAGTTCACATGTGTGTTTCATTGAAGCTCTtatcagtgctcagtaaatacaaaTTAATGATCAGACATGAAATATTCATATATTGGAATTCTTATTATTATAGGAGTTCATTACTGAACAAAACACCTCTTTGCTAAAGACATAAAATTCTGGCTTCAGATATGTAACCTACTCCCGATTTCTCTATGGATGAATCACTTAATTACTCATTTTTCAATTCATTTCTGGATAttgaataaagaggaaaatgagaatgtTTTCATCGGAGCCAACTAAACTATaatgaggagggagagcaggggagtAGAGTGGAAAGTTAACTGGCTTGACGGTCAGAATGACCTGCGTTCTCCTGTAGGTCCTGCTACTTACTAGCCGTGTGCCTTGGGTAACACActtcacttccctgagcctcagtgttcaaaaaaataaagtaaaataaaatggaagtaagACCACCTACTTCCTGGGGTCATTTGAAGATTGAGTGAACACAGAGCAGGCAGGtagtctggcacatagtagtcgTTTTGTAAGTGTAGGCTTTACACCTGCCACCGACCACTGCTGCGAGTTCTCCTTGGAGACGGACTAGTCAAAACCATGGctgttcttcctttttacttctgtTCGCTGCTTGTTGGAGTCTAACTATTCAGTAAGATCCTTATCACAGCATAACCAGGAGAAAGTGAGAAAGACATTCAGATATGTTGATATGGCTTAGTTAAGGTTTGATGGAGAGCATAATTAAGATCTAGGGAgcttgaaaaatgaataaaagcttttaaattaaaaaaaaaactgagacatGACAGTAGACTCAAAATTGCACATGGAAATCATGCATCGAAGTTTGTCCAAAAAAGCCCTATTTGGGACACTTTTCTGCTGCTAGTCAATTCCTGGCTTTCTAACAAGGcaattttcctttctcaatttaaattttgaaaaggaacATTCAGAAAGCAGGCAGATTCTAAGACTCAAAGATAGGTAAGCAACCTGTACCTTTAAAAAGTAATCTCACtagaatgagttttaaaaaaaaaaagacaggaaaaaggaggaagacagCCAAGGGCTAAACCCAGGCTATGAAAATTCAATGATTTTGCAGCCTGTTTGCCTTGGGTTGAATAATTTGGTTGCCACATGTCTAAAAACACAAAAGTTGGGTCATATTACTCAATATTTattcagtaactttttttttttctaaaagtctgAGCGCTTTGGTGAATTCCAAAGGgataaaaagaaaccaaggaaGGCAAAGTTCTGGGAAAAGAGTGAGTTCTGACTgagattttaaaagctttctaaaCTCGCAATATTCCAGGTGTGTGGTCCCCTTGGAAGCGCCTGGTATTTGAGTAGGATTACAAACAGAGCTCTAGAGGGGCCCTAAGCTCCTGCCTTCTTTGGGGAGCATTTTTCCTTTGTAGTGAGACTTGTTGTCATTTTGTAAAAGACACAACATCAGTCTCGACATCAGCAGTAGGAACTGGTGTCTTCAAGGGAGACAACAGCcataagtcattttttttttaatgattcatttttggggtaataaattttaatgttctcaagaaaaatgtggtaaaaaaaaaaaacccaggtttaaaaaaaaatagctggaAGCAGATAGCAATGGAGGAGATGTACATAATTAATGAGTCAGCTCAGCTCTTGGAAGGACAGTAGTGGTGGAACGAGACTCTTTTACTTGGATGAAAAGACAGTTATGAGGCATTTGAAGGCTTGAGAATAGAAGCATGACTTGTGCTTCCTTATGCTTTCTCGTCTCAAGTTAACGAGACAGATGCCTATAGTAGAGAAAACCACAGAGAACACAGAATCAGCTTTTCATCACAATAAAGATGGAGACCAGGGTAATTGTACAGAGAAAATGAGGAGGGCTCAAGAGGTAATTGAAGCCCCTCTCAGGTGTTACTCTGGTCTCTGACATCAAACTCATATAAAGACTGAAATATGCCAAAATGGTCAACATAAGAAGCCCGGTATTTTTAAACaaccaagaaaattttaaataattttcaagaatTAGAAAAGCCAAAGTATAAACAGTGGTTTAAAGCTTGACATTTTTGGAGAAATAGATCACACTATAACATCTCTGGGTTTCAACTTTCTTGTCAGTAAAATGGGTCACTTTAAGATATAaacaatatatgttttttttaaattgtaacaaCTAGGTATAcacatttccttccattttgctttaaattctgtTATGGAACAATTGTGGAAAGGGCTTTAGATTGGCAATCAGGAGGCTCAGACTAACTCAGTAGGATTACTGACAAAATCTGTGATGTTAGGCCTGAAGTTCTTTACTGGAAGAATTATAGGTTACATTAGGTAACTAAAGATCTCTTTCAAATCCAGCACCATGGGTTTTTAggattgtttttatattttcagtatctAGCCCAAGAGAAAACTTTGAATAATTGTTTGTTTAAGGCAATGACTGAGTCATGTCGTCTTGTTTGTGTTGCTGgtatgtttttgattttgctttgttttgttcttgtctTTGATTTGGAAAACCTCTGGCTAGAGGATAAATTGGATGAAGACTATCCTGTAGTCACTCATGCCTGGAGGAAGTTTAGGCGATTGACCTGTTCCTTCATGCCCAGAGCACTCTGCAGAGATAAGTCTCCAATTCGAAATTAATGCgaacctttattttaaaagaaattagttCACCTATGAGCACTGTCTAGAGAAAGAGGGAATCTGGTAGGGTTCTAAGAAACAGTTATTCTAACCTTTAAATCTGCtaaacagaaatgagaacaaTTGCTATTTCTTTCATTACCCCTACTAACTTTGACCTCCGATTAGGGTTTGCTGGATGACAGTGGACTTGAGGGACAGTTACATGGGTTGGGTGACAGTGGACTTGCTTAAAACACTGTCTATATCAACCATTTTAGGCTAATTCAAAAATTGGTGCCACTAACAATGGTCACACTTAACCTTGACCTTCACATCCTCTCACTATCACCGTAGTCATTTGTTTGAACATGTATTCATTGAGCATCTAATGCATGCCAGGCGAGTAGGTACcctgaaaaatacacaaatattatgTGTAGTCCACCTTCAAGTGACTTTATAACTGATTTGCATAATGACAGCACAAACACTTGAAAAGTAAAGTAACAATAGAAATAAGTTATTCCAGTAGATGGAGTGGCTATCAGAGCCAGTaatgggtgagggaggaggacagaAACACCAAAGCAGGTGGTTAGTGAGAAGACACAGTCAGCATGAGCCTCTCTCTACCATATGTGAACTTCAGCATCCTTTTCTCAGCTGCGTTATGACTAGTCACTCCATTCAGCCCATTCCAGTTGTGCAGCTGATGATAGTGACAAATCACGCCTTCCCCATTTTTTATTCATATTCGTCTTTGCCCTTGTCTCCCTTTGCCTAACAACGTATCCTAGGCCTTTCCCCCATCCACTGACTCCTAATATTGacggctcctcctcctcctccaagtctCCGTTAACCGTCAGCTCTTCATAGAGACTGTCTTGATTACCACGAGAAGTAGTGTACTTGTTCCCTTCCGGCATCTGAATGACCCATGTGCGTTATAACTTAGGACTATCTGTCATTATTGCTATCAGTTGGCTCACACAGTAGAATATGggcttcatgagggcaggagcCTTTTATGACTTGTTAACTGTAGCATCCCAGCATCGAGCATGAGTTCCCAGCATACTTTAGGTTCTTTTCTTTGAGTGACTGAATGAACATGTTAACTGTGCTTGGTGAAAACGCCTATTCTATACCCAGGGTTGATTCACACTCAGTCACCAAACCTGACAGCCCTTTCAGGGGTGAGTTTCTATAAAGTCTTGGTACACAAGTTTTCCTGTTTAATAATAACCTTAATAGGGTTACAAGGCACATGGCTTCCAGAAGCATGTGTTACATGCGTCTTTCAAGACTTTGCATCCGTAATTATCTCCCCAGTTAAAAGGAAGCCTTGAGGCAGGTGAATAAAGTGTGTAGAACAGAGCCCTATACAGCTTAGCACTGGCAAATGTTAGACTTTGATAATGGTTGACACATATGGAATTATTGACCATTTTAGACATGACCTGACACAGTTATTTCtgttccaaatatatatatttagctttaGGTTGTATTCTCTTCTACTTCGTCATTTCTAGTCACTGTGTTTTCTGTTCCCTGTCACCGGTAAAGGAACTTGGGCAAAAGCATGACTAATTCATTCTTCAACATACAAGAATGTTACTGTCTGTCAACGACACCTAAACCTTAATAATATGGTGATTTCCTCTCCAGGGATATGTGACTTTTGACTAGTAAGGAAGCTGATGCACTAAGgtcatatgagaaaaaaaaaaaagaaaagcttttgggCAAAGCACTCAGAATCCACGTGGAATAATCTTATTCCACTGAATCATGTCCCCTGCTGTTGAACAGCCCTAAAGAACTCCTTGTTAATAGGTGATTCTGTCTGCCTGGAGTGGAAACCTATGAGTAATCTATGTGTCACTGTGACTCATGTCATGGCCACCACTTCCAACTCCCCATCAACTGAAAGTTggcaaaaagaatatatactgcTCAACTCAtgacaggaaggagccaggacGGAGGAAAGACCTAATAACAAGTATTattgattaaaaagaagaaagctagCAAAGGAGGATGACCAAGGGCAAGAACAATGGCTTTTTAGTCACCACCTAAATCATTCTCTTCCTTGACATCTGTTTCCAATGTGCTTTCAGTGCCAGCATGGCCATTCTTCCCTCCACATCCTTGGATCTTTTTACAGATTTTccctatttttagattttttttccccaactcttAAATTACTTTCAAGAAAGGGCTTTGATAGCAATAAGTTTAACCAAGAGGAGGGACAAATCAGGACAAACTTGTGTTTAATTCCACTACAAAACCATTATTCAACCCGACAGAACTTTCGTTTGCTggctttcttcttattttttctctattcctcTGCAATTCTGATTAAAATGGCACATCATTTGATGATGTGGTTAGCTTACTGGGAAGCTGGCACCTTTAAAATAAAGTGTCTTTGTGAACTCAGGGGactcacaaaatatatatatatatatatatatatggattagGTGTGTAGGTAGGAGAAGAGATTTTTGAAAACCAGATTTTATAAACATGTTTGCTGTAAATCAGTTGAGGAGAGTGTGTGTACGGTAACCGATCTGGGCAAGAAAGACCATCCTGAGCtgcagttttatgttttttttaatctagccAAAGAGGGAGTTACTTATAAGGAAATCTGTTCCCTTCCTGCTATTCAGTGTATTTTCTGGTCCTACTGGGCTCCTTCACTTTGTTCTATTTGGTTATTTTCACACTAAGGTTTTCTAGAGGTTGTGCTCACACCTCCCCTTGAAAGTATCAttaaacttctttctttctacaaaTCTATTAGTTTGGGAGCTAAGCTACTTGGCTAGAGGATTATCATGACTTTCTCTCCTGGAGACTTTCAGTCTTAGTACCAATCCCAGCTCTTTCCAAAAGCCGGAGGGGCACTAGCTTTGTTTGCCACTAAGCCTCGGTGGTCACCATGTGACATTAGTGCAGTTAACTTCCTGATCTTAGACAGACTATTTTTTTATATCAAAGTTTATCTCTAATTAGTCATACCCCAGACCAGATTTACACAATAGTGATTTTATACCCTTCCCCAGAAATCCAATATTTATAAGTTTTTCCATAGAATTTTATCATAAAGATGGCTTCATCTACTTggcgggggctggaggagggaagtaCTCCAGTGGATTAGAAGTCAGACAACCTGGGTTCTCGACCTGTCTGGTCCTAAAaagtcatgtgatttttttttccagttgcgTGATCGTAAGAATCTAACTTTGCCAACCTGGGTTTCCATTTCTGTTGTTATCAAACAGGGAATGTTATTAGActgtttttaatatcttttactgattttaaattataaattcttcTTCAAATACTTGACCAGTGATTAAATTTGTGGCCTCAAAGAGGTATACCTTAAAGAATAATACATTTAGTCACTAATTATCTCCAATTTTGGAATCTGTGATTACCTAGATATCATGCAATTCCATGAATACACATTTTCGTAGTCAAATCTGCAGTGTTCTTGATGTTTTAAAGAACATTACTGATGATCTTTGTGGATATCAGGGTTATTTCAACATTGCATAGatgcttattttcttttagataaactctaaataaaatgttcagataTAGCAGGTATTCAAACAGTTGAGAAGAGAGCAAAGATGAAAAACTGTTTTATAAAGTTTTGGAATTTTAAAGTAATCTAACATTTAGAGATGGGGGACCTACTTATAaaatgtttgtgtgcatgtgtgggggtgtgtgtgtctgtctgagtGTTTGTGTGACTCAGTTCAGAGACTCTTGGGTTGAACTACAACCGTGCTGTAAACATTAGAACCAGAAGCAGATCTAATTGCCCACAGTGGAAAGGACCAGCCTGCGGCACTGTGAAAGATTTTACTTGTTTGGGCTTTCCATGTAGCCCACTAAAACTGCTGAAACCTATCATCATTACCTAGTCTCACGCCAGCTACATCTGGTAACTCACTTTGACATATTAGTTTCTAGGTAGGAGCTCCCAGACAATGGCTCATGTGATGTCAGTGCCAGCTGGATTCAGctgtaaacaacaacaaaaaaaagtattttaaatagatacttcatatgtttaaattttaaataaacaaaggcAGAGTGCATACGTTTCCAAGTAAGTCAGTTGCCCAGTTGTGCTGGTTGGTACAACCAAGTGCTGTATCAGTGGGGTCTTCAGCTGCCGCATCGTGCAGACATGTAGGAAAAGTGGCATGAAAAGAGTGGGCGTGCACTAAAGATGAATCACGAAGGATGCTCTTCCATTAAGCTCTGAGAGTTTATACACTGATACCATCCCACTAGGACCCAAGTAATACGCAAGCCTTTGGAACCATTTGCAGTATATGCTTCAAAGTCCAGACCAAAGCCACTAGGTAAGTTTTATGAGTCCCTCTAGAAAAGCAGTTTCTTGTCAGGGCTGGAAAAATAATGTTACAAAGGATCCAGTAACAACAGAATTTGGGCCTGTTAGTTAGATTCCAAGTTGATGCCTCTTCCAAAAGGACAGCATCCAGAAAAACAATTTATCCCCTAGGCCAGGAGAGTAACTGTAACATTCAGGATTAAGAAAAGTTTGATGTATAGTCTATTATACACAGGGTGCAGAGCTATGGGGGCAAATCTGtctaccattttatttgtttcaacaAAAACCATCACGATGGCAAGTTTCTCCAAGATCATCCCTTGTGGGGGCTCAGGACAAGAGACAGGAAGATGTATGTCACTGGCTTTCCAAAATCTAGCTTGCTTCTCAGAGTTCCTTTAAGAATACAGTACAGCTGAGAGGGCAAGTTTGCATATGGTCCTATACAATGACTGCAGTGACCAATGGCCAATAAATCAATTACATCTTGTGTACCCTATTTAACCTTTGGATATGATGAATATTTGGAGTGTGTTTTTTGTGGTCTCAAGCCAAAAGTTAACCACAGCCTAGAGATTTTTAAATCTCAGGAAAAGAACTAGAAATCTATGTTCCCTGTTTTATTCTCAGTGCAGTTGGAGAGAACAGAATTCTGTTAAAGGGAGACAGTGTAATTGTGTTCATAACGTCCGAGGGTTGAGAGGTTGCTTTTCCATGGTGCCTTTCTTCCAAAGATCATAAAACACTTCACATATGctatttcatttatattccaAACGTCTTTATGGTGTAAGAAAAGAGTACTTAGTACAATtctaggaaaaaattaataatttaatgcaGTTTAGCAAAACTAAtcaaagtaacatttatttaatttaatatacaGGGGAAACCAGTATTTAGTAACATTCAGAATGAAAAATGGTCAGTGAGtgagttcattatttttatctttaattaaaatttttactttacttttcccAACTTCTCTAGATCACATTgattattctcttttcttaactCTAACTGTGTTGATGTGTTAAGTGCTTGATTCACACATTTTGTCACATTGTTATACACTGTCATATtgccttctttgttttctatgtattgtTGTCTCAATAGCTACTCACAGGGAGAGACTGGTGTATACTGGAAATGACACCGACTGGAAGACAGAGCCCTGCGCGCCAGCCTTGTGTCTGCCCCTCACTGTCACTCAGCAAGCTCACATCCTTCCTATGCTCCAGTTTTTTCACCAGCAAATGGGGGAAAAATGTCCgcacaaatgaaacaaaatcttaaaaaattgttGTGAGGTTCAAATaagatggggtttttttttcccctaaaaatacTATGCATATATGTAAGACATTATGATGATTAACTCTGTGAAGGCAGAGACAATATTTTGTACCTTTCTTACCTATTTTCCAGGACCTAATGTGATGTTGTGTTAATATGATTTCCCAACGAAATATTTAATTAATGTCTCTTAATAACAATCACTGAATACTTACTACTTTCCAAGAATCATTGTTAGGTACTTTACAAGGAACATTACACTTAAGAGGTTGCTACCTTAAGAGATCGTTTTGTTATTACCTTCACGTTGTAGATGGTGAAACCGAGGCTTCTCCATCTACAAGCGAGGCTAAGCGGCCTGACCAAGGGCACGCAGCTGACACTTGACTCCTGGCAGTTTGGCCACACCGACCAAGTTTTGAAACTCTTTACTCTGCATGTCTTTCATAGAACCAGAGGTCCTGGAGGAATGCAGAGGTTATGAAACTTCCTtcttaaaactattaaaaagtgGAAGAATATTTAAGACATGGATAGGCATTTGTGAGAGTGTGCTGGGTAAAATTGGAACTCAGAGTTTGAAAT |
|  | TGATAAACACACCAACTTCTTGATATATGTGTTTGGGTTGCAATCATATGATTTTCTTCAGAGGTGTTTAGTTCAGAGAAACACTTCAGAGCATTTGATttggcaaaatattaaaaactgaagttACAGCCTGTAAGATATTTCTATTCATCTCCTGGTCAGCTAGTTACAAATTCTTCTCAGAATCGTGGGAGCATAAATCCGATTATGCACAAGGCCAGTGGGAACAAGATAATTGCATAGATCTAGTACCCATCCATTACACTCAGCATCATAAATCACTGAACTGCCGCAGCCTCAGCTTTCTGGAATCCCTATGTTATTTGAAATACTCTACTATGGCCATTTATGGAAGAACCATATTAGACTGTCTTTGCCTTAAGtgctatcaaaataaaatttaaaattcaagtgtTTATCCTTCAGAGGGTGCTCATGGGGTAGAACAatggaaaagatgagaaaacaaatcaaaatctgATATCTTCTAACATGAATGGGGGCAAGCACCTCCGTCTAAGGAACTGGTTAGCCCCGTTAAGAAATTAAGTTGggtagttctctttttagttctttctgtgatggaaattaacatttgttaGCAGAAATATGCATCTGTTTTTCCCTCACATGCACCTCACGCACTTGCCAACTCCATAGTGGGGAGTCTGACAAATGTGTACTTCTTGAACAAAGCAAGCAAAGCATTTGGCCTGAGTAGAGATGAAATGATCATAGCTAACAAATTAAACTGAGTTCATGCCCAAGACTTATGTGAGCCTTTAAacaatggaattaaaattaaGTTGCAAGGATGAATCAATTCAGGTTTCCTATCTcgcattttctttctattcaatGTAGCGCGTTTATTGAGCATCGTCTGGGTGGTGGGTATGAAAGAAAGGTCACTGGCCTCCACTCCCGCAAGGAGCTCagtttagtgaaaaaaaaaaacaaaaaaccagataCACAAACAAGCAACTTTGCAAACATGAGCTAAGTAAGATAATGGAGGCATGCACTGTAAAGTTGTGGCACAAAGGAGAGATCGACTATATCTTAATAGATGAAAAAAGAGccacagaaaatgagaaactaaTTGCACcggtttttaaaggaaaagaaatacatattttgaccAGGGGAGGAAATGGCTTGTTTTTGAATGGAGCAGAGTGATAGAAAGCAATATTACTCATTCCTAATAGTggattttcacttcattttttcctCATGACAGCCCCATGGAGGAGCATGTCACTGTCCTAATTTTAGAGGTTCAGAAAAGTAGACATCTCAAGAATGCAGGGCAGATAAATGATATGATCTTGACTTTCCTGTGTTGCCAAAGCTGAGCACTGAGAGCtttgagaagaggagaaagatgaaGCTGAAGGGAAAGCAGGGCCAGCTGGTGGAGGGCCTCCTACGACATGCATTTGGACAGCATCCTTTAGTGCAGGGGAGTCAGTAATGAGTTTTTCAGCAGAGGAGTGGGTGACAGCAGCAGTTTTGCCTGAATTTCAGATAGCATCCTGGTAATGGCATAGAGCTTGGATTTGGCTGGGCGTGAGGCAAAACCTCAGTGCTTTATGGAATGAAAGGTCGGTACTAATAACGGGACCCGTCCTCCTACTTTTTATGGTAAGGATGCTTCAGGCATCTCTGAGTCTTAGACTTCCACCCTAATATCTCTGTCCATCATCTCCCACTTTCTGTCTTGTTTGGACCTGTTTTTAACTCTCCTGCTCATCTTTCTTTCAATCTATGAAGTGTATGTGTATCTGAGTTACTAAAGATGCAAAGAACAAATTCACGACATGAAGAAGTACACTTTTTTAATGTACGGCAGAGCTTTGAAGGACCTTCCAAATGGGCCAAATATGTATGTCCAAAAAGTCCATGCATCCTACTGCTAGGAACCTGCAGTAACACAACCCATCAAAGGCTCTGTCTACACTACCTGGAAGTGCCCCCACCCTCCGACATTCCACTTGAGACCTACTGAGCCTCTCAGGCTCCCAGGAGCCTTACTTCAAACAACttgatttataaaaaatgaaaattttcaaaagcatAGTATCAGATGATGAAATATTGACCTCAggtattttaaagttgctaacTCTACTGgtacatttgaaatataattagATATTTATGAAAACCCCAGAAATGCATGCATTTGTACTTTGAGGCAAACTGGTATTTCTCATCGTGTTTTTTTGtgttgctttgtttcatttttaaaggcagtaATCCCAATTTAAATCCAAGCAAGGCTGTCTCCTTCTGGGCTGATAGGAacacatttctcttttccttgtcaTTCTCCAGCTCTTTTTGCTAACTTTCCCTTTAGTCTTGAAATGGGCAGACCCCTCAGGATCCTCTTCCCCAGTCCCATTGCTCACAACACAGTTTCCTGTGAATTGAGAACGTCACCCCCTTGCCATTTATCTGGAGGAGCTCTGTGCAAACCACAGCTCCAGGATGGAACAGAGAAAGAAGTGACACATTCAGAAACTCAAAGAATCCCTCTCTTTTCAAGGAACCAAACATTTACCTCTCTGAAAGAGTTTTATacaagaataaaattcaaaagttaaaaaaaaataaaaataaaaggctttatTAATGTTtgctggagaaacagaaaagctttTTCCACAAGAAAAGTCCTTTCTGTACTGAAATTGGCAGCTAGACGGCCTGAGCATTTATCTCCTGCATAGAAAGTGCTCTGTCAGGCAAGCTGTGATTAACAATAAGCTGAAAAACACGCCCCAGACTtccactgtattttatttggcagaGAGGAATTATTCCTCAGCACAAAGTATTTGCAACTGGCATCAGTTAAACAAGAAATTACAACCTTTAAAAGCCCTATTTATCATTAGCTGCAAAAGTGCAGATTTCACCGTCAATTACTCTGACAAAGGATTATAGCCCATCAGCACCACACACAGGATAACGCTGGCCTTGGCGGCCAGCAGGCCACTCTGAAGGGAGGATTAGAAAGTTAGCAGCCAAGAGCCCTTTCTCCAGAGACCCCCTCGACCCTCGTCTAATAGAGGCTGGTCTACCAGCAAGCTTTCAGCAGCAAGATGTGATAGTTGTGAAAGGCATTGCTTAAGAATCCTTGGCCAAATCAAGTCAGCGGCAACTTTGCTCCAATTCTGCTCTGAATGTGTTCTGttgataaaaatggaaatcacaagATTGgtttctgaaaaccaaagaaataatatGGTCTTATTCCCCTCAGCTGCTTCATTTAGAGGCGAAATGGTCAGGAAAATAGTGAATTTTAATAAGCTAAAGGCATCTACAGTGATTCTAAAACACCCTCCCAAACCCTGAGTGGTGCCCATGTATTCTTGGAAGAAGACTTTTTACATTTCACTGAGGCATTAAACCATCTTTAAATGATTTCATcctttaggaaacagaaaaacaaagagatgcTCTGTTTTCCagactttaaaattaaaacaaaatttattctcAAGTGCATATATAAGTAAGTGGTAAGACACCTTTCTCAAGAAACATATGTGTATATCCTTATTTAGATTTAACTTTCCAAAGAAGTGTGAGGACATTGTCGGTGTTACTGTTACTTTGTTGCACCTTAAGTTtactttaacttaaaattatagattaatattattaattacagATTCATATTATTATTCCCttcaacattttacttttattggcTATATTTATCTTAGTATACAAACATCAATATTTCATGAGAGTGAAGTAAaatcacattatatatataaaccacTTATATTTTGTTTGGTATATAGTGTTCAATAAAGGACGATGATGATGGTAGCAATAATGTTGataaagatgaggaggagggggataTGCAGTATTAAAAGTTGTGTTTATAAAGGATAAGCAAGTTAGACATACGGTATATTCTTAACTATATAAAACAGtgctcagggaaaaaaaggactagaaggaaataaaccaaaatattaaGAGTATTCACCCCtgagtaattaaataataaatattttctcttattaccTTTTCTATACACTTCCCTAATTTTCTATGATGAGCTTGTCTTACATTTATTATgaggaacaaaatagaaaagatacaGGCAAGGAAAAAAATCGAAACTAGAATGTTCTATCTGTAAGGTGATTGTGAATCTATATACTTTTCGCATCCAATTTGACAACCCTTCAAGGGAGACAGTTGTTCCCATTTTTACAGTAAGTAACCGAGATCTAAAGAGATGAAGAGATTTGCCCAATAGAAACCAGTTAGGAGCCACAGCCAAAACTGAGACACACCTCATTTTACTTCTAGGCCTGTGTACTTTCCCTTTCTCCTGGCTGCCTCCTGGAcggatgagtgagtgagtgtgtgtgtgtgtgtgtgtgttcacgttCCATACCTTCTCCAGTTAACGTGTGTTTAAGAAGTGGGTCTCTTCACATATGATGCTCCCGATCCCTCCAGATCAGAGCATGTTCCTTCACATTTGGTCTGCTGCATTCATAAAAACCATCACAGGGCTATCACTTGTGGGTGACCTCCTCCAAAAAATCAAAGGAGTCTGGATGGCTTTATTTAGCTCTGTTAGGTTCAAAGAATGTGCCAGGCAGATTCTCAGTCCAAAAATAATCTCCTTTTATTGGTGCTCTAAATTAACTCACAGAGGAGTGAGCCGAGCTAAATTAAAGCTTTCATATAGCAAGGCCCCTGGATTTTTACTGAGTGGTAAacgttttctctctctgtgagaactttactatttccttcctctAAGAGAGGCTGTGCTTAGATGCAAAGAAAGTAGCTCAAAGCAGATCTTGgagaattcattttatttgacaAAGACTCTTTAGTTTGAGTCCCCAGTGTggaatatttaagattttatagGTTTTCCTAGCTTTTAAGGtaatttacatgtaaataaagCGATACTAATCTTTaacacaaatggcaaaatgtgtTCTTTGCCCTTTTTCAACTGGAAAATTGGATCATAAGAGTCATTCGGTGAAGTATTTTTACTTATAAAGGACTTCAAATGGCTTGACAAATCTTAACCACATCCCTTCTGGACTGCCAGCTGTACCTTATGTTAACTGTTAGAACACTGGGGCACATTCCTCTCTGAGAACTGCACACTAGGGAACAGgcaccataaaacaaaaaatatgatattgAGATTCAAGGTTGCCTCTTTTTCTCTAGTACAGCCATGTCACTGCTGGTCGTGACTAATGATGCATTTATCACCCTTCCTGCGGTGTGGGAGTAACACCCACTGCGTTTCCAAATAGCTCTAGACAGGCATGCCGGGCTCGTCTTACTGTCATGTGGCCCTGGGACTTTTCATGTGTCACTCTCTCCTTTGTTCCTGCCTACCTGGCACCTTGCATGAGTGACCAGTGCAGATGTGAGGTGTTgatcctctctgtctctcttttgtgCTGGGTTTTggacacacacaccaccacaGGGAGAGGAAACTAAAGCCCCAAGCCTTTTATGTTGGAAGCAcctctctattttatttctccacACTGCACATCTAGAAAGTCCCAGAAAAGTCATCCTAAATTGCTCTAAGCCTGTGCTGTAATACTTTGACTGAGGTCTCAGCAAAGGTCCCAGAGAACAGAGCAAAAAAAGAGTATGTCCAGGGAGAGAAGTGAAAAGAGAAGATGCACTTAGAATAGTTGGGGGAGAAGATGTGGATTCATACCTCTTACAGATTTCTTGTTGACTGGAGATGTGAATTTCTAATAGGAACTAATAGAAGTacttttttttcatcttaactatacagaaatgttacttttaaaaaatgaaataaaattaattgtttcAGGGCCTCAAGTACatctagttttatattttattttttattatttatccgTAACTATTGTTCTTATATCTAATACCAGTATGAGGCTATAAGTTTGTTCATagaataaagactttaaaaatgattcctCACTGAAATGAGCAAAAAAGCACATCCACAATTATTAGTCACTATTAACTGTTAATCAAATCACTGGTTATATGTGTtgaagattttatatttaaaataatgaaccaGCAGTAATTCAGTTGCTCTCTTATTTTAGCACAACCCAGTATCCACTGAAACGAAAAGCtgtgtggtaaaaaaaaaaaaaaagaaaagaaaagatagatgaacttatatacaaaacagaaacagactcacagacatagaaaacaaaataaaggttacctaggaggggagggggcgagaagggataaatttggagattgagatttatagatactaactactacatctattttttaaaaatagataaataacaagttcatactgtataacacagggaactatattcactatcttgtagtaacttgtgaaaaagaatatgaaaacaaatatatgcatgttcatgtatgactgaagcactgtgctgtacactagaaattgacacaacattgtaaactgactatacttcaataaaatatatatatatacaaaaaatcaacactaaaaaataaataaataaactttgttttggccaaaaaaaaaaaaaaaaaatctttgatgtTTGACTCCAGCCAGCATACAAATCCAAAAATGTTGGTGGTGATAGTCTCCACTTTCctttttgagttttttatttttatttatcttcatattgaagtatagttgatttacaatattgtgacAGGAAGTGACTTGAACAGGAAACAGAATTGGCTGGGAGTTGGTACGTCCAAGTAAAAAGAACTCCAGCACAGCAGGCAGTAGAAACACTTTTGGTCCTGACTCTGAAGAAGAAAATACTCTCCTCTATCGAATTCTGTTTAAGACTCTCACACGTGTACATATTCCCAGCGTGGAAGAAGGACCTTTCCACCCAAGGACACATACTCCTTCCCCAAGCGTGTGCCAACAGACAGATGACACGAACAACGACTATATTCCAGAACAGTTTTACTAAAAGCAGGGAGGGACAATTGATGGAATGTGTAGGTATGCAGTAGTTacacagcattttattttgagTGGTACAGCCAGAAGGGAGCTATGGAAAAGCCACCGGTGGAACATAATGCAGATTAAACAGGAGCTCCGGTTGTTGGCATAGTTCTGTGACTAGCTAGCTGCTCTTGGGCAAATCACCTCATATTTCTAATTTTGCAGAGAAGAGGATTGGAAGGACACCCTCTCCAGGCCCTTCTAGCTCCCATAGTCCATGGGGGTAGAGTTATTGCATTTAGCCATTCTGATATCAGGTATCTTGTAAAGCAGGCCTCCGAAGACACCCCAGCGGGTGAACTAGACCCTTGCATTGCCAGGCCTCGGCACCTCTCCCAGCGCACTGGCGTGTGAGACGCGGCCAGTTCCCTGGTTTACCGTGCGGAGAGGCTGAATGACGAATTAGAAGAACTGATGCCTTTAGCCACCTCTTAGTTCCTGCATCTATTGTTactgtaacaacaacaaagagtCCTTAGATGTTATcgtacttaaaattttttatccatAGAGACACTTCAAGCACAGCCGTTACTCATTAGACTTCAGTGGCTGAGAGCATTTGGTTACTACGggaacagggaagaagaaaaatcagtaacGTTTTATTTACAGTCTgcttttattgtgaaataatagAAGTTCTTGAGTCAGTAAGAGGATGTGCTTGGTACTAAAAACCTAAATGTGCTTTCTTAAAAGAGTGGTAAACACTACGCACACATTTCCCCGATTTTAAGCACTATTCCAATTCTCTTATTTTCCCCATAAGTATGGTTACACTTCTAAGCTCATGTGACTAGcttttaaataaggaaatgaagTTACATGGGGACTGTGcctcttcaaaatatattattatacatgcacacagacatacatgtgtacaaaatttgcatataaattttagaaagataatttccACCCTACAAAGGAAACAGCTGTTCTGTGAGATAGTGCACTCCCTAGCTGAGAAAATCTTTAAGCAAGCCCTGGATTCTGTCTGCCAGGAATAGCAGAGAAGGAATTCCAGATTCCAGAATAGGTTTGATTCCATCACTTATAAGTTCTTTTTCAACCTTGAGTTCGAAGATTCTGTAAGTAACTATTTTTCcctaaattcatttaaaaatttgatcgAAGCTTTCTAGTGTGTTTCGTGTGTTGGCTGGTTCTACAACAAACATCAGTGAGAAACTAAAACTTTATGGTGCTGAAATTTGTATACATTCGCAGCCAGCATTCTGGACACATTTCCACCTAGAAACCAAAGTATGTGGTAATAAAACACTTTGTGGCAGCAACCTTCCTCCAAGAAAATTGTGAAGTGCTATAGAAAACTAAAACTGTATTTGCCTCATGGAATCTGTGTGTGGTGAACAATACTAGTCCCGCTTCTCACAGGCAGAAAACGTGGAAATTAGGAATcaaatgtttgtaaataaaatggaatcattgCCCCCAGCCATTTCACTTTGTAGGCAAAGACTCATTAGCAATTTCCACTCTGTAGACTCCTTGCTTTCCTATTACATCTCACTTTCCTCCAATCAGATCAAATTTCTGAGAGGATAAATTTTCCTCAATCCCTTGTTTCATCTCAAAATTAAACCCAATTCATGAGCTGCTCTTCTCATCTTTTTAATTCAATTTCCAAGAAATTGCAAGTAATAAATTCCTCCATCAACCCCATTACCCATCCTCCATCATACCAACCACACTATCAAATGTCTTATTCCTTAAAGTTCTCCTTTTAAGAGTCTGGCTGAGCAAGTTTTACCTGGCAATAccaattttcaaattatttacaaCGGTGATTGTTTAGTGAAGGTTGTGGATGGCAGACGAGGGGGATCACAGCCCAGCAAGAAAGTCAACTGTTGTTCTTTCCTGGTCGCCTTCATAAGTAAGGCAGTGCTGCCATATCAAATATCCTGTTTCCTGAAAACCTGACTCTGAGATTGGTATGTGCAGGAAATTACATCATAGCAGAATTCAGTCACGTTGACCTTAAGTGCACCCATATTCCAGCTTTTgccctctgacctcacctccttAATCACATTTGCATGCTGTCTCCACTCTTTATAGTAATTTAGAACCTTTCTACAGCATATTCTATCTTGCAAGCCATTTATTAATGCTAATTCATCTTTTCTCACCCTCATTCCTTTGAGGTAGAGCCCTGTGATTAGTAATAATAACAAGACCATGGTATTTGGCTTCTCTATAGCTTCTGTCTCCTGAGAACTCTACAAATATTAACAGCACGCACAGCGGTCTCCCAGAATGGCTCAGGTCACGTAAGGATCGGAGAAGAATGCTATGACTAGCCTAAGTGGACTAAATTGAAGGTTGGATTGGAACTGAATCAAGATGCCGGTGTTAATATGCCCAACTATTTAGGAAAAGTTCCAGGATCACTTAATGATGAAAAGCAATAAGGCTGTCAAAATTTGCCTGGAATATATATAGGATTAGGCCAGCTCCTAAAATTTCCCAAGGTACCTCAAGGTCAAGGGCTTTCTGCAGAAGGTAAGCCTTTTTGATGATACAAAAATTCCAAAAGCCCCAGATGCAGAAAAGTGTAAGAGAGGTAGgggtgcgtgtgcgtgtgtgaatgtgtgagtgtgtgtgtgtgtgtgtgtgtgtgtgtgtgtgtatgagagagagagaaagagagagagagagagagggagacagaaagagaattaGTTATGTAGTCAAAGATTTCTTTAACTTATTTCCATGAAGTTTCTCTACTGGATCCATTTCAATCCTCAATGCTTGTAATTCCTTATCttgaagaaaaaggcaaatataattCTTGAATAACAAGAGAATAATGCTAGTGActtgaaataaaaacagctttaaatTTTCATCAGATATATGTAATGCAGAACTGAATAATATAAAGTTATAATGCTTATAATAATGTAGAGCTATAATAATATAAGGTTATCTTTTAACTACATTCTAGACGGTTAGCTACATTCCAGTGTTTAATTCAGTAGTGACTCACAGAAGGATATTCCAACAGGCTTACTAACCCCTTTTCTTGGGATTTTTAAGGCTCTTCTTTTGAGTTAAACCCTTCAAGTTAAGAATTTAATCAAAAGGTTTGGAactatggaaaaataattatatcgTTAACCCATAAGGATTAATGTCAGTTATTAGGAAGGACTTCCTGATCTTGGACGATTGTTAATATAAATGCATATTCTTTTCTGGAAATGTAAACCTGAATAAATCCTACTCTAATTTTGGATTTCTAAGATTAAAAACGGCTCCTGTATCAAATAAGTTGTTCCTCTCATAACATcacttttcatttgaaattatatttatttgtgtgtttatctaATCTAGTGTCTAGATCCTAGACCTAGCCATCAACTGAAGTTTCTTAAAGGACAAGGAACTTTGTATTCTCAGAGTGCTTAACACAGTATCATGCACACAGTTGTGCATTAAACATGTCTTGAATTAATGTTGCAATAAGGCAGGTTTGAGTGTTACAATAAGGCATAGCTCCCCTGACCCCAATGAAATGTCACATCGTCTCCGGAAACTTGCTTCTAAGACTCTGTCAGCCACCAGAGCAGCAGGGTTAAAAACTGCCCGCTTTATGTTAAACCCTTGTGACTGTgccacattattttatttattttcaattctgtATTTGTCACTCACATAAgaataggggaggaaaaaagagggagagggaggaggagaaaagaaaaggtgggggaggagagaaaaagaagaaaaggagggaaaaagaaaagagagaggaagaaaagatgggaaCTGGGGAAAACTACATAATCCAATGTacctgaaataaaagcaaaaacatgagaaagaaacaaaaaaagagagggagggaagcaggaaggaaggcagactcttgcattttctgaaatttccatctctctgaagAATTTCTAAGCTTTATTTATACTTTTCCCAGAATCACACTTCCAGAGCTCTCTGACAAAAAATCCAGGTGACCCATATTTAATCCATCCTGATCGCATAAAAATATCTGAAGGTTTAAAGCCCAATTTCTCTTATAATTACTCTTACCACACAAATCCTTGTATAtaggaggaggtggggagtgtTGCCAAATGTGTAGTCTCTCATACTTTCAAAGTCAAAACTAGGCTACTCATGGTAACTACCGATTTTGCTTTTGGGTACGACCGTGACTAAATGCTAAGACCCACCCAGCTTTTCTCATACCCACCACACACACTTGAACTGTTCACAGTGGCAAGATGGACTATTACGTATAATAATTTCTCTCCCCTAATCTGTTCTAAGTGATTAACATCGATTCATCTTTCATTATGCAACTCAACTTTCATATCTTCCACGAAGTTCTTCCTTAGCCATTGGGCTGACAGCTTCATCCTTTGTAACTCTTCTTTATCCTGCCTTGATTCTGGCTCTATCCTAGAATCCATAACATGTCGTTGATTTAGGGTTCGCATGTCTGCCTCTTCTAAACTACAAGGTCCTAGGGGGCACCAGACCTTACATAATACAATATTTCTAATGCATAAAACAGTGCGTGCAACATTGTAGTGCTCtgtgaatgttgaatgaataggagagaagggaggaggagggccttATATAGAAAGAAGTACTAATCAAAAATGGGTAACAGGAGCTACAGACCTTCTGGTAGGTAGTGCCTGTATTTTCCAAGTGAAATACTGTCTGGAATCTGATGAGAAAACCGTTTCATACAGATGATAAATGCCAACCTAGTCTCTCTcaacaaaatgtaacaaaatcattaaaacattttcctttttttatctgCCTgcgttacacacacacacacataacagtATTGAACCTGTTCAGTTTGCTCTCCAGACACCTATCCTCTTTCCCTATGACATAATCCAACCTCAGTGCCCCAAACTCCCCGCATCTCACCTCATATCGCCACCTGGTGtctgaaagcaagaaaaatacctaagaaatgaaaaggatttttttcaagcTAATTCAGGACCCTAGAATCATatactgaaagtttaaaaaaaatcacacgcTTCTTGGCATAGAATTCTGGTCACAAACAAAGATTAAGTGCACAAAACTCCCTCAAAGCACAGCCTTGTTTCTACTCacatcttcacacacacacacacacacacacacacacacacaaatgctctCAATCTTTTCAGCTCTGTGGACCACCTACCCCATAgattccatgttttaaaaaatttaccagaaaataaattataacttgTTATTTGCATTAAATAAGCTCAAAATTGTCAGTCTATGGTTCCGGTTAGCCTGTGATAGCCAGGAATATCATCATTCATTATGTGTTCAATCAGCATTTTAATGAGTCCCTACTGTGTTCTAAGAGCGATACAAGGCTCCACAGAGTTACAGTAAACTAGTATCAGTCAAAtcaagaaatgtgaaaaattgtTTTGTCTGTAGAGAgttaacatatattttcattaggtgtttcaaaataacaaaattagtAAAGTTTTCCTATTACTGCCCCTAAGAAGTTGGGGAATCTGAGATTGGGTACCAGTGGATAAGGAAAAAGTGTATCCTGATGGATTCAGTACAACTTCAAATCAACAATTCCTGAAAGAAAGTCTCAAATGCCTGCTTCTAACTCAGATTATCTTTCATCTGGTTTTAAATATGCAATTGGTTGTTAATGCATATTGATGATAATTTTATTGTCATAGAACTAAGAACTGAAAATGCATAACTAAACAACAGTATGAATTTTCCTTGAACTTGAGTCATCACAAAATCATCACATGAGTTTCAATAGTGAAGTCAGcaattatataataaatgcaGGAAGCAAAAAACTCCTTAATCAAAGATTCCCAGTCAACAGTGCTCAGAACAAGGACAAAGCTTTCAAGTTTCCAATACAAAATGGAGAAGGGGGGAAATAAGTACAAAGCAGTGAGTTTCTTCATAAGTCATATGCACTTATCAGTTTTATTCTGAGATTacagtcattttattattttgatgcaaTAATATGCTTTCCTTTATGAAAGATAGGGTCAGAAACGAGTAGGAGGTTTACAAAAATGTCCTTACTTGGCTAAATAAGTAAGGgtactcagaatttttttaaataaaattttacaaataaacaaaatttctgAAGTCTGGATGATCCTGATCGTgaatctgaaattaattttgaaggTTTAAAAGTTGGAAACCCTTAAAACCTGACTTGATGTTATCTTtaagagcaaattttaaaaatccacttgcTTTTTTCAAATGGACTGGTTAAATAAGAGCAATTtctaagaaagacagaaaaaaaatcaaatacttcaATTTTGGCAATTATAGCCCCATTCCACAGATCCCTCTTAACACTATGTGACATTACATTTTCTTCACCCGCATCGCACTCTGTGTGTTAGTGTTTCCTTCAGTTATTTTACAGTGTTTACTGGGTCCAAACTCAGCAAGCAGTTCTAGGCTTAATCTCCCAAGAGCTCTAGGTTAATagacatcatcatcatcatcatcatcatcatcatcatcatcatcatcatcatcatcaaattttTAGAGCTATGGCACTGGATGTAGAGGTAACTCAGCAAATAAATGGATACATAGCCTagatgttttccctttttaaagtCACAGAATAAGCAACATATTGCTTCTCGAACAAAACAGGCTTGATGCTTGGCTTCCAGCAAAATCTATTTCTCTCCCGccccttctgtctctccctctttctctctccttctctctcaccaCAAAGCTTTCCGGAGCGAGGAGAAGTATTTGGTCACTTGCTCTTCGACCCCAGAGTCTCCCgagaatatatattcattttaagaaTGAGTCCCTTTAGTTACCTAAAGTGTCCTCGGACTAAATTGTTTCCAgatgtttacaaatatttcaagGAAATATTCACacagtttttatggctgagtgataaaGGACTCCGCGGTGTCTGCTCAATTGTAAAATCAGGCTCAAGCGCTTACTACGAAATCACTTCCATCCGGCAAGTATTTGCTGCACTCCCGGCACACGGCTGGCCACGCTCAGCACGGTACCTCACTGGACGCTCAGCACAGCCCTACGTGGAGGTGTTGTAATCCTCAtttcagagaaagggaaggtgAGGAAATGGGCTACTATGCCAAGTCCAGTTCTTGGTCGGTGGGTCCTGCCTCTCTCTCAGAACAAAGCTCTTCTCCTGTTGGCTTagccacagaaaagaaaggaagagaattcaCAAAAGTTGTGGAGGGTCCCTGAACATCCAAAGAAATTTCCTTTAAGAGGGTAGAGAGGGAAATTTGTAGACCAAAAGCCCTTGCTTAATCTCCATGAGTATCTCCCATGGACTTGTTCACCAAATATAACTTGAACAGACTGTAAATGTACCACACCAACCttctctcattatttaaaaaccagCTGTCATATATGGGGTTTGAAATTTGCCTGTTTAGTGTTTCTATTGTTCTTCACTCACAATATTCtgatctgtaaaaaaaaaatccttaatggTCAAGgtgacctttttttaaaaaaaagctcttaaTATCATAAACACTaagctttttgtttaattttgtgttACTCTGAAAAGAGGCTAGTGTTAATTAGAAAGCttaaataatttgcatttggGGTGGGGCTGAAGAATTGTCCAatcaattcttttgtttttgatgtattaCAATCAATTCTTGATTGTTCTAACAGCAAGGCATGACCATTTAAAGGTACTCAATGCATGTTAGTTCCACTCCCCAAAACAAAAGGTAGTCCAAAAGCCTTCTGTATTTatgcttgaaaaaataaatactagtgtctttgttttcatttacccAGGAGGTAGACACTTCTAACGAGTTCTGCCTAGGTTTAGTTTCCAGATGATTTGGGGTTCTCAATGTGTAGTCTGTGGGATAAGGAAATGAAGTGACTCTGGCATGTACATGTCAGAGGCGGCAACGGTAGATTCTGAAAGAATCCACCAAGTGGGAAATCCAAATGCATATGCAGGCAATTAAGAGGTGACTCTGTTTGTTCTGGAAATGTCTCTCCTGGAATTGAGCGCACAAGCTCCTGCAGCCGCCTGTCTCCTGCCGATGTTAAATGTCACCCCTCATGCCAcctctttcctctgtcctcaTCAAACAGCTACCCCCCTAAGGCTAGAATCTGAATGAGTGTCAGAAATTCCTGACCTTGGCAAGTCACATTTTCCTTAGTCCCACTCCCCAAGGCACCACAGAACTCTCAGACTTTTGACCTTGCTGAAAACCCTGGGATTATTTGAACGAGAAATTCTCTTCCCcacttctcttcttccccttccgTTCCCCCTTTTCCAGGGCCGCCCGTGGctccatttaaagaaaagctGGATTATAAAACGCAGAGCTGCCCGCTTTTGCCCTCTTAGGTTTTCGGTCCTCTAAGCCAGCGTTTTTTCCAAGCTGCTGCATTCTCCACTCACCagaccatatttttaaaaactatctggAATACAAACAGCAACTTGTTTTTTGTACCCTTGACTAAAAAATCtaccaggctttttttttaagtgtaatgttgttacaaatcatttttaagtgaGTTTAATGCTGGTGAAAGGTGCCCAGATTGACAGCCTGGAGGCGGAGGGTCTGTATTTTTAACTCTAACGGAGTGAAGCAGTGTTAGCTCCTGCCAAGGAAATTCACAGGCCTCACCCTTCTGCTCGGAAGGGTTCTTGTGGGATTGAACAGGTACAAGTCCATTTGAAATGTGCTTGCTCAGTATTTCTATTGTTCTTAACTCACAATATTCtgacttgaaaagaaaattatatatccAAGTCCCGTTCAAAtgctgcctcttccaggaagcctttcgTCATATATCCCTGCTAGTTTGAAAAATCTACCTTCTGCCTATTACCCTCCTGTATAATAGACTTTTATAGTAGTGTTGATCAGACCTCAAACCGTATACATCTTTTCTGCCATGCTGTGCCCCTCAAGACCCcatgataaaatgagaaaaatcaggcattctcattccattttataggtgggtAATAGAGGCAGAATTGATCTACTGATATCTAGATATTCTGATCCCAAATTCTTTATTCTTCATCTactaaaacaagtattttttaaacttcctagTCTGGATGAAAGCACTGTCATTCCCCAATCATCTTCCCGCTAAATGTGTCTTGCAGATTGCCTTCATAAATCCTGTGGCTTGGAGTCAGCCTTCTGAGGGCTGCTGAGCCATGCCCTAGTCAGCTACTTGTCCAGACCTGACTCACTCTTTGACAGGACTATCCCAAGCCTGCGCTGACTCTGAACGGCCttcccaggctgcaggctgcccTCCGTGTCCCCCTGCATCGGGAGATGCGCAGGGCTGCTTGTTCTAGCCACAATGATTTATCCTGACCCAgtttcttctgctccttttttcAGATCATTAATAAAGATATTAGTAGAAgctcagaagaagagagaagcctAATAAAACCTCAAGTAAGCCCTGTTGTAAAGCCAGTATCTCAGCCTCAggaaaaatattgtttaaattacCTGTGCTAAGAAATTAATAAACTGTACCTGAATCCTAGTCTCGAAGTCAGGGGACTGTCATCTTGTTTCAAAGTTTGGTTTATATGaaatccagtttttcttttcaaatactgAATTTCTCTGCGTGGCCTGCTTAATCGATTACATACTTGTAAGTTTATGCTGACTGTGACTTTATCACATTTGCCAAATAACTTGAAACATCACGAGAAGAGTTAGAAGGTTTAGGAGGCTGAGGAAGATGAGATCATGAGGCATTTCTTAACTCTCACGGTGCTCCAAGCTGCCCAGTGCTTAAGTATTGGGGGTTCAAAATCATCCTGAAAAGCAAGCTGTGCCTATTCCCAGTTTCTTCTGAGTGTTCTCCAACAGCTTCTTACAGGTTTGAAATTCTTCCTCCAGTCTGGAATCACTTCTGAGTCAGACAAAATTCTGTCTGTAAAGATCTACAAGGAAAGATCTGATCAAGCCCCTGTGAGACAAATGTCTGCCCCGAGAAACAGGCATGTAGGATGGAAGACAGGAAGGGGCTACGGTTGCTGTTGAGATTTTGAGCTGCTTCCACCTGGTAGCTGAGGTACCATTGAGTGatgaggaaaagaggaggaacaGGTATGCAGGATTGGTGATCTCAGGATTGATCATGTTACAATTACACTGAAGGTGAGCTAGGGGAGGGCCAGGGTTAGATTCACCTCACCGATTAGATGATTGTCTCTTCCAGCAAAAGCCCGGGTCATATGTTTCTTTAGTATCCTCcaaagtgcttggcacagtgcTGAACCCAGTGAATACTTATTAGACCAATGGCCATGGTAGGTAAAATGCTCTGACTTCAGCTCATCTGAATGATGCGTACTGTTTCACTGTTTGGAGACAAGACCTCACGGACTTCTGACCTTTGGCTGCGTTAAAGCATACACACCGAAGGGGAGTGACTCCAGCCTAGGAAATACTGATAACTGCCCAAGGCATTTCAGTTACACAAGTCCTCACGTAACTGAATTGTGGTCGTTAGCAGTACCGCCTTCTGGTGTTTTGCTAAATTACAAAGAGGTTGCTTTTGTAGAACTTAAAACGGAACCCTTCCTTTCTGGGAAAATTACAGGCAGTACCTGAATTACAGGCTCTAACTTTTCAAAtaacaaactcaaaaaaaaaattgctcctATTTCACACCCCACTGCACCCAACCACCCCCTCTTCTTTGTCAACTGCTTCTAGGGAGCCCTCTGTGAAAAGGTTCACAAGTTTGAGTGAAAACCTAGgtaaggaaatacagaaaagatgaGGCTGAGAAAAGGAATACCTGGGGTCAGTGCTCCAGGTGAGGCCTCTTCTATCTACTAAGCCAGCCGGCTCCTGTCAGCCTCCCGGGCCCCTAGAAGAAGGACAGTCTGGGTGCCCACCACCACTGCTCAGCTTGTCATGCAGATGGCTGGTTCCAAACACCATCATTTCTCATATGGCCCAatgcttctcctttctcctctaaTACCTATTTTCCATATAGAAGTCATGTTGTTCTTGTAAATATATCAATCAGATTACAACCCTCCCCCCAGCTCAAACCTCGCCAGTGGCTTCCCACAGCCATCACACTTAGAATATAAGCCCAGCTCTTCCCTATGACCTACAAGGTCACTATCACCTGGCACCTACCTGTGTCTGTGATCTCATCTGCCAAACTCCCATCACTTGCTATCTTACAGTCACACTgactttctttctgttccttggaTCTATCAAGCTTgttcctgccacagggcctttgcacttgtggTTCATTCCCACCACCTGAGAGGCTCTTTACTCTGATCTCCTCAAGGTTAACtccttcttttaatttatatcCTTGCTTAATAatcctgtttcatttcctttataggacttatctttatttataattgaatcatttatctagttgtttgtttaattacatatgtattgCCTTTCTTTCCCAGGAGAATGTAAGGTTTATGAGGAGAAacaatttgtctttttcactgCTGAATCCCCAAAACCTAGAATAGTGGTGGGCACACAGCAGGTATTCAATACATAGTTGTCAAACATGGGAAaatgagtgagagaaagagaaaaacagtggaAGGGAAGTCTGTTGTGCAGTCATGACAGACTTAAACCTAGGCCTCTAACCTTGTCTTATTGGCAAATCTCAGAAGAAGGGGCCTTGGGATGCTCAGGGTTTACTGAGTGTTTGCCCAGATGATGACAATTCAGTATATTATTCCCCATTAAACTTCTCTTCTATGTGTTTATCTTCCTCAGTAAGAACTGTAGAGAAGAGGGGTGAGCAGGATAATCGAGGCACACAAAACCTCAGTGGTATTCTTAAAGAAATTGGGCAATGTGACCATGCCATAAATGATGTGAGTGCACCAGGAGAGAAGTGCTGAGTTCAGACCTTGGTGGGGCAGTGGGTGTTCAAGAAGGCTGCCCAGAGACTAAATTCATCCAGTTCTAATTTTGCCCAGCACTGGTCCTATACCAGCGAAGAGGGAAAGGCATCCCTTTCCTCCTTACTGTTGAGTCACTAAGACCCTTCCAGGAGGCAGCTTTCCAGCTGCCTCCATGGTCACTACTGTTCATCTGTGTCATATATTTACTAGTCTTTTGGAGGCTTCCCGGGAACCTAACCACCACTTATAACATTGCATCTGGAAAAATGAATTTCCGATTCCAAACAACCAAATCAGACTGACTCTTTCAAATAAAATCCTATTCTTCActataataaaatcattttcagtgggagagggcatagctcagtggtagagtgcatgcctagcatgcatgaggtcctgggttccaccccctaGGAATGAATATAGCCCAAAATTTTGAGCCTGTTTTGtttcaatatattttgtttttggctgggggaggtaattaggtttatttatttatttttagaggaagttcTGGGGAatgaaccgaggaccttgtgcttgctaagcatgtgctttaccacttgagctataacctccttgctttgtttcagtattttaatcCAGTGTTAGACCTGATACAGTCTTCAATGGACTTGTAAAAAATATACTGCTATTCTTCCCACAGCTCAGAGGGGCTGGGTGCCACGTTCAAGGTCAGAGCCATGATCAAAATCAACTCCTCATCTTCAGGAAAGCTCTCTGGCTCACCTAATCTCTTCTCTAGTGCCGTCTTCCAACTCACCCCTTTAGTCAAGTTCACTGAGCCCTGTAATTAACCTCAACTTTGCTGCTTAACTGTCATTAAATTATCCAGTGCCATGAGGGGTTTTCTTTGCAAAGATGTAATTTTCTGAAagactatttcctttttttttaatttgtaagccTAATACACAGTCCAATACCACAGTTACAGGAGTCCTGTGTTCCACTGATTAAACGTCACAGAAAAGCCATTCAACTCAATCGTCTTCTTTCCAATTTTCTAGCCACTGGAGGATAAGTGATAGGATAATGGAGTATGGGGTGCCCCGGGGGTCAAAACAAGTTCTGGTTTTGTCAGGATTAACTTTGAGGCTTCAGGCAAGCTGCTctgctctctctgagcctcaaagcCCTATTTGTAAATTAGGCATGACAATATCTACCACTCAGCATTACTGTGAAAATTAAACGTAAATCACCCAGTACAGTACCTCGTCATGGTGCGTGCTcaataaatcttcatttctttccctcaaCCCCCTTCCTCCATTGAAAGACTCAAAGGCGCTAGATGAGTGGGGTCATCTCTGAGCCCAAACACAAAGTAACATGGAAACTAATCCAAATTCCCTCTGCTAGCCAAGGAACCTTCTCTGTTATCTCATTGATACATTTTACATCCTTATGGGTTAATCAGAATAGAACCGCATTTTACAGTTACACTACAGAGGTCAGTATCACGTAGATGATTATTTTTCTATGGAAGCATTATGCTTGCTGCTTAGAAAACTGTATTTAAACCAGAAGCCCAGATGTCTTTGGTAACTGTTTCCCCCAAAGGAAAACTCATTGAGAAGGTGAGTCAGCAGGGATAACCTTCAGGACAATCACAACCAAGGCGAGCATCTGCAAGATCTGAAATATGGGATGTTATGACACTTGGTGAATTGCTTCTGACGTACTTAATTATGCAACAATGAGATTTGCAGGAGCATAACCACACAGTTCCCCTGAGGATGCCGCCAGTGTGCTCCACAAGTGATTccgtataaaataaacaacagctaACTGTGAATTATGAAGTTGTAATTCCACTAAGGGGGTTCCAGTGATGGCATCAACCACCAGAACAAAGTTTATGTCACCAAAGGATCCCAGATGGACTTAGGGGCCTGTAACTCAGCCGTGGTCTCTGCTTCAGATAGAGCTCCAGGACAGCCTTTCCCCTGGGTTTCTGCCCTTCCTCGTCTCTGCAAAGGCCCTCCTTTATTGTTCTCTTCCTCAGAGCTTTCTAAAGTCAGGCTAAGCCTGCTTTTCTAAATGTagctcttttttaattaaatggtcTGTCATCCAAGAGGCTATCCACATCGCCCCACACCCTCTGTGGACAGCAATCTATGAATTAGAGATACTTCATTAGAAAAAAGCAAAGCCtgatatttaagagaaaaaatccATTTAGGGCAAAAGCCCTGGAGCGCACCTTAAAAGGAGATATTTGAGACGAAACTTTAGTACGTGCCAAGCGAATGTATGTGCCCACTAATGGAATAAATTTGTCTCTAAACATGGAAAACTTGAAAAAGTTATgctgaaatttcaaaatacaacCTTCCCAAAGTCAACGTCTGAGAAAAAAAACTGGTATATTAAACCTTAGCTTTTCTCAGATCATATAGAAGGTAAGTACACGAAACTGCATCAATATTAGAAAAACTGTATCAACCATCAGAAGGAAAGACAATACAAAACACTGAACTGTAACTTCCCAAACTTACCTAAAACTAAAGTGAAATTGCAAATCTCAGTATCTGTGTGCTAGACGGCCCCTTCTTTAATTCATGCTTTTTCTTCTAGAGGAGCTGAGATAATAATCACCGAAAATTCATCATAGCGAGGATTTTTACAACCCCCCCAAATTGCcttaaactgtttttgttttgtgtgaaaCACTAAAAACAGTGCCAGGCACCCAGGGAGTgcttttctgtgtgcttttttaaATCTCCCACTTGTTCTTCcgtgcattcatttattcaataacgGCTTTTTTATTAAGCCACCTACAATATGAAAAGCACCGTGATAAACGCTCTGAAGTTACACAAtttctgcctctctgtccttGCCTTCAAACTTCAGACACCTGGTTGAAAAGATAAGCTTGACCCTTAGGGAGACTTAAATAACAATGGAACACACGAGACACAGTGGATGCCACAAAGAGACACGTCATTCAGTTCCGATTACTGTGAGAGAAAGTGGTGCACATCAGTGTTTTCAAATCTTGCGGCACATAGACATCATCTGGGATACCTAAAATGCACATATTTAGGAGATTCGGATTCCCTTGGTGTGGGCGGGGCTCTGGAAGCTGCATTTGAAGACACCGCTGTTCTTTCTGATGCGGGTGAGCCAAGCATCCCGCTTTAAGATATTCTGGATGAGGCTGTAAGCAGCTCAGAGAAAGAATCAGATTATCTGttatttctctttatccttttttctctAAATCATTGGGAATGAACTGCATGTGGGAGAAACATatacaggagaaagaaaggaagaatataaGGACCAAGGGAAAATTTTCCTAGATGAGGAAGAACTCCATGCTCTCTAAAGGAACACAGTAACCCAGTTATCTACACCCCCAGTTAGAGACTCTACATTACACTGTAAAGTGTGCACACAGCATTTGTAGCAAACACACACTTTTCCATACATCAGAACTGATACTCCCCACAGAGCTGTGCTCCTAACCTCTGACCGATGCCCACCGAGCATATTTAGGAGATGGTCTGTCTCTCATCTACCACATGCATAAACTGCTTCTCCAGACTGCAGGCGCACTAGACCTGCAATAAACTGCTGTCAGAGTGCCTCAGCCACAGTGACCCGTTTTCTCCATGTGCACCACAAGCGTTAGCCATGCTGATCTGTTACCAAAGAATGCCAGAGAGACCTAATCGTTAACTGCttggcaaaaaaaattaaagagtccTGGATGCTTCATGATACAGAAATGCAGTCTGTATGTCTTCCAGGCTTATCAGATTTCAAAGCCATACATACAAAAGCAGGTCACGTATCAAACTGAAAACAGCATGTTTATCTTACTCAACTGGTTAGGAAATCTAAGTTGGCAAAAGAGTGGCAACTTTATGTATAATCATAGTTAACATGGTGAGTATtattcctgtaaaaaaaaaattaaataaactccACAGAAATCTCAGAGGAGTGGTAAGACAGTGCTATGTTAGAGACATTGTACCACAGAGAAAATTTAATGTGTCTGATCTCCTGGAAACTTACAGGCTATGTCGGGTCTGGGATCAGAAGTCTTGACACCTGGTATCTTAAGCGTATGTCGTATATTCTTCGTTAAATTTGGTGTTAAAATTAGAGGAGGGTGTTTGTATTCTTCAGTCTCTGTAAAGTCCGCAGTGATGGATGACGCACATGGAAATATGAAAGGTTGGTGCCAttccaaagttttaaaagatttgcAATCTACTTCTTACTAAAGAAGGGGAGGGTGGCCTGAAGGAGAGGGCCGTAGTCAGGAATGTCTGAGCCAGactcctggctctgctgggcaCTGAGGGTGTGGTCTTAGGAGTGTCACCAAAAATTCTAACTTCTCAGTTCCTAACTGTAGAGCAAAAACGGATGTCTCCTTAGTCACTCCGTAAGCGACATGCTGTTACGACTGTGATTTTAGAGTATGGGAATATTCCTAATTTCAGGGACATTAAATGGCCTTAGCAATGGATTGGGAAACCACACTGAGTATGTAACTGAGCCTGAACTTGTCAGAGATGCTGTCGCAGTGGAGAACAGGGAAAGGGCGAGCATCCAGTGGGAAGGGAAGTAAGGTGTTGGGGTCTTTTTTACATCATCTCTTTATTCCAAAGAACAAACCTGTGTGACAGATACTAATGCACCAGAATTTTTACATGAGAAAATCAAGACCCAGAGGTTTGTCTAAAGTAAATTATAGAACTGTCATTGAACATGGAATTGTCCAAAAACAAAGTAAATCGCTTCGAAGCTCCTAAGGTCCCtaaatctaaattaaatattcatgCTTTTGAGAGCTGGGTATAAATTTattgaaatgcatttttgtttttgatctttatttgataatttaattTAGAAGAGTCTCCATTTGATTTCCGTCACGTGCTGTTAACGTGCCCGGTTTATAACAAAGAGCAAGAGCCAGAGGAATGAGAAGAGATTTGTGCAGAATGTCAATGTGTCAAGAAGTGGTGTGCTGGTGTCCCTATTCCTAACTCAGTATTGATTCACTGGAAATCATGTATATGAAAGAATCTGGTTGACATCAAAGGTGATAggatacagtgaaaaaaattaaaatggaatcagGAGAGAAAGATTTGAGTCCCTCATCAGCAGCACACTAAAATACTCCAAGCcacagttttcttatctgtaaaataaaaataattatgctatCCCCACCTACTCCTTAGAATTGGCTTTGTGAAACGTAATAGCATTGGCAAGTGTTCTTAGAGCTTGTGCAGCAGTTTCATTGTTCTATCTCGCTTTATTGTtcacattattaatattattaatccTTTAAGACAACATAAGTCATCAATTTATACAAGTAATAAGGGAAAAGAATTAAGCAAACCAGCAGTCTGAGTATATTAATTCAGTTCAGTTAAAACGATTGTACTTTTAAAGCCTACCCAAACACAGCTGGAACTTCCTAACTGTTCAAGGCAGCCCTGAATCTCCAAACCAAAAACATCCAAAACAGTGCCGACAAAAGGTATTCTTCCCACCCTGAGAAAGGAGAATGTTGGGGTGAAgcaaaaaagatataaatttaaagaaCACTCCATATATTCTGAGTAAAAATGATCACACGCGGAAAAATCTCTTCCTCCCTGAGGGCAGCTGAGAACAGACTCGTAACAGTCAAACACGCAGagacattattttctgtttctcttgtcaGAAGACATTCAGCCTTTGAAATATGCAAAGCGAAACTGCAGATTTCAGTTTCGTGATTAATGAACCAAAttgggttttatttcttaaatgtgttGAGGATTCATCCTGTGGTCTTCCCTCGAACTTGAGGATTAGAGCTGCTGAACAGCACATCTGGTTGTAGGCAAAGAGCTCTCTGGGTGGTTCCCCGTCTCTCCGCCCTGAGACGAGGCAACGTGGGGGAACTGCAGAAACTTTTACTGGGCAAGCGTGGCTTTGCATCCTCAAACATGATGATTATGCTCTCCGTTCCCTCTTTCTCATGAGAAGGGGAAAGATTGTTTGGCTGTatctaattcatattttttaaaattttgttcagcaTGATTTTATATCACTTGCTAATACTTTCCctaaggaaaatatttgcattccTCAGGCAAATGTTTTCACGTCAGCTTGCTGAGGAATGATTTTTACATAGACTTCAtaataacaatttatattttctctgtaattatcactttaaataaGGGCTAGGGGCCTGTGGACTTTCATATCTGGATAAATGcattatctttgatttttcttaagtGCTTCTTCCTCTGTCAAAATACATGACAGTGTCCTGGAATATGTCACTGTCCGACCTGATCATCGTTCTACAAGGTATAGAGAAAGAAACGTcgtttttctcctcctttttctacAAAAACACATGTGAGTCTTgaaacatttaacaaaagaaaattcaagatcCAGAGATCTACACTAGTAGAAAATTTTCAGTCCATTCCCTTAAGAAAGTTTCTCCCCCACTTGCTTTGTGGTGCTTTTCCTTACtaaattatgtaaaaacaaattttttgagCAGTAGCTCTCTCCAACTGTGGTCTCGGAACGTCCTTTAGGTATTATGCAGGCTTTTCCAATTTTCAAAAGGAGACATGTGCTCATGTCTAGGGAAGGTTCCCAGCTAAGaacaagcagaaaagaaaaagaaactgtttaCTCTGAAGGCCTTTACTGCTGGAAATGTACCCATTGGAACTTGAGAACACTGGGAAATAGCTGTGATGTCACAGCTCTCATCATCCTTGGGgaacttttgtttgttgtttgtgtgtttaattttctgatgAGAGCAAGTGGGTTCaatgtgtttatgttttcttaataTGGGGGAAACAcagtctttgaaaaattaaagaaaaatcagtcatCAAGAATGTTAatagaaactgtaaaaaaaaaaaaaaaaaaagaaatgctttaagACCAGACTGTTCAGGGTGGTGAAATATAGGTGGCTTGACTGTcccacttaaattttttaaatgtaaaataatgcattttctaaaatgagcaCTTATTACACTTTGGTATtactcttatatttaaaaaattacttgcaCTAGAGATTGCATAAACAGTGGGACCACACAATAACCCACCCCCCCATTatctatttaatttctttgcttaGTGCTACTGTGGAATATGCTACGCTCCCCCCACCGAAGTATCTAATCTTAAGAAAATTCTCAAAGTAAATtaacaatgataaaatatttagaaaatcattAAGCAATACTTGTAGAGGCACTGATTATTTCTACAGTAAGCATGAGAAAAGGCATTTGCTTGGTCTGAAAGAAGCAGTCAAGGAAGGCATCAcgaggaggtgagagaggagcAGAACTTGGAAGGTAGCATGAGTGGGTCTGCGGAAAGTGCCACGGAGCAAGGACACTGCAGGGCAGAAGGCAGAGAAGTGGAGGAGAGACTGGTGGGTGCACGGGTACTTGCCTGAGTAGCAcactggggagaggtggggaaacAGCTCAAGTCTGCACATGTGTGTGAACGAGCTAACTCCTGGGGTAGGGGTGGAAATATAGAGTAtaaagaatgacttttttttaaaaagaggacaaaaaattGGTCTTGggatggaaaagaatgaaaagcaacCGTGAGTTCTAAAGAAAGGGAgagccatgattttttttttaaatgtgtttctaaaAGAATGCACTagcatttactcattcatttggcaaatattccTTGAAGATGTATTATATCCACTCTGCCATCACCTCCGCAGAGAGGAGGACAAGCATGGATGCAATTAGACCCAATTAGTTAGGAGGCTGGCTTCGCAGTGCAGGTGAGATACCATAGAGATTTATAAAGGAGATGAATTCAAGTGATTTTAGAAGGTCAACTTGTTGGGATTTGGAGATGAATTGAACAGAGGCATAAAGGAAAGAGTCAAGGATGCGTCTGAAATTTCTGAGTTTTGCACTCACATGTTGTGATATGGGAAAATAGAGAAAGCTAAAGGAGACATATGGAGGAAAAGGAACTCATTAAAGCAATATTTTGACTGGCTTTGAGGTTGAGGCACTTTTGTGATGTTCGAATAGAAATGTCGAGTAGGTAGCTACATAAATGAACCTGCAGCTCCAAGCGGGAGCCTTGAAGCTACAGCCTGAGAATCAGTATAGAGATGGCTCTAAAATGGTGAGCCTAGAGGATCTCTCCAAGGCAAAGGGTTGAAAGTGAGAAGGGGAGATGAGGGGGCCTAGTACTTGCACTAGGTTGGAAAGGGGATCCACAAAAATCGGGGAGCTCATTCTATCACTCAAGAAGCACTTACTGAGCACAGACAATGGTTTGGCAGCTATGCCAGTGGTACGTGCCTGCTGTCACCCAGACAAGTACTGAGGGAAGTAGCACTggtagaaaatggaaagaaaagtgaCAATGTATATAATTATCTAATGTCCCCTCCCAGTCTGGATTAATGGTGATGAATGTCAGTGCCCAGGTATAAAGATTTAGATTCAATGCCTATTTCCATTGAAACTTTCAACGTCATCTGTTTATGATTacttgaaagagaaacagaatagtAACCAGTTCCCCAAAACTCAATCCCTAAGAAGTCTCTAACTGGgctattataaaatgaattatcaCAGAGGACGAATACATGAAAATACCTAGTACATTACGTATCATTTTGCTCAACAGCACTCCCTCTTCTGAGGATTCTAGAACAGCATTTAGGAGGATTCTGTGGCCTCCAGGCACCAGTTTTCCCTGCAGCTCCCAGGTCTTGGACACAGGGGCACAGGGAATgaatgcaaagaaggaaaagtatGGCAGACGAGGTCAGGCCGAGACCCTGAATCCCCACCACCCATCTGGCCCGAGCCAATTCTACCAAGCCCTCTGTAGACTAGCATGAATGTGTGTGGATAAATACAACAGAAACTTGAGAAAAACCTGGTTCTCTGTATATATCCAAACTGCTGGCTCGGCCTTTCACTTTGCCCATAGTCAAGCTGATTGAACTTAGCATATCCTGGCTCAAATTTTGATGGAGGTGGGCCTCTTGGCCTTTAAGTTGTATATCTCAATCGTCTTTGAAAAGGTTTGGCCTCCCCACGTATATGCAGAGTACTGGATGTGCACCCATCTGTTTTTATGCTTATTTATACTCTACTACCTTCCAGCGCTGTGAGGTCCTTTAAAATATAACGACATGACTGATGATGAAAAATTAAGTGAATGGAGTGAATATTCTTACTCTCAGACAAACGTTTGTGGGAAAGCAATTTCCCTTACAGTTCTGGCTTCATGCTCCTGTTTTAAGTGAACACCCAAGCTCAGATGATCTTGGAAGGTATGTATTAACCACCATCAATGCTAAGACGACAAAATATGTGTTCTGTCAGTTTGTTGCTCCCAGTATTGCCTTGTCCTGTTGACCCTCTAAGGTCAGGGTGTGAAATGGACcaggagagaaatagaaaatcccaaggttttcaaggcacagggcagagggaggattAAGAATGTAGCCTCATCTGTCCTGTAAGTCTCTGTAAGTCCCAGGTTTCAGGCACACACAAGACTGTGGGGGCAActactgtttttcctttgagaGAAAGCAGTTTACCCTAACCCCAGGGCCTAACTCTAAGGTGGCCGATGTTTTGCCCTATCTAATGGTTCCAGGTACCAATGTGGGTTTCTGGAGCACTTTTGCAGCATCTCACTACTACCGCCAGCTCTCCATCTCTAAATCTGCCCTTCCTTCTTTAAATACCTGGAGATCATACACGTAAATGTCTCACACCGAACTCCACAGGGACAAACTGTCTCCTTCCCCATGGAACCCAGGATCCAGCCCTAGCACCCAGTGACTCAGGGTCATCTAGTCCCACTCTCCTCCTGTCCCTTCCTACTAACCCTGGAATCCTGACCACTCTCATTTCACACAAAGCTGTGTGGCATTATGTGGTCCCTGGAGTGACTTCACGCTATTGACATTTGCCAATTTTTGCTGCTTGTGTAGAAGGGTCATCCTGCAtgtctccctctcctgcctgAGTCCAAGGGTTTTTGAAGGCAGGCTTAATTAAGCCCCCTGCCTGGGAGGGGAATGCGCCCCCTTTCGGCGGGAGAAGGCAAGGAGACGCGCGTCGGGCAAGACTCGTGGGTACGTTCCAGACTCCGCCCGGGAGCTGCGTCCCAGCGCCCTGGGACCTGGGAGGACCAGGGCAGAGGTGGTGTGAGGCTGTTTGCATCTTTGTCCCGGCGGCAGAGATGGAGAACACTAAAATCTAAGATACTGTGGGGtctgttttacaaattaaaattcttCACTGAATTACTGAATCAATTCTCCCTCGAACTATTTACTCCTTCCTCCAGGTCCCCCCGCCCCCGCAAAAAGAGGTAAGATTCATAGACAGCCTGCAGTTCAGGCCAGTGCGATCGCCATTCCTGTATGAGGGTTTCGGTATAGTCTCTCCTCGCTCAGCCATTAAGAACATTTCCAGTTGCTTGCAAATTAACTCCGTGAAAGCAGACAAAATAGGAACCAGAGGACCTGTCCTTGTCCTTTCTCATAACTCGGATCTTGGAGGTTCTAATTAATACACCCTTTTCAAATGCAGTCCTCATTTTGAGCTGAGTCTGGAAAAATCAATAATGGCAAAAATAAGGCTCAAAGTCAAAGTCCTTGGGAATTAGAGCTCCCTCATTACGTGGTACAGCAAAGGGGCTGGGACATCCAGTGCTCCAGGACCGCATTCTGGTCATAACTCGGACCGATATAAATTCCTGTATTTGAGAACTGCGGGTACAGCAGAATCCTTGATTCTGATTGTCCTCAGACTCTTCTGAGAAGTCACCTGTGTATGTGATTGATTTGTTCTgacatgtttttttaattttctttcacaaaatttcCTTAACCTACCACCATTTGTGCCTTCgtgcccctcccatcccctcaaGTGGCTATTGCCCCTGTAATCGGCTTCCCAGATATTTTTCAATCAAGGAAAGTGGTTCCAGGACAAGTGAAacgtagtgatttttttttggtcttaaacCTAAAACTGATAGCCCATAATTCTCTGTAGAACTACTTCCTTGAGACTGCCTCACTAAATTAGACAAATGATGATGCCAAGAATCTACTAATATTCAGACCACGGTGGAGACTCCTTACTCAAAAGATTACATACGTGGAAAACAAACCCCTCGAGCCTGAAACACCGACGATTGTTCCTCTTCAGATATCTTGGCTATATATCTCCGCATTGTTTGATTTAATAACAAACAGCACTTAGGGAAAAGGAGGTTGTGGCAGTTTGTTGATATTATTGTTACCGTTGGAGATAGTTGGTTTTTAATTGAGTGTTGTTAGCCTGCTCACCAAGCTATTTGATTTTCAAGAATCCAAATTCTTGTGGATAGATAATAATGCTGTTGACAGATGCACCTTCTTTGGATTGTTGAAAAGGGCGTTGAAGGAAACCAAAACAACGGGAGTGGTACTGGCCCCGTATCgaattaaaatgcagaatttgaCAAAGAGCGTCCAATTGGATTACATTAGCATGCAATCCCAGGTAGTTATGTAAATGTTTGGACACCCAGTGGCAACAGAGAGTTGGCTCCTTATCCAAATGTTTCCTGTAAATATCGGATCTCTAGCTCGGAGCAGCACTCCGAGGATCCTGTTGGTTGAACTTAGAAATGGCTTGGATGGTGGATGCTGAATACCTATGTATTTTCTTCGGGAGGATTCTTCCCCCCAAAACTCTTCTACCcactgcaagagaaaaaaaagcaagcaaaacgCAAGCCTTATCTCCTCTATATATACACTCCCCTGATACCTAAAAGTTGTACTTCCTATGGCTTCTGCTGACTCCATTTACCTCCTaaagggtaaaaaataaatacatattctcaGAGCCATTTAAAGCTCGAACCGCCAGGAGCGAAAGGTGCTCAGGAGTCTCCGGGAACGTACAGGCGCGCACAATTCTGCAACTTCCCTGTTCCTGAGGCCCCGGGGCCCCTGAGAAGGAGAGAGGTTCTCCAGGAGTAAGAAGAGGTCTGCTTTGATGGGTTGCCCTGACGAAGGAGGGAGCCGGTAAACAGGATATAGGAGAGGAACTGCAAAAGAAACacatcttctaaaataaaaatcaaattggtGTAGGGCTCCTCACCGTTGAAAATCTATAGATGCCATTTAAAAGTGAATAAGCACCCGGAGGTGGCTGCTGGCCAGAAACGAAGTTCAAAATTCGGATCTGACCGGATCTGAGCCAGTGGACTAGGAAGATATCTTTGGCTCCGAAATTGCTGGGGTTTTTTTCGCAGGGGGCGGGAGGGAGGTGACATCTTGTTGGTTTGGAGATGATTTACTTACTCATGTTGTAAAATCTAAAGCGACGTTCCTGGGAAGACCAAGGAACTGCCGGGACCCGTGTGACCTCCTGCTGCCCGCCTGACGCCCCTAGGCTCTGACTTGAGCTTGCCGGGAAGAAGGTTAAGGTCGCCGAGCAGAAATGCCGGGTTGCGCGACGCCTAAGGTGCGGGAAAGGCGCTCCGGAGTCTTGGGAAGGGCATCCCGGCGAAATGAACGGTAAAGCCAGTAACTCACTGTCTTCTAGAGTTGGGGGATCTGGAGGCCCAGCGAGTATTGGCAGTCAGGGCCTCGGACTTTAGAGGCTTACCTCCCGGGCGCTTAAAACTCCAAGCGCCTTTCGCTCCTAATCGCGAGGGAGTCACCAGACTGtaaatttctccaaaatattAAGAGAAGGAAGCCATTCAACCCCTTGCTTCTGTATGTTAAGATAAACAGGATTCACAGCCTCTTCTCTGGACTTGACATTCGACCTTGAGGACTCAGTGGCCTCCCATGATCCTAGATTTCActgcctcctctaaaaagaagaaagaaaaagcaaaaaaccctcCAGGCTACGCTTAAAACGGTATATTTCATTAGCTCCCCTTTGAATCGTGGTAACTGAGGCCCAAATTCAGAGAGGCAGCACCGGCTCCAAGGCCCCACGGGGTGGTGCCGCATAAGAAAATTTCTGGTGCCGTAAGGACTGCAAATGCTGAGACGCCAGCAGAATTGATATTCCCGTGCGAGTTCCACGCGGGAGCGCCCTGGAGAACGTTCCCCGCTCTCCTGAGCGTGTCTCTGGCCTAGAGAAGTCGGCGGAGATGGGCCCTGGGGAGTGTGGCAGGCGGCTGGGGCGGGTGAGTCCTTCGCGCCGCGTTCACACCTCTCCCTTTGTGACAAATCGATTTCCCGCGACGCTGTTCTGTATTTGCATCCCGCCGCGACCCGGGGCCCAGCTGCGCCCGGTCCACACTTAGCACCGCCTTGtgaatgtgtgcgtgtgtgcgcgcgtgtcTAATTGACTTTCCGTCTACCTTTTTATGACTACATGTGTTTCCAACAAAAGGTCTTATTAGGACAATCTGGTTTCACTAATTTAGAGGTTGGGAACATGGAaggcaggcttttttttttttttttaagagtttgcaGAAGTTCAGATTAAGAAA |
|  | TGACAGCGGGATGCACCATCCTCCCAGGAGTGGGTCTGCCTATTTTTTATGAgttgtttctccttcctccctgactaCCCCTGTTCTGCCACCACCCCACTTCACACTCCACACTCCCCCATTAGCCATTGGATTCCTAACCGTGTTTAAGGAACGTTTCTTTAAACACTTAAGGTATTTGAACCCAGAAGTTTGAGAAGTATACTAGGCTGGGAGTATcagtaaaaggagaaagaaatcctTCAACAGAGAGGAAAGTGGAGTGGATGAAAATCAGGGGGGAAACAGGGGAGTGGGGGGACCATCCAGAAGCCTTCACTTTGGACAGTGATTAGCACCTCTCGGCTACACTGGACCCAGTTTGTAAAAAAATTTGCAATATTAATGATTAATATTGCCAGTGTAAATAgagtctgtttttcttatttcagctGAACAGAAATCTTAGGAAGGATGTGTTTCTGTATAGCTCTAAGAGGAAAGGGTGTCTGTTAAATGTCCCCCGTCTCTCCAAAGGAGGTTATGGTTTCTCACTCTGGAGTAGTAGACTCCCAGGAGGCTGCAAGGGGCGTCTGACTCCCGATGCCCTCCTCTGTCCCATGCAGCTCGAAGCCCAGCTCTAGTCCTCTGCCTGGAGACTCCTTTCTTCCCTAATTCACAGTCTGAGCCATTCTGTCCACTGCAGACAGGGAGGCCTAGTGCCACCCCACCGGTCCTGAATTCCACGTAGCTCATGACAGCCCTCTTGGCAACTTCTGGTGGGGGCCCAAGACTCAGGACAGGTGTCCTCTTGGACACTTGCTGTTACCCATCTTACCCTGCACTTAGTCTCATcccagagatggggaaggaggttGAGGAAACATTGCTTTGAAAAGCAACTACTACTTGTTAGAATAGGCCCCGGCTGTCTTTACAAAGCAAGGCCTGTGGCCTGGCTGTGGGTGTGTTTGGGTGTGTGTTTTCTCAGCTACTTTCCCAGCCCCAGACGTCCATGACAGAGTTTCGGCATTCTGCGACCTTCTTACGAAATTGCCTATCATTCTTACTCAAGCATCctctctctctgaacctcagtttccctctggGGTTCACTCCACACTAGGCCTTAGGTTACCACTTGAAACTTCAAGATGTGTTCATGTATCTAACAGGCAGGTTCCCGCAGATCTCAACCGTTTCCCTTGGGACACAGGTCCCAACTCCAACCGCCACCCTTATGTGTCCCCTCGCCCCCCGTGGATGCCACGTGATCATTGCAGCCTCTCCTGCACCAGGCCTGGGTCTTGCAGGAACCATACCTAGGCCACATCTCCTGGCCTCCGTTTCAGCTTGCGGGTGCTTCCCAGCCAGCCTCCCCGGCCTAGCCTCGCTCTCTACCCACTAGGAGGCCACCAAGAACCCGAGCTTCCTTCTCGTAGCGTCCGCCCAAAGTCAAACGCCAGGAATGTCAGCATCCCTCCAGCCCTGTAGTACGCGGGGCCCAACTCCCGGCCGCCTGGACCCCGGGCTTGCTGCGGCCACCGACCCTGGGCCTGCTGTTCACATTTCCTCCGCTTGCTCTTGAGGCAAAGCAACCCTGGCTGgtctctcctgccccaccctggtcCTCTATGCAGCAGTTTCTTCACGACCCTGTTTTTCTGTCGTGGGTGCAGCCTCTAGTTCTCTGAACAGTGATAAATGTTATGATTAATAATTAGTCATGGAGAAAGAAGAACGGCAGAATTTGCAGAATTAGTGAAGCTGGCTGATGGGTACATGGAGATACGTAATATTAGTCTCCGTGCTCTTGTATGTGTAAATATTCCctagtaaaatgttttaaaataatcatattatttCGCAGTGCATTTTCCTGTAACTAAAAATGTCTACGCGAAAGATTAATTCACGCTGCCGGCTGTGCTGTGCGAGCAGCAAACCGCTGCTACCCACCCC |
| LNC000105 | TTTTATTACACTAATCTCACAATTCTGAAAGGGCGCAGGGCACTTCCTCTAGTGGAAATGAAGCTTCTATAGGTTTTCAGCTCTGCATTCTTCTGTATGTCATTTTGAAGTGTCTCCAAAACAGCCAACAGAGAGTGCGCTTGTACTTTCTACTGCCATGGGAATGTCCCAATGTTACCagttcttcccagagcttctGTGTCTACAGAAACACCAGTGGAAGCATATGTATGGATATCATACTTCAGGGCCTGCTGGGATGATCCCGCAGACCGACCTATGTGTCCTCGTTGCAGTACCCTGTCAGTGAAGTCCAAAATATAGCATCTGCATTTGGGTGGTAATGCTGTAAgaaatgcttcatcttctcatGGTATGGACTTTGACCACTCTTCCTAGTTCTCTCATTCTTGAGTTATGGAATCACGAAGGCAACCACAGAGCTGTTCCCCATCCTCTGCCTCAAACCAGAGTGTAATCCCTATCCAGGTTTGGAATGTAGCAGATCCTAAAtcttttaatactgatttcaaaccCAACGCCCCCTGGAACCCTCAGTCCAGATGCATGATACCTCTGATTGAGCCCCACGTGTGCTCTATTGGCAAAATGTCAAATTGGACCCTGGTCCTGCAGATGCAGTGGATGTGGCCATAGGACATATCGATAAACTCAACCTCACGAGCCAGTGTGTTTGCTGTCTCACTGAGTTCACAGTTTTCTTTGCTCTCTTGAAAGTACCCACCAAATCCCACAACACACTCTAGATCCCTGAGTTGTGCGAGAGCCATCATCCGTAGCCCTATCCCTCTTTTTCCGCTGCCTCTGCTAGCTCAGATTTGGCAGCTCGGATCTTGGTCTCAGATTCAATTGTGGGGATATTTTGAGCtgatttctttgagttctgtcagccaagcttctgctgtgcactcttctaacactcagcACATCACCTTCAATCCCATGCCTGCTGCCCGCTTGAGAGTGTGCTTCCACGGAAAACAGAGTTTCACCTTTGATGCTCCTTTACCAGAGGTCAGATCCTGCACTGGTTTCCATTCTCTactttgccttttcctgcttACGGTTTTTCTGAGCA |
| LNC000948 | TATTCCCTACTACAAACGCACCATCACCGAGAGCCAGGCTTTTCTGCCCAGAGTACAACTGGAGGCAGCGGCCAAGACACACTTACCTCCCGAGAAAAGACAGTGACGGGCTGTGACGTTTACCACACCCAGGAAGACTCCAAGTTGCACAAAGCACACCGTACAAAGCTGGAGGGAGGGCTTTGCCCCTTTTCCAGTTACCAGGATCTGAATGGACATTCTAAACAGGAGAATCACCTGGCCTGAGCCGGGAAACCAGTCAAAAGAACGACGACACAGAAATCCGTCTCAGTGACCCAGGAGCAACCCCAGGTGCAGGAGGTCAGGAAGGAGTCAGCCGAGCCAGGAGAAGTCAGTGTGGAGCGAGGGCTCACCCTCCTCACGCTGTTTCGAGGAACCCACAGTCAAAGTCTGCGGGTCCCGGCTCCTAAGCACTCCC |
| LNC002464 | GCTTTGGGGAAGGGCAGCCTGAGTAAAAGGAGCAGGTGGGAAACAGCGTAAGGCATTAAGGAAACTAGACCACTGTTACTGGCGGTGGAATGGGAAGACAAAACATGCTGCcttgaaaatgaaacatcacTTAATTAATTTCAAGTGAGGACGGCTAAAACAAGAACCAACACCAAGATGCACCTAACTTTCCCAGGgatgaatttcttttcaatatCCTCTAATGAACTGTAAGGGAAAGACCCTGAAAACAAGAATGCCCATACAGACACTGGCTTCACTAGGTGAGTCTGGAAAATGAACACAAGTGCATTATCCAGTCCTACGGCATTAAGCACAGAATCACATCCTAAACTGTAAGTTAAAAGCATAAGTCAAAAATTCTCATCAGCAGATGCAAACGTGAAGATGCACACTCACACCAACCCCCGGTTTATGTCTCAGAATCCCCTGGTACTTATATAGTCACACAAGGACATTAACAACAAGAGACTGTACCTCATCCCAGGaagattctcttcctctctcttctgtaaagtggcGCACTGAcacaccatctgtaaaatgtagttaCAGGTACATTAATGAGAATATTCACGAATGTATATTAAAACCACCATGTGCGTCTATCTCCGTTCGTGGATGTGTCAAAACAAAAGTGGCAGAAAAGACTTTCAAAGAGTTagagcattttctgaaaaaaatgcttaagtcACGGTTGGGATATATTTCAAACCAAGTAGTAAGAGGAAAACACGTAAAAAGAATGGATATGGAAGTTGGGTTTGCGCACTATGTATTTCTAACATTTGAagtttgctcttgtttttgttttttcttttcttctgtttttaaatggaggtaccgggaaTGAGCACACGGAATTCggcatgctaagcaagcattcTATTTACCACTAACCTATGTAATTCCCCGAGTCAACTAGCTTCTCGgcatagataaaataaaatacaaaacactcTTTCACGGTTCACTGCCTTACAAAACATGTTAGTTTGCTGAAGCGCAAAAGATCTTTGAGGCCTTCTTCCCCGTAACACACTAAGTCTCACATCAGACCGAGTGGGAGACAGGTCTCAGGACCCTTGACTGAAGAGACCACAGGCCCCGGGTTCGGGTGTGTCTTCCATAACCGATCATCGGCTGTGGAAGTT |
| LNC002465 | GTGCCCTGTGCAACCTCGCCCGCCACCGCCAGCTGCTCCGCTCGGTAAGAGGAAAGCAtagtcctctgtttcttccttgagtCACTGCAGAAGAAGGGCAGATCGCTCTCCGGGGCTTCCCCGTGCACGGGCGGCCGTGACTGGGCTCACGGCCGTGTCCCTCTAGTCCCTTAGCAGACGCACTTGATGGACCGCGTCTTCCCcgtcctgccctggggagggaggtgtctgTTGTAGAGGGAAAGAGGGTCCTCTGACGGAGGGCAGACGTGTCGGTTCTGCACACGGGCCTTCAACAGACACCACGTTCCGGGCTCCCTGGAGGACAAGCCCGCCCCTGTCACTGCAGCGAGTGGAGCCCTGAACTTCCACAGCCGATGATCGGTTATGGAAGACACACCCGAACCCGGGGCCTGTGGTCTCTTCAGTCAAGGGTCCTGAGACCTGTCTCCCACTCGGTCTGATGTGAGACTTAGTGTGTTACGGGGAAGAAGGCCTCAAAGATCTTTTGCGCTTCAGCAAACTAACATGTTTTGTAAGGCAGTGAACCGTGAAAgagtgttttgtattttattttatctatgcCGAGAAGCTAGTTGACTCGGGGAAGGACATAGGTTAGTGGTAAATAgaatgcttgcttagcatgccGAATTCCGTGTGCTCAttcccggtacctccatttaaaaacagaagaaaagaaaaaacaaaaacaagagcaaactTCAAATGTTAGAAATACATAGTGCGCAAACCCAACTTCCATATCCATTCTTTTTACGTGTTTTCCTCTTACTACTTGGTTTGAAATATATCCCAACCGTgacttaagcatttttttcagaaaatgctctAACTCTTTGAAAGTCTTTTCTGCCACTTTTGTTTTGACACATCCACGAACGGAGATAGACGCACATGGTGGTTTTAATATACATTCGTGAATATTCTCATTAATGTACCTGTAac |
| LNC001684 | CTTCCTAGACCTTCGATGAGTAAGGAGGCTCACTGCTCATCCTCGATCCCTTTGCTGGTGGCTAAAAGGGACCAAGGCCCACGAGAGCGCTTCTGGGAGCTGTCTGCCGAAACCTGGAACAGGACTGCTCAGGACTGGCTCTGCAGCAGGAGCGAAGGGAGGCAATGTCGCCACCTGGTGGACATCCTGGGTAGTGGCGGTAGGAGGCCTAGCACCCTTGGTATGGAGGTGGCAGGACGAGGTCCTACGGGATGATCGTTTCCACGGgtcttaaaaatcattgcttGATATATTTTATCCGCTTTGGGGTTTACTGTAGGATGGTAAATATGGTCTCCGTTACTCCTTCTTAGCTGGATTGATGAATTGTTTTTGAggttaattttctcttcagtgatttgGAAGCTATATTGCCTTTTAAGGAAAATTCTCtctaaatgtttataaatcatcTCGAAGTTCATAACTCTATATTTAATTCGACCAGTATTGCTACAAGAGCCTCAAAAACGAAATCATCTATTAGATCTTGTTTCTCAATCATGATACATTTAGCATATTTtacctctctgctcttcccaatggtgcgtgtgtgtgagcaCG |
[truncated: 190,562 more chars]
